# Tree diversity and mycorrhizal type independently shape multitrophic biodiversity, with stronger effects belowground than aboveground

**DOI:** 10.1038/s41559-026-03147-6

**Published:** 2026-08-31

**Authors:** Huimin Yi, Olga Ferlian, Phillip J. Becker, Henriette Christel, Yuanyuan Huang, Michael Köhler, Ina C. Meier, Hafeez Ul Haq, Tesfaye Wubet, Nico Eisenhauer

**Affiliations:** 1 German Centre for Integrative Biodiversity Research (iDiv) Halle-Jena-Leipzig, Leipzig, Germany; 2 Institute of Biology, Leipzig University, Leipzig, Germany; 3 Functional Forest Ecology, University of Hamburg, Hamburg, Germany; 4 Institute of Biology/Geobotany and Botanical Garden, Martin Luther University Halle-Wittenberg, Halle (Saale), Germany; 5 Department of Community Ecology, Helmholtz Centre for Environmental Research (UFZ), Halle (Saale), Germany

**Keywords:** Ecosystem ecology, Forest ecology, Food webs

## Abstract

Tree diversity declines and shifts in mycorrhizal dominance may have cascading effects on multitrophic diversity. Whether the effects are additive or synergistic and consistent across taxa and functional groups is unclear. Here we test how microorganisms and invertebrates spanning multiple trophic levels, both above- and belowground, respond to experimental manipulation of tree species richness and mycorrhizal type in a young forest stand (the MyDiv experiment in Germany). Tree diversity increased belowground abundance and taxonomic richness of microorganisms and lower-trophic-level nematodes, cascading to meso- and macrofauna predators, whereas aboveground effects were limited to foliar fungi and soil-surface omnivores. Mycorrhizal type strongly structured belowground food webs and influenced aboveground predators, but mixtures showed only additive effects. For most trophic groups, diversity responses were similar across mycorrhizal types. Tree diversity and mycorrhizal type effects were mediated by leaf quality, canopy complexity and nutrient availability. Our results highlight tree diversity and mycorrhizal type as potentially distinct drivers of multitrophic biodiversity, primarily governing belowground bottom-up effects.

## Abstract (web summary)

Tree diversity and mycorrhizal dominance are considered major drivers of multitrophic diversity and ecosystem functioning in forests. Here the authors disentangle the effects of these factors on above- and belowground trophic levels in a tree diversity–mycorrhiza diversity experiment.

## Main

Forests support over 80% of terrestrial biodiversity but have been widely degraded by human activities^1,2^, disrupting global carbon and nutrient cycles^3^. Although global forest restoration has accelerated, it is often dominated by monocultures^4^, which limits primary productivity^5^, homogenizes habitats and contributes to biodiversity loss^6,7^. Whereas vertebrates have received considerable attention^8^, microorganisms and invertebrates, driving key ecosystem functions such as pest control, nutrient and carbon cycling^9–11^, remain understudied. Belowground communities, which host ~59% of global biodiversity and support aboveground life^12,13^, have received even less attention. An integrated above–belowground perspective is essential to reveal how tree diversity underpins forest biodiversity and ecosystem functioning, guiding more effective restoration and conservation^11,14,15^.

Biodiversity is structured into food webs of interacting trophic groups, and understanding the diversity across trophic levels provides insights into community structure and ecosystem functioning beyond taxonomic patterns^16,17^. Within this framework, changes in tree communities can propagate to higher trophic levels along the food chain (bottom-up effects)^18^. More specifically, tree mixtures can increase canopy complexity and productivity through spatial and resource partitioning^19,20^, benefiting aboveground herbivore abundance^21^. Meanwhile, greater allocation of photosynthates to roots promotes root growth, mycorrhizal colonization and exudation^22,23^, thereby increasing the abundance of belowground herbivores and microorganisms^10,24^. Moreover, plant communities shape soil nutrient cycling, which structures soil microbial and nematode assemblages^24,25^. The increased total abundance of organisms is more likely to support more taxa^26^, and tree mixtures may reduce the dominance of individual species^27,28^. These cascading effects ultimately influence populations of microbial feeders and predators^18,21^. Thus, lower trophic levels may respond more strongly than higher trophic levels^18,29^, whereas more mobile aboveground organisms may react faster to local changes in plant diversity than belowground biota, such as shown for grasslands^18^. Therefore, investigating the responses across trophic levels provides a comprehensive perspective and mechanistic insights into how plant diversity shapes above- and belowground communities^30,31^. Yet these links remain insufficiently tested in forests^10,15^.

Symbiosis with arbuscular mycorrhizal (AM) and ectomycorrhizal (EcM) fungi influences soil-nutrient cycling and life strategies of host trees, with potential cascading effects on associated microbial and invertebrate communities^22,32–35^. AM fungi enhance uptake of inorganic nutrients from mineral soil layers, whereas EcM fungi access organic nutrient pools in the top soil layer^22,36^. Complementary resource-use strategies may partition soil nutrients and space, such that mixed AM and EcM (AM+EcM) forests can be more productive than single-type forests^37^. Such overyielding may reflect synergistic effects, whereby the benefits of mixing different mycorrhizal types exceed those expected from additive effects alone. This elevated productivity may in turn support higher consumer abundances, particularly among generalist taxa in soil food webs^38^. Moreover, AM trees generate nutrient-rich, fast-decomposing litter that supports bacteria and bacterivores, whereas EcM litter favours fungi, fungivores and mesodetritivores^32–34^; and EcM roots experience reduced herbivory due to protection by the fungal mantle^35^. Together, these contrasts indicate that mycorrhizal type may structure soil food webs by altering multiple energy channels. Such differences may also extend aboveground, as AM trees typically prioritize growth over defence and experience higher herbivore pressure than EcM trees^32,39^. Consequently, mycorrhizal mixtures may also generate synergistic effects on the diversity of both above- and belowground communities by broadening the trophic niches^24,40^. Because mycorrhizal-mediated processes act primarily in soils, synergistic effects are expected to be strongest belowground and at lower trophic levels. As climate warming, drought, and anthropogenic pressures shift mycorrhizal dominance in forests^41–43^, empirical tests are urgently needed to predict biodiversity responses across trophic levels.

Increasing evidence shows that tree-diversity effects on ecosystem functioning are amplified by functional trait differentiation among tree species^44–47^. Accordingly, tree-diversity effects are expected to be stronger in mixed mycorrhizal communities than in stands dominated by a single mycorrhizal type. However, empirical support for interactive effects between tree diversity and mycorrhizal types remains largely confined to tree productivity^48^. Beyond the tree community, existing evidence has mainly focused on individual taxonomic groups in isolation (for example, microbes^49,50^ and nematodes^51^). Therefore, an integrated multitrophic approach is required to disentangle the effects of mycorrhizal types and their interactions with tree diversity across the entire food web.

We used the MyDiv experiment^52^ to test how tree diversity and mycorrhizal type influence biodiversity across trophic levels above- and belowground. We sampled microorganisms and invertebrates from the canopy, the soil surface and the soil. Sampling was conducted at the start and end of the growing seasons, allowing us to capture seasonal dynamics and to identify treatment effects that remained consistent across seasons. Nearly 100,000 invertebrate individuals were identified and assigned to trophic groups. Biodiversity facets were quantified for each trophic group, and the biomass and alpha diversity of soil bacteria and fungi, along with foliar fungal diversity, were assessed. We also measured potential mediators of tree diversity and mycorrhizal effects: tree-wood volume, canopy complexity and nutrient concentrations in leaf, root and soil. Tree diversity, mycorrhizal type and their interaction effects were tested using mixed-effects models. Structural equation models (SEMs) identified key mediators of tree diversity and mycorrhizal effects on multitrophic abundance and diversity, with backwards selection applied to determine the most influential variables for each trophic group. We tested four hypotheses: (1) Increasing tree species richness enhances the abundance and diversity of microorganisms and invertebrates, especially (a) at lower trophic levels and (b) in the aboveground compartment. (2) Mixing mycorrhizal types increases the abundance and diversity of microorganisms and invertebrates, with stronger effects (a) at lower trophic levels and (b) in the belowground compartment. (3) Positive effects of tree species richness on the abundance and diversity of microorganisms and invertebrates are amplified in communities with mixed mycorrhizal types. (4) These positive effects of tree diversity and mycorrhizal mixture are mediated by increases in tree biomass, canopy complexity and nutrient availability in leaves, roots, and soil.

## Results and discussion

### Tree diversity promotes belowground more than aboveground biodiversity

**Fig. 1:**
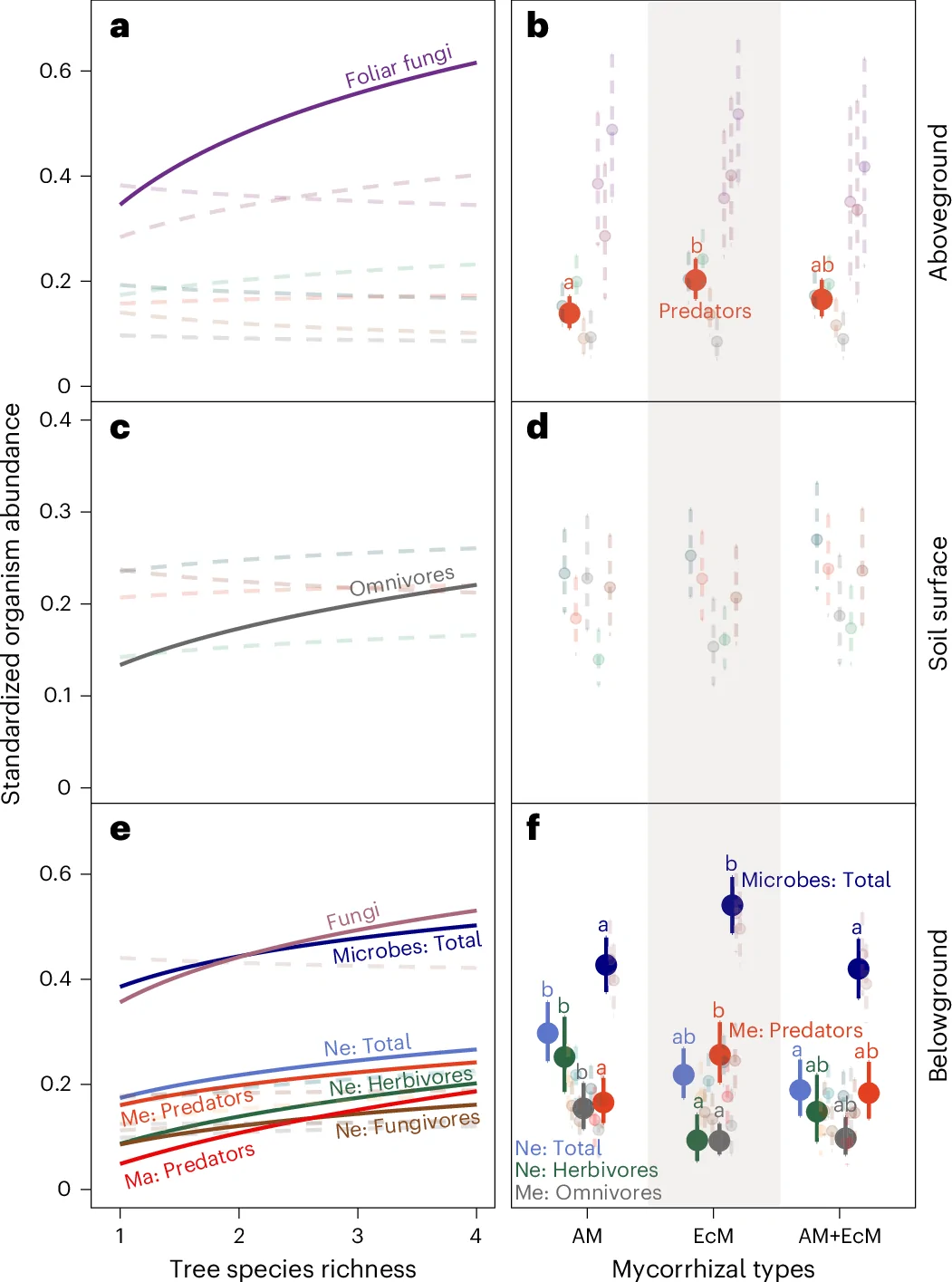
Effects of tree species richness and mycorrhizal types on organism abundances. **a**–**f**, Effects of tree species richness and mycorrhizal types, respectively, aboveground (**a**,**b**), at the soil surface (**c**,**d**) and belowground (**e**,**f**). All response variables were scaled to [0,1]. Linear mixed-effects models with two-sided tests were fitted separately for each trophic group within each stratum using plot-level observations. Most models included 160 observations from 80 plots sampled twice; foliar fungal groups were sampled once with fewer plot-level observations (endophytes, *n* = 39; total foliar fungi and epiphytic fungi, *n* = 34 each). **a**,**c**,**e**, Prediction across the tree species richness gradient; solid lines indicate significant effects (*P* < 0.05), whereas dashed transparent lines indicate non-significant effects. Estimated means and 95% confidence intervals for each mycorrhizal type (**b**,**d**,**f**) were obtained using emmeans from the fitted models. Solid dots and letters (a, b, ab) denote significant differences among mycorrhizal types after Tukey adjustment (*P* < 0.05); transparent dots indicate non-significant differences; the EcM column is given in grey shading. Colours indicate trophic groups, with labels shown only for significant responses to tree species richness or mycorrhizal type. Belowground organisms are abbreviated Ne (nematodes), Me (mesofauna) and Ma (macrofauna). See Supplementary Tables 1–3 for power transformation values and Supplementary Tables 4–8 for *F*-values, *P* values, degrees of freedom and model *R*^2^ of each model.

**Fig. 2:**
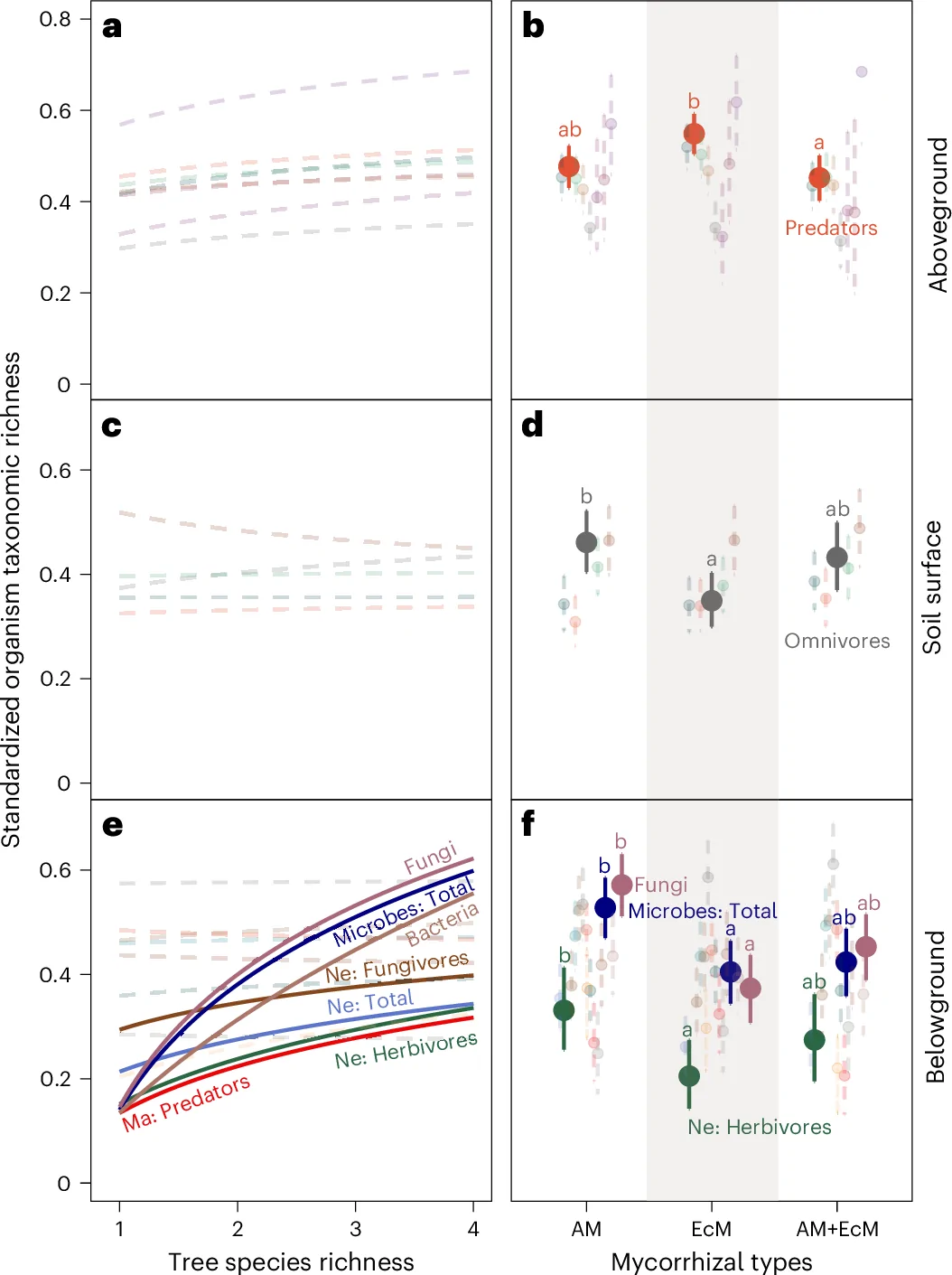
Effects of tree species richness and mycorrhizal types on organism taxonomic richness. **a**–**f**, Effects of tree species richness and mycorrhizal types, respectively, aboveground (**a**,**b**), at the soil surface (**c**,**d**) and belowground (**e**,**f**). All response variables were scaled to [0,1]. Linear mixed-effects models with two-sided tests were fitted separately for each trophic group within each stratum using plot-level observations. Invertebrate models included 160 observations from 80 plots sampled twice. Soil microbial groups were sampled once across 80 plots, and foliar fungal groups were sampled once with fewer plot-level observations (endophytes, *n* = 39; total foliar fungi and epiphytic fungi, *n* = 34 each). **a**,**c**,**e**, Prediction across the tree species richness gradient; solid lines indicate significant effects (*P* < 0.05), whereas dashed transparent lines indicate non-significant effects. Estimated means and 95% confidence intervals for each mycorrhizal type (**b**,**d**,**f**) were obtained using emmeans from the fitted models. Solid dots and letters (a, b, ab) denote significant differences among mycorrhizal types after Tukey adjustment (*P* < 0.05); transparent dots indicate non-significant differences; the EcM column is given in grey shading. Colours indicate trophic groups, with labels shown only for significant responses to tree species richness or mycorrhizal type. See Supplementary Tables 1–3 for power transformation values and Supplementary Tables 4–8 for *F*-values, *P* values, degrees of freedom and model *R*^2^ of each model.

Tree diversity (that is, tree species richness) significantly increased the abundance of belowground invertebrates and microorganisms across trophic levels (Fig. 1e and Supplementary Tables 7–8). However, the abundance of aboveground and soil-surface trophic groups showed only limited responses, with significant effects restricted to total foliar fungi (*F*_1,22_ = 4.6, *P* = 0.04; Fig. 1a and Supplementary Table 5) and soil-surface omnivores (*F*_1,54_ = 4.52, *P* = 0.04; Fig. 1c and Supplementary Table 6). A similar pattern was observed for taxonomic richness: tree diversity increased the richness of most belowground trophic groups (Fig. 2e and Supplementary Tables 7–8). Only a marginally significant effect of tree diversity was observed on total aboveground taxonomic richness (*F*_1,54_ = 3.8, *P* = 0.06; Fig. 2a and Supplementary Table 4), whereas no significant effects were detected for soil-surface fauna (Fig. 2c and Supplementary Table 6). The strongest tree diversity effects were observed in the belowground compartment, which contrasts with our expectation that plant diversity effects would be stronger aboveground (hypothesis (1b)). This pattern was supported by the analysis of log-response ratios comparing predicted values of polycultures with monocultures (Supplementary Figs. 11–13). The log-response ratios for the majority of aboveground and soil-surface groups had 95% confidence intervals overlapping zero, whereas most belowground groups showed positive responses with confidence intervals excluding zero (Supplementary Figs. 11 and 13).

The results may partly reflect differences in dispersal limitation and resource stability between above- and belowground systems. Increased aboveground productivity at higher tree diversity^19^ likely leads to greater carbon inputs belowground through root growth^53^, rhizodeposition^54,55^ and mycorrhizal associations^22,36^, thereby enhancing resource availability for belowground communities. Tree diversity probably also increases belowground resource heterogeneity via these pathways^56^. Belowground resources are often spatially structured, and soil organisms typically exhibit lower mobility than organisms in higher strata, making them more strongly influenced by local resource availability and plot-level environmental conditions^57^. This localized resource dependence could lead to stronger bottom-up effects^58^, promoting consumer abundance and diversity at higher trophic levels (as discussed below). In contrast, aboveground organisms often exhibit stronger host specialization (for example, herbivores) and greater mobility (for example, predators), making consumer-community structure more dependent on tree-species identity or functional traits rather than plot-level conditions^59,60^. In addition, tree diversity-driven differences in resource inputs to soils may accumulate over time^13^, leading to stronger biodiversity responses belowground than in higher strata, where communities are more strongly influenced by seasonal dynamics (Supplementary Tables 4–6). Notably, field samples taken at or near canopy closure (in May and September) may underestimate aboveground effects, as reduced variation in light and microclimate can dampen tree-diversity responses. Although the mechanisms we refer to above are well established, their role in our study remains inferential and should be tested directly in future work.

Our results did not show consistently stronger effects on the abundance or taxonomic richness of lower than higher trophic levels across invertebrates and microorganisms (Figs. 1 and 2 and Supplementary Figs. 11 and 13; hypothesis (1a)). Aboveground and soil-surface responses to tree diversity were limited to foliar fungi and soil-surface omnivores, providing no consistent support for stronger effects at lower trophic levels. In the belowground compartment, the strongest effects on abundance were observed on macrofauna predators (*F*_1,56_ = 11.6, *P* = 0.003), followed by soil fungi (*F*_1,54_ = 8.5, *P* = 0.005), nematode herbivores (*F*_1,56_ = 7.9, *P* = 0.008) and fungivores (*F*_1,56_ = 5.64, *P* = 0.02), as well as mesofauna predators (*F*_1,56_ = 4.87, *P* = 0.03; Fig. 1e and Supplementary Tables 7 and 8). Moreover, tree diversity most strongly affected the taxonomic richness of soil fungi (*F*_1,56_ = 83.0, *P* < 0.001) and bacteria (*F*_1,56_ = 62.3, *P* < 0.001), with smaller effects on macrofauna predators (*F*_1,56_ = 10.2, *P* = 0.002), nematode herbivores (*F*_1,56_ = 11.2, *P* = 0.001) and fungivores (*F*_1,56_ = 4.2, *P* = 0.04) (Fig. 2e and Supplementary Tables 7 and 8). The magnitude of increase among these trophic groups did not differ significantly, as indicated by the overlap of their 95% confidence intervals for effect sizes (log-response ratios of predicted values in polycultures relative to monocultures) (Supplementary Fig. 11).

In the belowground food web, the effects of tree diversity on soil fungi, fungivorous and herbivorous nematodes, as well as top predators, may reflect bottom-up effects^18^. However, this pattern was not observed for lower-trophic-level meso- and macrofauna groups. The increase in fungivorous nematode abundance under higher tree diversity was linked to increased soil fungal biomass, whereas the lack of response in bacterivorous nematodes was consistent with unchanged bacterial biomass. Aligning with previous studies^49,61^, fungal diversity increased with tree diversity, which may broaden trophic niches and support greater taxonomic richness of nematode fungivores^24^. Nematode herbivores probably benefited from greater tree productivity^62^ and a larger host pool in tree polycultures. The abundance and diversity of mesofauna and macrofauna predators was likely linked to greater prey availability and diversity, such as nematodes^10,56,63^. In contrast, lower-trophic-level meso- and macrofauna groups, such as fungivores, detritivores (including earthworms) and omnivores, did not respond significantly to tree diversity (Figs. 1 and 2). They may not have responded to tree diversity as rapidly as microorganisms and nematodes with high turnover rates or predators with greater mobility. Meanwhile, their populations were likely suppressed by the increased abundance of predators (top-down effects)^29^. Cautiously, for consistency, we assigned soil meso- and macrofauna to trophic groups based on established classifications; however, this did not capture trophic plasticity driven by resource availability and environmental conditions^64^. Many soil taxa, especially mesofauna (for example, oribatids), are omnivorous and often were identified at low taxonomic resolution; thus, grouping them into broad trophic categories likely limited our ability to detect diversity responses^65^. To directly test the mechanisms proposed to explain the observed patterns, future studies should manipulate resource heterogeneity^66,67^ and use stable-isotope analysis^68^, isotope labelling and molecular analyses of consumer tissues^69^ and gut contents to trace resource transfer through the food web^70^.

Overall, our findings underscore that tree diversity exerts stronger effects on belowground communities, across multiple trophic groups, than on aboveground and soil-surface communities. Moreover, the abundance and diversity of microorganisms and invertebrates showed similar responses to tree diversity. This suggests that tree mixtures do not simply favour specific taxa but also reduce dominance, foster coexistence and potentially enhance community stability (Supplementary Fig. 8)^21,71^. By integrating above- and belowground food webs, we provide empirical support for diversifying future forests to enhance soil biodiversity that is vital for carbon and nutrient cycling, aboveground predator support and ecosystem multifunctionality^4,10,11,14^.

### Mycorrhizal types structure microbial and invertebrate communities

The abundances of microorganisms and invertebrates in AM+EcM communities consistently fell between those of AM and EcM tree communities across above- and belowground compartments (Fig. 1b,d,f). A similar pattern was observed for taxonomic richness (Fig. 2b,d,f). These findings contrast with our expectation (hypothesis 2) that mixing mycorrhizal types would enhance microorganism and invertebrate abundance and diversity. For abundance, this lack of support is consistent with the previously reported absence of synergistic effects on tree productivity in AM+EcM tree communities^62^. Six years after establishing the MyDiv experiment may not be long enough for such effects to emerge^10,19,62,72^, whereas Luo et al. ^37^ reported synergistic productivity effects in mature forests. Additionally, the use of weed tarps may suppress potential gains in taxonomic richness of soil-surface and belowground organisms^10,52^ by reducing resource and microhabitat heterogeneity that is typically driven by the distinct litter traits of AM and EcM tree species^32,56^. Ray et al.^19^ reported that mycorrhizal mixtures in MyDiv did also not generate any synergistic effects on tree-canopy complexity, a structural feature linked to microhabitat and refuge availability.

Nonetheless, AM and EcM tree communities supported distinct belowground food webs, and their effects extended to the abundance and diversity of aboveground predators. However, mycorrhizal type did not exert stronger effects on the abundance of lower trophic levels belowground. For example, AM-tree communities supported the highest abundance of total nematodes (*F*_2,54_ = 3.5, *P* = 0.039), nematode herbivores (*F*_2,54_ = 8.9, *P* = 0.005) and mesofauna omnivores (*F*_2,54_ = 3.4, *P* = 0.041), whereas EcM tree communities had the highest soil microbial biomass (*F*_2,54_ = 5.5, *P* = 0.006) and mesofauna predator abundance (*F*_2,54_ = 3.5, *P* = 0.038) (Fig. 1f and Supplementary Tables 7 and 8). Effects on taxonomic richness were limited to lower trophic levels, with richness being highest in AM-tree communities for total soil microbes (*F*_1,54_ = 82.8, *P* < 0.001), soil fungi (*F*_1,54_ = 79.2, *P* < 0.001) and nematode herbivores (*F*_2,54_ = 4.2, *P* = 0.020; Fig. 2f). Soil microbial biomass was higher in EcM-tree communities, probably due to a larger proportion of photosynthates allocated to belowground compared to the AM trees^73^, despite expectations that the acquisitive traits of AM trees would favour these groups^34^. The elevated microbial and fungal taxonomic richness in AM-tree communities was driven by the enrichment of saprotrophs, pathogens and mycorrhizal fungi under AM-dominated tree assemblages (Supplementary Fig. 5), in line with previous studies^74,75^. AM plots harboured the highest abundance and richness of nematode herbivores, likely reflecting a lack of physical protection of AM-tree roots^35^. The highest abundance of mesofauna predators was found in EcM-tree communities, instead of AM-tree communities with more abundant prey (for example, nematodes). Bönisch et al.^76^ showed that EcM trees at MyDiv exhibited stronger nutrient resorption than AM trees, resulting in lower nitrogen concentrations in EcM-leaf litter. Thus, the lower-quality EcM litter accumulates at the soil surface and creates shelters to protect soil invertebrates from larger predators, such as beetles and spiders at the soil surface^56^. Our results show that AM communities supported the highest microbial and nematode diversity, broadly consistent with previous studies linking AM dominance to higher plant diversity in mature forests^35,77^. Therefore, the mycorrhizal-type effects on belowground diversity may extend to large-scale, mature forests, although further empirical evidence is needed.

Mycorrhizal-type effects also extended to aboveground organisms: EcM-tree communities supported higher abundances (*F*_2,54_ = 3.8, *P* = 0.029) and diversity (*F*_2,54_ = 4.0, *P* = 0.025) of predators than AM communities (Figs. 1b and 2b). EcM-tree species, such as oak and beech trees, with dense branch systems and later leaf senescence, provide greater and more stable habitats for predators, favouring both their abundance and diversity^78^. Another possible explanation is that stronger defence investments in EcM trees trigger predator-attracting cues, so-called ‘cry for help’ signalling, although this assumption remains to be experimentally validated^79^. Contrary to our expectations, mycorrhizal type had no significant effects on abundance or taxonomic richness at lower trophic levels (Figs. 1 and 2 and Supplementary Tables 4 and 5). This unexpected pattern was consistent with little response of aboveground organisms to tree diversity (Figs. 1a,b and 2a,b)^29^. Different organism groups were favoured by AM- and EcM-tree communities, which may explain the lack of synergistic effects in AM+EcM mixtures. Temperate forests are shifting towards AM dominance at the expense of EcM trees due to anthropogenic influences and climate change^41–43^. Our results provide empirical evidence to predict how such shifts may affect multitrophic diversity across above- and belowground food webs. Further observational studies are needed to assess biodiversity changes along such gradients, which underpin ecosystem functioning and conservation.

### No interactive treatment effects for most trophic groups

Our results show that the interaction between mycorrhizal type and tree diversity had no significant effects on any biodiversity index across trophic groups or total communities in the aboveground and soil-surface compartments (Supplementary Tables 4–6). Similar patterns held for soil microorganisms and nine out of twelve belowground invertebrate groups (Supplementary Tables 7 and 8), indicating that tree-diversity effects on the abundance and diversity across multiple trophic groups were likely independent of mycorrhizal type. This contradicts our hypothesis (3), which predicted that tree-diversity effects on multitrophic biodiversity would be amplified by mycorrhizal mixtures. Our findings, however, are consistent with those of Ray et al.^19^, who reported that tree diversity increased productivity regardless of mycorrhizal types. Notably, strong sampling and composition effects might limit the further detection of interaction effects, leading to the non-significant results^10,71^.

Only three belowground trophic groups showed significantly differential responses to tree diversity across mycorrhizal types (Supplementary Fig. 10 and Supplementary Table 7). In AM and EcM communities, the abundance and taxonomic richness of nematode herbivores significantly increased with tree diversity in AM and EcM plots, but this trend reversed in AM+EcM mixtures, possibly due to strong dilution effects^35,80^. This suggests that mixing mycorrhizal types may reduce root damage, confirming findings from Yi et al.^72^ for foliage damage aboveground. Soil-macrofauna predators increased in abundance with tree diversity only in EcM-tree communities (Supplementary Fig. 10b); their taxonomic richness increased in EcM and AM+EcM communities but not in AM communities (Supplementary Fig. 10d). These results are consistent with Yi et al.^10^, where enhanced belowground predation occurred only in EcM plots. The increase in soil-macrofauna predator richness in EcM-tree communities likely contributed to the overall rise in total soil-macrofauna richness (Supplementary Fig. 10e). Nematodes, with fast turnover and reproduction rates and mobile macrofauna predators, seemed to respond earlier to tree diversity with different mycorrhizal types, consistent with their strong responses to each factor individually (as discussed previously). Interaction effects in other groups may take longer to emerge^81^. The effects of tree diversity on root feeders and predators depended on mycorrhizal type. Incorporating EcM-tree species into AM-dominated plantations may enhance predator abundance and diversity across above- and belowground food webs, thereby reducing herbivory and disease risk in AM-tree stands.

### Leaf quality, canopy complexity and nutrient contents mediate tree diversity effects

**Fig. 3:**
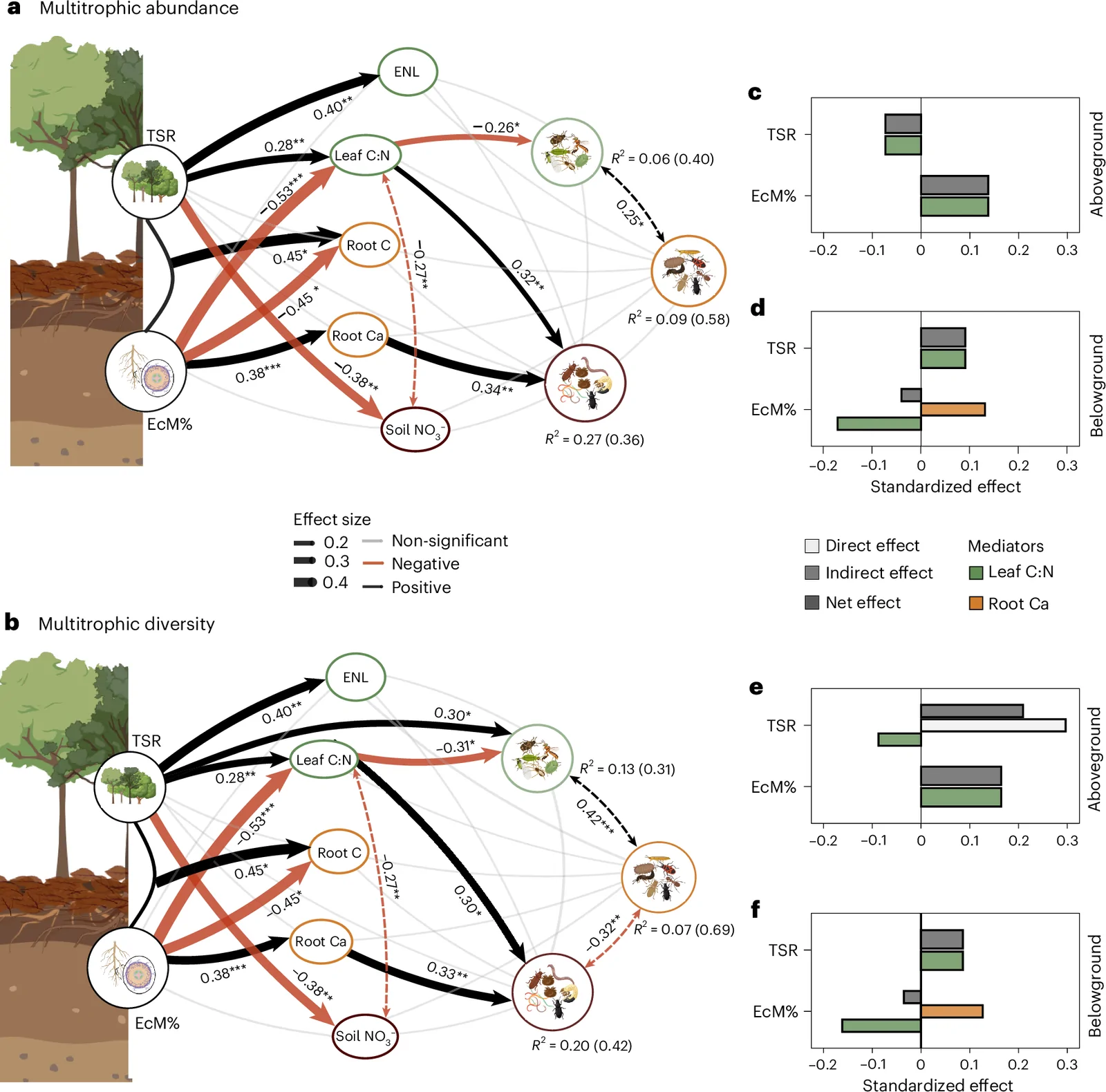
Structural equation models on multitrophic abundance and diversity. **a**,**b**, How tree species richness (TSR) and mycorrhizal type (proportion of EcM-tree species (EcM%)) affect multitrophic abundance (**a**) and diversity of invertebrates (**b**). The invertebrates are grouped by strata: aboveground (green bubbles), soil surface (orange bubbles) and belowground (brown bubbles) strata are displayed on the right side of each model. Treatment effects are mediated by canopy complexity (ENL), leaf C:N, root C and Ca and soil NO_3_^−^. Model fit: abundance (Fisher’s *C* = 35.63, d.f. = 44, *P* = 0.812); diversity (Fisher’s *C* = 32.52, d.f. = 42, *P* = 0.85). **c**–**f**, Total (direct + indirect) effects. SEM results for abundance and diversity, respectively, across strata aboveground (**c**,**e**) and belowground (**d**,**f**). Significant effects (*P* < 0.05) are shown as solid black (positive) or red (negative) lines; non-significant paths are shown in grey. Standardized path coefficients are shown with significance levels (***P < 0.001, **0.001 ≤ *P* < 0.01, *0.01 ≤ *P* < 0.05). Interactive effects of tree species richness and mycorrhizal type on root C are shown by a connecting line between two icons. Path coefficients were tested using two-sided tests. Marginal *R*^2^ values for each final endogenous variable are noted next to their icons (conditional *R*^2^ values in brackets). Conditional *R*^2^ values for mediating variables are reported in Supplementary Table 11. The multitrophic abundance and diversity were calculated as the mean of min–max transformed values for each trophic group within the corresponding stratum. The sensitivity analysis based on the *Z*-score transformation is shown in Supplementary Fig. 15 and Supplementary Table 12. Icons in **a** and **b** created in BioRender; Eisenhauer, N. https://biorender.com/jvnwool (2026).

**Fig. 4:**
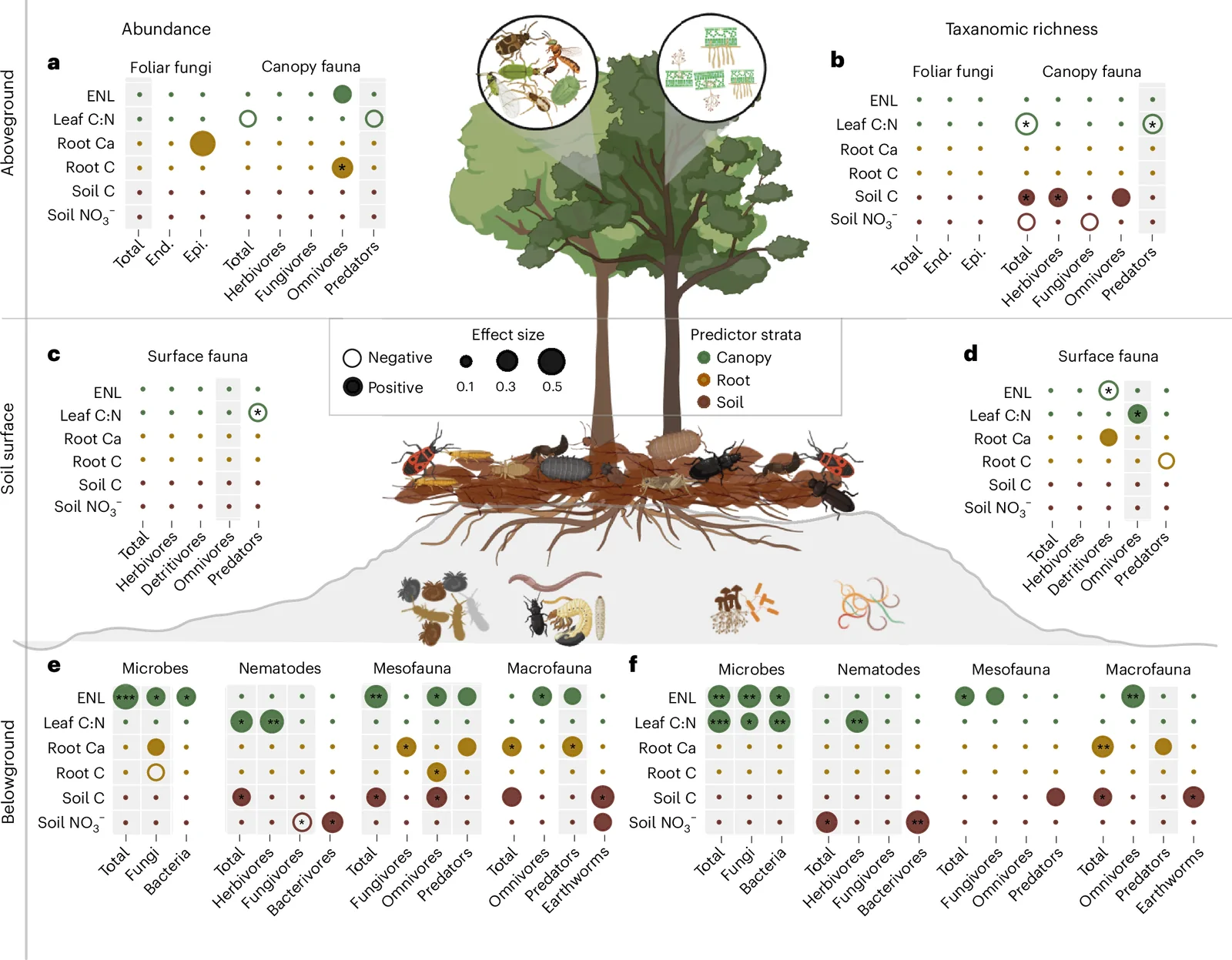
Results of multiple linear regressions. **a**–**f**, Effects of six mediators on standardized organism abundance and taxonomic richness, respectively, aboveground (**a**,**b**), at the soil surface (**c**,**d**) and belowground (**e**,**f**). Within each panel, individual trophic groups are shown as columns grouped into broader categories (for example, foliar fungi, canopy fauna, microbes, nematodes, mesofauna, macrofauna) labelled at the top. Linear mixed-effects models with two-sided tests were fitted separately for each diversity index of each trophic group within each stratum, using all six proposed explanatory variables. Only variables retained in the best-fit models after backwards selection with the lowest AICc are shown. Standardized coefficients of retained explanatory variables are represented by circles, with filled circles indicating positive effects and open circles indicating negative effects; circle size is proportional to the absolute standardized coefficient. Non-selected variables are shown as small dots. Statistical significance is indicated by asterisks: ***P < 0.001, **0.001 ≤ *P* < 0.01, * 0.01 ≤ *P* < 0.05; no asterisk indicates marginal significance (0.05 ≤ *P* < 0.1). Colours indicate the source of each explanatory variable: green for canopy conditions (leaf C:N ratio, ENL), yellow for root nutrients and brown for soil conditions. The model structure and performance are presented in Supplementary Tables 15 and 16. Panels are shaded when the biodiversity response was significantly affected by tree species richness and/or mycorrhizal type (see Figs. 1 and 2). Icons created in BioRender; Eisenhauer, N. https://biorender.com/jvnwool (2026).

Utilizing structural equation modelling and integrating multitrophic abundance and diversity metrics, we tested hypothesis (4) stating that the effects of tree diversity and mixed mycorrhizal types are mediated by increasing tree biomass, canopy complexity (effective number of layers (ENL)), nutrient levels in leaves and roots, and soil properties. Aboveground multitrophic abundance and diversity were negatively correlated with leaf carbon-to-nitrogen ration (C:N) (Fig. 3 and Supplementary Fig. 15), a pattern largely driven by predators (Fig. 4a,b). EcM plots had lower leaf C:N, indicating higher leaf N than AM plots (Supplementary Fig. 6). The N-rich EcM-tree leaves may attract predators via enhanced volatile emissions, such as those of linalool and indole^79,82^, thereby supporting the highest abundance and diversity of predators. However, tree diversity had a direct positive effect on aboveground multitrophic diversity (Fig. 3b), independent of all mediators, probably due to the greater tree species pool attracting more specialists^83,84^. This aligned with the fact that tree composition accounted for the largest share of variation in aboveground organisms (Supplementary Table 4 and Figs. 1 and 2), aligning with prior findings in tree-diversity studies^59,85^. All mediators were assessed during the growing season. This may have limited our ability to capture mechanisms by which aboveground organism responses were driven by phenological variation among mycorrhizal types and tree species^76^. No mediators correlated with multitrophic abundance and diversity on the soil surface; the multitrophic metrics were rather explained by the random factor of tree composition (Fig. 3 and Supplementary Fig. 15), which was further confirmed for each trophic group (Fig. 4c,d and Supplementary Table 16). We acknowledge that limited data on litter quality and its heterogeneity constrained our ability to resolve how tree diversity influences soil-surface fauna^56^.

Belowground multitrophic abundance increased with tree diversity, partly via leaf C:N (Fig. 3a,d and Supplementary Fig. 15). Moreover, leaf C:N negatively and root calcium (Ca) positively mediated the effects of EcM-tree species on multitrophic abundance, resulting in a relatively weak net mycorrhizal effect. The same mediating effects occurred for belowground multitrophic diversity (Fig. 3b,f and Supplementary Fig. 15), supported by the More Individual Hypotheses^26^, and echo findings that vegetation effects on consumer diversity are mediated by consumer abundance^86^. Increased leaf C:N with tree diversity suggests greater carbon allocation to shoot growth over root defence^87^, thereby supporting more abundant and diverse herbivorous nematodes (Fig. 4e,f)^35,88^. Similarly, the elevated leaf C:N in AM-tree communities also explained the higher abundance and diversity of nematode herbivores. Root Ca concentration, as an indicator of root nutritional quality^89^, was elevated in EcM plots (Fig. 3 and Supplementary Fig. 6) and positively associated with the abundance of meso- and macrofauna (for example, fungivores and predators). This likely underlies the higher predator abundance in EcM plots (Fig. 1) and also aligns with evidence linking Ca-rich litter to increased soil-fauna abundance and activity^33,89^. The SEMs showed that soil NO_3_^−^, which decreased with tree diversity due to efficient plant uptake^87^, was not significantly associated with multitrophic abundance or diversity (Fig. 3), as responses varied among trophic groups (Fig. 4). Soil NO_3_^−^ was negatively correlated with nematode-fungivore abundance but positively correlated with bacterivore abundance and richness (Fig. 3e,f). This pattern is consistent with the bacterial energy channel being favoured by mineralization via nitrifying bacteria over the fungal channel^90^.

Soil C was not included in the SEMs (Methods), yet it was significantly associated with increased nematode and mesofauna abundance, particularly omnivores (Fig. 4e). As an indicator of habitat structure and organic matter availability^15^, soil C together with root C may explain the higher abundance of these groups in AM plots (Fig. 1). The SEMs captured mediating effects on invertebrate communities (Fig. 3) but did not include microbes. Tree diversity increased canopy complexity (ENL), reflecting greater aboveground biomass, litter input and microclimatic buffering (Table 10)^19,91^ and was positively associated with microbial biomass and diversity (Fig. 4e,f). In addition, leaf C:N, which increased with tree diversity and was higher in AM plots, was positively correlated with soil microbial diversity (Fig. 4f and Supplementary Fig. 5)^74,75^. Together, these results suggest that the effects of tree diversity and mycorrhizal type on microbial communities are mediated by canopy complexity and leaf C:N. Nevertheless, although these SEM pathways were grounded in established theory and supported by statistical associations, they remain inferential rather than directly causal and should be tested explicitly in future experiments. Moreover, we were not able to account for fine-root growth and turnover, which are key resource inputs to soil food webs and differ between AM and EcM communities^92,93^. Incorporating these processes in future studies would help clarify mechanisms underlying AM–EcM differences in soil food-web structure^53^.

Taken together, this comprehensive assessment of above- and belowground microbial and invertebrate communities in a temperate-forest experiment indicates that tree diversity and mycorrhizal type effects were most pronounced in soil communities. However, trophic-group responses to tree diversity were independent of mycorrhizal type. Moreover, by identifying key mediators of tree diversity and mycorrhizal-type effects on the abundance and diversity across trophic levels, we show that tree diversity and mycorrhizal-type effects were mediated by leaf C:N, canopy complexity and nutrient availability. By using a controlled field experiment, we highlight tree diversity and mycorrhizal types as distinct drivers of multitrophic biodiversity, primarily governing belowground bottom-up effects.

### Limitations and implications

Tree diversity effects are known to vary with climate and soil fertility, with stronger effects often observed under harsher conditions, such as drier climates or low-fertility soils^44,94^. This study was conducted in relatively young tree stands (~6 years), where the effects of tree diversity and mycorrhizal type may still be developing. Therefore, replicating comparable experiments across regions and long-term monitoring would help test the generality and temporal development of the observed effects. Observational studies across mature forests can further complement experimental approaches by capturing longer-term and broader environmental contexts, and experiments remain essential for establishing causality^46^. The lack of understory vegetation is a strength for isolating tree-driven effects. However, it may limit comparability with observational studies in forests, where understory plants influence resource heterogeneity, microhabitats and mycorrhizal associations. Moreover, although our findings may be applicable to broadleaf forests, many EcM-tree species in European forests are conifers; therefore, future experiments that include coniferous EcM species as an additional treatment would broaden the applicability of these findings. Still, this study integrates soil biodiversity with aboveground and soil-surface communities and contributes to our mechanistic understanding of above- and belowground community responses to tree-diversity loss and shifts in mycorrhizal dominance. Our findings might inform the design of diverse broadleaf forestry systems to sustain ecosystem functioning. We also highlight the need to consider belowground biodiversity in forest management decisions, as it represents ~59% of global biodiversity^12^ yet is currently largely overlooked in monitoring and conservation schemes^95^.

## Methods

### Study site and experimental design

This study was carried out at the tree diversity–ecosystem functioning experiment MyDiv^52^, which is located at the Bad Lauchstädt Experimental Research Station of the Helmholtz Centre for Environmental Research-UFZ, southwest of Halle, Saxony-Anhalt, Germany (51°23′N, 11°53′E; elevation: 114–116 m a.s.l.). The site has a temperate oceanic climate, with an average annual temperature of 8.8 °C and mean annual precipitation of 484 mm (ref. ^96^). The soil at the MyDiv site is highly fertile, classified as Haplic Chernozem, with a pH ranging from 6.6 to 7.4, and features a thick humus layer. The land had historically been used for agriculture until 2012 and was then transformed to a hay meadow.

The MyDiv experiment was established in March 2015^52^ and manipulates both tree species richness and mycorrhizal types. The experiment includes only deciduous tree species, despite many EcM-dominated forests in Europe containing multiple conifer species, to avoid introducing additional variation in leaf quality, soil acidification and fungal communities associated with conifer species. More specifically, MyDiv comprises ten deciduous angiosperm tree species commonly found in Germany, which can be categorized by their predominant mycorrhizal association. AM-tree species, referring to tree species associating predominantly with AM fungi, include *Acer pseudoplatanus* L., *Aesculus hippocastanum* L., *Fraxinus excelsior* L., *Prunus avium* L. (L.) and *Sorbus aucuparia* L.; EcM-tree species, referring to tree species associating predominantly with EcM fungi, include *Betula pendula* Roth, *Carpinus betulus* L., *Fagus sylvatica* L., *Quercus petraea* (Matt.) Liebl. and *Tilia platyphyllos* Scop. The design consists of a tree-diversity gradient (one, two and four species) and three mycorrhizal-type treatments (only AM-tree species (AM plots), only EcM-tree species (EcM plots) or both mixed (AM+EcM plots)). In total, there are eight treatment combinations: 3 × AM plots (one, two, four species), 3 × EcM plots (one, two, four species) and 2 × AM+EcM plots (two and four species), each replicated ten times, resulting in 80 plots (11 m × 11 m) located across two blocks. The restricted richness range in our study reflects the limited local tree-species pool and mirrors typical European forest stands, where more than 80% contain fewer than four tree species^97^. All data collection focused on the core 8 m × 8 m area of each plot, minimizing potential edge effects. Plots are separated by 3-m-wide grass paths, and water-permeable weed tarps were installed between trees to suppress weed growth (see Ferlian et al.^52^ for details). Understory plants were rare before and during the study period.

### Sampling organisms across above- and belowground

We assessed canopy fauna, soil-surface fauna, soil fauna (that is, nematodes, mesofauna and macrofauna, including earthworms) and soil microbial biomass and composition twice in 2021, once at the beginning of the vegetation period (May) and again at the end (September) of the growing season. We assessed foliar fungi and soil microbial diversity once between August and October 2021.

### Aboveground organisms

#### Canopy fauna

We used a technique similar to branch beating to collect invertebrates from tree canopies^10,98^. We first targeted four adjacent trees and branches from other trees projecting over the sample area (1 m × 1 m sampling square). A rubber-headed mallet was used to strike the branches to dislodge invertebrates, followed by shaking until no more material fell into the sampling tray (conical; 70-cm diameter, 30-cm depth). The collected material was transferred into 70% ethanol in a 200-ml plastic flask. In each plot, this procedure was repeated four times within the southeastern quadrant of the core area. With a stereoscopic microscope (Leica APO S8), the invertebrate specimens were identified to the family (for most specimens) or genus level (for Coleoptera and Formicidae), or if not feasible, at least to order level (for immature insects)^99–102^.

#### Foliar fungi

To assess foliar fungal communities, we sampled eight tree species (out of ten), excluding *Aesculus hippocastanum* (AM-tree species) and *Tilia platyphyllos* (EcM-tree species), from 42 plots spanning all tree-species richness levels and species compositions between 17 August and 10 September 2021^103^. Leaf samples were collected from the interaction zones of four neighbouring trees, including all species in the plot. Per plot, two interaction zones were sampled, amounting to a total of eight trees per plot. Ten leaves were collected from five random heights equally distributed across each tree. Afterwards, half of the sample was sterilized to assess endophytes using the protocol of Guerreiro et al.^104^, and the other half was used to assess the full phyllosphere (epi- and endophytes). Leaves were milled, and DNA was extracted using the ChargeSwitch gDNA Plant Kit (Thermo Fisher Scientific), with modified reagent volumes and lysis buffer. The fungal IT1 region was amplified using ITS1F (CTTGGTCATTTAGAGGAAGTAA)/ITS2 (GCTGCGTTCTTCATCGATGC) primers (Metabion, Planegg)^105^ with Illumina adaptors, sequenced (2 × 300 bp) on an Illumina MiSeq and processed in DADA2^106^. Samples below 5,000 raw reads were excluded from further analysis. Taxonomy was assigned with the naïve Bayesian classifier and the UNITE database (v8.3)^107,108^. Epiphytes were calculated by subtracting all endophytic taxa from the phyllosphere dataset; for details, see Köhler et al.^103^.

### Soil-surface fauna

Mobile invertebrates on the soil surface were collected in the northeastern quadrant of each plot using a combination of sieving and hand-sorting^10,109^. Initially, we gathered all litter and organic soil within an area of 50 × 50 cm on the weed tarp, sieved them (mesh size 2 cm) into a container and picked out the animals visible to the naked eye. Simultaneously, we conducted a 10-min survey of the bare plot area (the same 50 × 50 cm) under the weed tarp to capture emerging arthropods using forceps. All collected invertebrates were preserved in 70% ethanol and identified to family or genus level, at least to the order level, as was done for aboveground invertebrates.

### Belowground organisms

#### Soil fauna

We randomly collected five soil cores from the southwestern quadrant of the core area in each plot (5 cm in diameter; 0–10 cm depth)^10^. The five subsamples were pooled into one composite sample and sieved through a 2-mm mesh, resulting in a total of 160 samples (2 seasons × 80 plots). We used a modified Baermann method^110,111^ to extract free-living nematodes from ~25 g of soil. The extracted nematodes were preserved in formaldehyde solution (4%). The individuals of each sample were counted before identification. We identified all well-preserved specimens (if fewer than 100), or at least 100 specimens (if more than 100) were randomly selected and identified to genus if possible or at least to family level at ×400–×1,000 magnification using a Leica DMI 4000B light microscope^112^.

For the assessment of soil mesofauna and macrofauna, we took further soil cores (diameter: 16 cm, depth: 10 cm) from the northwestern quadrant in the core area of each plot. We extracted soil invertebrates from each core using the heat-extraction method^113^, gradually increasing the temperature from 25 °C to 55 °C over ten days. The extracted invertebrates were preserved in ethanol (70%), counted and identified under the stereoscopic microscope. Soil mites were identified using taxonomic keys^101,114,115^, classifying mites at the suborder level (Astigmata and Prostigmata), the superfamily level (Gamasina or Uropodina for Mesostigmata) and, if possible, at the family level for Oribatida. Collembola were classified at the family level^116–118^. The extracted soil macrofauna were identified to family level if possible or at least to order level (particularly for immature individuals)^101^. For the groups Protura, Pauropoda and Symphyla, only abundances were assessed.

Earthworm communities were sampled within a 50 × 50 cm area in the southwestern quadrant of each plot using a combination of digging and hand-sorting the upper 10 cm of soil, followed by extraction with mustard solution^109,119^. After clearing the upper soil layers to a depth of 10 cm, we applied 5 L of mustard solution (100 g per 10 L) to the hole and observed the area for 15 min, manually collecting any emerging earthworms. The procedure was then repeated with the second half of the solution for another 15 min. Additionally, the excavated soil was carefully examined by hand for the presence of earthworms. Retrieved earthworms were stored in 70% ethanol and taken to the lab. If possible, they were identified to species under a stereoscopic microscope^120^. Otherwise, they were assigned to ecological groups (anecic, endogeic or epigeic).

#### Soil microorganisms

Aliquots of the soil samples for nematode extraction were further used to assess total microbial, fungal, and bacterial biomass. Soil microbial biomass (Cmic; μg C per g dry soil) was measured using an O_2_-micro-compensation apparatus, based on the maximum initial respiratory response (μl O_2_ per h per g dry soil) to D-glucose (8 mg per g dry soil) addition within the first 10 h after substrate addition^121^. Soil microbial biomass was calculated as 38 × maximum initial respiratory response^122^. The phospholipid fatty acid (PLFA; ng per g dry soil) contents of soil samples were used to estimate the biomass ratio between fungi and bacteria (*F*:*B*) (refs. ^123–125^). More specifically, the ratio was derived from the sum of the PLFA markers 18:2ω6,9 and 16:1ω5 (neutral lipid fatty acid) for fungi and the sum of the PLFAs i15:0, a15:0, i16:0, i17:0, cy17:0 and cy19:0 for bacteria^125–127^. Moreover, the fungal biomass (*M*_F_) was estimated with the equation ${M}_{\rm{F}}=\frac{{C}_{\rm{mic}}\times F:B}{(F:B+1)}$ and bacterial biomass (*M*_B_) with the equation (${M}_{{\rm{B}}}=\frac{{C}_{\mathrm{mic}}}{(F:B+1)}$)^10^.

The fungal and bacterial communities in bulk soil were assessed by using paired-end amplicon sequencing^49,128^. We collected four soil cores (2 cm in diameter; 0–10 cm depth) from the centre of four neighbouring trees in September 2021^49^. The four cores were pooled, sieved through a 2-mm mesh and transported in a cooled box to the laboratory, where they were stored at −20 °C. Genomic DNA was extracted using the DNeasy PowerLyzer PowerSoil Isolation Kit (Qiagen) and quantified with a NanoDrop ND-1000, adjusted to 10–15 ng μl^−1^. The bacterial 16S rRNA gene’s V4 region was amplified using the primer pairs 515f (GTGCCAGCMGCCGCGGTAA) and 806r (GGACTACHVGGGTWTCTAAT)^129^ with Illumina adaptor overhangs. The fungal ITS2 rDNA region was amplified through a seminested polymerase chain reaction using the primer set ITS1F (CTTGGTCATTTAGAGGAAGTAA) and ITS4 (TCCTCCGCTTATTGAATGC)^130^, followed by amplification with the fITS7 (GTGARTCATCGAATCTTTG) and ITS4 (TCCTCCGCTTATTGAATGC) primer pair with Illumina adaptor sequence^131^. Amplified polymerase chain reaction products were purified (AMPure XP beads), indexed (Nextera XT), quantified (Qubit), pooled equimolarly and sequenced (2 bp × 300 bp; Illumina MiSeq). The raw reads were processed using QIIME 2 (v2022.2)^129^ as described in Ul Haq et al.^128^. Briefly, primers were trimmed from the Illumina MiSeq paired-end raw reads, where the forward and reverse reads were demultiplexed based on their respective index combinations through the Illumina standard procedure, using the cutadapt function (q2-cutadapt plugin). Subsequently, the trimmed sequences were denoised, merged, cleaned from chimeric reads and grouped into amplicon sequence variants (ASVs) using the DADA2 denoise-paired algorithm (via q2-dada2 plugin). Taxonomic assignment was done using the SILVA 132-99-515-806 naïve Bayesian classifier reference database^132^ for bacteria and the UNITE (v9) dynamic classifier reference database for fungi^108^. The respective fungal and bacterial ASV matrices, taxonomic tables and representative sequences were filtered to remove non-target taxa. Those ASVs assigned at least at the phylum level were retained. The per-sample read coverage was normalized to the minimum number of reads in both datasets.

### Assessing potential mediators of tree-community effects

We hypothesized that tree diversity and mycorrhizal type influence above- and belowground communities indirectly through changes in aboveground tree-stand characteristics (for example, canopy structure complexity^19,133^, productivity^5,62^ and leaf quality^87^). Moreover, soil properties^87,134^ and root nutrient contents^135–137^ may mediate the effects of tree diversity and mycorrhizal type on both soil-surface and belowground communities. Details of the hypothesized framework and the supporting literature are shown in Supplementary Fig. 1, and all mediating parameters measured in this study are described below.

### Aboveground tree-stand characteristics

We quantified treewood volume as a proxy of aboveground tree biomass^138,139^. We measured the stem diameter at 5 cm above the ground (*d*_0_) and the height (*H*) for all living trees in the core area in December 2020^62^. Then, we estimated the wood volume for every living tree in the core area by using the equation ${\rm\pi }\times {(\frac{{d}_{0}}{2})}^{2}\times H\times 0.5$ (refs. ^19,140,141^). The volumes of all living individual trees were summed to determine the totalwood volume per plot^72^.

We quantified aboveground canopy structural complexity using the ENL determined by terrestrial laser scanning in the core area of each plot in September 2021^19,142^. ENL provides a holistic and reproducible description of the three-dimensional vertical stand structure, independent of human estimation and classic dendrometric parameters^72,143,144^.

To measure leaf C and N concentrations as a proxy of leaf quality, we sampled nine healthy leaves at 2.5 m height from three individuals for each species per plot in July 2021^145^. The nine leaves for each species per plot were pooled into one sample and were dried at 60°C and ground with a ball mill (MM 400; Retsch). Five milligrams from each sample were used to determine leaf total C and total N concentrations with a CHNS elemental analyser (vario EL cube; Elementar Analysensysteme GmbH). The average leaf C, N, and C:N ratio of all species was used to define the leaf quality in every plot^72,146^.

### Root-nutrient parameters

We collected eight soil cores (5 cm diameter × 10 cm depth; two from each plot side in the second tree row) from each of the 80 experimental plots in October 2021 to quantify fine-root C and nutrient concentrations. The cores from each plot were pooled into a composite sample and stored at 3°C until further processing. Fine roots were extracted from each composite sample using a stacked sieving system (1 mm, 0.5 mm and 0.1 mm; from top to bottom). Soil was placed on the top sieve and gently washed with low-pressure water to disaggregate the soil matrix and recover root material. Recovered roots were manually cleaned of remaining soil particles and organic debris under running water. Fine roots were sorted into absorptive roots (first and second order) and transport roots (third order and higher)^147^ and oven-dried at 70°C for 72 h. Dried root material was ground using a ball mill (MM 200, Retsch), ~2 mg of the ground roots were placed in a tin capsule, and C and N concentrations were measured using a CHN elemental analyser (EURO-EA 3000, Euro Vector). For further elemental analysis, ~100 mg of the ground roots were digested via microwave-assisted acid digestion (START microCHEMIST 1500, MLS GmbH) using 2 ml HNO_3_ (2 mM) and 1 ml H_2_O_2_ (30%). The digested solutions were filled up to 15 ml with ultrapure water and then analysed via inductively coupled plasma optical emission spectroscopy (iCAP 6300 Duo, Thermo Scientific) to quantify elemental concentrations (for example, Ca and magnesium (Mg))^148^.

### Soil properties

The subsamples for soil fungal and bacterial diversity were used to measure soil pH and soil total C and N. Ten grams of ground soil per sample were used for pH measurements by adding 0.01 mol CaCl_2_. Soil total N (TN) was determined on an auto-analyser (SEAL Analytical GmbH) using the Kjeldahl method. Soil total C (TC) was measured using a TOC analyser (Liqui TOC II, Elementar Analysis System GmbH). Soil C:N was calculated using the soil TC and TN values. Soil ammonium (NH_4_^+^), nitrate (NO_3_^−^) and phosphate (PO_4_^3−^) were detected from ion-exchange membranes^87,149–151^.

### Trophic groupings

Organisms were assigned to trophic groups based on feeding preferences^152^. Canopy fauna were analysed as a whole group and further classified as herbivores, fungivores, omnivores and predators (including parasites and parasitoids). Foliar fungi were grouped as total, endophytic and epiphytic communities. Soil-surface fauna was analysed as a whole group and similarly grouped as herbivores, detritivores, omnivores and predators. Belowground organisms included both invertebrates and microorganisms. Invertebrates were first divided by body size and sampling method into nematodes, mesofauna and macrofauna. Nematodes were analysed as a whole group and then further divided into herbivores, bacterivores and fungivores. Predators and omnivores were grouped into the same group. Mesofauna was first analysed as a whole group and then categorized into detritivores, fungivores, omnivores and predators. Macrofauna (excluding earthworms) was classified into herbivores, omnivores and predators. Earthworms were analysed as a separate group. Feeding niches often differ between life stages, particularly in insects. Therefore, we assigned trophic groups separately for juvenile and adult stages within the same taxonomic group^10^. For example, Diptera exhibit ontogenetic shifts in feeding preferences and habitat-use between larval and adult stages. In contrast, many Hymenoptera are parasitoids throughout their life cycle; although the parasitic stages were not sampled and adults rarely feed, we classified them as parasitoids (or predators), following previous studies, to reflect their ecological role^11,18^. Soil microbial communities were grouped as total microbes, fungi and bacteria. Trophic-group definitions are detailed in the Supplementary Information and Supplementary Data 1.

### Statistical analyses

#### Calculation and transformation of biodiversity metrics

All statistical analyses were conducted by using R (version 4.5.3; http://www.Rproject.org). We calculated abundance and taxonomic richness for each invertebrate trophic group within each stratum and for entire canopy and soil-surface communities, as well as for total nematodes, mesofauna, and macrofauna belowground. Specimens within each trophic group were collected using the same method and from the same area. Abundance was calculated as the total number of individuals per group. Richness metrics varied by taxonomic resolution, with earthworms assessed at the species level, nematodes at the genus level and other groups primarily at the family level. For canopy and soil-surface fauna, Shannon–Wiener diversity indices were calculated for each trophic group and for the whole community (R package ‘vegan’^153^). For belowground fauna, due to low abundance in some trophic groups, Shannon–Wiener indices were calculated only at the community level (that is, total nematodes, mesofauna and macrofauna), not per trophic group. Due to incomplete information for the belowground compartment, the statistical results for the Shannon–Wiener indices are shown only in the Supplementary Information. All invertebrate trophic groups were sampled in all 80 plots (ten replicates per treatment) during two sampling campaigns.

The foliar fungal community from two interaction zones with four individual trees each was aggregated to a single sample per plot, after which diversity indices were calculated. DNA-sequence reads were used as proxies for the abundance of total foliar, endophytic and epiphytic fungi^154^. For foliar fungi, taxonomic richness was estimated using the Chao1 richness estimator (Chao1)^155^. The Abundance-based Coverage Estimator^156^ was also used for comparison; the values were similar to those obtained with Chao1, and therefore only Chao1 is presented in the results. The Shannon–Wiener diversity index was calculated. In total, we calculated biodiversity metrics for endophytes in 39 plots (out of 42) and for total foliar and epiphytic fungi in 34 plots^103^. For soil microbial communities, total microbial biomass was used as a proxy for overall microbial abundance, with fungal and bacterial biomass serving as proxies for fungal and bacterial abundances, respectively. The taxonomic richness (Chao1) and Shannon–Wiener diversity indices were calculated for soil fungi and bacteria. Soil microbial biomass was measured in both seasons (80 plots × 2 seasons), whereas fungal and bacterial diversity indices were determined once per plot (80 plots) at the end of the growing season.

To facilitate comparison of the effects of tree diversity and mycorrhizal types across strata and trophic groups, we standardized diversity indices (*y*_*i*_) to a range of [0,1]^18,81^ using the following transformation:

$${y}_{i}=\frac{{y_i^0}-{y}_{\min }}{{y}_{\max }-{y}_{\min }}$$

where ${y_i^0}$ represents the original (untransformed) diversity index for each plot *i* in each month, and ${y}_{\min }$ and ${y}_{\max }$ are the minimum and maximum diversity values, respectively, observed across all plots and sampling seasons. This transformation scales the diversity values within each group to a [0,1] range for consistent comparison across different strata and trophic groups^18,81^. The summary for the minimum and maximum values for each trophic group at each stratum is shown in Supplementary Tables 1–3. To avoid any potential confounding influence of extreme values on subsequent statistical analyses and transformations, we identified and removed outliers for each biodiversity metric before transformation. Outliers were detected using the interquartile range (IQR) method to account for the potential for extreme values arising from organisms with small body sizes, such as aphids in the tree canopy. Specifically, we assessed whether values below the 5th percentile fell below the lower fence (Q1 – 1.5 × IQR) or whether values above the 95th percentile exceeded the upper fence (Q3 + 1.5 × IQR) (ref. ^157^). All removed values are listed at the bottom of the corresponding summary tables (Supplementary Tables 1–3). The analysis results were consistent before and after outlier removal; however, removing outliers substantially improved model performance, with residuals more closely following a normal distribution. Therefore, we report only the results obtained after outlier removal.

Due to the use of different fungal and bacterial sequencing primers, taxonomic richness and Shannon–Wiener diversity for whole soil microbial communities could not be calculated directly. Therefore, we averaged the min–max transformed taxonomic richness and Shannon–Wiener diversity values for fungi and bacteria to represent the overall taxonomic richness and Shannon–Wiener diversity of the total soil microbial community^158^. A similar approach was applied to estimate total soil macrofauna: we averaged the min–max transformed abundance, taxonomic richness and Shannon–Wiener diversity of macrofauna (from the heat extraction) and earthworms.

As a synthesis, we calculated multitrophic abundance and diversity for each stratum by averaging min–max transformed abundance and taxonomic richness of each trophic group within the stratum^11,158,159^. For the aboveground communities, we averaged the transformed abundance of herbivores, fungivores, omnivores and predators to obtain multitrophic abundance while excluding foliar fungi due to incomplete coverage (37 out of 80 plots). The multitrophic abundance at the soil surface was calculated by averaging the transformed abundance of herbivores, detritivores, omnivores and predators. For consistency, belowground multitrophic abundance was calculated from the transformed abundance of nematode herbivores, fungivores, bacterivores, and predators (including omnivores); mesofauna fungivores, omnivores and predators; and macrofauna herbivores, predators, omnivores and earthworms, but excluding soil microbes. The multitrophic abundance was calculated as the arithmetic mean across trophic groups with equal weighting. The missing trophic-group values were omitted on a per-sample basis such that each sample’s metric reflects the mean of the groups available. Multitrophic diversity was calculated similarly by averaging the min–max transformed taxonomic richness of trophic groups within each stratum. Additionally, we conducted a sensitivity analysis using *Z*-score transformations^10,158,159^.

#### Effects of tree species richness and mycorrhizal types

We used mixed-effects models to analyse the effects of tree diversity and mycorrhizal types on biodiversity indices. For each index within each group of organisms in each stratum, we started from the model lme (*y*_*i*_ ≈ month + Mycor × log(Richness), ~1| composition/month, data) (R package ‘nlme’^160^). Type I Sums of Squares were used to account for the variance explained by sampling season before testing the effects of two treatment factors. To meet the assumptions of normality and homoscedasticity, we applied power transformations ${y}_{i}{^t}$. Parameters *t*∈[0,3] were selected using bootstrapping to optimize residual normality in the mixed-effects models. Although the procedure identified a range of suitable *t-*values, subsequent models were fitted using the single value that yielded the best residual normality based on the Shapiro–Wilk test (Supplementary Tables 1–3). We used power transformation rather than the log transformation log(${y}_{i}$ + a) because the latter would overly suppress smaller values in ${y}_{i}$ scaled to the range [0,1] beforehand^161^ and also was not applicable for taxonomic richness and Shannon–Wiener diversity that followed either normal or left-skewed distribution. The power transformation ${y}_{i}{^t}$ could effectively handle right-skewed abundance data (with *t* < 1) and left-skewed Shannon–Wiener indices (with *t* > 1) (ref. ^162^). Therefore, the same mixed-effects modelling framework allowed us to accommodate a broad range of linear and nonlinear responses across trophic levels, food-web compartments and biodiversity indices. In practice, this also streamlined the workflow and improved the reproducibility of our analyses. We generated *F*-test analysis of variance tables for the models using the optimal *t*-value identified via bootstrapping (Supplementary Tables 4–8).

However, interactions effects between the treatment factors were non-significant for all three biodiversity indices in most trophic groups (26 out of 29) across above- and belowground compartments, with the exception of three groups: nematode herbivores, soil-macrofauna predators and total soil macrofauna. Consequently, we simplified the models as lme ((*y*_*i*_)^t^ ≈ month + Mycor + log(Richness), ~1| composition/month, data) after transforming (*y*_*i*_) with the optimal *t*-value for all fauna and soil microbial biomass. Additionally, for these three trophic groups that showed significant responses to treatment interactions, we visualized the results in the Supplementary Information based on the full model outputs. For foliar fungi, alpha diversity of soil fungi and bacteria sampled once in September 2021, we used the models lme ((*y*_*i*_)^t^ ≈ Mycor + log(Richness), ~ 1| composition, data) after transforming (*y*_*i*_) with the optimal *t*-values. Due to zero inflation, we excluded nematode predators/omnivores, soil-macrofauna herbivores and fungivores from the analyses.

The same analyses were also performed on the original data (before min–max transformation). The results are presented in the Supplementary Information (see Supplementary Figs. 2–5). The statistical significance of treatment effects, as well as the overall trends observed in the min–max transformed data, were consistent with those obtained from the original data. Importantly, the transformation facilitated comparisons of treatment effects in the figures, including the magnitude of increases along the tree-diversity gradient and differences between mycorrhizal types^18^.

Because group-specific power transformations were applied, the coefficients of log(Richness) could not be directly compared to determine which groups exhibited stronger tree-diversity effects across the three strata. To facilitate statistical comparisons of tree-diversity effects across trophic groups and food-web compartments, we extracted model predictions (mean ± 95% confidence interval) at three levels of species richness (one, two, four) using the emmeans package. The log-response ratios of predicted values for two versus one and four versus one tree species were calculated as standardized effect sizes to quantify the magnitude of tree-diversity effects across trophic groups and food-web compartments. In addition, the log-response ratios were calculated to assess the magnitude and robustness of tree-diversity effects beyond significance levels. The log-response ratios were calculated using both the min–max transformed data and the original values. For most trophic groups, the results were consistent between the two approaches, confirming the robustness of our findings (Supplementary Figs. 11 and 13). The only exception was soil fungal and bacterial taxonomic diversity, where the log-response ratios based on the original data were slightly lower than those derived from the transformed values.

We calculated mean effect sizes (log-response ratios) of trophic groups within each compartment for abundance and taxonomic richness using the min–max transformed data. Differences were tested with the model (mean(log-response ratio) ≈ Stratum × Richness contrast), where the stratum included aboveground, soil-surface and belowground compartments, and the richness contrast included the two- and four-species treatments. These results summarize the response magnitude across food-web compartments but may be biased, because more trophic groups were included in the belowground compartment and variation among trophic groups was not accounted for.

#### Relationships between mediators and biodiversity matrices

To investigate how tree-stand characteristics, root nutrients and soil properties mediate the experimental treatment effects on the biodiversity of invertebrates and microbes, we used SEMs (piecewiseSEM^163^) and multiple linear regression models (‘nlme’ package^160^) combined with backwards selection based on the Akaike Information Criterion with correction for small sample sizes (AICc) (‘MuMIn’ package^164^).

Before analysis, we first selected the potential mediators that were significantly affected by tree species richness and mycorrhizal types from all of the parameters we measured (Supplementary Fig. 6 and Supplementary Table 9). All aboveground tree-stand characteristics, including total-wood volume, ENL, leaf C, N and C:N, were affected by tree species richness and/or mycorrhizal types. Amongst the root C and nutrient contents (C, N, Ca and Mg contents of absorptive and transport roots and their C:N, respectively), C, Mg and Ca contents of absorptive roots were significantly affected by either tree species richness or mycorrhizal types, whereas Ca contents in transport roots showed significant differences amongst mycorrhizal types (Supplementary Fig. 6 and Supplementary Table 9). For soil properties (pH, C, N, C:N, NO_3_^−^, NH_4_^−^, PO_4_^−^), only TC, TN and NO_3_^−^ were influenced by mycorrhizal types and/or tree species richness (Supplementary Fig. 6 and Supplementary Table 9). We assessed collinearity among nine potential mediators influenced by the experimental treatments to simplify the model and reduce the multicollinearity (Supplementary Table 10; Pearson’s *r* < 0.40) (ref. ^165^). Wood volume and ENL were strongly positively correlated (*r* = 0.73); because ENL captures both aboveground productivity and canopy complexity, we retained ENL and excluded wood volume (Supplementary Table 10)^19^. Leaf C was strongly positively correlated with leaf N (*r* = 0.52), and leaf N was significantly negatively correlated with C:N (*r* = −0.97); we retained C:N as a single indicator of leaf-nutrient quality (Supplementary Table 10)^87^. Root Mg and Ca were strongly correlated (*r* = −0.62); we used root Ca to represent root-nutrient content^89^, alongside root C. Soil TC and TN were positively correlated (*r* = 0.74); we retained TC, whereas soil N status was represented by soil NO_3_^−^, which is a powerful predictor of soil-nutrient availability (Supplementary Table 10)^87^.

We used piecewise SEMs to investigate whether these parameters (ENL; leaf C:N; root C and Ca; soil C and NO_3_-) mediate the effects of tree species richness and mycorrhizal types on multitrophic abundance and diversity of aboveground, soil-surface and belowground organisms. This analysis was limited to the values in September to align with the sampling period of the mediator variables. For aboveground organisms, we assumed that treatment effects could be mediated via aboveground tree-stand characteristics (ENL and leaf C:N), whereas for soil-surface and belowground organisms, we also included root and soil parameters as potential mediators (Supplementary Fig. 7). Mycorrhizal types were recorded as the proportion of EcM-tree species (EcM%; 1 = EcM plots, 0.5 = AM+EcM plots, 0 = AM plots) to meet the requirements of piecewiseSEM. However, this linear coding could not capture non-additive patterns (for example, soil C in AM+EcM plots being significantly lower than in AM plots and Supplementary Fig. 6), which is why we excluded soil C from the SEMs. We also included a richness × mycorrhizal interaction for root C (Supplementary Fig. 7). Model fit was assessed using Fisher’s *C* statistic (*P* > 0.05 indicating adequate fit^163^). Non-significant paths were removed, and direct paths from exogenous variables (tree species richness and mycorrhizal type) to endogenous variables (multitrophic abundance and diversity) were retained only if they remained (marginally) significant (*P* < 0.1) after accounting for mediators. The SEM framework was applied to multitrophic abundance and diversity by using min–max transformation in the main analysis. It was also used in sensitivity analyses with *Z*-score transformation. To test whether aboveground productivity alone influenced the abundance or diversity of consumers, particularly multitrophic abundance, we conducted a sensitivity analysis by replacing ENL with total-wood volume and by adding total-wood volume to the SEMs (Supplementary Tables 13 and 14). In both cases, wood volume had no significant or marginal effects on any endogenous variable (Supplementary Tables 13 and 14), further supporting the structural robustness of our final model.

The above analysis of multitrophic abundance and diversity revealed general trends across ecosystem compartments. However, when individual trophic groups respond differently, the effects of mediating parameters may be concealed. Therefore, we examined how these parameters mediate the effects of tree species richness and mycorrhizal types on each trophic group individually. This also allowed us to investigate mediating effects on foliar fungi and soil microorganisms. To align with the mediator sampling period, we still used only the abundance, taxonomic richness, and Shannon–Wiener diversity of each above- and belowground trophic group sampled in September. The biodiversity matrices were min–max scaled. The scaled biodiversity matrices were further power-transformed by using bootstrapping to select *t*-values from *t*∈[0,3] to meet model assumptions of normality and homoscedasticity. For each trophic group, we fitted a full linear mixed-effects model by adding six mediators as fixed factors and tree composition as the random factor. We then applied backwards selection (‘dredge’ function) to identify the best-fitting model with the lowest AICc. Finally, we standardized the coefficients of the selected models for comparability across predictors (‘parameters’ package^166^). In these group-specific analyses, we were able to further include the mediator of soil C that we had to leave out of the SEMs.

### Reporting summary

Further information on research design is available in the Nature Portfolio Reporting Summary linked to this article.

## Supplementary information

Supplementary Information Supplementary Methods, Figs. 1–15 and Tables 1–17.

Reporting Summary

Peer Review File

Supplementary Data 1 This file contains all taxonomic names identified in this study, as well as information on trophic-group classifications.

## Data Availability

The datasets used in this study are publicly available in the MyDiv database (https://mydivdata.idiv.de). Dataset ID 220 (ref. ^152^) contains plot-level biodiversity metrics across food-web compartments. Dataset ID 276 (ref. ^167^) contains taxonomic information and trophic-group classifications, referred to as Supplementary Data 1 in the main text. All mediator data used in this study are also deposited in the MyDiv database. Leaf nutrient data^146^, canopy complexity data^142^ and soil parameters^150,151^ are publicly available. Root nutrient data (ID 213) are available upon request because the data owners are preparing related publications.

## Code availability

All R scripts used for the analyses are publicly available in the MyDiv database under dataset ID 276 (ref. ^167^).
