# Supplementary material for: Tree diversity and mycorrhizal type independently shape multitrophic biodiversity, with stronger effects belowground than aboveground
Source: Nat Ecol Evol. 2026 Aug 31;10(9):1628–43. doi: 10.1038/s41559-026-03147-6 (PMC13541612; doi:10.1038/s41559-026-03147-6)
Supplement: Supplementary file 1 [file 41559_2026_3147_MOESM1_ESM.pdf]

nature ecology & evolution

Supplementary information

https://doi.org/10.1038/s41559-026-03147-6

Tree diversity and mycorrhizal type independently shape multitrophic biodiversity, with stronger effects belowground than aboveground

In the format provided by the authors and unedited

Table of Content

Supplementary Methods 3
Details for trophic groupings 3
Construction of Structural Equation Models 4

Supplementary Figures 6

Figure S1. Conceptual figure of the hypothesized structural equation model (SEM) framework for tree species richness, mycorrhizal type, and environmental mediator effects on multitrophic abundance and diversity across above- and belowground food webs 6
Figure S2. Effects of tree species richness (TSR), mycorrhizal type (Mycor), and their interaction on the abundance, taxonomic richness, and Shannon–Wiener diversity of trophic groups in the aboveground compartment 8
Figure S3. Effects of tree species richness (TSR), mycorrhizal type (Mycor), and their interaction on the abundance, taxonomic richness, and Shannon–Wiener diversity of trophic groups in the soil surface compartment 9
Figure S4. Effects of tree species richness (TSR), mycorrhizal type (Mycor), and their interaction on the abundance and taxonomic richness of trophic groups in the belowground compartment 10
Figure S5. Effects of tree species richness (TSR), mycorrhizal type (Mycor), and their interaction on the taxonomic richness and Shannon–Wiener diversity of fungal functional groups 12
Figure S6. Relationships between parameters for aboveground tree stands (A–C), root nutrients (D–F), soil properties (G–I), and tree species richness across three mycorrhizal type treatments 13
Figure S7. Simplified framework of the hypothesized structural equation model (SEM) for multitrophic abundance and diversity in above- and belowground food webs 14
Figure S8. Scatterplots showing correlations between abundance and taxonomic richness for each trophic group across (A) aboveground, (B) soil surface, and (C–E) belowground compartments 15
Figure S9. Effects of tree species richness and mycorrhizal type on organism Shannon–Wiener diversity aboveground (A–B), at the soil surface (C–D), and belowground (E-F) 16
Figure S10. Effects of mycorrhizal type, tree species richness, and their interaction on standardized abundance (A–B) and taxonomic richness (C–E) 17
Figure S11. Standardized tree diversity effect sizes on abundance (A, D, G), taxonomic richness (B, E, H), and Shannon–Wiener diversity (C, F) across trophic groups and food-web compartments (aboveground, soil surface, and belowground) 18
Figure S12. Averaged effect sizes of trophic groups across three food-web compartments 20
Figure S13. Standardized tree diversity effect sizes on abundance (A, D, G), taxonomic richness (B, E, H), and Shannon–Wiener diversity (C, F) across trophic groups and food-web compartments 21
Figure S14. Results of multiple linear regressions showing the effects of six mediators on standardized organism Shannon–Wiener diversity aboveground (A), at the soil surface (B), and belowground (C) 22
Figure S15. Sensitivity analysis showing how tree species richness and mycorrhizal type, expressed as the proportion of EcM tree species, affect multitrophic abundance (A) and diversity (B) of invertebrates 23

Supplementary Tables **24**

Table S1. Summary of response variables for aboveground specimens on the original scale 24

Table S2. Summary of response variables for soil surface specimens on the original scale 25

Table S3. Summary of response variables for belowground specimens on the original scale... 26

Table S4. Summary of mixed-effects models testing the effects of sampling season (Month), tree species richness (TSR; 1, 2, 4 species), mycorrhizal type (Mycor; AM, EcM, AM + EcM), and their interactions on diversity indices of aboveground invertebrates 27

Table S5. Summary of mixed-effects models testing the effects of tree species richness (TSR; 1, 2, 4 species), mycorrhizal type (Mycor; AM, EcM, AM + EcM), and their interaction on diversity indices of foliage fungi 28

Table S6. Summary of mixed-effects models testing the effects of sampling season (Month), tree species richness (TSR; 1, 2, 4 species), mycorrhizal type (Mycor; AM, EcM, AM + EcM), and their interaction on diversity indices of surface invertebrates 29

Table S7. Summary of mixed-effects models testing the effects of sampling season (Month), tree species richness (TSR; 1, 2, 4 species), mycorrhizal type (Mycor; AM, EcM, AM + EcM), and their interaction on diversity indices of belowground invertebrates 30

Table S8. Summary of mixed-effects models testing the effects of sampling season (Month), tree species richness (TSR; 1, 2, 4 species), mycorrhizal type (Mycor; AM, EcM, AM + EcM), and their interaction on diversity indices of soil microbes 32

Table S9. Summary of mixed-effects model analyses testing the effects of tree species richness (TSR; 1, 2, 4 species) and mycorrhizal type (Mycor; AM, EcM, AM + EcM) on potential mediators 33

Table S10. Pearson's correlations among mediators significantly influenced by treatment factors, including tree species richness and/or mycorrhizal type 34

Table S11A. Structural equation model for multitrophic abundance of invertebrates, with min–max transformation 35

Table S11B. Structural equation model for multitrophic diversity of invertebrates, with min–max transformation 37

Table S12A. Structural equation model for multitrophic abundance of invertebrates, with z-score transformation 39

Table S12B. Structural equation model for multitrophic diversity of invertebrates, with z-score transformation 41

Table S13A. Structural equation model for multitrophic abundance of invertebrates, with min–max transformation and ENL replaced by tree wood volume as a mediator 43

Table S13B. Structural equation model for multitrophic diversity of invertebrates, with min–max transformation and ENL replaced by tree wood volume as a mediator 45

Table S14A. Structural equation model for multitrophic abundance of invertebrates, with min–max transformation and tree wood volume included as a mediator 47

Table S14B. Structural equation model for multitrophic diversity of invertebrates, with min–max transformation and tree wood volume included as a mediator 49

Table S15. Model structure and performance for detecting mediator effects on biodiversity indices of aboveground organisms 51

Table S16. Model structure and performance for detecting mediator effects on biodiversity indices of soil surface organisms 53

Table S17. Model structure and performance for detecting mediator effects on biodiversity indices of belowground organisms 54

Supplementary Methods

Details for trophic groupings

Canopy fauna

Invertebrates were classified into trophic groups based on their ecological roles as predators, fungivores, herbivores, pollinators, and omnivores (details listed Supplementary Data 1 [1–14]). The following taxonomic groups were identified and classified:

Predators: Predatory arthropods included 17 families from the orders Araneae, two families of Opiliones, and three families of Coleoptera (Coccinellidae, Cantharidae, Carabidae). Additional predatory taxa included two families of Diptera (Hybotidae, Dolichopodidae), the genus *Formica* from the family Formicidae, one family from Hemiptera (Reduviidae), and families from Raphidioptera, Odonata, and Mesostigmata. Three families of Prostigmata (Anystidae, Bdellidae, Trombidiidae) were also categorised as predators. We also grouped twelve families of Hymenoptera as predators, including Vespidae, Crabronidae, while the other ten families were categorised as parasitoids.

Fungivores: Fungivore families comprised five families of Coleoptera, four families of Psocoptera, three families of Collembola, and two families of Thysanoptera.

Herbivores: Herbivores included eight families of Coleoptera, eight families of Hemiptera, two families of Diptera (Bibionidae and Drosophilidae), and families (e.g., Rhaphidophoridae and Tettigoniidae) from Orthoptera. Additional herbivores included larvae from Lepidoptera, Hymenoptera, Neuroptera, the genus *Lasius* from the family Formicidae, and two families of Gastropoda.

Omnivores: Omnivores included three families of Coleoptera (Anthicidae, Elateridae, Staphylinidae), and additional representatives from Dermaptera, Diptera, and mites from Oribatida and Prostigmata.

Foliar fungi

Total foliar fungi: From non-sterilised samples representing the entire phyllosphere fungal community [15].

Endophytic (Endo.) fungi: From surface-sterilised samples representing the endophytic community *sensu stricto*.

Epiphytic (Epi.) fungi: A derived subset was generated by subtracting taxa present in the endophytic subset from the non-sterilised phyllosphere data.

Soil surface fauna

Invertebrates at the soil surface were categorised into trophic groups based on their ecological roles as predators, detritivores, herbivores, and omnivores [1–3,10,12,13,16,17]. The following classifications were established:

Predators: This group comprised 14 families from Araneae and Opiliones, four families of Coleoptera, and three families from Chilopoda. Additional predatory taxa included two

families from Diplura, Mesostigmata mites, Pseudoscorpiones, and the family Neanuridae from Collembola.

Detritivores: Dominated by six families of Diplopoda, detritivores also included two families of Coleoptera (Anobiidae and Cholevidae), the family Campodeidae from Diplura, Enchytraeidae and Lumbricidae (Oligochaeta), and Isopoda.

Herbivores: The herbivores consisted of seven families of Coleoptera, seven families of Hemiptera, two families of Diptera (Bibionidae and Drosophilidae), larvae from Lepidoptera and Neuroptera, two families from Orthoptera, Tetranychidae mites, and various families from Gastropoda.

Omnivores: Omnivores included larvae of Coleoptera, two families of Coleoptera (Silphidae and Staphylinidae), larvae of Diptera, three genera of Formicidae, and Dermaptera (Forficulidae).

Soil fauna

Soil invertebrates were categorised by body size into nematodes, mesofauna, and macrofauna. Different extraction and identification methods were applied based on body size, and taxonomic and trophic groups were classified as follows to ensure comparability:

Nematodes: Nematodes were analysed as a whole group (Ne:Total) and further classified by feeding preference into herbivores (Ne:Herbivores), fungivores (Ne:Fungivores), and bacterivores (Ne:Bacterivores) [18–20]. Due to overall low densities and therefore issues with zero inflation, predator and omnivore nematodes were grouped into one group in diversity analyses [21,22].

Mesofauna: Mesofauna was analysed as a whole group, with some taxa further classified by function [10,17,23,24,24–29]. Fungivores (Me:Fungivores) included seven families of Collembola, three families of Oribatida mites, Protura, Psocoptera, and Thysanoptera. Predators (Me:Predators) included Mesostigmata, two families of Oribatida (Oppiidae and Hypochthonidae), the family Neanuridae from Collembola, Anajapygidae from Diplura, and Pseudoscorpiones. The remaining mesofauna were considered omnivores (Me:Omnivores).

Macrofauna: Macrofauna were grouped into herbivores (Ma:Herbivores), fungivores (Ma:Fungivores), predators (Ma:Predators), and omnivores (Ma:Omnivores), and earthworms [1,10,17,30–33].

Soil microbial communities

Total soil microbes: Total soil microbial abundance was represented by total microbial biomass, and alpha diversity was calculated as the average of both bacterial and fungal alpha diversity (details provided below).

Soil fungi: Fungal abundance was represented by fungal biomass, and alpha diversity indices were obtained from sequencing data.

Soil bacteria: Bacterial abundance was represented by bacterial biomass, and alpha diversity indices were obtained from sequencing data.

Construction of Structural Equation Models

For the final SEM framework in Fig. S7, we only included mediators that were significantly affected by the treatments (see Table S9, Fig. S6) and were not strongly correlated with each other (Table S10; details written in lines 823-843). The final set of mediators included ENL, leaf C:N, root Ca, root C, and soil NO_3^-. Soil C was excluded, because its response pattern indicated non-additive treatment effects (e.g., lower values in AM + EcM mixtures than in AM plots; Fig. S1), which could not be adequately represented by the linear coding used in the SEM.

Based on the hypothesized relationships (Fig. S1; Fig. S7), we first fitted a full piecewise structural equation model (SEM) including all plausible pathways among treatment variables, mediators, and multitrophic responses. All component models were fitted as linear mixed-effects models with composition included as a random effect, except for root Ca, for which the random-effect variance was negligible. EcM% represents the proportion of EcM tree species, and log(Richness) represents log-transformed tree species richness. The multitrophic diversity and abundance are shortened as multi-indices, or multi-abundance, or multi-diversity.

aboveground multi-index ~ Leaf C:N + ENL
surface multi-index ~ ENL + Leaf C:N + root Ca + root C + Soil NO3-
belowground multi-index ~ ENL + Leaf C:N + root Ca + root C + Soil NO3
ENL ~ log(Richness) + EcM%
Leaf C:N ~ log(Richness) + EcM%
root Ca ~ log(Richness) + EcM%
root C ~ log(Richness) + EcM% + log(Richness) × EcM%
NO_3^- ~ log(Richness) + EcM%

The initial SEM was simplified by sequentially removing unsupported paths while monitoring model performance, marginal R^2, and tests of directed separation. Removal of unsupported predictors had little effect on marginal R^2 or overall model fit. Additional direct pathways were included only when tests of directed separation indicated missing relationships ($p < 0.1$). The final SEMs therefore represent reduced versions of the hypothesized full model.

The final models for multitrophic abundance were:

aboveground multi-abundance ~ Leaf C:N + ENL
surface multi-abundance ~ ENL + Leaf C:N + root Ca
belowground multi-diversity ~ ENL + Leaf C:N + root Ca + log(Richness)

The final models for multitrophic diversity were:

aboveground multi-diversity ~ Leaf C:N + ENL + log(Richness)
surface multi-diversity ~ ENL + Leaf C:N + root Ca
belowground multi-diversity ~ ENL + Leaf C:N + root Ca

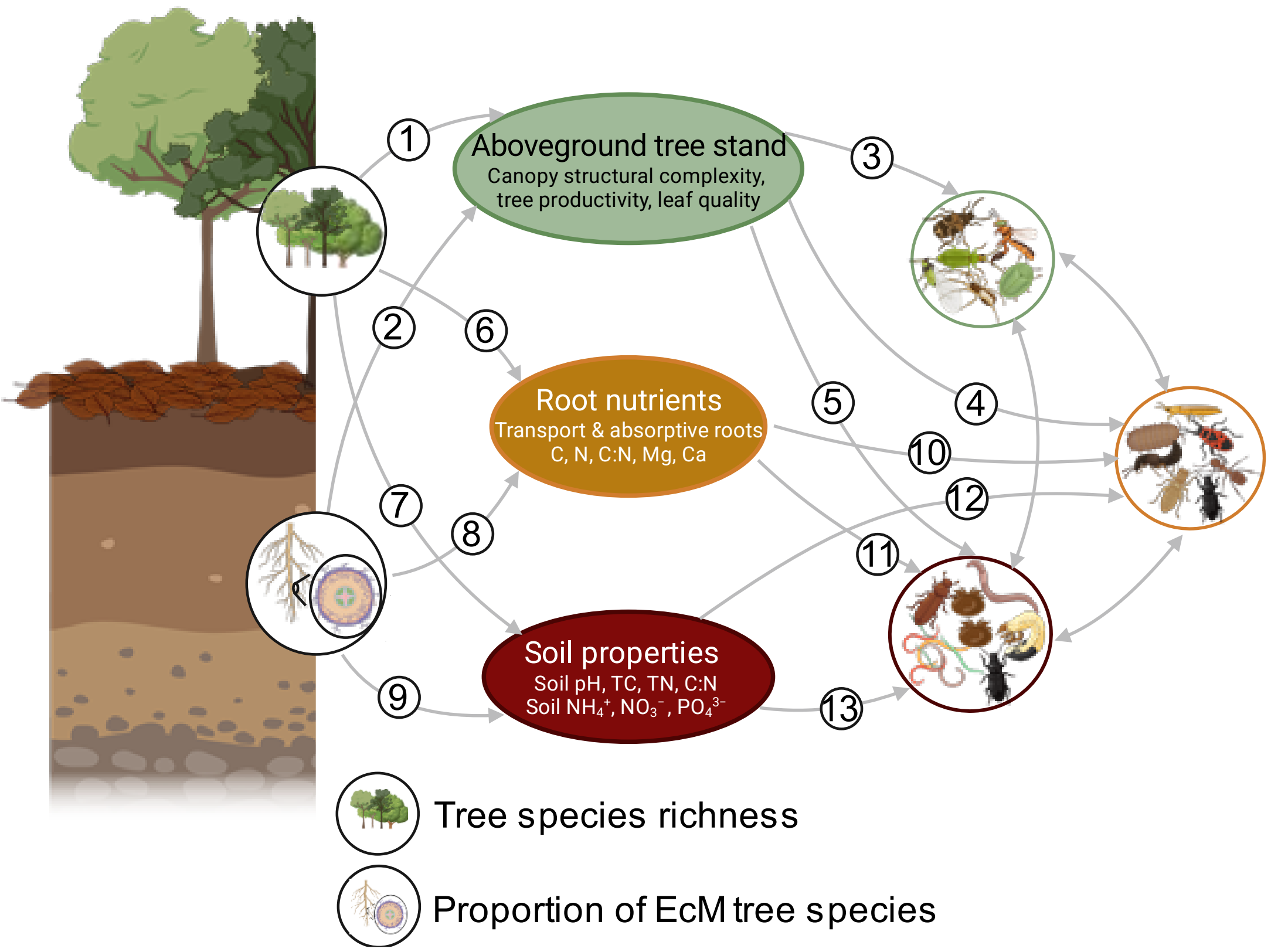

Figure S1: Conceptual figure of the hypothesized structural equation model (SEM) framework for tree and mycorrhizal type effects as well as environmental mediators on multitrophic abundance and diversity across above- and belowground food webs. The invertebrates across the aboveground (green bubbles), soil surface (orange bubbles), and belowground (brown bubbles) strata are displayed on the right side of the framework. Arrows indicate proposed causal relationships. We tested how tree species richness and mycorrhizal types (the proportion of EcM tree species; EcM%) influence multitrophic metrics via a range of mediators representing the aboveground tree stand, root nutrients, and soil properties. Mycorrhizal type was coded as the proportion of EcM tree species (1 = EcM, 0.5 = AM+EcM, 0 = AM). As this is a conceptual figure, the final analysis included only mediators that were significantly affected by the treatment factors (see Table S9, Fig. S6) and were not strongly correlated with each other (Table S10). The theoretical basis for each pathway (indicated with number in the cycles) is described below. Created in BioRender [34].

Pathways 1-2: tree diversity and mycorrhizal types → aboveground tree stand characteristics

We hypothesized that tree diversity influences above- and belowground communities indirectly through changes in canopy structure complexity [35,36], productivity [37,38], and leaf quality [39]. In addition, we assumed that these variables mediate the effects of mycorrhizal type [40–43]. Specifically, we assumed that tree canopy structural complexity (measured as the effective number of layers, ENL), aboveground tree productivity (total wood volume), and leaf quality (leaf nitrogen content and C:N ratio) act as key mediators linking tree diversity to biotic communities.

Pathway 3: aboveground tree stand characteristics → aboveground biodiversity

For aboveground communities, we expected canopy structural complexity to increase habitat heterogeneity and resource availability, thereby promoting the abundance and diversity of foliar fungi [15] and invertebrates [44,45]. Higher tree productivity was expected to increase the overall availability of plant-derived resources [46], while leaf quality was assumed to influence herbivore and foliar fungal communities through changes in leaf nutrient content and palatability [47,48]. These effects were expected to propagate to higher trophic levels such as predators [6].

Pathway 4-5: aboveground tree stand characteristics → soil surface and belowground biodiversity

For soil-surface and belowground communities, we hypothesized that canopy structural complexity enhances microclimatic stability (e.g., temperature buffering and soil moisture) [49,50], which benefits invertebrates and microbial communities. In addition, higher aboveground productivity was expected to increase carbon inputs to the soil through root growth, root exudates, and litter production, thereby supporting microbial communities, detritivores, and higher trophic levels [51–55]. Finally, leaf quality was expected to indicate litter quality and decomposition processes, affecting resource availability for microbial communities [42] and microhabitats for litter and soil fauna [56,57].

Pathway 6-9: tree diversity and mycorrhizal types → soil properties and root nutrients

For soil-surface and belowground communities, we hypothesized that the effects of tree diversity are mediated by soil properties [39,58] and root nutrient contents [59–61]. These variables are also expected to mediate the effects of mycorrhizal type [42,62–65]. Specifically, we assume that soil properties (soil pH, soil total C and N contents, and C:N ratio), soil nutrient availability (NO_3^-, NH_4^+, and PO_4^{3-}), and nutrient contents in both transport and absorptive roots (e.g., C, N, Ca, Mg, and root C:N ratio) act as key mediators linking tree diversity and mycorrhizal types to soil-surface and belowground communities.

Pathway 10-11: root nutrients → belowground and soil-surface biodiversity

Root nutrient contents can determine the palatability of roots for root-feeding organisms [66]. Following root turnover, nutrients contained in root litter further regulate the abundance of detritivores, such as earthworms [67,68], and influence soil microbial community structure. These effects can propagate through the entire soil food web [69,70]. In addition, roots, particularly those of ectomycorrhizal (EcM) trees, often forage for nutrients in the top-soil and litter layer, which may also influence soil-surface communities [71].

Pathway 12-13: soil properties → belowground and soil surface biodiversity

Soil properties directly determine the structure of soil microbial communities and nematode assemblages [42,72–74]. For example, higher nutrient availability was expected to promote soil bacteria and bacterivorous nematodes, whereas higher soil organic matter and carbon contents are likely to favor soil fungi and fungivorous nematodes [72,75,76]. Soil carbon content and the soil C:N ratio have also been shown to be key predictors of microbial biomass [77] and oribatid mite diversity [78].

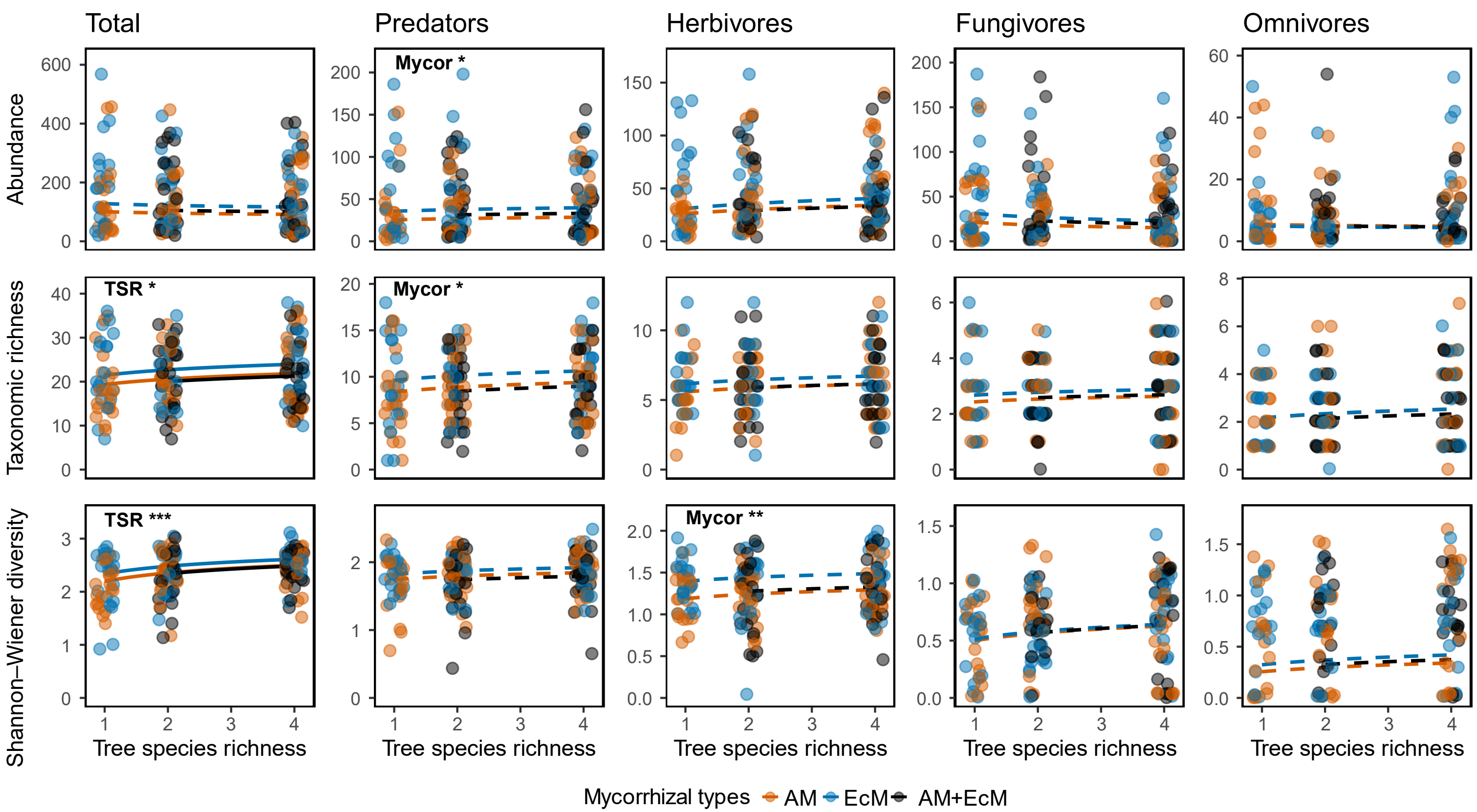

Figure S2: Effects of tree species richness (TSR), mycorrhizal type (Mycor), and their interaction on the abundance, taxonomic richness and Shannon-Wiener diversity of trophic groups in the aboveground compartment. The significance of fixed effects (TSR, Mycor, and TSR × Mycor) is indicated within each panel. Significance levels are indicated as *** for $p < 0.001$, ** for $0.001 \leqslant p < 0.01$, and * for $0.01 \leqslant p < 0.05$. Effects with $p \geqslant 0.05$ are not labeled for the visual clarity. Solid lines indicate significant effects of tree species richness, whereas dashed lines indicate non-significant effects. Points represent observed values (n = 160), and lines show model-predicted responses from linear mixed-effects models fitted as: lme(y_i^t ~ month + Mycor × log(Richness), random = ~1 | composition/month, data). Trophic groups are labeled at the top of each column. Abundance (upper row; number of individuals per 4 m² sampling area), taxonomic richness (middle row; number of taxa, with most organisms identified to the family level) and Shannon–Wiener diversity (lower row) are shown separately.

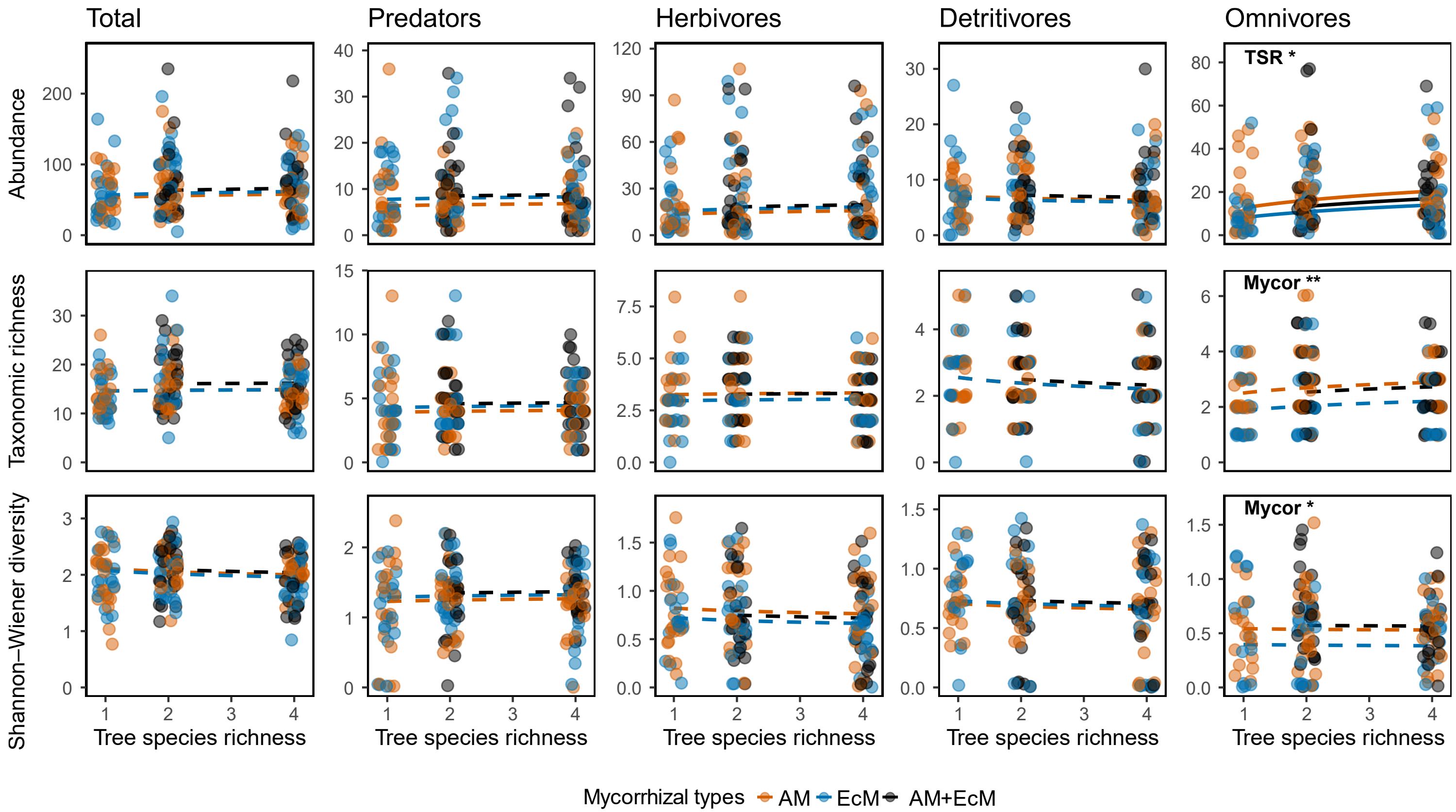

Figure S3: Effects of tree species richness (TSR), mycorrhizal type (Mycor), and their interaction on the abundance, taxonomic richness and Shannon-Wiener diversity of trophic groups in the soil surface compartment. The significance of fixed effects (TSR, Mycor, and TSR × Mycor) is indicated within each panel. Significance levels are indicated as *** for $p < 0.001$, ** for $0.001 \leqslant p < 0.01$, and * for $0.01 \leqslant p < 0.05$. Effects with $p \geqslant 0.05$ are not labeled for the visual clarity. Solid lines indicate significant effects of tree species richness, whereas dashed lines indicate non-significant effects. Points represent observed values (n = 160), and lines show model-predicted responses from linear mixed-effects models fitted as: lme(y_i^t ~ month + Mycor × log(Richness), random = ~1 | composition/month, data). Trophic groups are labeled at the top of each column. Abundance (upper row; number of individuals per 0.5×0.5 m² sampling area), taxonomic richness (middle row; number of taxa, with most organisms identified to the family level) and Shannon–Wiener diversity (lower row) are shown separately.

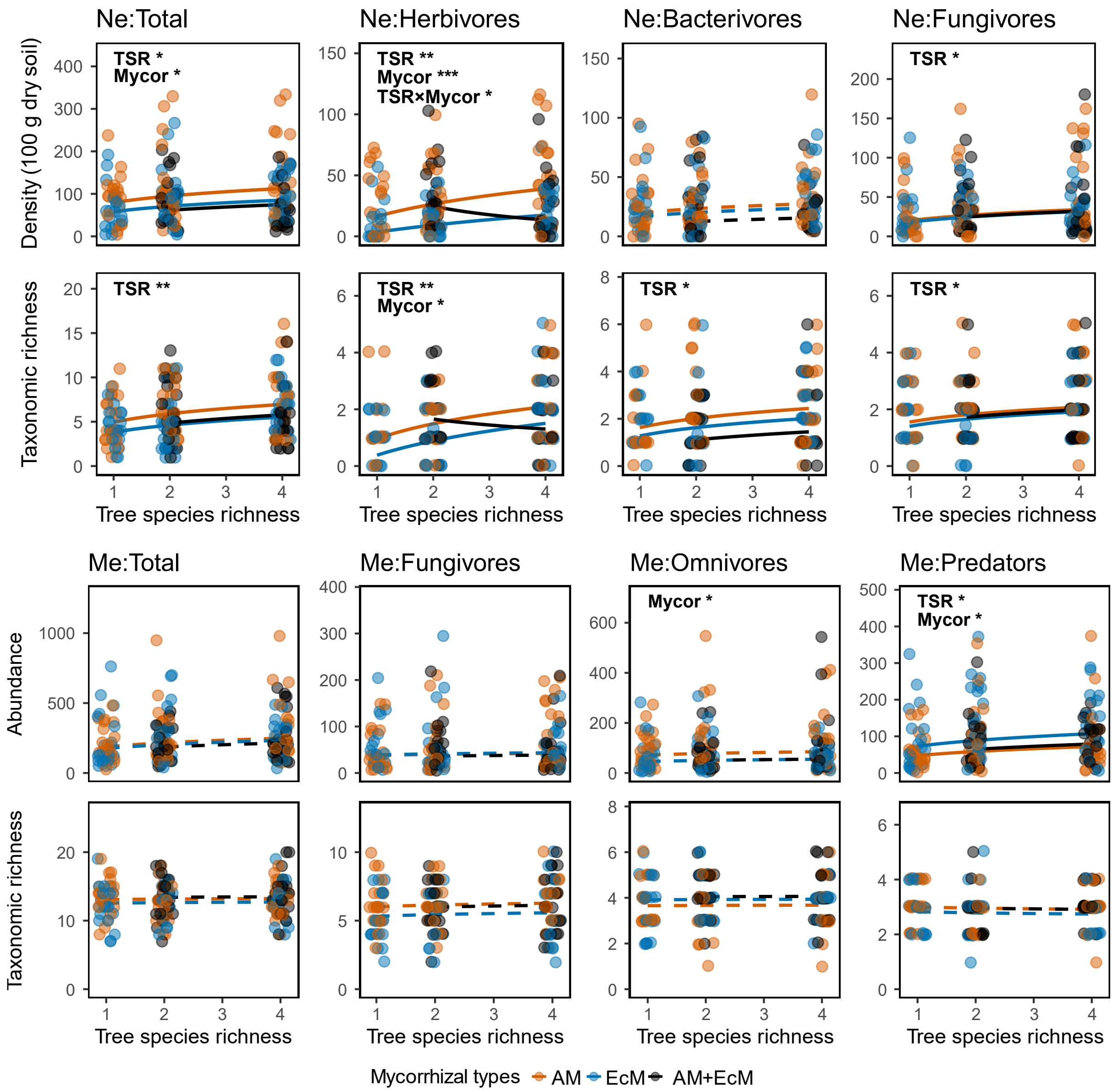

Figure S4: Effects of tree species richness (TSR), mycorrhizal type (Mycor), and their interaction on the abundance and taxonomic richness of trophic groups in the belowground compartment. The significance of fixed effects (TSR, Mycor, and TSR × Mycor) is indicated within each panel. Significance levels are indicated as *** for $p < 0.001$, ** for $0.001 \leqslant p < 0.01$, and * for $0.01 \leqslant p < 0.05$. Effects with $p \geqslant 0.05$ are not labeled for clarity. Solid lines indicate significant effects of tree species richness, whereas dashed lines indicate non-significant effects. Points represent observed values (n = 160), and lines show model-predicted responses from linear mixed-effects models fitted as: lme(y_i^t ~ month + Mycor × log(Richness), random = ~1 | composition/month, data). Trophic groups are labeled at the top of each column. The first two rows represent nematode trophic groups (Ne), with density (number of individuals per 100 g dry soil) in the first row and taxonomic richness (number of taxa, with most identified to the genus level) in the second row. The last two rows represent mesofauna trophic groups (Me), with abundance in the third row (total number of individuals per soil core in each plot, 16 cm diameter and 10 cm depth) and taxonomic richness in the fourth row (number of taxa, with most groups identified to the suborder level).

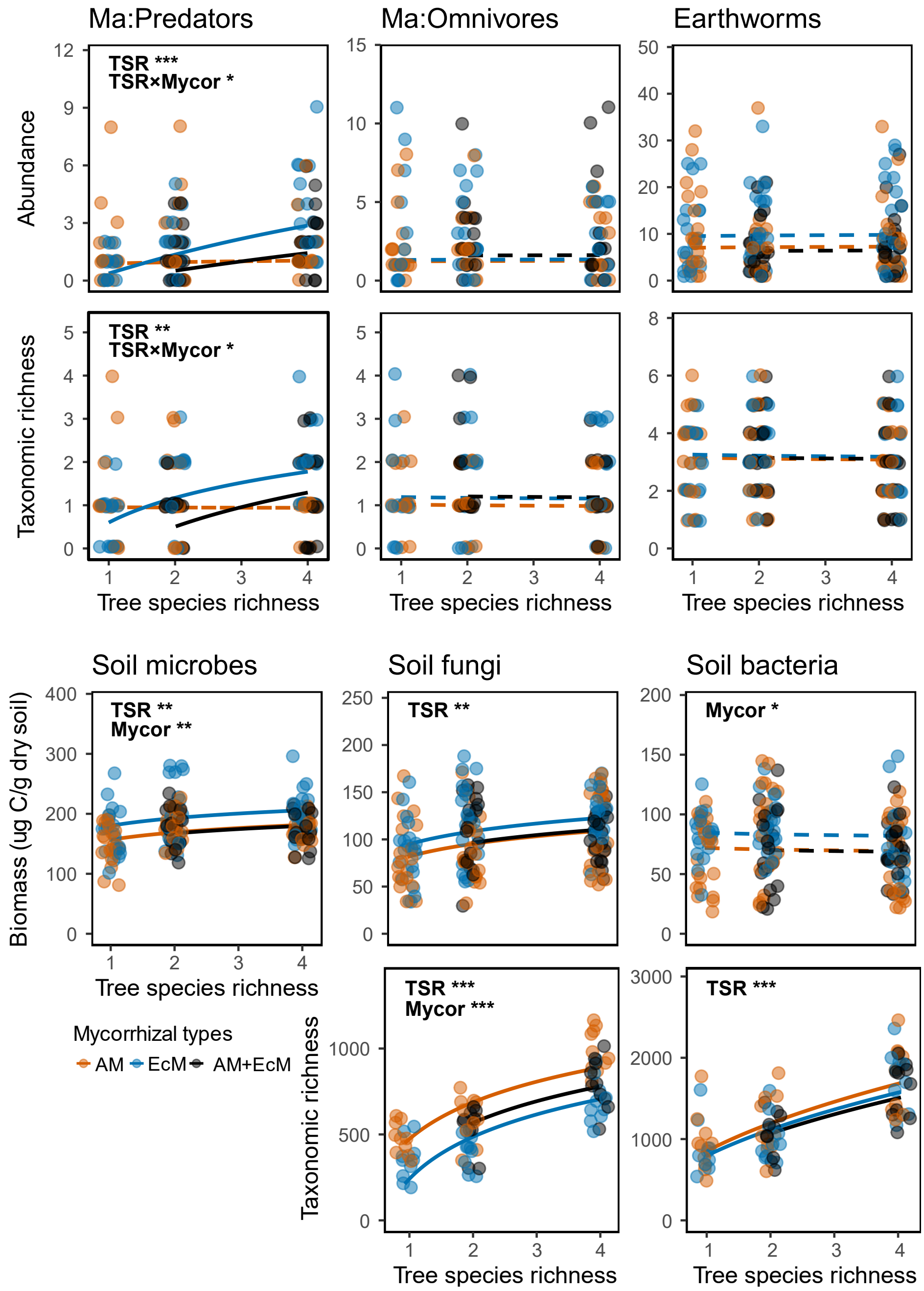

Figure S4 (continued): Effects of tree species richness (TSR), mycorrhizal type (Mycor), and their interaction on the abundance and taxonomic richness of macrofauna (Ma) and soil microbial communities in the belowground compartment. Trophic groups are labeled at the top of each column. Abundance of macrofauna (predators and omnivores) represents the total number of individuals per soil core (16 cm diameter, 10 cm depth), while taxonomic richness represents the number of identified families. For earthworms, abundance corresponds to the total number of individuals sampled from a 0.5 × 0.5 m² area using a mustard extraction solution, and taxonomic richness represents the number of identified species. These metrics are shown in the upper rows. Points represent observed values (n = 160). The lower rows indicate microbial biomass (as a proxy for abundance, n = 160) and estimated taxonomic richness (Chao1; n = 80). Other details, including model specification, significance levels, and line types, are as described in Fig. S3.

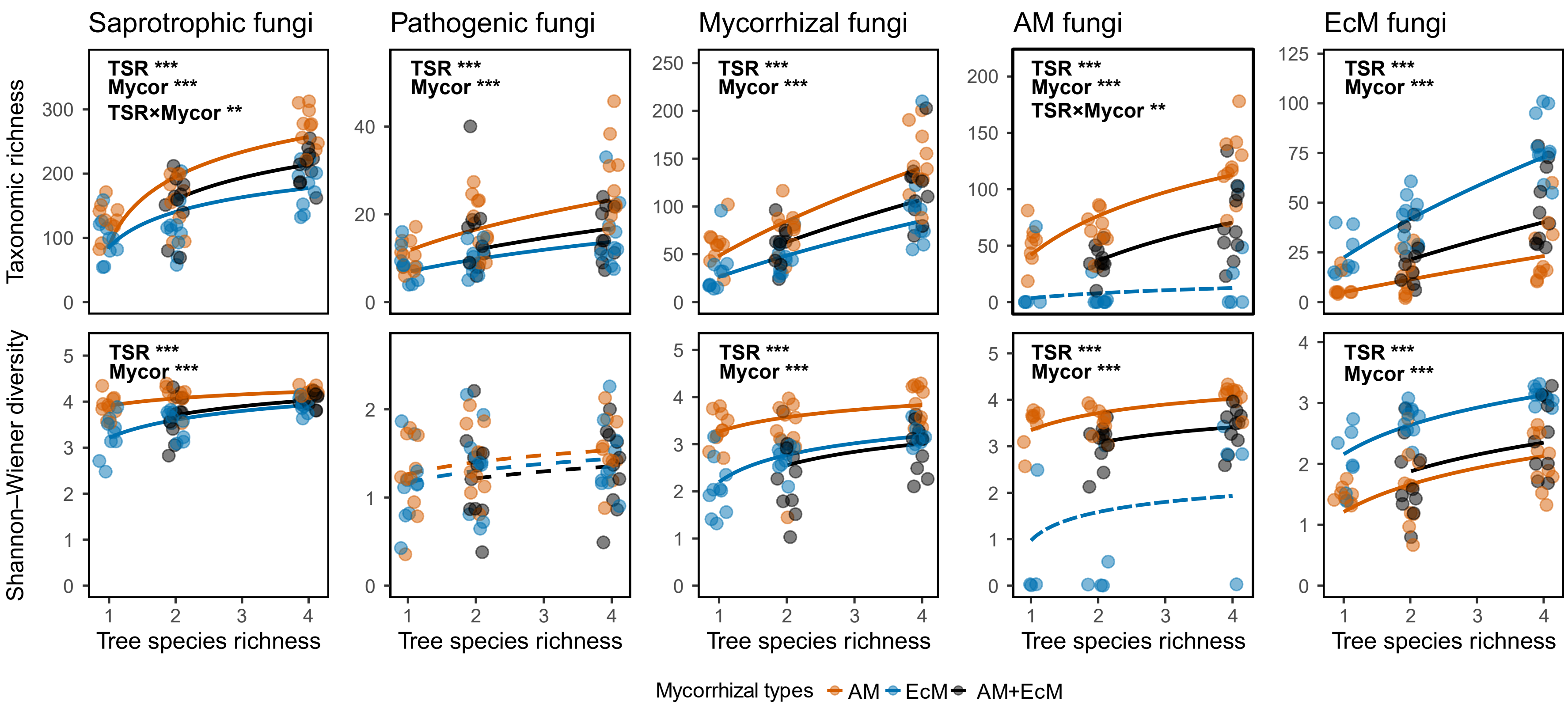

Figure S5: Effects of tree species richness (TSR), mycorrhizal type (Mycor), and their interaction on the taxonomic richness and Shannon-Wiener diversity of fungal functional groups. The significance of fixed effects (TSR, Mycor, and TSR × Mycor) is indicated within each panel. Significance levels are indicated as *** for $p < 0.001$, ** for $0.001 \leqslant p < 0.01$, and * for $0.01 \leqslant p < 0.05$. Effects with $p \geqslant 0.05$ are not labeled for clarity. Solid lines indicate significant effects of tree species richness, whereas dashed lines indicate non-significant effects. Points represent observed values (n = 80), and lines show model-predicted responses from linear mixed-effects models fitted as: lme (y_i^t ~ Mycor × log(Richness), random = ~1 | composition, data). Fungal functional groups are labeled at the top of each column; taxonomic richness (Chao1; upper row) and Shannon–Wiener diversity (lower row) are shown separately.

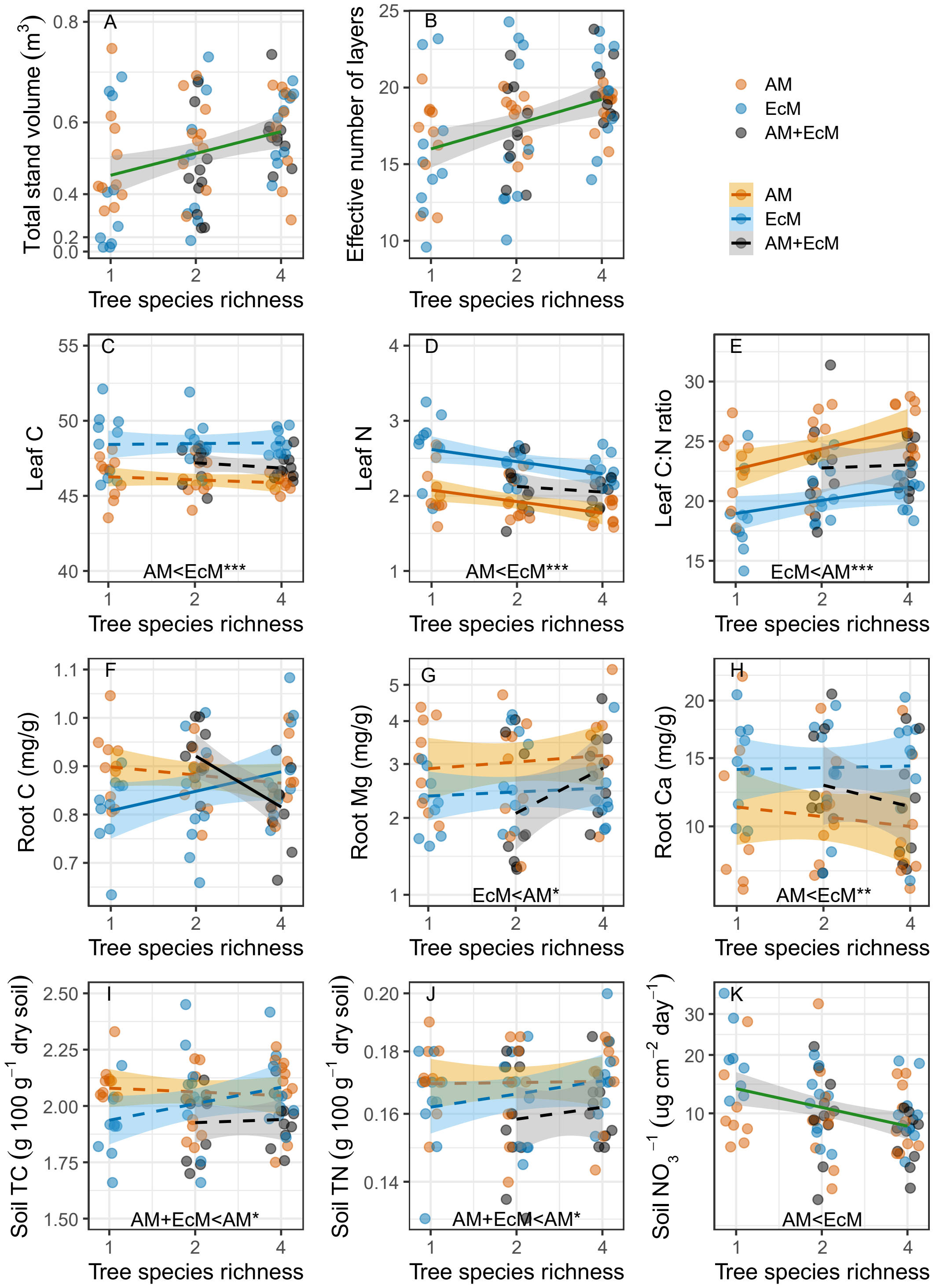

Figure S6. Relationships between parameters for aboveground tree stand (A–C), root nutrient (D–F) and soil properties (G–I) and tree species richness across three mycorrhizal type treatments. Significant relationships with tree species richness are shown with solid lines (± SE), while non-significant relationships are shown with dashed lines. Points represent observed values (n = 80). Differences among mycorrhizal types are indicated by lines in different colors and labeled at the bottom of each panel; where no difference is present, a single green line is shown. Statistical results from the mixed-effects models are provided in Table S9.

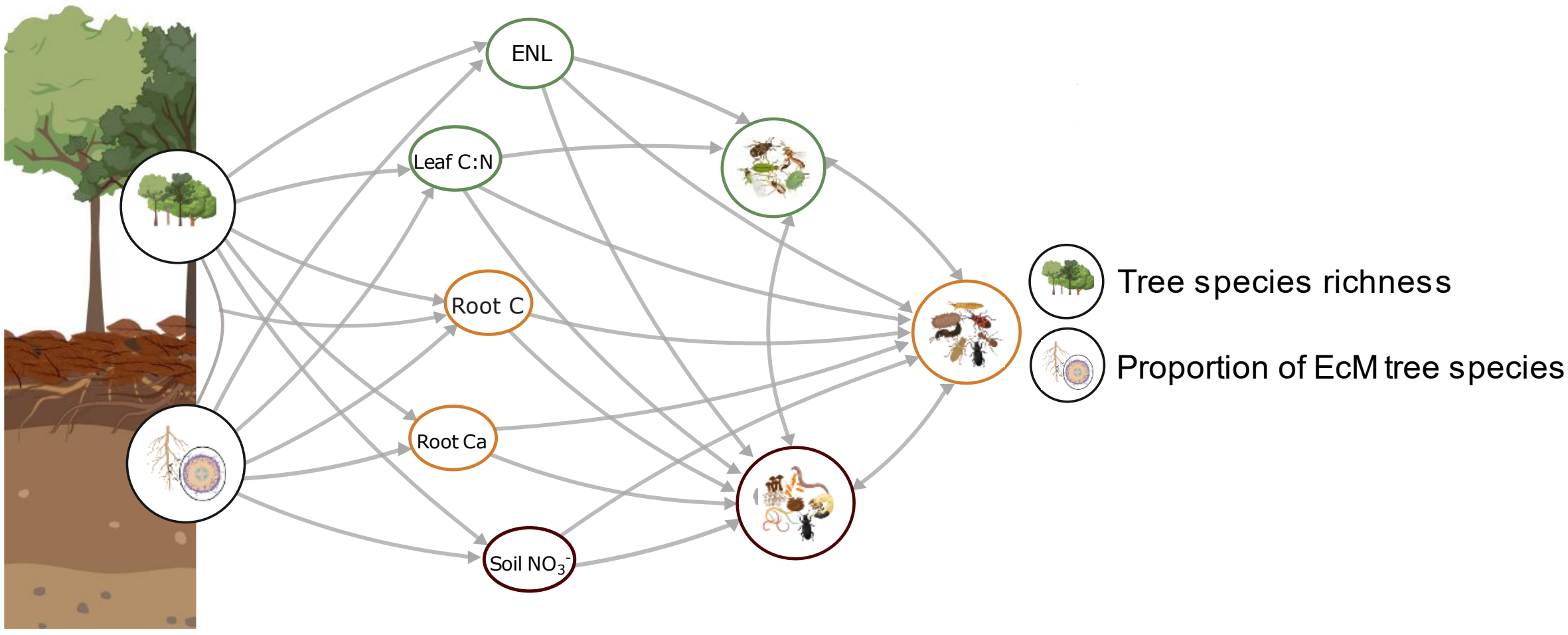

Figure S7: Simplified framework of the hypothesized structural equation model (SEM) for multitrophic abundance and diversity above- and belowground food webs. This is the final framework used for the statistical analysis (see Supplementary Methods: Construction of Structural Equation Models). It was simplified from the hypothesized framework in Fig. S1 by selecting the parameters significantly affected by the treatment factors (Fig. S6) and retaining only those that were not significantly correlated with each other (Tables S9-S10). More specifically, we tested how tree species richness and mycorrhizal types (the proportion of EcM tree species) influence multitrophic metrics via mediators: aboveground tree stand characteristics (ENL and leaf C:N ratio), root C and Ca, and soil NO_3^-. Mycorrhizal type was coded as the proportion of EcM tree species (1 = EcM, 0.5 = AM+EcM, 0 = AM), but soil C was excluded due to non-additive responses (e.g., the lowest values in AM+EcM plots; Fig. S6). A richness × mycorrhizal interaction was included for root C. The invertebrates across the aboveground (green bubbles), soil surface (orange bubbles), and belowground (brown bubbles) strata are displayed on the right side of the framework. Arrows indicate proposed causal relationships. Direct paths from tree species richness and mycorrhizal types to multitrophic abundance or diversity were retained only if significant/marginal after accounting for mediators ($p < 0.1$). For clarity, covariances between mediators (double arrows) are not shown but were tested and retained only if significant/marginal ($p < 0.1$). Icons from BioRender [34].

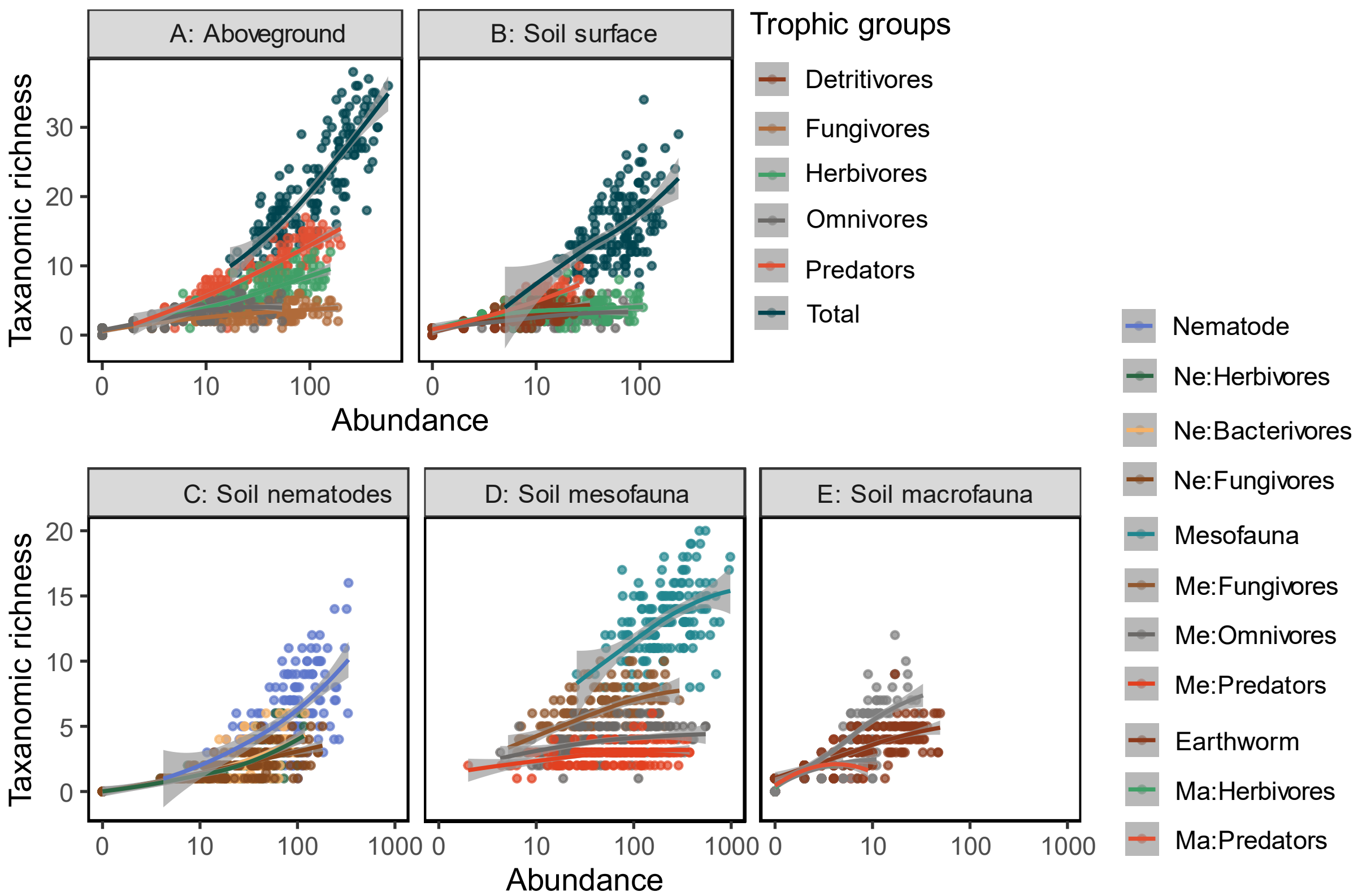

Figure S8. Scatterplots showing the correlation between abundance and taxonomic richness for each trophic group across (A) aboveground, (B) soil surface, and (C–E) belowground compartments. Belowground invertebrates are presented separately as (C) soil nematodes, (D) soil mesofauna, and (E) soil macrofauna. Different functional groups are distinguished by colors. Belowground organisms are abbreviated as Ne (nematodes), Me (mesofauna), and Ma (macrofauna). In general, taxonomic richness increased with abundance. Note: the x-axis (Abundance) is $\log_{10}$-transformed. Points represent observed values, with colors indicating different trophic groups (n = 160 per group).

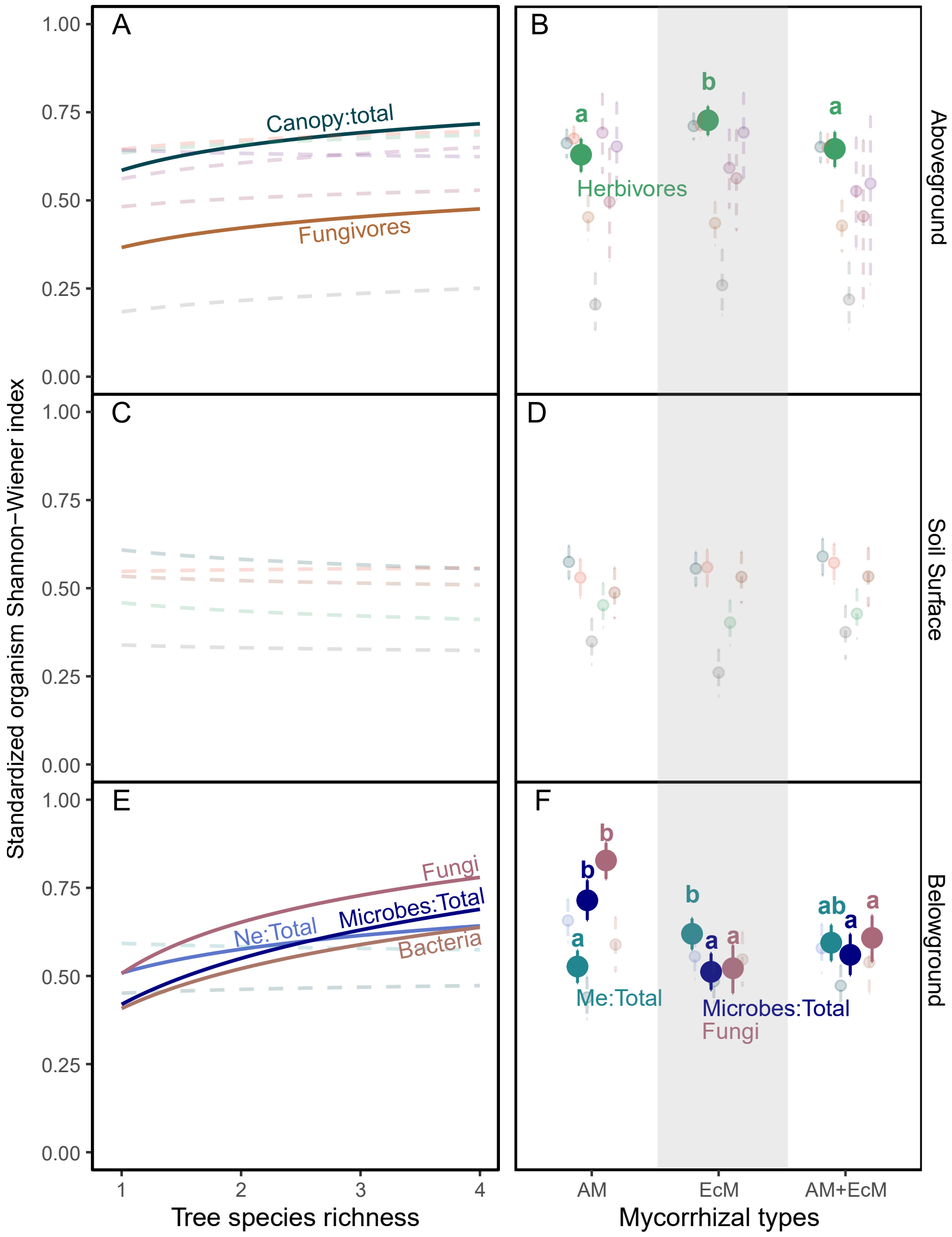

Figure S9: Effects of tree species richness and mycorrhizal types on organism Shannon-Wiener diversity aboveground (A-B), at the soil surface (C-D), and belowground (E-F). All response variables were scaled to [0,1]. Linear mixed-effects models with two-sided tests were fitted separately for each trophic group within each stratum using plot-level observations. Invertebrate models included 160 observations from 80 plots sampled in two seasons. Soil microbial groups were sampled once across 80 plots, and foliar fungal groups were sampled once with fewer plot-level observations (endophytes, n = 39; total foliar fungi and epiphytic fungi, n = 34 each). Curves (A, C, E) show prediction across the tree species richness gradient; solid lines indicate significant effects ($p < 0.05$), while dashed and transparent lines indicate non-significant effects. Estimated means and 95% confidence intervals for each mycorrhizal type (B, D, F) were obtained using *emmeans* from the fitted models. Solid dots and letters (a, b, ab) denote significant differences among mycorrhizal types after Tukey adjustment ($p < 0.05$); transparent dots indicate non-significant differences. Group names are shown next to lines or points when responses to tree species richness or mycorrhizal type were significant. Belowground organisms are abbreviated as Ne (nematodes), Me (mesofauna), and Ma (macrofauna). Colors correspond to organism groups. See Tables S1-3 for power transformation values and Tables S4-8 for F-values, p-values, degrees of freedom and model R^2 of each model.

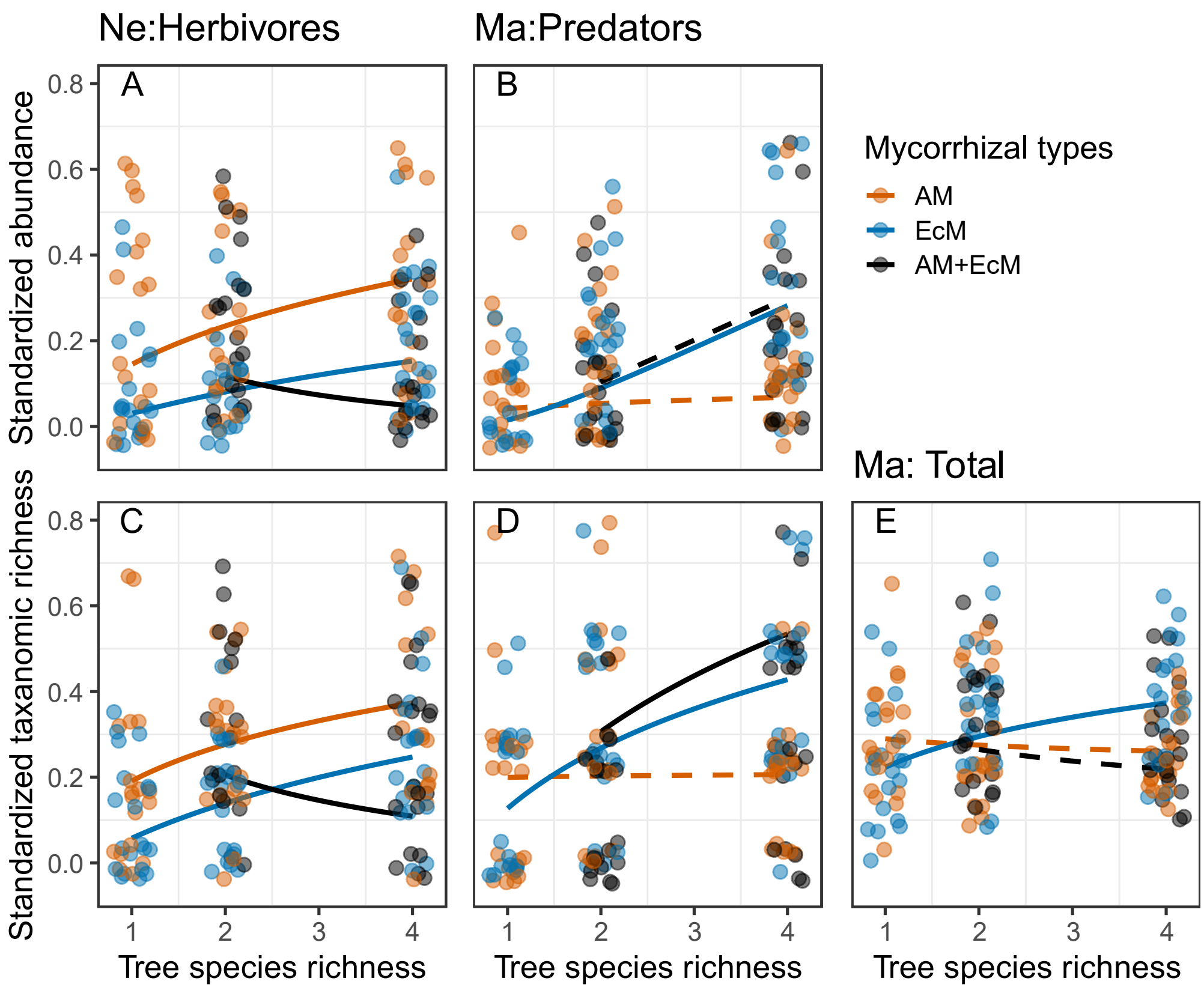

Figure S10. Effects of mycorrhizal type, tree species richness, and their interaction on standardized abundance (A–B) and taxonomic richness (C–E). Only the trophic groups that showed significant interaction effects are presented here (Tables S4–S8). Colors indicate mycorrhizal types. Solid lines represent significant relationships; dashed lines represent non-significant ones. Lines were predicted from the model *lme*(y_i^t ~ Month + Mycor × log(Richness), random = ~1 | composition/Month, data). For visualization, predictions were averaged across sampling seasons using the *emmeans* package. Panels within each column correspond to the same trophic group: nematode herbivores (Ne:Herbivore), soil macrofauna predators (Ma:Predators), and total soil macrofauna (Ma:Total). Points represent observed values (n = 160).

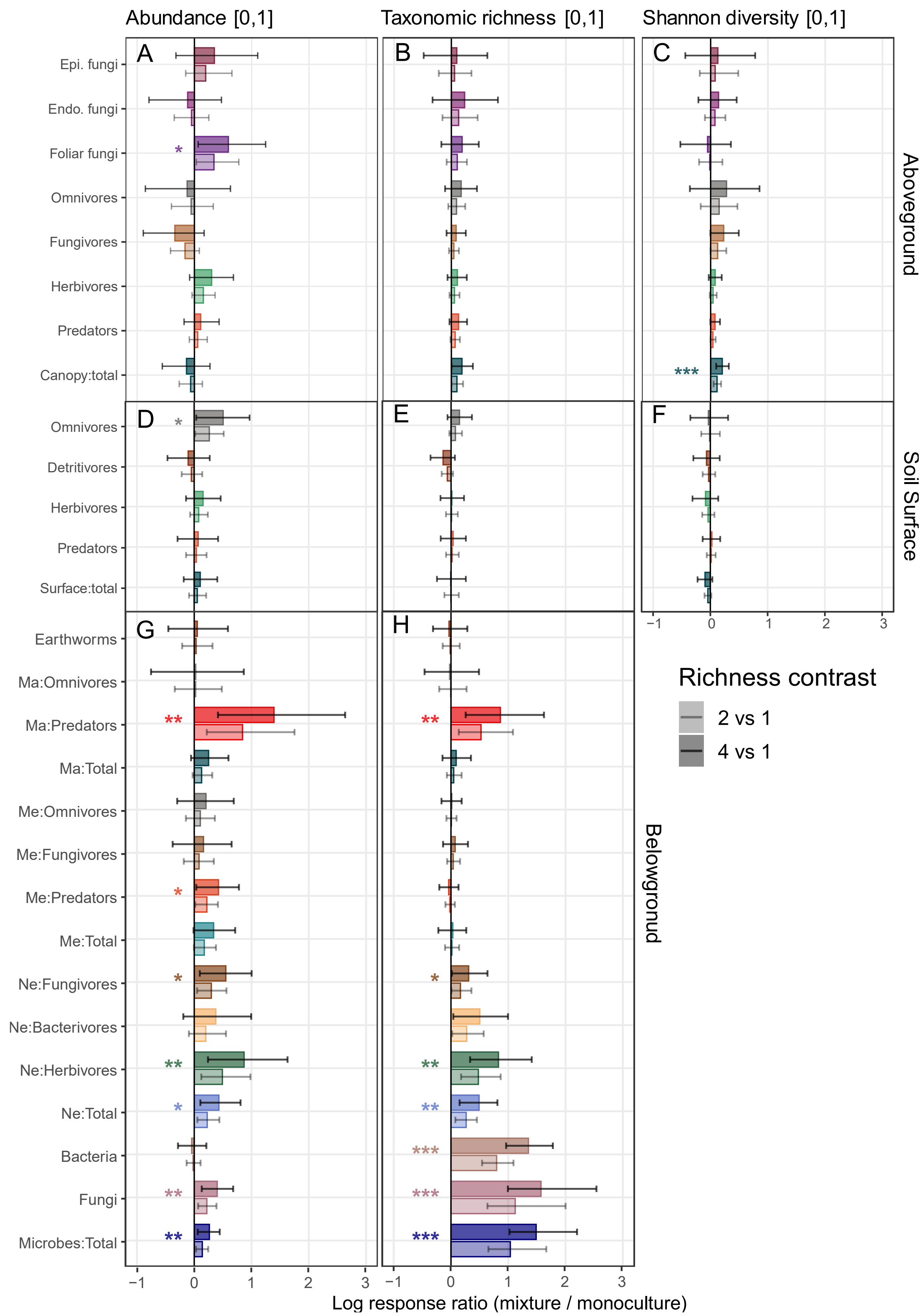

Figure S11. Standardized tree diversity effect sizes on the abundance (A, D, G), taxonomic richness (B, E, H), and Shannon–Wiener diversity (C, F) across trophic

groups and food-web compartments (Aboveground, Soil surface, and Belowground). Effect sizes were calculated from predicted values for monocultures and tree mixtures (2- and 4-species; indicated with different transparency) obtained from linear mixed-effects models (*lme*: y_i^t ~ Month + Mycor + log(Richness)) fitted to min–max transformed data. The linear mixed-effects models with two-sided tests were fitted separately for each diversity index of each trophic group within each stratum using plot-level observations. Invertebrate models included 160 observations from 80 plots sampled in two seasons. Soil microbial abundance was sampled twice across 80 plots (n = 160), but soil microbial taxonomic richness and Shannon-Wiener diversity were only assessed once across 80 plots (n = 80). Foliar fungal groups were sampled once with fewer plot-level observations (endophytes, n = 39; total foliar fungi and epiphytic fungi, n = 34 each). Effect sizes were expressed as the log-response ratio, log(mixture/monoculture). Asterisks (*) indicate cases where the effect sizes for both 2- and 4-species mixtures are significantly greater than zero (95% confidence intervals do not overlap zero). These significance levels correspond to those shown in Figs. 1-2 and Fig. S9. For trophic groups not significantly affected by tree diversity, the 95% confidence intervals overlap zero. For belowground trophic groups, abbreviations are as follows: Ne = nematodes, Me = mesofauna, and Ma = macrofauna. The magnitude of biodiversity change for each trophic group was calculated as exp(log-response ratio) − 1, representing the percentage change relative to monocultures in two- and four-species mixtures.

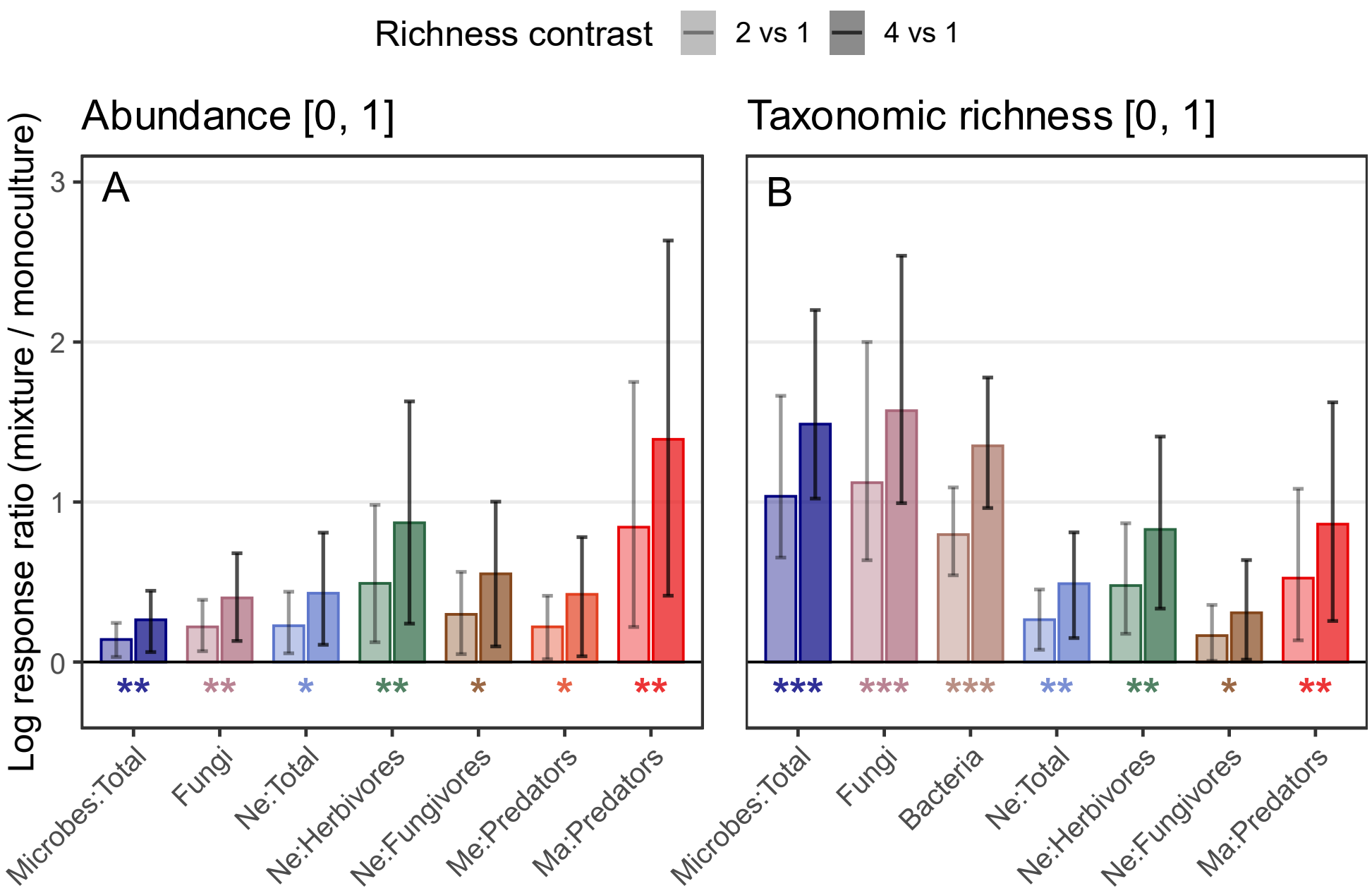

Continued Fig. S11. We zoomed in on the belowground trophic groups (G & H) that responded significantly to tree diversity. This allows comparison of effect sizes across trophic groups and helps address Hypothesis (1b). Based on the overlap of the 95% confidence intervals, the effect sizes do not differ significantly among trophic groups.

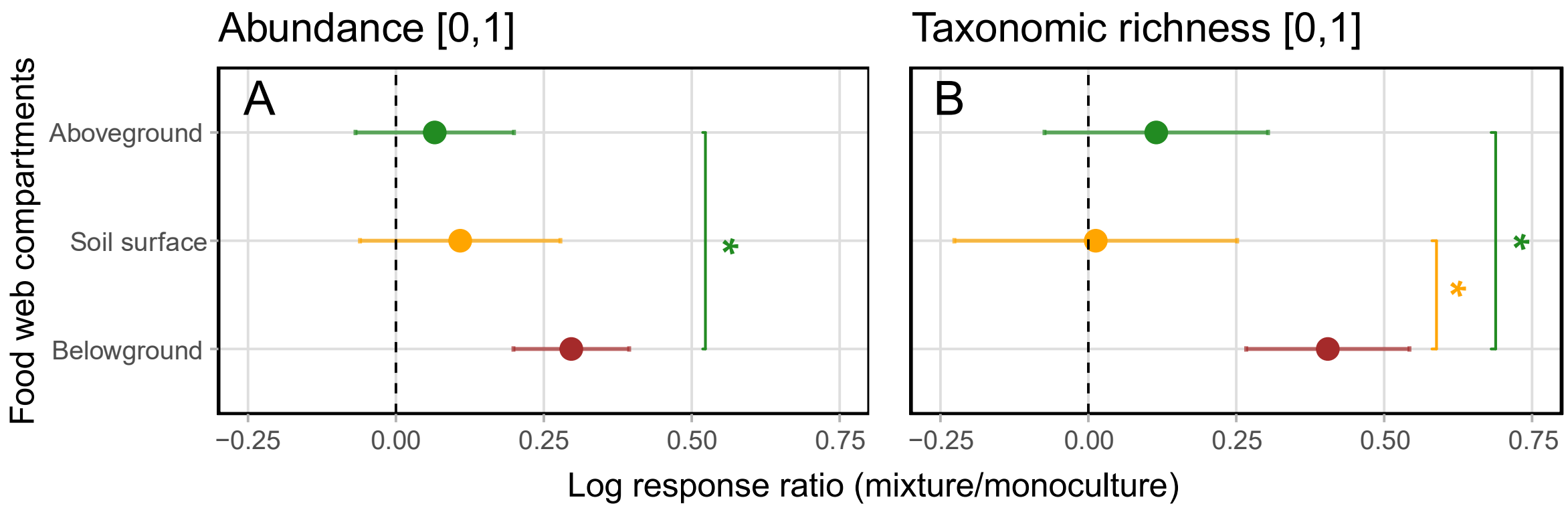

Figure S12. Averaged effect sizes of trophic groups across three food-web compartments. This figure was derived from Fig. S11 by extracting the mean effect sizes of trophic groups within each compartment for both abundance (A) and taxonomic richness (B). Eight groups were from aboveground compartment, five groups were from soil surface, and 15 groups were from belowground compartment. Differences in effect sizes were tested using the model (mean(log-response ratio) ~ Stratum × Richness contrast) (n = 56). Richness contrast (2- and 4- species) had no significant effect on the averaged effect sizes, whereas effect sizes differed among food-web compartments, indicated by "*" ($p < 0.05$). Notably, these results may be biased, because more trophic groups were included in the belowground compartment than for aboveground and at the soil surface compartments, and we did not account for variation among effect sizes across trophic groups. Therefore, this figure should be used only as a summary reference, while the main results rely on Fig. S11 and Figs. 1&2.

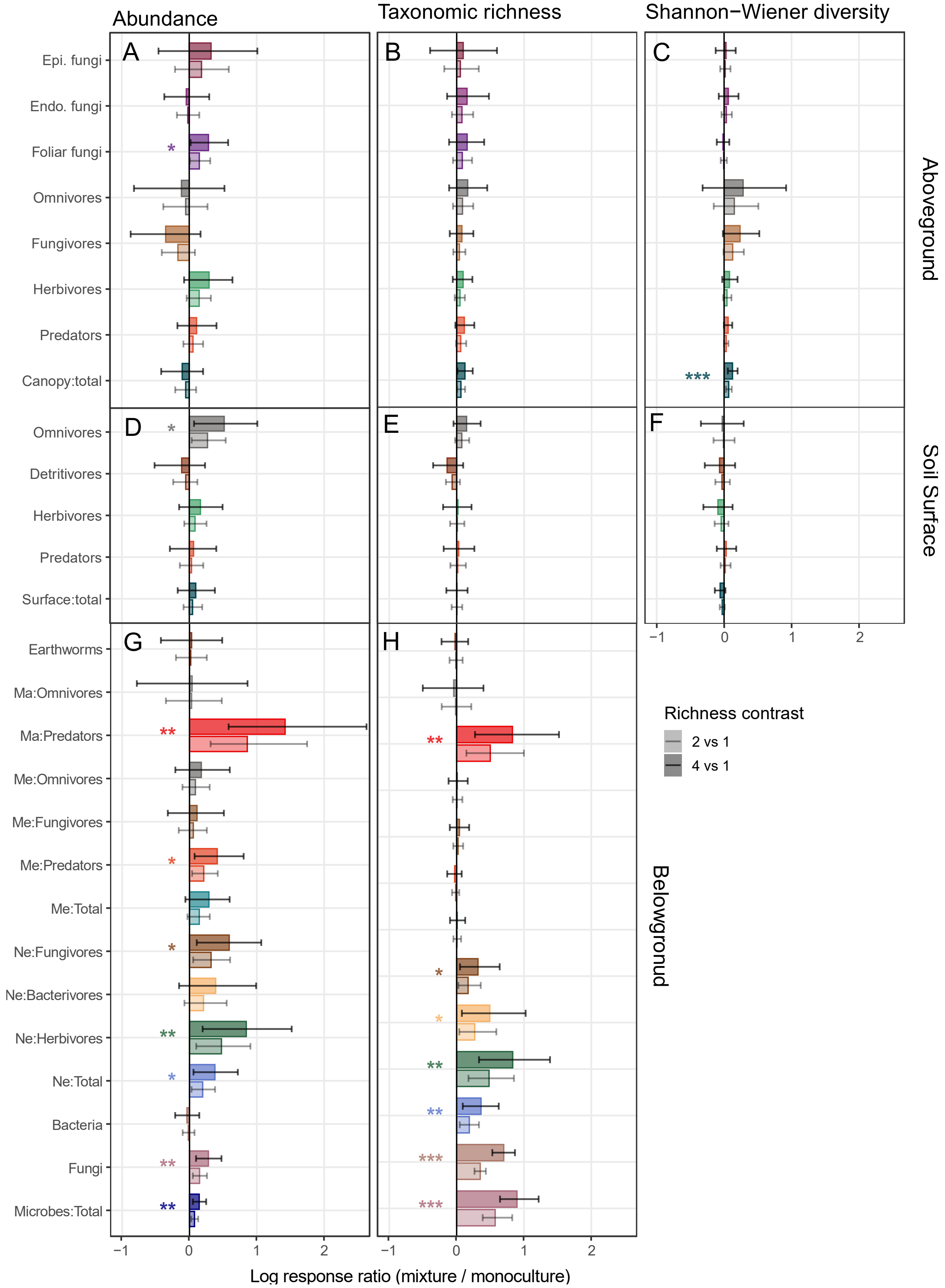

Figure S13. Standardized tree diversity effect sizes on the abundance (A, D, G), taxonomic richness (B, E, H), and Shannon–Wiener diversity (C, F) across trophic groups and food-web compartments. This figure follows the same structure and pattern as Fig. S11, but is based on predictions from models fitted to the original data.

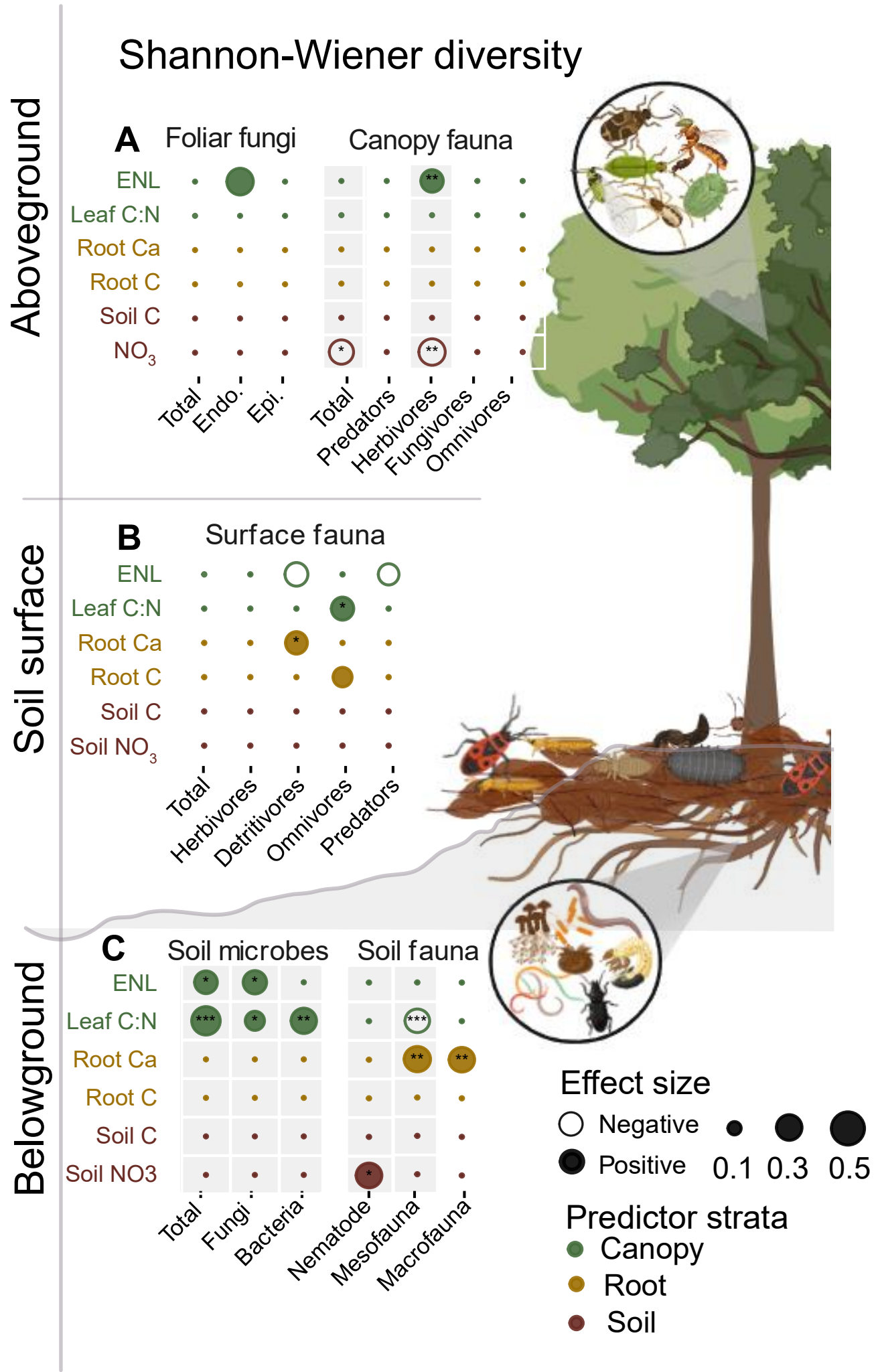

Figure S14. Results of multiple linear regressions showing the effects of six mediators on standardized organism Shannon-Wiener diversity index aboveground (A), at the soil surface (B), and belowground (C). Within each panel, individual trophic groups are shown as columns along the x-axis, grouped into broader categories (e.g., foliar fungi, canopy fauna, soil microbes, soil fauna) labeled at the top. Linear mixed-effects models with two-sided tests were fitted separately for each trophic group within each stratum, using all six proposed explanatory variables. Sample sizes were n = 80 for soil microbes and fauna groups, n = 39 for endophytes, and n = 34 for total foliar fungi and epiphytic fungi. Only variables retained in the best-fit models after backwards selection with the lowest AICc are shown. Standardized coefficients of retained explanatory variables are represented by circles, with filled circles indicating positive effects and open circles indicating negative effects; circle size is proportional to the absolute standardized coefficient. Non-selected variables are shown as small dots. Statistical significance is indicated by asterisks: * $0.01 \le p < 0.05$, ** $0.001 \le p < 0.01$, ***$p < 0.001$. Colors indicate the source of each explanatory variable: green for canopy conditions (leaf C:N ratio, ENL), light brown for root nutrients, and earthy brown for soil properties. Marginal R^2 (R^2m; fixed effects only) and conditional R^2 (R^2c; fixed and random effects) are listed in Tables S15-S17. Panels are shaded when the biodiversity response was significantly affected by tree species richness and/or mycorrhizal type (see Fig. S9). Icons from BioRender [34].

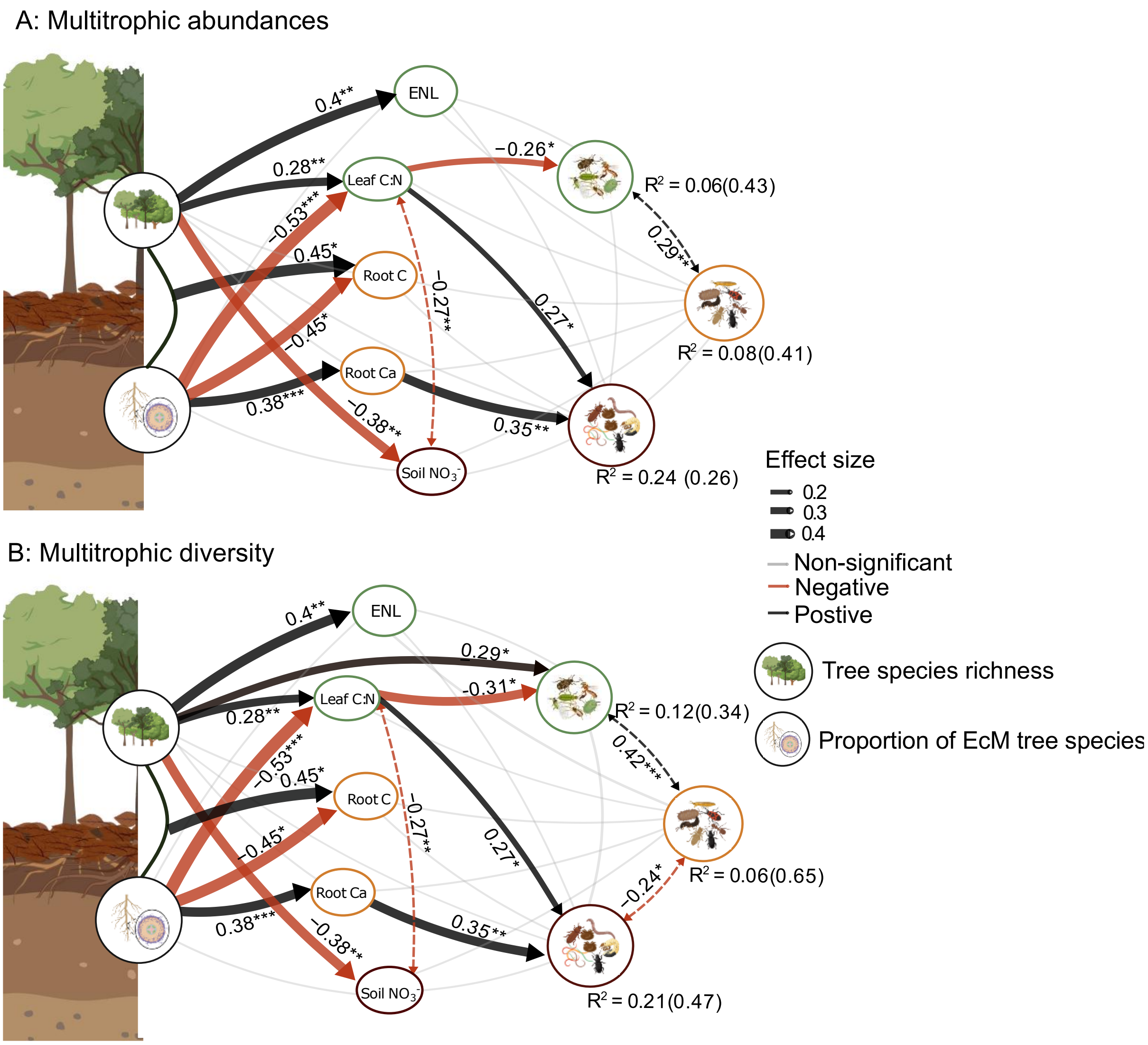

Figure S15. Sensitivity analysis on how tree species richness and mycorrhizal type (proportion of EcM tree species) affect multitrophic abundance (A) and diversity (B) of invertebrates. The invertebrates across the aboveground (green bubbles), soil surface (orange bubbles), and belowground (brown bubbles) strata are displayed on the right side of each model. The multitrophic abundance/diversity were calculated by using z-score transformation. Pathways include direct and indirect effects through tree stand characteristics (ENL and leaf C:N ratio), root C and Ca, and soil NO_3^-. Panel (A) represents multitrophic abundance (Fisher's C = 34.98, df = 44, p = 0.83); panel (B) shows multitrophic diversity (Fisher's C = 32.48, df = 44, p = 0.90). Significant and marginal paths ($p < 0.1$) are shown as solid black (positive) or red (negative) lines; non-significant paths are shown in grey. Significant covariances are indicated with dashed lines with double arrows. Standardized path coefficients are shown along each line, with significance denoted as *** ($p < 0.001$), ** ($0.001 \leq p < 0.01$), and * ($0.01 \leq p < 0.05$). Interactive effects of tree species richness and mycorrhizal type on root C are shown by a connecting line between two icons. Conditional R^2 values for each final endogenous variable are noted next to their icons (marginal R^2 values in bracket ()). For clarity, conditional R^2 values for mediating variables are not shown here but are reported in Table S12. Icons from BioRender [34].

Table S1: Summary of response variables for aboveground specimens on the original scale. Specimens identified aboveground were categorized into five functional groups, with summaries provided for diversity indices, abundance (a), taxon richness (b), and Shannon-Wiener diversity (c). This table can be used to compute transformed values y' for each observation y_i in each plot and sampling season, using the formula: $y' = \frac{y_i - y_{min}}{y_{max} - y_{min}}$.

	(a) Abundance					(b) Taxononic richness					(c) Shannon-Wiener diversity				
Func.group	*Min.*	*Med.*	*Mean*	*Max.*	t.value	*Min.*	*Med.*	*Mean*	*Max.*	t.value	*Min.*	*Med.*	*Mean*	*Max.*	t.value
Total	17	114	151	568	0.37	7	21	22	38	0.92	0.96	2.44	2.35	3.15	1.93
Predators	2	34	46	198	0.40	1	9	9	18	1.22	0.45	1.83	1.78	2.40	1.66
Herbivores	3	32	44	158	0.35	1	6	6	12	0.87	0.00	1.33	1.29	2.00	1.92
Fungivores	0	22	38	187	0.29	0	3	3	6	0.76	0.00	0.65	0.58	1.46	0.96
Omnivores	0	5	9	54	0.30	0	2	2	7	0.76	0.00	0.64	0.58	1.63	0.46
Foliar.fungi	42810	82592	89577	140679	0.89	50	257	256	395	1.15	2.83	3.76	3.68	4.22	0.74
Endo.fungi	34512	94750	96120	189292	0.86	55	132	138	273	1.20	1.50	2.56	2.62	3.37	1.60
Epi.fungi	41	1239	1474	4062	0.85	6	69	77	159	0.53	2.49	3.41	3.34	4.22	0.85

Notes: Diversity indices for foliar fungi were derived from DNA sequence data. Abundance was measured as the total number of sequence reads, and taxonomic richness was estimated using the Chao index. Outliers were identified and removed using the interquartile range (IQR) method. This resulted in the exclusion of one sample for predator abundance (= 278; n =1), five samples for herbivore abundance (≥229; n =5), one sample for fungivore abundance (= 222; n =1), and four samples for omnivore abundance (≥60; n =5). For foliar fungi, outliers in abundance included one sample for total foliar fungi (= 13,113; n =1) and one for epiphytes (= 6,715; n=1). Outliers in Shannon-Wiener diversity included one sample for total foliar fungi (value = 1.71; n =1), one for endophytes (= 0.349; n =1), and one for epiphytes (= 1.55; n =1). No outliers were identified in the remaining datasets.

Table S2: Summary of response variables for soil surface specimens on the original scale. Specimens from the soil surface were categorized into five functional groups. Summaries are provided for abundance (a), taxonomic richness (b), and Shannon-Wiener diversity (c). This table can be used to compute transformed values y' for each observation y_i in each plot and sampling season, using the formula: $y' = \frac{y_i - y_{min}}{y_{max} - y_{min}}$.

	(a) Abundance					(b) Taxonomic richness					(c) Shannon-Wiener diversity				
Func.group	*Min.*	*Median*	*Mean*	*Max.*	t.value	*Min.*	*Median*	*Mean*	*Max.*	t.value	*Min.*	*Median*	*Mean*	*Max.*	t.value
Total	5	64	70	235	0.40	5	14	15	34	0.78	0.81	2.03	1.99	2.95	1.60
Predators	0	7	8	30	0.49	0	4	4	10	0.81	0.00	1.16	1.10	2.16	1.55
Omnivores	0	13	18	77	0.28	0	2	3	6	0.54	0.00	0.50	0.48	1.53	0.93
Herbivores	0	12	25	107	0.30	0	3	3	9	0.65	0.00	0.68	0.72	1.72	1.12
Detritivores	0	7	9	33	0.57	0	3	3	6	0.96	0.00	0.84	0.81	1.56	1.33

Notes: Outliers were identified and removed using the interquartile range (IQR) method. This resulted in the exclusion of four sample for total abundance (values ≥ 271; n = 4), three samples for predator abundance (≥32; n = 3), six sample for detritivores abundance (≥ 43; n =6). One sample of total taxonomic richness (=36; n =1) and one for predators (=12; n =1) were removed. No outliers were identified in the remaining datasets.

Table S3: Summary of response variables for belowground specimens on the original scale. Specimens from the belowground compartment were first categorized into nematodes (Ne), mesofauna (Me), and macrofauna (Ma) based on body size. Within each broad group, specimens were further classified into functional groups based on feeding preference. Summaries of diversity indices, abundance (a), taxonomic richness (b), and Shannon-Wiener diversity (c) are provided below. This table can be used to compute transformed values y' for each observation y_i in each plot and sampling season, using the formula: $y' = \frac{y_i - y_{min}}{y_{max} - y_{min}}$.

Func.group	(a) Abundance					(b) Taxonomic richness					(c) Shannon-Wiener diversity				
(a) Abundance	*Min.*	*Median*	*Mean*	*Max.*	t.value	*Min.*	*Median*	*Mean*	*Max.*	t.value	*Min.*	*Median*	*Mean*	*Max.*	t.value
Ne:Total	4	83	95	334	0.43	1	5	6	16	0.59	0.00	1.55	1.46	2.52	1.21
Ne:Herbivores	0	17	25	116	0.52	0	1	2	6	0.61					0.75
Ne:Bacterivores	0	20	27	120	0.47	0	2	2	6	0.60					0.99
Ne:Fungivores	0	23	36	180	0.50	0	2	2	5	0.76					1.02
Me:Total	26	204	256	980	0.35	7	13	13	20	1.01	0.50	1.74	1.71	2.61	1.24
Me:Fungivores	5	37	58	295	0.31	2	6	6	10	0.89					2.04
Me:Omnivores	4	64	94	547	0.26	1	4	4	6	0.92					0.59
Me:Predators	2	73	97	374	0.31	1	3	3	6	0.78					1.33
Ma:Total					0.38					0.66					0.99
Ma:Predators	0	1	2	9	0.54	0	1	1	4	0.89	0.00	0.00	0.22	1.39	0.70
Ma:Omnivores	0	1	2	11	0.53	0	1	1	4	0.94	0.00	0.00	0.24	1.33	0.90
Earthworms	1	9	13	49	0.55	1	3	3	9	0.87	0.00	0.91	0.85	2.00	1.37
Total.microbes	81	178	179	296	0.89					1.15					0.74
Soil.fungi	30	101	103	188	0.86	191	582	604	1165	1.20	3.34	4.53	4.46	5.15	1.60
Soil.bacteria	18	78	76	149	0.85	488	1236	1271	2462	0.53	5.68	6.26	6.27	6.76	0.85

Note: Shannon-Wiener diversity for each functional group of nematode and mesofauna was excluded due to excessive zeros. Soil fungal and bacterial biomass served as proxies for abundance. For soil microbes, taxonomic richness (Chao) and Shannon-Wiener diversity were derived from DNA sequence data. Total macrofauna (*Ma:Total)* combines macrofauna extracted by heat and mustard methods, using the average of min-max transformed values. Microbial richness and diversity represent the average of min-max transformed values for soil fungi and bacteria; thus, no separate summaries are shown. Outliers were removed, but only found in abundance data, including: total nematodes (≥414, *n*=3), nematode herbivores (≥142, *n*=2), bacterivores (≥146, *n*=5), fungivores (=280, *n*=1); total mesofauna (≥1069, *n*=4), mesofauna fungivores (≥228, *n*=3), omnivores (≥628, *n*=4), predators (=583, *n*=1); macrofauna predators (≥10, *n*=3), omnivores (≥21, *n*=4), earthworms (=25, *n*=1); and total soil microbial (≥362, *n*=1) and fungal biomass (≥262, *n*=1).

Table S4: Summary of mixed-effects model testing the effects of sampling season (Month), tree species richness (TSR; 1, 2, 4 species) and mycorrhizal type (Mycor; AM, EcM, AM+EcM), and their interactions on the diversity indices of aboveground invertebrates. Three diversity indices included (a) abundance, (b) taxononic richness, and (c) Shannon-Wiener diversity index.

Predictors	*Total*	*Predators*	*Herbivores*	*Fungivores*	*Omnivores*
(A) Abundance					
Month	**$F_{1,59}$ = 312.4*** **	**$F_{1,59}$ = 472.7*** **	**$F_{1,55}$ = 155.3*** **	**$F_{1,58}$ = 89.2*** **	**$F_{1,58}$ = 94.9*** **
Mycor	$F_{2,54}$ = 1.5	**$F_{2,54}$ = 3.8* **	$F_{2,54}$ = 0.9	$F_{2,54}$ = 1.4	$F_{2,53}$ = 0.0
Log(TSR)	$F_{1,54}$ = 0.5	$F_{1,54}$ = 0.3	$F_{1,54}$ = 2.6	$F_{1,54}$ = 1.4	$F_{1,53}$ = 0.1
Mycor×Log(TSR)	$F_{2,54}$ = 0.4	$F_{2,54}$ = 1.1	*$F_{2,54}$ = 2.5*	$F_{2,54}$ = 0.1	$F_{2,53}$ = 0.3
R2m(R2c)	0.60(0.80)	0.73(0.85)	0.52(0.68)	0.38(0.52)	0.29(0.54)
(B) Taxonomic richness					
Month	**$F_{1,59}$ = 279.7*** **	**$F_{1,59}$ = 213.3*** **	**$F_{1,59}$ = 119.3*** **	**$F_{1,59}$ = 82.8*** **	**$F_{1,59}$ = 101.2*** **
Mycor	*$F_{2,54}$ = 2.4*	**$F_{2,54}$ = 4.0* **	$F_{2,54}$ = 1.3	$F_{2,54}$ = 0.6	$F_{2,54}$ = 0.1
Log(TSR)	*$F_{1,54}$ = 3.8*	$F_{1,54}$ = 2.4	$F_{1,54}$ = 1.6	$F_{1,54}$ = 0.7	$F_{1,54}$ = 1.4
Mycor×Log(TSR)	$F_{2,54}$ = 0.2	$F_{2,54}$ = 0.2	$F_{2,54}$ = 0.7	$F_{2,54}$ = 0.5	$F_{2,54}$ = 0.0
R2m(R2c)	0.63(0.82)	0.59(0.76)	0.46(0.67)	0.35(0.35)	0.36(0.49)
(C) Shannon-Wiener diversity					
Month	**$F_{1,59}$ = 58.9*** **	**$F_{1,58}$ = 62.5*** **	**$F_{1,59}$ = 32.4*** **	**$F_{1,59}$ = 24.9*** **	**$F_{1,59}$ = 87.8*** **
Mycor	$F_{2,54}$ = 1.6	$F_{2,54}$ = 2.3	**$F_{2,54}$ = 5.4** **	$F_{2,54}$ = 0.1	$F_{2,54}$ = 0.4
Log(TSR)	**$F_{1,54}$ = 14.6*** **	*$F_{1,54}$ = 3.2*	$F_{1,54}$ = 1.8	**$F_{1,54}$ = 4.6* **	$F_{1,54}$ = 0.8
Mycor×Log(TSR)	$F_{2,54}$ = 0.2	$F_{2,54}$ = 0.1	$F_{2,54}$ = 0.1	$F_{2,54}$ = 0.8	$F_{2,54}$ = 0.1
R2m(R2c)	0.38(0.70)	0.32(0.39)	0.24(0.47)	0.20(0.30)	0.37(0.48)

Note: Statistical significance is indicated by asterisks: *$p < 0.05$, **$p < 0.01$, ***$p < 0.001$. Marginal significance ($0.05 < p \leq 0.1$) is in italic. For each parameter of each trophic group (y_i), min–max transformed data were further power-transformed using the t-values reported in Table S1. Models were then fitted as linear mixed-effects models: *lme*(y_i^t ~ Month + Mycor × log(Richness), random = ~1 | composition/Month), using Type I sums of squares. Outlier removal was conducted separately for each biodiversity index and functional group, resulting in different final numbers of observations among models. Therefore, the numerator and denominator degrees of freedom ($F_{numDF,\ denDF}$) are reported specifically for each response variable and parameter.

Table S5: Summary of mixed-effects model testing the effects of tree species richness (TSR; 1, 2, 4 species) and mycorrhizal type (Mycor; AM, EcM, AM+EcM), and their interaction on the diversity indices of foliage fungi. Three diversity indices included (a) abundance, (b) taxonomic richness, and (c) Shannon-Wiener diversity index, which derived from DNA sequence data.

Predictors	*Phyllosphere*	*Endophytes*	*Epiphytes*
(A) Abundance			
Mycor	$F_{2,22} = 0.1$	$F_{2,24} = 0.1$	$F_{2,22} = 0.7$
Log(TSR)	$\boldsymbol{F_{1,22} = 4.6}$*****	$F_{1,24} = 0.1$	$F_{1,22} = 0.8$
Mycor×Log(TSR)	$F_{2,22} = 1.2$	$F_{2,24} = 0.9$	$F_{2,22} = 0.1$
R2m(R2c)	0.18(0.18)	0.05(0.05)	0.08(0.29)
(B) Taxonomic richness (Chao1)			
Mycor	$F_{2,22} = 1.1$	$F_{2,24} = 0.8$	$F_{2,22} = 0.3$
Log(TSR)	$F_{1,22} = 1.4$	$F_{1,24} = 0.7$	$F_{1,22} = 0.1$
Mycor×Log(TSR)	$F_{2,22} = 0.4$	$F_{2,24} = 1.2$	$F_{2,22} = 0.2$
R2m(R2c)	0.12(0.12)	0.11(0.11)	0.03(0.03)
(C) Shannon-Wiener diversity			
Mycor	$F_{2,21} = 0.9$	$F_{2,24} = 1.5$	$F_{2,21} = 0.3$
Log(TSR)	$F_{1,21} = 0.0$	$F_{1,24} = 0.8$	$F_{1,21} = 0.1$
Mycor×Log(TSR)	$F_{2,21} = 0.3$	$F_{2,24} = 1.5$	$F_{2,21} = 0.2$
R2m(R2c)	0.07(0.18)	0.16(0.16)	0.04(0.15)

Notes: In total, we sampled 37 plots for foliar fungi. Statistical significance is indicated by asterisks: *p < 0.05, **p < 0.01, ***p < 0.001. Marginal significance (0.05 ≤ p < 0.1) is in italic. For each parameter of each trophic group (y_i), min–max transformed data were further power-transformed using the t-values reported in Table S1. Models were then fitted as linear mixed-effects models: *lme*(y_i^t ~ Mycor × log(Richness), random = ~1 | composition), using Type I sums of squares. The variable “Month” was not included because foliar fungi were assessed only once, in September 2021. Outlier removal was conducted separately for each biodiversity index and functional group, resulting in different final numbers of observations among models. Therefore, the numerator and denominator degrees of freedom ($F_{numDF, denDF}$) are reported specifically for each response variable and parameter.

Table S6: Summary of mixed-effects model testing the effects of sampling season (Month), tree species richness (TSR; 1, 2, 4 species) and mycorrhizal type (Mycor; AM, EcM, AM+EcM), and their interaction on diversity indices of surface invertebrates. Three diversity indices included (a) abundance, (b) taxonomic richness, and (c) Shannon-Wiener diversity index.

Predictors	*Total*	*Predators*	*Omnivores*	*Herbivores*	*Detritivores*
(A) Abundance					
Month	$\mathbf{F_{1,59} = 9.6{*}{*}}$	$\mathbf{F_{1,59} = 44.7{*}{*}{*}}$	$F_{1,59} = 0.3$	$F_{1,59} = 0.6$	$F_{1,59} = 0.1$
Mycor	$F_{2,54} = 1.0$	$F_{2,54} = 0.8$	$\mathbf{F_{2,54} = 5.6{*}{*}}$	$F_{2,54} = 0.8$	$F_{2,54} = 0.1$
Log(TSR)	$F_{1,54} = 0.0$	$F_{1,54} = 0.6$	$F_{1,54} = 2.6$	$F_{1,54} = 0.2$	$F_{1,54} = 1.5$
Mycor×Log(TSR)	$F_{2,54} = 0.5$	$F_{2,54} = 0.7$	$F_{2,54} = 0.6$	$F_{2,54} = 1.7$	$F_{2,54} = 0.1$
R2m(R2c)	0.09(0.60)	0.24(0.34)	0.09(0.22)	0.04(0.19)	0.01(0.19)
(B) Taxonomic richness					
Month	$\mathbf{F_{1,59} = 14.6{*}{*}{*}}$	$\mathbf{F_{1,59} = 45.1{*}{*}{*}}$	$F_{1,59} = 0.1$	$F_{1,59} = 0.4$	$F_{1,59} = 0.0$
Mycor	$F_{2,54} = 0.9$	$F_{2,54} = 1.0$	$\mathbf{F_{2,54} = 5.1{*}{*}}$	$F_{2,54} = 0.6$	$F_{2,54} = 0.0$
Log(TSR)	$F_{1,54} = 0.0$	$F_{1,54} = 0.1$	$F_{1,54} = 1.8$	$F_{1,54} = 0.0$	$F_{1,54} = 1.7$
Mycor×Log(TSR)	$F_{2,54} = 0.7$	$F_{2,54} = 0.5$	$F_{2,54} = 0.5$	$F_{2,54} = 1.5$	$F_{2,54} = 0.1$
R2m(R2c)	0.12(0.62)	0.26(0.48)	0.08(0.22)	0.03(0.26)	0.01(0.22)
(C) Shannon-Wiener diversity					
Month	$\mathbf{F_{1,59} = 16.8{*}{*}{*}}$	$\mathbf{F_{1,59} = 35.2{*}{*}{*}}$	$F_{1,59} = 0.0$	$F_{1,59} = 0.3$	$F_{1,59} = 0.2$
Mycor	$F_{2,54} = 1.1$	$F_{2,54} = 1.1$	$\mathbf{F_{2,54} = 4.1{*}}$	$F_{2,54} = 0.6$	$F_{2,54} = 0.1$
Log(TSR)	$F_{1,54} = 0.0$	$F_{1,54} = 0.2$	$F_{1,54} = 1.1$	$F_{1,54} = 0.0$	$F_{1,54} = 1.6$
Mycor×Log(TSR)	$F_{2,54} = 0.7$	$F_{2,54} = 0.4$	$F_{2,54} = 0.5$	$F_{2,54} = 1.4$	$F_{2,54} = 0.1$
R2m(R2c)	0.14(0.77)	0.23(0.71)	0.07(0.19)	0.03(0.26)	0.01(0.24)

Note: Statistical significance is indicated by asterisks: $^*p < 0.05$, $^{**}p < 0.01$, $^{***}p < 0.001$. Marginal significance ($0.05 \le p < 0.1$) is in italic. For each parameter of each trophic group (y_i), min–max transformed data were further power-transformed using the t-values reported in Table S2. Models were then fitted as linear mixed-effects models: *lme*(y_i^t ~ Month + Mycor × log(Richness), random = ~1 | composition/Month), using Type I sums of squares. Outlier removal was conducted separately for each biodiversity index and functional group, resulting in different final numbers of observations among models. Therefore, the numerator and denominator degrees of freedom ($F_{numDF, denDF}$) are reported specifically for each response variable and parameter.

Table S7: Summary of mixed-effects model testing the effects of sampling season (Month), tree species richness (TSR; 1, 2, 4 species) and mycorrhizal type (Mycor; AM, EcM, AM+EcM), and their interaction on diversity indices of belowground invertebrates. Three diversity indices included (a) abundance, (b) taxonomic richness, and (c) Shannon-Wiener diversity index.

	Nematode				*Mesofauna*				*Macrofauna*			
Predictors	*Total*	*Herbi-*	*Bacteri-*	*Fungi-*	*Total*	*Fungi-*	*Omni-*	*Pred-*	*Total*	*Pred-*	*Omni-*	*Earthworms*
(A) Abundance												
Month	**$F_{1,56}$ = 23.3*** **	**$F_{1,58}$ = 7.7** **	$F_{1,54}$ = 1.0	**$F_{1,58}$ = 52.8*** **	**$F_{1,56}$ = 5.1* **	**$F_{1,57}$ = 62.7*** **	**$F_{1,57}$ = 10.0** **	**$F_{1,58}$ = 21.6*** **	**$F_{1,58}$ = 22.0*** **	**$F_{1,58}$ = 4.8* **	$F_{1,58}$ = 2.2	**$F_{1,59}$ = 41.0*** **
Mycor	**$F_{2,54}$ = 3.5* **	**$F_{2,54}$ = 8.9*** **	$F_{2,54}$ = 1.8	$F_{2,54}$ = 0.3	$F_{2,54}$ = 0.1	$F_{2,54}$ = 0.2	**$F_{2,54}$ = 3.4* **	**$F_{2,54}$ = 3.5* **	$F_{2,54}$ = 2.0	$F_{2,54}$ = 1.0	$F_{2,54}$ = 0.3	*$F_{2,54}$ = 2.8*
Log(TSR)	**$F_{1,54}$ = 6.3* **	**$F_{1,54}$ = 7.9** **	$F_{1,54}$ = 1.7	**$F_{1,54}$ = 5.6* **	*$F_{1,54}$ = 3.0*	$F_{1,54}$ = 0.3	$F_{1,54}$ = 0.7	**$F_{1,54}$ = 4.9* **	$F_{1,54}$ = 2.1	**$F_{1,54}$ = 11.6** **	$F_{1,54}$ = 0.0	$F_{1,54}$ = 0.1
Mycor×Log(TSR)	$F_{2,54}$ = 1.9	**$F_{2,54}$ = 3.5* **	$F_{2,54}$ = 1.1	$F_{2,54}$ = 0.9	$F_{2,54}$ = 0.1	$F_{2,54}$ = 0.5	$F_{2,54}$ = 0.3	$F_{2,54}$ = 0.2	$F_{2,54}$ = 1.7	**$F_{2,54}$ = 3.4* **	$F_{2,54}$ = 0.3	$F_{2,54}$ = 0.8
R2m(R2c)	0.21(0.21)	0.22(0.26)	0.06(0.18)	0.28(0.28)	0.06(0.30)	0.25(0.41)	0.11(0.21)	0.18(0.20)	0.17(0.31)	0.17(0.50)	0.02(0.18)	0.21(0.37)
(B) Taxonomic richness												
Month	*$F_{1,59}$ = 3.5*	$F_{1,57}$ = 2.3	**$F_{1,59}$ = 7.0* **	**$F_{1,59}$ = 20.9*** **	**$F_{1,59}$ = 34.9*** **	**$F_{1,59}$ = 50.9*** **	$F_{1,59}$ = 0.0	**$F_{1,59}$ = 10.8** **	**$F_{1,58}$ = 23.1*** **	**$F_{1,59}$ = 9.3** **	*$F_{1,59}$ = 2.9*	**$F_{1,58}$ = 23.8*** **
Mycor	*$F_{2,54}$ = 2.6*	**$F_{2,54}$ = 4.2* **	*$F_{2,54}$ = 2.5*	$F_{2,54}$ = 0.4	$F_{2,54}$ = 0.9	$F_{2,54}$ = 2.3	$F_{2,54}$ = 1.3	$F_{2,54}$ = 0.8	$F_{2,54}$ = 0.4	$F_{2,54}$ = 1.2	$F_{2,54}$ = 0.5	$F_{2,54}$ = 0.1
Log(TSR)	**$F_{1,54}$ = 8.2** **	**$F_{1,54}$ = 11.2** **	*$F_{1,54}$ = 3.8*	**$F_{1,54}$ = 4.2* **	$F_{1,54}$ = 0.1	$F_{1,54}$ = 0.4	$F_{1,54}$ = 0.0	$F_{1,54}$ = 0.2	$F_{1,54}$ = 0.5	**$F_{1,54}$ = 10.2** **	$F_{1,54}$ = 0.0	$F_{1,54}$ = 0.1
Mycor×Log(TSR)	$F_{2,54}$ = 1.7	$F_{2,54}$ = 2.4	$F_{2,54}$ = 0.4	$F_{2,54}$ = 0.2	$F_{2,54}$ = 0.5	$F_{2,54}$ = 0.4	$F_{2,54}$ = 0.3	$F_{2,54}$ = 1.5	**$F_{2,54}$ = 3.3* **	**$F_{2,54}$ = 4.1* **	$F_{2,54}$ = 1.5	$F_{2,54}$ = 0.9
R2m(R2c)	0.13(0.19)	0.17(0.25)	0.11(0.34)	0.14(0.15)	0.16(0.40)	0.24(0.44)	0.03(0.21)	0.10(0.39)	0.16(0.39)	0.20(0.49)	0.04(0.17)	0.13(0.23)

Continued table S7

(C) Shannon-Wiener diversity	*Nematodes*	*Mesofauna*	*Macrofauna*
Month	$F_{1,59}$ = 1.7	**$F_{1,59}$ = 41.4*** **	**$F_{1,59}$ = 11.4** **
Mycor	*$F_{2,54}$ = 3.0*	**$F_{2,54}$ = 4.6* **	$F_{2,54}$ = 0.6
Log(TSR)	**$F_{1,54}$ = 7.2** **	$F_{1,54}$ = 0.2	$F_{1,54}$ = 0.7
Mycor×Log(TSR)	$F_{2,54}$ = 1.4	$F_{2,54}$ = 0.3	**$F_{2,54}$ = 3.2* **
R2m(R2c)	0.13(0.24)	0.24(0.31)	0.13(0.40)

Herbi- denotes herbivores, Bacteri- denotes bacterivores, Fungi- denotes fungivores, Omni- denotes omnivores, and Pred- denotes predators.

Note: Statistical significance is indicated by asterisks: *$p < 0.05$, **$p < 0.01$, ***$p < 0.001$. Marginal significance ($0.05 \leq p < 0.1$) is in italic. For each parameter of each trophic group (y_i), min–max transformed data were further power-transformed using the t-values reported in Table S3. Models were then fitted as linear mixed-effects models: *lme*(y_i^t ~ Month + Mycor × log(Richness), random = ~1 | composition/Month), using Type I sums of squares. Outlier removal was conducted separately for each biodiversity index and functional group, resulting in different final numbers of observations among models. Therefore, the numerator and denominator degrees of freedom ($F_{numDF,\ denDF}$) are reported specifically for each response variable and parameter.

Table S8: Summary of mixed-effects model testing the effects of sampling season (Month), tree species richness (TSR; 1, 2, 4 species) and mycorrhizal type (Mycor; AM, EcM, AM+EcM), and their interaction on diversity indices of soil microbes

Predictors	*Soil microbes*	*Soi fungi*	*Soil bacteria*
(A) Abundance (biomass)			
Month	**$F_{1,58}$ = 4.8***	$F_{1,58}$ = 0.0	**$F_{1,59}$ = 5.7***
Mycor	**$F_{2,54}$ = 5.5****	$F_{2,54}$ = 1.5	*$F_{2,54}$ = 3.0*
Log(TSR)	**$F_{1,54}$ = 7.5****	**$F_{1,54}$ = 8.5****	$F_{1,54}$ = 0.1
Mycor×Log(TSR)	$F_{2,54}$ = 0.9	$F_{2,54}$ = 1.1	$F_{2,54}$ = 0.1
R2m(R2c)	0.19(0.38)	0.12(0.42)	0.08(0.30)
(B) Taxonomic richness (Chao1)			
Mycor	**$F_{2,54}$ = 6.3****	**$F_{2,54}$ = 14.8*****	$F_{2,54}$ = 1.1
Log(TSR)	**$F_{1,54}$ = 82.8*****	**$F_{1,54}$ = 79.2*****	**$F_{1,54}$ = 59.8*****
Mycor×Log(TSR)	$F_{2,54}$ = 0.9	$F_{2,54}$ = 2.2	$F_{2,54}$ = 0.2
R2m(R2c)	0.61(0.76)	0.68(0.90)	0.46(0.50)
(C) Shannon-Wiener diversity			
Mycor	**$F_{2,54}$ = 16.4*****	**$F_{2,54}$ = 31.7*****	$F_{2,54}$ = 0.5
Log(TSR)	**$F_{1,54}$ = 43.0*****	**$F_{1,54}$ = 30.5*****	**$F_{1,54}$ = 13.9*****
Mycor×Log(TSR)	*$F_{2,54}$ = 2.5*	$F_{2,54}$ = 1.9	$F_{2,54}$ = 0.1
R2m(R2c)	0.55(0.67)	0.61(0.78)	0.17(0.25)

Note: Statistical significance is indicated by asterisks: *$p < 0.05$, **$p < 0.01$, ***$p < 0.001$. Marginal significance ($0.05 \leq p < 0.1$) is in italic. For microbial, fungal and bacterial abundance (y_i), min–max transformed data were further power-transformed using the t-values reported in Table S3. Models were then fitted as linear mixed-effects models: *lme*(y_i^t ~ Month + Mycor × log(Richness), random = ~1 | composition/Month), using Type I sums of squares. For taxonomic richness and Shannon diversity, min–max transformed data were power-transformed using the t-values in Table S3, and models were fitted as *lme*(y_i^t ~ Mycor × log(Richness), random = ~1 | composition). “Month” was excluded because soil fungal and microbial diversity were measured only once (September 2021). Outlier removal was conducted separately for each biodiversity index and functional group, resulting in different final numbers of observations among models. Therefore, the numerator and denominator degrees of freedom ($F_{numDF,\ denDF}$) are reported specifically for each response variable and parameter.

Table S9: Summary of mixed-effects model analyses testing the effects of tree species richness (TSR; 1, 2, 4 species) and mycorrhizal types (Mycor; AM, EcM, AM+EcM) on potential mediators. The potential mediators included (a) aboveground tree stand conditions, (b) adsorptive root carbon and nutrients, (c) transportation root carbon and nutrients and (d) soil properties.

(A) Aboveground tree stand characteristics

predictors	*Df*	*denDF*	*Leaf C*	*Leaf N*	*Leaf C:N*	*Wood volumes*	*ENL*
Mycor	2	54	**21.39*****	**22.92*****	**14.40*****	0.37	0.65
Log(TSR)	1	54	0.26	**9.52****	**6.61***	**7.70****	**7.03***
Mycor:Log(TSR)	2	54	0.24	0.19	0.53	0.45	0.36
R2m(R2c)			0.42(0.71)	0.46(0.64)	0.37(0.61)	0.14(0.61)	0.14(0.89)

(B) Absorptive root nutrient concentration

Predictors	*Df*	*denDF*	*C*	*N*	*C:N*	*Mg*	*Ca*
Mycor	2	54	1.25	0.47	0.97	**3.99***	**6.40****
Log(TSR)	1	54	0.03	0.01	0.13	2.19	0.56
Mycor:Log(TSR)	2	54	**6.90****	0.42	1.39	1.82	0.47
$R^2m(R^2c)$			0.17(0.22)	0.02(0.02)	0.06(0.06)	0.15(0.20)	0.15(0.15)

(C) Transportation root nutrient concentration

Predictors	*Df*	*denDF*	*C*	*N*	*C:N*	*Mg*	*Ca*
Mycor	2	54	0.26	1.94	*2.92*	0.35	**3.21***
Log(TSR)	1	54	0.01	0.35	0.86	0.00	2.68
Mycor:Log(TSR)	2	54	0.46	1.56	1.35	0.30	0.80
$R^2m(R^2c)$			0.02(0.02)	0.09(0.09)	0.11(0.11)	0.02(0.02)	0.12(0.12)

(D) Soil properties

Predictors	*Df*	*denDF*	*pH*	*TC*	*TN*	*C:N*	NO_3^-	PO_4^-
Mycor	2	54	0.37	**3.56***	*2.79*	0.03	**4.00***	0.39
Log(TSR)	1	54	0.01	1.03	1.31	0.03	**7.12***	1.78
Mycor:Log(TSR)	2	54	0.06	1.31	0.35	0.67	1.30	0.11
$R^2m(R^2c)$			0.01(0.62)	0.13(0.35)	0.09(0.09)	0.02(0.26)	0.22(0.53)	0.04(0.28)

Statistical significance is indicated by asterisks: *p < 0.05, **p < 0.01, ***p < 0.001. Marginal significance (0.05 ≤ p < 0.1) is in italic.

Table S10: Pearson's correlations among mediators significantly influenced by the treatment factors (tree species richness and/or mycorrhizal type).

Variables	*Wood volume*	*ENL*	*Leaf C*	*Leaf N*	*Leaf C:N*	*Root C*	*Root Mg*	*Root Ca*	*Soil TC*	*Soil TN*
ENL	**0.73*** **									
Leaf C	**-0.29** **	-0.06								
Leaf N	-0.17	-0.16	**0.52*** **							
Leaf C:N	0.12	0.15	**-0.38*** **	**-0.97*** **						
Root C	0.14	*0.2*	-0.1	-0.14	0.08					
Root Mg	0.03	0.08	**-0.24* **	-0.18	0.12	-0.1				
Root Ca	0.07	0.05	**0.22* **	*0.19*	-0.14	-0.11	**-0.62*** **			
Soil TC	0.09	0	-0.15	-0.13	0.09	-0.06	**0.29** **	-0.01		
Soil TN	0.16	0.11	-0.1	-0.03	0	-0.04	**0.3** **	0.01	**0.74*** **	
Soil NO_3^-	**-0.28* **	*-0.21*	**0.28* **	**0.41*** **	**-0.35** **	-0.04	-0.1	*0.2*	**-0.31** **	-0.14

Note: Statistical significance is indicated by asterisks: *p < 0.05, **p < 0.01, ***p < 0.001. Marginal significance (0.05 ≤ p < 0.1) is in italic.

Table S11 A: The structural equation model for multitrophic abundance of invertebrates, with min-max transformation

Response	*Predictor*	*DF*	*Std.Estimate*	*Std.Error*	*p-value*
Canopy organisms	Leaf C:N	18	**-0.257***	0.005	0.042
	ENL	18	0.026	0.0052	0.830
Surface organisms	ENL	17	-0.193	0.0055	0.133
	Leaf C:N	17	-0.172	0.0052	0.163
	Root Ca	17	0.129	0.0268	0.236
Soil organisms	ENL	17	0.105	0.0025	0.347
	Leaf C:N	17	**0.319****	0.0024	0.008
	Root Ca	17	**0.345****	0.0133	0.003
	Log(TSR)	58	0.170	0.0166	0.144
ENL	Log(TSR)	57	**0.397****	0.8434	0.005
	EcM%	57	-0.051	0.9918	0.694
Leaf C:N	Log(TSR)	57	**0.285****	0.6425	0.007
	EcM%	57	**-0.537*****	0.7809	<0.001
Root Ca	Log(TSR)	77	-0.084	0.1136	0.424
	EcM%	77	**0.380*****	0.1419	<0.001
Root C	Log(TSR)	56	-0.272	0.0273	0.116
	EcM%	56	**-0.455***	0.0361	0.015
	Log(TSR)	56	**0.450***	0.0406	0.047
Soil NO3-	Log(TSR)	57	**-0.379****	0.0873	0.003
	EcM%	57	*0.208*	0.1055	0.079
~~Canopy organisms	~~Surface organisms	80	**0.254***	-	0.012
~~Canopy organisms	~~Soil organisms	80	*0.153*	-	0.089
~~Soil organisms	~~Surface organisms	80	0.069	-	0.273
~~Leaf C:N	~~Soil NO3-	80	**-0.272****	-	0.008
~~Root C	~~ENL	80	0.016	-	0.445

Note: Statistical significance (in bold) is indicated by asterisks: *p < 0.05, **p < 0.01, ***p < 0.001. Marginal significance ($0.05 \le p < 0.1$) is in italic. EcM% represents to the proportion of EcM tree species in each plot. The model fit was good (Fisher's C = 34.249, df

= 40, p = 0.726). Likelihood-based χ^2 statistics could not be calculated because the model structures (combined both simple linear regression and mixed effect models).

Response	*family*	*Marginal Rm2*	*Conditional R2c*
Canopy organisms	gaussian	0.06	0.40
Surface organisms	gaussian	0.09	0.58
Soil organisms	gaussian	0.27	0.36
ENL	gaussian	0.15	0.88
Leaf C:N	gaussian	0.37	0.59
Root Ca	gaussian	0.15	NA
Root C	gaussian	0.08	0.16
Soil NO3-	gaussian	0.18	0.54

Table S11 B: The structural equation model for multitrophic diversity of invertebrates, with min-max transformation

Response	*Predictor*	*DF*	*Std.Estimate*	*Std.Error*	*p-value*
Canopy organisms	Leaf C:N	19	**-0.306***	0.0041	0.014
	Log(TSR)	58	**0.297***	0.0273	0.016
Surface organisms	ENL	17	*-0.252*	0.0055	0.057
	Leaf C:N	17	0.003	0.005	0.982
	Root Ca	17	0.117	0.025	0.252
Soil organisms	ENL	17	0.125	0.0029	0.267
	Leaf C:N	17	**0.301***	0.0028	0.012
	Root Ca	17	**0.333****	0.0155	0.005
ENL	Log(TSR)	57	**0.397****	0.8434	0.005
	EcM%	57	-0.051	0.9918	0.694
Leaf C:N	Log(TSR)	57	**0.285****	0.6425	0.007
	EcM%	57	**-0.537*****	0.7809	<0.001
Root Ca	Log(TSR)	77	-0.084	0.1136	0.424
	EcM%	77	**0.380*****	0.1419	<0.001
Root C	Log(TSR)	56	-0.272	0.0273	0.116
	EcM%	56	**-0.455***	0.0361	0.015
	Log(TSR)	56	**0.450***	0.0406	0.047
Soil NO3-	Log(TSR)	57	**-0.379****	0.0873	0.003
	EcM%	57	*0.208*	0.1055	0.079
~~Canopy organisms	~~Surface organisms	80	**0.411*****	-	<0.001
~~Canopy organisms	~~Soil organisms	80	-0.011	-	0.460
~~Soil organisms	~~Surface organisms	80	**-0.206***	-	0.034
~~Leaf C:N	~~Soil NO3-	80	**-0.272****	-	0.008
~~Root C	~~ENL	80	0.016	-	0.445

Note: Statistical significance (in bold) is indicated by asterisks: *$p < 0.05$, **$p < 0.01$, ***$p < 0.001$. Marginal significance ($0.05 \leq p < 0.1$) is in italic. EcM% represents to the proportion of EcM tree species in each plot. The model fit was good (Fisher's C = 33.551, df = 42, p-value = 0.821). Likelihood-based χ^2 statistics could not be calculated because the model structures (combined both simple linear regression and mixed effect models).

Response	*family*	*Marginal Rm2*	*Conditional R2c*
Canopy organisms	gaussian	0.13	0.32
Surface organisms	gaussian	0.07	0.69
Soil organisms	gaussian	0.20	0.42
ENL	gaussian	0.15	0.88
Leaf C:N	gaussian	0.37	0.59
Root Ca	gaussian	0.15	NA
Root C	gaussian	0.08	0.16
Soil NO3-	gaussian	0.18	0.54

Table S12 A: The structural equation model for multitrophic abundance of invertebrates, with z-score transformation

Response	*Predictor*	*DF*	*Std.Estimate*	*Std.Error*	*p-values*
Canopy organisms	Leaf C:N	18	**-0.26***	0.02	0.042
	ENL	18	0.03	0.02	0.837
Surface organisms	ENL	17	-0.19	0.03	0.134
	Leaf C:N	17	-0.13	0.03	0.295
	Root Ca	17	0.14	0.15	0.222
Soil organisms	ENL	17	0.12	0.01	0.274
	Leaf C:N	17	**0.27***	0.01	0.02
	Root Ca	17	**0.35****	0.06	0.003
	Log(TSR)	58	0.15	0.07	0.178
ENL	Log(TSR)	57	**0.4****	0.84	0.005
	EcM%	57	-0.05	0.99	0.694
Leaf C:N	Log(TSR)	57	**0.28****	0.64	0.007
	EcM%	57	**-0.54*****	0.78	<0.001
Root Ca	Log(TSR)	77	-0.08	0.11	0.424
	EcM%	77	**0.38*****	0.14	<0.001
Root C	Log(TSR)	56	-0.27	0.03	0.116
	EcM%	56	**-0.45***	0.04	0.015
	Log(TSR)	56	**0.45***	0.04	0.047
Soil NO_3-	Log(TSR)	57	**-0.38****	0.09	0.003
	EcM%	57	*0.21*	0.11	0.079
~~Canopy organisms	~~Surface organisms	80	**0.29****		0.005
~~Canopy organisms	~~Soil organisms	80	*0.17*		0.066
~~Soil organisms	~~Surface organisms	80	-0.02		0.42
~~Leaf C:N	~~Soil NO_3-	80	**-0.27****		0.008
~~Root C	~~ENL	80	0.02		0.445

Note: Statistical significance (in bold) is indicated by asterisks: *$p < 0.05$, **$p < 0.01$, ***$p < 0.001$. Marginal significance ($0.05 \leq p < 0.1$) is in italic. EcM% represents to the proportion of EcM tree species in each plot. The model fit was good (Fisher's C = 26.232, df

= 40, P = 0.954). Likelihood-based χ^2 statistics could not be calculated because the model structures (combined both simple linear regression and mixed effect models).

Response	*family*	*Marginal Rm2*	*Conditional R2c*
Canopy organisms	gaussian	0.06	0.43
Surface organisms	gaussian	0.08	0.41
Soil organisms	gaussian	0.24	0.26
ENL	gaussian	0.15	0.88
Leaf C:N	gaussian	0.37	0.59
Root Ca	gaussian	0.15	NA
Root C	gaussian	0.08	0.16
Soil NO_3-	gaussian	0.18	0.54

Table S12 B: The structural equation model for multitrophic diversity of invertebrates, with z-score transformation

Response	*Predictor*	*DF*	*Std.Estimate*	*Std.Error*	*p-values*
Canopy organisms	Leaf C:N	19	**-0.31***	0.02	0.014
	Log(TSR)	58	**0.29***	0.15	0.019
Surface organisms	ENL	18	*-0.23*	0.03	0.078
	Root Ca	18	0.1	0.12	0.315
Soil organisms	ENL	17	0.18	0.01	0.108
	Leaf C:N	17	**0.27***	0.01	0.024
	Root Ca	17	**0.35****	0.06	0.003
ENL	Log(TSR)	57	**0.4****	0.84	0.005
	EcM%	57	-0.05	0.99	0.694
Leaf C:N	Log(TSR)	57	**0.28****	0.64	0.007
	EcM%	57	**-0.54*****	0.78	<0.001
Root Ca	Log(TSR)	77	-0.08	0.11	0.424
	EcM%	77	**0.38*****	0.14	<0.001
Root C	Log(TSR)	56	-0.27	0.03	0.116
	EcM%	56	**-0.45***	0.04	0.015
	Log(TSR)	56	**0.45***	0.04	0.047
Soil NO_3-	Log(TSR)	57	**-0.38****	0.09	0.003
	EcM%	57	*0.21*	0.11	0.079
~~Canopy organisms	~~Surface organisms	80	**0.41*****		<0.001
~~Canopy organisms	~~Soil organisms	80	-0.03		0.409
~~Soil organisms	~~Surface organisms	80	**-0.24***		0.015
~~Leaf C:N	~~Soil NO_3-	80	**-0.27****		0.008
~~Root C	~~ENL	80	0.02		0.445

Note: Statistical significance (in bold) is indicated by asterisks: *$p < 0.05$, **$p < 0.01$, ***$p < 0.001$. Marginal significance ($0.05 < p \leq 0.1$) is in italic. EcM% represents to the proportion of EcM tree species in each plot. The model fit was good (Fisher's C = 34.818, df = 44, p-value = 0.837). Likelihood-based χ^2 statistics could not be calculated because the model structures (combined both simple linear regression and mixed effect models).

Response	*family*	*Marginal Rm2*	*Conditional R2c*
Canopy organisms	gaussian	0.12	0.34
Surface organisms	gaussian	0.06	0.65
Soil organisms	gaussian	0.21	0.47
ENL	gaussian	0.15	0.88
Leaf C:N	gaussian	0.37	0.59
Root Ca	gaussian	0.15	NA
Root C	gaussian	0.08	0.16
Soil NO_3-	gaussian	0.18	0.54

Table S13 A: The structural equation model for multitrophic abundance of invertebrates (min-max transformation), replacing ENL with tree wood volume as a mediator

Response	*Predictor*	*DF*	*Std.Estimate*	*Std.Error*	*p-values*
Canopy organisms	Wood volume	18	0.05	0.12	0.66
	Leaf C:N	18	**-0.26***	0.00	0.038
Surface organisms	Wood volume	17	0.03	0.12	0.776
	Leaf C:N	17	-0.2	0.00	0.108
	Root Ca	17	0.11	0.03	0.3
Soil organisms	Wood volume	17	0.04	0.05	0.745
	Leaf C:N	17	**0.31***	0.00	0.01
	Root Ca	17	**0.34****	0.01	0.004
	Log(TSR)	58	0.19	0.01	0.101
Wood volume	Log(TSR)	57	**0.32***	0.03	0.014
	EcM%	57	-0.1	0.04	0.432
Leaf C:N	Log(TSR)	57	**0.28****	0.64	0.007
	EcM%	57	**-0.54*****	0.78	<0.001
Root Ca	Log(TSR)	77	-0.08	0.11	0.424
	EcM%	77	**0.38*****	0.14	<0.001
Root C	Log(TSR)	56	-0.27	0.03	0.116
	EcM%	56	**-0.45***	0.04	0.015
	Log(TSR)	56	**0.45***	0.04	0.047
Soil NO_3-	Log(TSR)	57	**-0.38****	0.09	0.003
	EcM%	57	*0.21*	0.11	0.079
~~Canopy organisms	~~Surface organisms	80	**0.25***		0.014
~~Canopy organisms	~~Soil organisms	80	*0.17*		0.073
~~Soil organisms	~~Surface organisms	80	0.05		0.322
~~Leaf C:N	~~Soil NO_3-	80	**-0.27****		0.008
~~Root C	~~Wood volume	80	0.12		0.139

Note: Statistical significance (in bold) is indicated by asterisks: *$p < 0.05$, **$p < 0.01$, ***$p < 0.001$. Marginal significance ($0.05 \leq p < 0.1$) is in italic. EcM% represents to the proportion

of EcM tree species in each plot. The model fit was good (Fisher's C = 35.715, df = 40, p-value = 0.663). Likelihood-based χ^2 statistics could not be calculated because the model structures (combined both simple linear regression and mixed effect models).

Response	*family*	*Marginal Rm2*	*Conditional R2c*
Canopy organisms	gaussian	0.06	0.42
Surface organisms	gaussian	0.06	0.56
Soil organisms	gaussian	0.24	0.28
Wood volume	gaussian	0.11	0.59
Leaf C:N	gaussian	0.37	0.59
Root Ca	gaussian	0.15	NA
Root C	gaussian	0.08	0.16
Soil NO_3-	gaussian	0.18	0.54

Table S13 B: The structural equation model for multitrophic diversity of invertebrates (min-max transformation), replacing ENL with Wood volume as a mediator

Response	*Predictor*	*DF*	*Std.Estimate*	*Std.Error*	*p-values*
Canopy organisms	Wood volume	18	0.08	0.10	0.475
	Leaf C:N	18	**-0.31***	0.00	0.015
	Log(TSR)	58	**0.27***	0.03	0.036
Surface organisms	Wood volume	17	-0.06	0.12	0.615
	Leaf C:N	17	-0.02	0.00	0.85
	Root Ca	17	0.11	0.03	0.317
Soil organisms	Wood volume	17	0.08	0.06	0.448
	Leaf C:N	17	**0.31***	0.00	0.01
	Root Ca	17	**0.34****	0.02	0.004
Wood volume	Log(TSR)	57	**0.32***	0.03	0.014
	EcM%	57	-0.1	0.04	0.432
Leaf C:N	Log(TSR)	57	**0.28****	0.64	0.007
	EcM%	57	**-0.54*****	0.78	<0.001
Root Ca	Log(TSR)	77	-0.08	0.11	0.424
	EcM%	77	**0.38*****	0.14	<0.001
Root C	Log(TSR)	56	-0.27	0.03	0.116
	EcM%	56	**-0.45***	0.04	0.015
	Log(TSR)	56	**0.45***	0.04	0.047
Soil NO_3-	Log(TSR)	57	**-0.38****	0.09	0.003
	EcM%	57	*0.21*	0.11	0.079
~~Canopy organisms	~~Surface organisms	80	**0.4*****		<0.001
~~Canopy organisms	~~Soil organisms	80	-0.01		0.465
~~Soil organisms	~~Surface organisms	80	**-0.23***		0.02
~~Leaf C:N	~~Soil NO_3-	80	**-0.27****		0.008
~~Root C	~~Wood volume	80	0.12		0.139

Note: Statistical significance (in bold) is indicated by asterisks: *$p < 0.05$, **$p < 0.01$, ***$p < 0.001$. Marginal significance ($0.05 \leq p < 0.1$) is in italic. EcM% represents to the proportion

of EcM tree species in each plot. The model fit was good (Fisher's C = 37.285, df = 40, p-value = 0.593). Likelihood-based χ^2 statistics could not be calculated because the model structures (combined both simple linear regression and mixed effect models).

Response	*family*	*Marginal Rm2*	*Conditional R2c*
Canopy organisms	gaussian	0.13	0.33
Surface organisms	gaussian	0.01	0.63
Soil organisms	gaussian	0.19	0.40
Wood volume	gaussian	0.11	0.59
Leaf C:N	gaussian	0.37	0.59
Root Ca	gaussian	0.15	NA
Root C	gaussian	0.08	0.16
Soil NO_3-	gaussian	0.18	0.54

Table S14 A: The structural equation model for multitrophic abundance of invertebrates (min-max transformation), including tree wood volume as a mediator

Response	*Predictor*	*DF*	*Std.Estimate*	*Std.Error*	*p-values*
Canopy organisms	Wood volume	18	0.05	0.12	0.66
	Leaf C:N	18	**-0.26***	0.00	0.038
Surface organisms	Wood volume	16	*0.31*	0.16	0.069
	ENL	16	**-0.42***	0.01	0.023
	Leaf C:N	16	-0.17	0.00	0.154
	Root Ca	16	0.12	0.03	0.258
Soil organisms	Wood volume	16	-0.03	0.07	0.84
	ENL	16	0.1	0.00	0.531
	Leaf C:N	16	**0.31***	0.00	0.011
	Root Ca	16	**0.34****	0.01	0.005
	Log(TSR)	58	0.17	0.01	0.141
ENL	Log(TSR)	57	**0.4****	0.84	0.005
	EcM%	57	-0.05	0.99	0.694
Wood volume	Log(TSR)	57	**0.32***	0.03	0.014
	EcM%	57	-0.1	0.04	0.432
Leaf C:N	Log(TSR)	57	**0.28****	0.64	0.007
	EcM%	57	**-0.54*****	0.78	<0.001
Root Ca	Log(TSR)	77	-0.08	0.11	0.424
	EcM%	77	**0.38*****	0.14	<0.001
Root C	Log(TSR)	56	-0.27	0.03	0.116
	EcM%	56	**-0.45***	0.04	0.015
	Log(TSR)	56	**0.45***	0.04	0.047
Soil NO_3-	Log(TSR)	57	**-0.38****	0.09	0.003
	EcM%	57	*0.21*	0.11	0.079
~~Canopy organisms	~~Surface organisms	80	**0.22***		0.027
~~Canopy organisms	~~Soil organisms	80	*0.17*		0.069
~~Soil organisms	~~Surface organisms	80	0.07		0.272

~~Leaf C:N	~~Soil NO_3-	80	**-0.27****		0.008
~~Root C	~~Wood volume	80	0.12	NA	0.139
~~ENL	~~Wood volume	80	**0.48*****	NA	<0.001

Note: Statistical significance (in bold) is indicated by asterisks: *$p < 0.05$, **$p < 0.01$, ***$p < 0.001$. Marginal significance ($0.05 \leq p < 0.1$) is in italic. EcM% represents to the proportion of EcM tree species in each plot. The model fit was good (Fisher's C = 45.006, df = 50, p-value = 0.674). Likelihood-based χ^2 statistics could not be calculated because the model structures (combined both simple linear regression and mixed effect models).

Response	*family*	*Marginal Rm2*	*Conditional R2c*
Canopy organisms	gaussian	0.06	0.42
Surface organisms	gaussian	0.13	0.57
Soil organisms	gaussian	0.24	0.29
ENL	gaussian	0.15	0.88
Wood volume	gaussian	0.11	0.59
Leaf C:N	gaussian	0.37	0.59
Root Ca	gaussian	0.15	NA
Root C	gaussian	0.08	0.16
Soil NO_3-	gaussian	0.18	0.54

Table S14 B: The structural equation model for multitrophic diversity of invertebrates (min-max transformation), including tree wood volume as a mediator

Response	*Predictor*	*DF*	*Std.Estimate*	*Std.Error*	*p-values*
Canopy organisms	Wood volume	18	0.08	0.10	0.475
	Leaf C:N	18	**-0.31***	0.00	0.015
	Log(TSR)	58	**0.27***	0.03	0.036
Surface organisms	Wood volume	16	0.17	0.15	0.263
	ENL	16	**-0.38***	0.01	0.034
	Leaf C:N	16	0	0.00	0.979
	Root Ca	16	0.11	0.02	0.262
Soil organisms	Wood volume	16	-0.01	0.09	0.956
	ENL	16	0.13	0.00	0.415
	Leaf C:N	16	**0.3***	0.00	0.014
	Root Ca	16	**0.33****	0.02	0.005
ENL	Log(TSR)	57	**0.4****	0.84	0.005
	EcM%	57	-0.05	0.99	0.694
Wood volume	Log(TSR)	57	**0.32***	0.03	0.014
	EcM%	57	-0.1	0.04	0.432
Leaf C:N	Log(TSR)	57	**0.28****	0.64	0.007
	EcM%	57	**-0.54*****	0.78	<0.001
Root Ca	Log(TSR)	77	-0.08	0.11	0.424
	EcM%	77	**0.38*****	0.14	<0.001
Root C	Log(TSR)	56	-0.27	0.03	0.116
	EcM%	56	**-0.45***	0.04	0.015
	Log(TSR)	56	**0.45***	0.04	0.047
Soil NO_3-	Log(TSR)	57	**-0.38****	0.09	0.003
	EcM%	57	*0.21*	0.11	0.079
~~Canopy organisms	~~Surface organisms	80	**0.4*****		<0.001
~~Canopy organisms	~~Soil organisms	80	-0.01		0.464
~~Soil organisms	~~Surface organisms	80	**-0.21***		0.032

~~Leaf C:N	~~Soil NO_3-	80	**-0.27***	0.008
~~Root C	~~Wood volume	80	0.12	0.139
~~Wood volume	~~ENL	80	**0.48*****	<0.001

Note: Statistical significance (in bold) is indicated by asterisks: *p < 0.05, **p < 0.01, ***p < 0.001. Marginal significance (0.05 ≤ p < 0.1) is in italic. EcM% represents to the proportion of EcM tree species in each plot. The model fit was good (Fisher's C = 44.71, df = 50, p-value = 0.685). Likelihood-based χ^2 statistics could not be calculated because the model structures (combined both simple linear regression and mixed effect models).

Response	*family*	*Marginal Rm2*	*Conditional R2c*
Canopy organisms	gaussian	0.13	0.33
Surface organisms	gaussian	0.08	0.70
Soil organisms	gaussian	0.19	0.41
ENL	gaussian	0.15	0.88
Wood volume	gaussian	0.11	0.59
Leaf C:N	gaussian	0.37	0.59
Root Ca	gaussian	0.15	NA
Root C	gaussian	0.08	0.16
Soil NO_3-	gaussian	0.18	0.54

Table S15: Model structure and performance for detecting mediator effects on biodiversity indices of aboveground organisms. Each biodiversity index for each trophic group was power transformed using the corresponding t-value (power-trans(y, t-value)).

A: Abundance

Fauna group	*t-value*	*Final model structures*
Total	0.66	power-trans(y, 0.66) ~ Leaf C:N , random = ~ 1|composition; R^2m=0.039 (R^2c=0.53)
Herbivores	0.56	power-trans(y, 0.56) ~ 1 , random = ~ 1|composition;
Fungivores	0.55	power-trans(y, 0.55) ~ 1 , random = ~ 1|composition;
Omnivores	0.37	power-trans(y, 0.37) ~ Soil C + Root C + Leaf C:N + ENL , random = ~ 1|composition; R^2m=0.195 (R^2c=0.311)
Predators	0.51	power-trans(y, 0.51) ~ Leaf C:N , random = ~ 1|composition; R^2m=0.043 (R^2c=0.577)
Foliar fungi	0.77	power-trans(y, 0.77) ~ 1 , random = ~ 1|composition;
Endo. fungi	0.68	power-trans(y, 0.68) ~ Soil C, random = ~ 1|composition; R^2m=0.063 (R^2c=0.063)
Epi.fungi	0.72	power-trans(y, 0.72) ~ Leaf C:N + Root Ca, random = ~ 1|composition; R^2m=0.261 (R^2c=0.528)

B: Taxonomic richness

Fauna group	*t-value*	*Final model structures*
Total	0.88	power-trans(y, 0.88) ~ Soil C + Leaf C:N + Soil NO_3^-, random = ~ 1|composition; R^2m=0.152 (R^2c=0.579)
Herbivores	0.85	power-trans(y, 0.85) ~ Soil C , random = ~ 1|composition; R^2m=0.056 (R^2c=0.501)
Fungivores	0.99	power-trans(y, 0.99) ~ Leaf C:N + Soil NO_3^-, random = ~ 1|composition; R^2m=0.065 (R^2c=0.065)
Omnivores	0.76	power-trans(y, 0.76) ~ Soil C , random = ~ 1|composition; R^2m=0.047 (R^2c=0.125)
Predators	0.95	power-trans(y, 0.95) ~ Leaf C:N , random = ~ 1|composition; R^2m=0.073 (R^2c=0.581)
Foliar fungi	1.38	power-trans(y, 1.38) ~ 1 , random = ~ 1|composition;
Endo. fungi	0.80	power-trans(y, 0.8) ~ Root C , random = ~ 1|composition; R^2m=0.063 (R^2c=0.063)
Epi.fungi	0.70	power-trans(y, 0.7) ~ 1 , random = ~ 1|composition;

C: Shannon-Wiener diversity index

Fauna group	*t-value*	*Final model structures*
Total	2.80	power-trans(y, 2.8) ~ Leaf C:N + Soil NO_3^-, random = ~ 1|composition; R^2m=0.099 (R^2c=0.535)
Herbivores	1.70	power-trans(y, 1.7) ~ Root C, random = ~ 1|composition; R^2m=0.026 (R^2c=0.508)
Fungivores	1.45	power-trans(y, 1.45) ~ Leaf C:N + ENL + Soil NO_3^-, random = ~ 1|composition; R^2m=0.194 (R^2c=0.334)
Omnivores	1.21	power-trans(y, 1.21) ~ 1 , random = ~ 1|composition
Predators	1.76	power-trans(y, 1.76) ~ Soil NO_3^-, random = ~ 1|composition; R^2m=0.029 (R^2c=0.276)
Foliar fungi	0.71	power-trans(y, 0.71) ~ Leaf C:N + Root Ca, random = ~ 1|composition; R^2m=0.189 (R^2c=0.189)
Endo. fungi	1.39	power-trans(y, 1.39) ~ Soil C + Root C + ENL, random = ~ 1|composition; R^2m=0.232 (R^2c=0.232)
Epi.fungi	1.63	power-trans(y, 1.63) ~ 1 , random = ~ 1|composition

Table S16: Model structure and performance for detecting mediator effects on biodiversity indices of soil surface organisms. Each biodiversity index for each trophic group was power transformed using the corresponding t-value (power-trans(y, t-value)).

A: Abundance

Fauna group	*t-value*	*Final model structures*
Total	0.41	power-trans(y, 0.41) ~ ENL , random = ~ 1|composition; R^2m=0.032 (R^2c=0.304)
Herbivores	0.43	power-trans(y, 0.43) ~ Leaf C:N , random = ~ 1|composition; R^2m=0.028 (R^2c=0.028)
Detritivores	0.63	power-trans(y, 0.63) ~ ENL + Root Ca, random = ~ 1|composition; R^2m=0.068 (R^2c=0.068)
Omnivores	0.48	power-trans(y, 0.48) ~ ENL, random = ~ 1|composition; R^2m=0.034 (R^2c=0.259)
Predators	0.42	power-trans(y, 0.42) ~ Leaf C:N, random = ~ 1|composition; R^2m=0.052 (R^2c=0.724)

B: Taxonomic richness

Fauna group	*t-value*	*Final model structures*
Total	0.88	power-trans(y, 0.88) ~ ENL, random = ~ 1|composition; R^2m=0.041 (R^2c=0.716)
Herbivores	0.77	power-trans(y, 0.77) ~ 1 , random = ~ 1|composition
Detrivivores	0.86	power-trans(y, 0.86) ~ ENL + Root Ca, random = ~ 1|composition; R^2m=0.102 (R^2c=0.440)
Omnivores	0.79	power-trans(y, 0.79) ~ Leaf C:N, random = ~ 1|composition; R^2m=0.058 (R^2c=0.058)
Predators	0.68	power-trans(y, 0.68) ~ Root C, random = ~ 1|composition; R^2m=0.031 (R^2c=0.679)

C: Shannon-Wiener diversity index

Fauna group	*t-value*	*Final model structures*
Total	1.24	power-trans(y, 1.24) ~ ENL, random = ~ 1|composition; R^2m=0.03 (R^2c=0.307)
Herbivores	1.32	power-trans(y, 1.32) ~ 1 , random = ~ 1|composition
Detrivivores	1.24	power-trans(y, 1.24) ~ ENL + Root Ca, random = ~ 1|composition; R^2m=0.11 (R^2c=0.437)
Omnivores	0.67	power-trans(y, 0.67) ~ Root C + Leaf C:N, random = ~ 1|composition; R^2m=0.101 (R^2c=0.101)
Predators	1.51	power-trans(y, 1.51) ~ ENL, random = ~ 1|composition; R^2m=0.044 (R^2c=0.501)

Table S17: Model structure and performance for detecting mediator effects on biodiversity indices of belowground organisms. Each biodiversity index for each trophic group was power transformed using the corresponding t-value (power-trans(y, t-value)).
A: Abundance

Fauna group	*t-value*	*Final model structures*
Nematode (Ne)	0.54	power-trans(y, 0.54) ~ Soil C + Leaf C:N, random = ~ 1|composition; R^2m=0.15 (R^2c=0.635)
Ne:Herbivores	0.47	power-trans(y, 0.47) ~ Leaf C:N, random = ~ 1|composition; R^2m=0.143 (R^2c=0.196)
Ne:Bacterivores	0.55	power-trans(y, 0.55) ~ Leaf C:N + Soil NO_3^-, random = ~ 1|composition; R^2m=0.096 (R^2c=0.096)
Ne:Fungivores	0.60	power-trans(y, 0.6) ~ Soil NO_3^-, random = ~ 1|composition; R^2m=0.061 (R^2c=0.156)
Mesofauna (Me)	0.44	power-trans(y, 0.44) ~ Soil C + ENL, random = ~ 1|composition; R^2m=0.173 (R^2c=0.52)
Me:Fungivores	0.26	power-trans(y, 0.26) ~ ENL + Root Ca, random = ~ 1|composition; R^2m=0.095 (R^2c=0.095)
Me:Omnivores	0.23	power-trans(y, 0.23) ~ Soil C + Root C + ENL, random = ~ 1|composition; R^2m=0.203 (R^2c=0.358)
Me:Predators	0.31	power-trans(y, 0.31) ~ ENL + Root Ca, random = ~ 1|composition; R2m=0.098 (R2c=0.194)
Macrofauna (Ma)	0.43	power-trans(y, 0.43) ~ Soil C + Root Ca, random = ~ 1|composition; R^2m=0.109 (R^2c=0.109)
Ma: Predators	0.58	power-trans(y, 0.58) ~ ENL + Root Ca, random = ~ 1|composition; R^2m=0.111 (R^2c=0.569)
Ma: Omnivores	0.58	power-trans(y, 0.58) ~ ENL + Root Ca, random = ~ 1|composition; R^2m=0.094 (R^2c=0.094)
Earthworm	0.46	power-trans(y, 0.46) ~ Soil C + Root C + Soil NO_3^-, random = ~ 1|composition; R^2m=0.144 (R^2c=0.144)
Microbe: total	0.69	power-trans(y, 0.69) ~ Root C + ENL, random = ~ 1|composition; R^2m=0.192 (R^2c=0.287)
Soil fungi	0.71	power-trans(y, 0.71) ~ Root C + ENL + Root Ca, random = ~ 1|composition; R^2m=0.121 (R^2c=0.388)
Soil bacteria	0.84	power-trans(y, 0.84) ~ ENL, random = ~ 1|composition; R^2m=0.058 (R^2c=0.144)

B: Taxonomic richness

Fauna group	*t-value*	*Final model structures*
Nematode (Ne)	0.53	power-trans(y, 0.53) ~ Leaf C:N + Soil NO_3^-, random = ~ 1|composition; R^2m=0.108 (R^2c=0.409)
Ne:Herbivores	0.78	power-trans(y, 0.78) ~ Leaf C:N , random = ~ 1|composition; R^2m=0.111 (R^2c=0.372)
Ne:Bacterivores	0.63	power-trans(y, 0.63) ~ Leaf C:N + Soil NO_3^-, random = ~ 1|composition; R^2m=0.128 (R^2c=0.37)
Ne:Fungivores	0.76	power-trans(y, 0.76) ~ 1 , random = ~ 1|composition;
Mesofauna (Me)	1.00	power-trans(y, 1) ~ Soil C + ENL, random = ~ 1|composition; R^2m=0.088 (R^2c=0.088)
Me:Fungivores	1.05	power-trans(y, 1.05) ~ ENL, random = ~ 1|composition; R^2m=0.044 (R^2c=0.215)
Me:Omnivores	0.91	power-trans(y, 0.91) ~ Root Ca , random = ~ 1|composition; R^2m=0.031 (R^2c=0.031)
Me:Predators	0.96	power-trans(y, 0.96) ~ Soil C, random = ~ 1|composition; R^2m=0.048 (R^2c=0.089)
Macrofauna (Ma)	1.07	power-trans(y, 1.07) ~ Soil C + Root Ca, random = ~ 1|composition; R^2m=0.151 (R^2c=0.381)
Ma: Predators	0.75	power-trans(y, 0.75) ~ Root Ca, random = ~ 1|composition; R^2m=0.034 (R^2c=0.529)
Ma: Omnivores	1.09	power-trans(y, 1.09) ~ ENL, random = ~ 1|composition; R^2m=0.128 (R^2c=0.166)
Earthworm	0.65	power-trans(y, 0.65) ~ Soil C, random = ~ 1|composition; R^2m=0.089 (R^2c=0.167)
Microbe: total	0.20	power-trans(y, 0.2) ~ Leaf C:N + ENL, random = ~ 1|composition; R^2m=0.276 (R^2c=0.758)
Soil fungi	0.56	power-trans(y, 0.56) ~ Leaf C:N + ENL, random = ~ 1|composition; R^2m=0.221 (R^2c=0.875)
Soil bacteria	0.48	power-trans(y, 0.48) ~ Leaf C:N + ENL, random = ~ 1|composition; R^2m=0.184 (R^2c=0.539)

C: Shannon-Wiener diversity index

Fauna group	*t-value*	*Final model structures*
Nematode	1.19	power-trans(y, 1.19) ~ Leaf C:N + + Soil NO_3^-, random = ~ 1|composition; R^2m=0.11 (R^2c=0.434)
Mesofauna	0.93	power-trans(y, 0.93) ~ Leaf C:N + Root Ca + + Soil NO_3^-, random = ~ 1|composition; R^2m=0.284 (R^2c=0.284)
Macrofauna	1.28	power-trans(y, 1.28) ~ Root Ca, random = ~ 1|composition; R^2m=0.087 (R^2c=0.653)
Microbe: total	1.24	power-trans(y, 1.24) ~ Leaf C:N + ENL, random = ~ 1|composition; R^2m=0.267 (R^2c=0.624)
Soil fungi	1.30	power-trans(y, 1.3) ~ Leaf C:N + ENL, random = ~ 1|composition; R^2m=0.134 (R^2c=0.795)
Soil bacteria	0.89	power-trans(y, 0.89) ~ Leaf C:N + ENL, random = ~ 1|composition; R^2m=0.147 (R^2c=0.276)

Supplementary references;

1. Hines, J. *et al.* A meta food web for invertebrate species collected in a European grassland. *Ecology* **100**, e02679 (2019).
2. Chinery, M. *Pareys Buch der Insekten: über 2000 Insekten Europas*. (Kosmos, 2012).
3. Laaksonen, M., Murdoch, K., Siitonen, J. & Várkonyi, G. Habitat associations of Agathidium pulchellum, an endangered old-growth forest beetle species living on slime moulds. *J Insect Conserv* **14**, 89–98 (2010).
4. Chandler, D. S. A Revision of the New World Cyclodinus Mulsant & Rey (Coleoptera: Anthicidae). *Transactions of the American Entomological Society (1890-)* **131**, 1–20 (2005).
5. Majka, C. G. The Anthicidae and Ischaliidae (Coleoptera) of Atlantic Canada. (2011).
6. Scherber, C. *et al.* Bottom-up effects of plant diversity on multitrophic interactions in a biodiversity experiment. *Nature* **468**, 553–556 (2010).
7. Goulet, H. & Huber, J. *Hymenoptera of the World: An Identification Guide to Families*. *Ottawa: Agriculture Canada* (1993).
8. Smith, D. R. The Xiphydriid Woodwasps of North America (Hymenoptera: Xiphydriidae). *Transactions of the American Entomological Society (1890-)* **102**, 101–131 (1976).
9. Wheeler, A. G. *Biology of the Plant Bugs (Hemiptera: Miridae): Pests, Predators, Opportunists*. (Cornell University Press, 2001).
10. Potapov, A. M. *et al.* Feeding habits and multifunctional classification of soil-associated consumers from protists to vertebrates. *Biological Reviews* **97**, 1057–1117 (2022).
11. Devetak, D. & Kloko, V. The feeding biology of adult lacewings (Neuroptera): a review. *Trends in Entomology* **12**, 29–42 (2016).
12. Schneider, N., Zaenker, S. & Dorow, W. H. O. Staubläuse (Psocodea, ’Psocoptera’) aus zoologischen Untersuchungen in Hessen und den angrenzenden Gebieten. (2012).

13. Jessica, D., Verheggen, F., Lognay, G. & Haubruge, E. Large carrion beetles (Coleoptera, Silphidae) in Western Europe: A review. *Biotechnologie, Agronomie, Société et Environnement* **15**, 435–447 (2011).
14. Majoros, S. E. & Adamowicz, S. J. *CanFlyet: Habitat Zone and Diet Trait Dataset for Diptera Species of Canada and Greenland*. (2023) doi:10.1101/2023.03.15.532812.
15. Köhler, M. *et al.* The effects of tree diversity and neighborhood on phyllosphere fungal communities. *Fungal Ecology* **76**, 101440 (2025).
16. Hendrix, P. F. *et al.* Detritus Food Webs in Conventional and No-Tillage Agroecosystems. *BioScience* **36**, 374–380 (1986).
17. Coleman, D. C., Crossley, D. A. & Hendrix, P. F. *Fundamentals of Soil Ecology*. (Academic Press, 2004).
18. Mulder, C. & Vonk, J. A. Nematode traits and environmental constraints in 200 soil systems: scaling within the 60–6000 µm body size range. *Ecology* **92**, 2004–2004 (2011).
19. Nemaplex.UCDavis.edu. Nemaplex. http://nemaplex.ucdavis.edu/ (2024).
20. Yeates, G. W., Bongers, T., De Goede, R. G. M., Freckman, D. W. & Georgieva, S. S. Feeding Habits in Soil Nematode Families and Genera—An Outline for Soil Ecologists. *J Nematol* **25**, 315–331 (1993).
21. Ferris, H., Bongers, T. & De Goede, R. G. M. A framework for soil food web diagnostics: extension of the nematode faunal analysis concept. *Applied Soil Ecology* **18**, 13–29 (2001).
22. Dietrich, P., Cesarz, S., Liu, T., Roscher, C. & Eisenhauer, N. Effects of plant species diversity on nematode community composition and diversity in a long-term biodiversity experiment. *Oecologia* **197**, 297–311 (2021).
23. Maraun, M. *et al.* New perspectives on soil animal trophic ecology through the lens of C and N stable isotope ratios of oribatid mites. *Soil Biology and Biochemistry* **177**, 108890 (2023).
24. Crotty, F. V. & Adl, S. M. Competition and Predation in Soil Fungivorous Microarthropods Using Stable Isotope Ratio Mass Spectrometry. *Front. Microbiol.* **10**, (2019).

25. Klarner, B., Maraun, M. & Scheu, S. Trophic diversity and niche partitioning in a species rich predator guild – Natural variations in stable isotope ratios (13C/12C, 15N/14N) of mesostigmatid mites (Acari, Mesostigmata) from Central European beech forests. *Soil Biology and Biochemistry* **57**, 327–333 (2013).

26. Haynert, K., Kiggen, M., Klarner, B., Maraun, M. & Scheu, S. The structure of salt marsh soil mesofauna food webs – The prevalence of disturbance. *PLOS ONE* **12**, e0189645 (2017).

27. Cuthbertson, A. G. S., Qiu, B.-L. & Murchie, A. K. Anystis baccarum: An Important Generalist Predatory Mite to be Considered in Apple Orchard Pest Management Strategies. *Insects* **5**, 615–628 (2014).

28. Hernandes, F. A., Bauchan, G. R. & Ochoa, R. New and little known feather mites (Acariformes: Astigmata) analysed with low-temperature scanning electron microscopy. *International Journal of Acarology* **43**, 499–517 (2017).

29. Zhang, Y., Zhang, Z.-Q., Lin, J. & Liu, Q. Predation of Amblyseius longispinosus (Acari: Phytoseiidae) on Aponychus corpuzae (Acari: Tetranychidae). *Systematic and Applied Acarology* 53–58 (1998) doi:10.11158/saa.3.1.8.

30. Sendra, A., Jiménez-Valverde, A., Selfa, J. & Reboleira, A. S. P. S. Diversity, ecology, distribution and biogeography of Diplura. *Insect Conservation and Diversity* **14**, 415–425 (2021).

31. Sherlock, E. *Key to the Earthworms of the UK and Ireland*. (FSC Publications, 2018).

32. Scheu, S. & Falca, M. The soil food web of two beech forests (Fagus sylvatica) of contrasting humus type: stable isotope analysis of a macro- and a mesofauna-dominated community. *Oecologia* **123**, 285–296 (2000).

33. *American Beetles, Volume II: Polyphaga: Scarabaeoidea through Curculionoidea*. (CRC Press, Boca Raton, 2002). doi:10.1201/9781420041231.

34. Eisenhauer, N. *Created in BioRender*. (2026) https://BioRender.com/ jvnwool.

35. Ray, T. *et al.* Tree diversity increases productivity through enhancing structural complexity across mycorrhizal types. *Science Advances* **9**, eadi2362 (2023).

36. Deng, X. *et al.* Forest biodiversity increases productivity via complementarity from greater canopy structural complexity. *Proceedings of the National Academy of Sciences* **122**, e2506750122 (2025).

37. Dietrich, P. *et al.* Tree diversity effects on productivity depend on mycorrhizae and life strategies in a temperate forest experiment. *Ecology* **104**, e3896 (2023).

38. Huang, Y. *et al.* Impacts of species richness on productivity in a large-scale subtropical forest experiment. *Science* **362**, 80–83 (2018).

39. Bönisch, E. *et al.* Mycorrhizal type and tree diversity affect foliar elemental pools and stoichiometry. *New Phytologist* **242**, 1614–1629 (2024).

40. Ren, J. *et al.* Tree growth response to soil nutrients and neighborhood crowding varies between mycorrhizal types in an old-growth temperate forest. *Oecologia* **197**, 523–535 (2021).

41. Averill, C., Bhatnagar, J. M., Dietze, M. C., Pearse, W. D. & Kivlin, S. N. Global imprint of mycorrhizal fungi on whole-plant nutrient economics. *Proceedings of the National Academy of Sciences* **116**, 23163–23168 (2019).

42. Phillips, R. P., Brzostek, E. & Midgley, M. G. The mycorrhizal-associated nutrient economy: a new framework for predicting carbon–nutrient couplings in temperate forests. *New Phytologist* **199**, 41–51 (2013).

43. Terrer, C., Vicca, S., Hungate, B. A., Phillips, R. P. & Prentice, I. C. Mycorrhizal association as a primary control of the CO2 fertilization effect. *Science* **353**, 72–74 (2016).

44. Schuldt, A. *et al.* Multiple plant diversity components drive consumer communities across ecosystems. *Nat Commun* **10**, 1460 (2019).

45. Staab, M. & Schuldt, A. The Influence of Tree Diversity on Natural Enemies—a Review of the “Enemies” Hypothesis in Forests. *Curr Forestry Rep* **6**, 243–259 (2020).

46. Root, R. B. Organization of a Plant-Arthropod Association in Simple and Diverse Habitats: The Fauna of Collards (Brassica Oleracea). *Ecological Monographs* **43**, 95–124 (1973).

47. Muiruri, E. W. *et al.* Forest diversity effects on insect herbivores: do leaf traits matter? *New Phytologist* **221**, 2250–2260 (2019).

48. Schuldt, A. *et al.* Plant traits affecting herbivory on tree recruits in highly diverse subtropical forests. *Ecology Letters* **15**, 732–739 (2012).

49. Huang, Y. *et al.* Enhanced stability of grassland soil temperature by plant diversity. *Nat. Geosci.* **17**, 44–50 (2024).

50. Schnabel, F. *et al.* Tree Diversity Increases Forest Temperature Buffering via Enhancing Canopy Density and Structural Diversity. *Ecology Letters* **28**, e70096 (2025).

51. Heijden, M. G. A., Martin, F. M., Selosse, M. & Sanders, I. R. Mycorrhizal ecology and evolution: the past, the present, and the future. *New Phytol* **205**, 1406–1423 (2015).

52. Chen, M. *et al.* Changes in Chinese fir plantations root exudation strategies seasonally and as tree age. *Forest Ecology and Management* **545**, 121239 (2023).

53. Eisenhauer, N. *et al.* Root biomass and exudates link plant diversity with soil bacterial and fungal biomass. *Sci Rep* **7**, 44641 (2017).

54. Eisenhauer, N. *et al.* Plant diversity effects on soil food webs are stronger than those of elevated CO2 and N deposition in a long-term grassland experiment. *Proceedings of the National Academy of Sciences* **110**, 6889–6894 (2013).

55. Huang, Y. *et al.* Positive effects of tree species diversity on litterfall quantity and quality along a secondary successional chronosequence in a subtropical forest. *Journal of Plant Ecology* **10**, 28–35 (2017).

56. Hooper, D. U. *et al.* Interactions between Aboveground and Belowground Biodiversity in Terrestrial Ecosystems: Patterns, Mechanisms, and Feedbacks. *BioScience* **50**, 1049–1061 (2000).

57. Peng, Y. *et al.* Litter quality, mycorrhizal association, and soil properties regulate effects of tree species on the soil fauna community. *Geoderma* **407**, 115570 (2022).

58. Chen, X. *et al.* Tree diversity increases decadal forest soil carbon and nitrogen accrual. *Nature* **618**, 94–101 (2023).
59. Brassard, B. W. *et al.* Tree species diversity increases fine root productivity through increased soil volume filling. *Journal of Ecology* **101**, 210–219 (2013).
60. Ma, Z. & Chen, H. Y. H. Effects of species diversity on fine root productivity in diverse ecosystems: a global meta-analysis. *Global Ecology and Biogeography* **25**, 1387–1396 (2016).
61. Sun, Z. *et al.* Positive effects of tree species richness on fine-root production in a subtropical forest in SE-China. *J Plant Ecol* **10**, 146–157 (2017).
62. Zheng, J. *et al.* A trait-based root acquisition-defence-decomposition framework in angiosperm tree species. *Nat Commun* **15**, 5311 (2024).
63. Luo, S. *et al.* Higher productivity in forests with mixed mycorrhizal strategies. *Nature Communications* **14**, 1377 (2023).
64. Bergmann, J. *et al.* The fungal collaboration gradient dominates the root economics space in plants. *Science Advances* **6**, eaba3756 (2020).
65. Chen, W. *et al.* Root morphology and mycorrhizal symbioses together shape nutrient foraging strategies of temperate trees. *Proc. Natl. Acad. Sci. U.S.A.* **113**, 8741–8746 (2016).
66. Bardgett, R. D. & Wardle, D. A. *Aboveground-Belowground Linkages: Biotic Interactions, Ecosystem Processes, and Global Change*. (Oxford University Press, 2010).
67. Reich, P. B. *et al.* Linking litter calcium, earthworms and soil properties: a common garden test with 14 tree species. *Ecology Letters* **8**, 811–818 (2005).
68. Schelfhout, S. *et al.* Tree Species Identity Shapes Earthworm Communities. *Forests* **8**, 85 (2017).
69. Mueller, K. E. *et al.* Light, earthworms, and soil resources as predictors of diversity of 10 soil invertebrate groups across monocultures of 14 tree species. *Soil Biology and Biochemistry* **92**, 184–198 (2016).
70. Zhou, Z. *et al.* Plant roots fuel tropical soil animal communities. *Ecology Letters* **26**, 742–753 (2023).

71. Smith, S. E. & Read, D. J. *Mycorrhizal Symbiosis*. (Academic Press, 2010).
72. Bongers, T. The maturity index: an ecological measure of environmental disturbance based on nematode species composition. *Oecologia* **83**, 14–19 (1990).
73. de Vries, F. T. *et al.* Soil food web properties explain ecosystem services across European land use systems. *Proceedings of the National Academy of Sciences* **110**, 14296–14301 (2013).
74. Tedersoo, L., Bahram, M. & Zobel, M. How mycorrhizal associations drive plant population and community biology. *Science* **367**, eaba1223 (2020).
75. Bardgett, R. D., Hobbs, P. J. & Frostegård, Å. Changes in soil fungal:bacterial biomass ratios following reductions in the intensity of management of an upland grassland. *Biol Fert Soils* **22**, 261–264 (1996).
76. Heděnec, P. *et al.* Tree species traits and mycorrhizal association shape soil microbial communities via litter quality and species mediated soil properties. *Forest Ecology and Management* **527**, 120608 (2023).
77. Cortois, R. *et al.* Possible mechanisms underlying abundance and diversity responses of nematode communities to plant diversity. *Ecosphere* **8**, e01719 (2017).
78. Marian, F., Brown, L., Sandmann, D., Maraun, M. & Scheu, S. Roots, mycorrhizal fungi and altitude as determinants of litter decomposition and soil animal communities in tropical montane rainforests. *PLANT AND SOIL* **438**, 1–18 (2019).
