# Supplementary material for: Tree diversity and mycorrhizal type independently shape multitrophic biodiversity, with stronger effects belowground than aboveground
Source: Nat Ecol Evol. 2026 Aug 31;10(9):1628–43. doi: 10.1038/s41559-026-03147-6 (PMC13541612; doi:10.1038/s41559-026-03147-6)
Supplement: Supplementary file 2 [file 41559_2026_3147_MOESM2_ESM.pdf]

nature portfolio

Corresponding author(s): Huimin Yi

Last updated by author(s): Jun 23, 2026

Reporting Summary

Nature Portfolio wishes to improve the reproducibility of the work that we publish. This form provides structure for consistency and transparency in reporting. For further information on Nature Portfolio policies, see our Editorial Policies and the Editorial Policy Checklist.

Statistics

For all statistical analyses, confirm that the following items are present in the figure legend, table legend, main text, or Methods section.

n/a	Confirmed	
☐	☒	The exact sample size (n) for each experimental group/condition, given as a discrete number and unit of measurement
☐	☒	A statement on whether measurements were taken from distinct samples or whether the same sample was measured repeatedly
☐	☒	The statistical test(s) used AND whether they are one- or two-sided<br>*Only common tests should be described solely by name; describe more complex techniques in the Methods section.*
☐	☒	A description of all covariates tested
☐	☒	A description of any assumptions or corrections, such as tests of normality and adjustment for multiple comparisons
☐	☒	A full description of the statistical parameters including central tendency (e.g. means) or other basic estimates (e.g. regression coefficient) AND variation (e.g. standard deviation) or associated estimates of uncertainty (e.g. confidence intervals)
☐	☒	For null hypothesis testing, the test statistic (e.g. F, t, r) with confidence intervals, effect sizes, degrees of freedom and P value noted<br>*Give P values as exact values whenever suitable.*
☒	☐	For Bayesian analysis, information on the choice of priors and Markov chain Monte Carlo settings
☐	☒	For hierarchical and complex designs, identification of the appropriate level for tests and full reporting of outcomes
☐	☒	Estimates of effect sizes (e.g. Cohen's d, Pearson's r), indicating how they were calculated

Our web collection on statistics for biologists contains articles on many of the points above.

Software and code

Policy information about availability of computer code

Data collection	No software was used for data collection.
Data analysis	All data analyses were conducted in R version 4.5.3 (R Core Team, 2026), using the packages 'vegan', 'nlme', 'MuMIn', 'piecewiseSEM', and 'parameters'. Further details are provided in the Methods section. All R scripts used for the statistical analyses are stored in the MyDiv database (https://mydivdata.idiv.de).

For manuscripts utilizing custom algorithms or software that are central to the research but not yet described in published literature, software must be made available to editors and reviewers. We strongly encourage code deposition in a community repository (e.g. GitHub). See the Nature Portfolio guidelines for submitting code & software for further information.

Data

Policy information about availability of data

All manuscripts must include a data availability statement. This statement should provide the following information, where applicable:

- Accession codes, unique identifiers, or web links for publicly available datasets
- A description of any restrictions on data availability
- For clinical datasets or third party data, please ensure that the statement adheres to our policy

This study did not use any publicly available code.
Taxonomic information, trophic group classifications, and R scripts are available at https://doi.org/10.25829/AEKQ-V240.

All data used in this study are stored in the MyDiv database (https://mydivdata.idiv.de). Most datasets are publicly available. Plot-level biodiversity metrics across food-web compartments are available at https://doi.org/10.25829/KJ6F-QG85. Leaf nutrient data are available at https://doi.org/10.25829/MFDW-KW26. Canopy complexity data are available at https://doi.org/10.25829/58EF-HN10. Soil parameters are available at https://doi.org/10.25829/Z5KP-BV11 and https://doi.org/10.25829/VY0G-B534. Root nutrient data (dataset ID 213) are available upon reasonable request because the data owners are preparing related publications.

Research involving human participants, their data, or biological material

Policy information about studies with human participants or human data. See also policy information about sex, gender (identity/presentation), and sexual orientation and race, ethnicity and racism.

Reporting on sex and gender	N/A
Reporting on race, ethnicity, or other socially relevant groupings	N/A
Population characteristics	N/A
Recruitment	N/A
Ethics oversight	N/A

Note that full information on the approval of the study protocol must also be provided in the manuscript.

Field-specific reporting

Please select the one below that is the best fit for your research. If you are not sure, read the appropriate sections before making your selection.

☐ Life sciences ☐ Behavioural & social sciences ☒ Ecological, evolutionary & environmental sciences

For a reference copy of the document with all sections, see nature.com/documents/nr-reporting-summary-flat.pdf

Ecological, evolutionary & environmental sciences study design

All studies must disclose on these points even when the disclosure is negative.

Study description

Global tree diversity loss may threaten ecosystem functioning through cascading declines in multitrophic biodiversity. Meanwhile, climate warming, drought, and anthropogenic pressures may shift mycorrhizal dominance in forests, potentially leading to changes in the biodiversity at higher trophic levels. While impacts on vertebrates are well studied, consequences for microorganisms and invertebrates remain poorly understood. Here, we build on the MyDiv tree–mycorrhiza diversity experiment, which manipulates a tree diversity gradient (1, 2, and 4 species) and mycorrhizal type (communities of only arbuscular mycorrhizal (AM) trees, only ectomycorrhizal (EcM) trees, or an equal mixture of both). In total, the experiment comprises eight treatments, each replicated ten times, resulting in 80 plots. We assessed biodiversity across aboveground, soil surface, and belowground compartments by sampling microorganisms (foliar fungi and soil microbes) and invertebrates spanning multiple trophic levels, together with measurements of tree biomass, canopy complexity, and nutrient concentrations in leaves, roots, and soil.

Tree diversity significantly increased belowground abundance and taxonomic richness of microorganisms and lower-trophic-level nematodes, cascading to meso- and macrofauna predators; but aboveground effects were limited to foliar fungi and soil-surface omnivores. Mycorrhizal type strongly structured belowground food webs and influenced aboveground predators, but mixtures only showed additive effects. For most trophic groups, diversity responses were similar across mycorrhizal types. Tree diversity and mycorrhizal type effects were mediated by leaf quality, canopy complexity, and nutrient availability. Our results highlight tree diversity and mycorrhizal type as key drivers of multitrophic biodiversity, primarily governing belowground bottom-up effects.

Research sample

At the beginning and end of the 2021 vegetation season, we sampled invertebrates from the tree canopy (aboveground) and soil surface with different methods. The belowground invertebrates were sampled from mineral soil (depth of 0-10 cm) by using three distinct methods to collect earthworms, nematodes, respectively, as well as meso- and macrofauna. All invertebrates were morphologically identified, and their abundances were recorded and resulted in 100,000 individuals. At the same time, we assessed the biomass of soil fungi and bacteria. Each of these groups comprised 160 samples (8 treatments × 10 replicates × 2 sampling seasons). At the end of the vegetation season, we further assessed the diversity of soil fungi and bacteria using DNA sequencing, resulting in 80 samples for each group. Foliar fungal diversity was also analyzed using DNA sequencing across 42 plots, excluding these plots planted with Aesculus hippocastanum (AM tree species) and Tilia platyphyllos (EcM tree species). Additionally, we assessed each tree community by measuring total tree volume, canopy complexity, and nutrient concentrations in leaves, roots, and soil once in 2021.

Sampling strategy

To account for seasonal differences, for most the organism groups, we sampled twice, at the beginning and end of the vegetation seasons.
Different sampling methods were used for different organism groups to ensure representative coverage of each plot.
Because most sampling activities were carried out within the same time, efforts were made to avoid overlap among sampling

locations. All samples were collected from the core areas of the plots (8 × 8 m within the 11 × 11 m plot), two rows of trees away from the plot edges.

Data collection	We collected canopy fauna using branch beating and soil surface fauna through a combination of sieving and hand-sorting. Earthworms were sampled using a combination of digging and hand-sorting, followed by the application of a mustard solution. Soil meso- and macrofauna (excluding earthworms) were extracted using heat extraction, while soil nematodes were extracted with a modified Baermann method. Soil mesofauna and nematodes were identified by taxonomic experts, whereas all other groups were identified by the first author using standard taxonomic keys. In total, we identified 100,000 invertebrate individuals, over 250 taxonomic groups. The biomass of soil fungi and bacteria was determined using the maximum initial respiratory response to D-glucose addition, and the biomass ratio of fungi to bacteria was assessed based on phospholipid fatty acid (PLFA) profiles. The diversity of soil fungi, soil bacteria, and foliar fungi was assessed using DNA sequencing.
Timing and spatial scale	The beginning of the vegetation season occurred in May and early June 2021, while the end of the vegetation season took place in September and early October 2021. Samples were collected from the tree canopy (aboveground), soil surface, and mineral soil (0–10 cm depth; belowground).
Data exclusions	We identified and removed outliers for each biodiversity metric prior to max-min transformation. Outliers were detected using the interquartile range (IQR) method, to account for the potential for extreme values arising from organisms with small body sizes, such as aphids in the tree canopy. Specifically, we assessed whether values below the 5th percentile fell below the lower fence (Q1 – 1.5 × IQR) or whether values above the 95th percentile exceeded the upper fence (Q3 + 1.5 × IQR).<br>Foliar fungi samples below 5000 raw reads were excluded from further analysis.<br>The ASVs (Amplicon Sequence Variants) assigned to soil fungi and bacteria at least at the phylum level were retained; taxa that could not be assigned to the phylum level were removed.
Reproducibility	We reported the effects of tree diversity and mycorrhizal type on invertebrate biodiversity indices and on soil fungal and bacterial biomass. All of these effects were consistent across both sampling seasons.<br>The treatment effects on the biodiversity of invertebrates and microorganisms were confirmed using both min–max transformed data and the original data. Our conclusions are based on both significance levels and effect sizes.<br>Moreover, we will publish the original data and the processed code to enable readers to reproduce all results presented in the manuscript.
Randomization	All samples were collected from the core area of each plot (8 × 8 m within the 11 × 11 m plot). In the southwestern quadrant of each plot, five soil samples were randomly collected to assess soil microbial communities and extract nematodes. Within the same quadrant, a 50 × 50 cm area was randomly selected for earthworm extraction. One soil core (diameter = 16 cm) was randomly collected from the northwestern quadrant for the extraction of soil meso- and macrofauna. Soil surface fauna were collected from a 50 × 50 cm area randomly selected in the northeastern quadrant. In the southeastern quadrant, canopy arthropods were collected from four adjacent trees within a 1 × 1 m area, and this procedure was repeated four times within the same quadrant. We assumed uniform environmental conditions across the four quadrants; thus, sampling in one quadrant was considered representative of the entire core area. Overlap between different sampling activities was avoided to minimize potential artifacts caused by concurrent measurements.<br>Tree composition represented the replicates of the treatment factors (tree diversity and mycorrhizal type). Therefore, we accounted for random variation using mixed-effects models, including the interaction between tree composition and sampling season as a random factor. This approach allowed us to capture the variability in responses among different tree compositions across the two sampling seasons.
Blinding	N/A

Did the study involve field work? ☒ Yes ☐ No

Field work, collection and transport

Field conditions	The site has a continental summer-dry climate, with an average annual temperature of 8.8°C and mean annual precipitation of 484 mm. The soil at the MyDiv site is highly fertile, classified as Haplic Chernozem, with a pH ranging from 6.6 to 7.4, and features a thick humus layer. The land had historically been used for agriculture until 2012 and was then transformed to a hay meadow.
Location	MyDiv site is located at the Bad Lauchstädt Experimental Research Station of the Helmholtz Centre for Environmental Research-UFZ, southwest of Halle, Saxony-Anhalt, Germany (51°23′ N, 11°53′ E; elevation: 114-116 m a.s.l.).
Access & import/export	Access to site and data collection by the principle investigator at each site followed standard practices and complies with laws.
Disturbance	N/A

Reporting for specific materials, systems and methods

We require information from authors about some types of materials, experimental systems and methods used in many studies. Here, indicate whether each material, system or method listed is relevant to your study. If you are not sure if a list item applies to your research, read the appropriate section before selecting a response.

Materials & experimental systems

n/a	Involved in the study	
☒	☐	Antibodies
☒	☐	Eukaryotic cell lines
☒	☐	Palaeontology and archaeology
☐	☒	Animals and other organisms
☒	☐	Clinical data
☒	☐	Dual use research of concern
☐	☒	Plants

Methods

n/a	Involved in the study	
☒	☐	ChIP-seq
☒	☐	Flow cytometry
☒	☐	MRI-based neuroimaging

Animals and other research organisms

Policy information about studies involving animals; ARRIVE guidelines recommended for reporting animal research, and Sex and Gender in Research

Laboratory animals	No laboratory animals were used in the study.
Wild animals	All collected wild animals were invertebrates. Canopy and soil surface fauna, as well as earthworms, were immediately preserved in 70% ethanol after collecting from field and subsequently transported. Soil meso- and macrofauna were extracted immediately in a field laboratory located near the study site. Soil samples containing nematodes were transported in cooled boxes to the main laboratory, where nematodes were extracted from the soil.
Reporting on sex	N/A
Field-collected samples	All collected invertebrates (except soil nematodes) were preserved in 70% ethanol and stored in a storage room at 20 °C. Extracted nematodes were preserved in 4% formaldehyde solution. Soil samples used for microbial biomass assessment were temporarily stored at 4 °C until processing. Soil samples for microbial community composition (phospholipid fatty acid analysis) and DNA analysis were stored at −20 °C immediately after fieldwork. All identifications and measurements were conducted in the laboratory at room temperature (20 °C).
Ethics oversight	Ethical approval was not required.

Note that full information on the approval of the study protocol must also be provided in the manuscript.

Dual use research of concern

Policy information about dual use research of concern

Hazards

Could the accidental, deliberate or reckless misuse of agents or technologies generated in the work, or the application of information presented in the manuscript, pose a threat to:

No	Yes	
☒	☐	Public health
☒	☐	National security
☒	☐	Crops and/or livestock
☒	☐	Ecosystems
☒	☐	Any other significant area

Experiments of concern

Does the work involve any of these experiments of concern:

No	Yes	
☒	☐	Demonstrate how to render a vaccine ineffective
☒	☐	Confer resistance to therapeutically useful antibiotics or antiviral agents
☒	☐	Enhance the virulence of a pathogen or render a nonpathogen virulent
☒	☐	Increase transmissibility of a pathogen
☒	☐	Alter the host range of a pathogen
☒	☐	Enable evasion of diagnostic/detection modalities
☒	☐	Enable the weaponization of a biological agent or toxin
☒	☐	Any other potentially harmful combination of experiments and agents

Plants

Seed stocks	No seed stocks were used or generated in this study. All trees were planted as saplings; therefore, seed-stock information is not applicable.
Novel plant genotypes	The tree species planted in the MyDiv Experiment are all commonly distributed in Germany and include Acer pseudoplatanus L., Aesculus hippocastanum L., Fraxinus excelsior L., Prunus avium L., Sorbus aucuparia L., Betula pendula Roth, Carpinus betulus L., Fagus sylvatica L., Quercus petraea (Matt.) Liebl., and Tilia platyphyllos Scop.No novel genotypes were used or generated in this study.
Authentication	No seed stocks and novel genotypes were used; therefore, additional authentication was not applicable.
