# Supplementary material for: Tree diversity and mycorrhizal type independently shape multitrophic biodiversity, with stronger effects belowground than aboveground
Source: Nat Ecol Evol. 2026 Aug 31;10(9):1628–43. doi: 10.1038/s41559-026-03147-6 (PMC13541612; doi:10.1038/s41559-026-03147-6)
Supplement: Supplementary file 3 [file 41559_2026_3147_MOESM3_ESM.pdf]

nature portfolio

Peer Review File

Tree diversity and mycorrhizal type independently shape multitrophic biodiversity, with stronger effects belowground than aboveground

Corresponding Author: Dr Huimin Yi

Version 0:

Decision Letter:

5th November 2025

*Please ensure you delete the link to your author homepage in this e-mail if you wish to forward it to your co-authors.

Dear Huimin,

Your Article entitled "Tree diversity more strongly promotes belowground than aboveground multitrophic biodiversity" has now been assessed by three reviewers. As you will see from their comments below, they find your work of considerable potential interest but have raised quite substantial concerns. In light of these comments, we would like to see a major revision along with a response to all the reviewers' comments.

We hope you will find the reviewers' comments useful as you decide how to proceed. Please do not hesitate to contact us if there are specific requests from the reviewers that you believe are technically impossible or unlikely to yield a meaningful outcome. If the reviewers' criticisms can be addressed without compromising the main conclusions, we will be happy to consider a revised manuscript. However, please bear in mind that we will be reluctant to continue the review process in the absence of major revisions, including additional analyses with the available data and reframing or removing those statements that the reviewers found too speculative in light of the data.

If revising your manuscript:

* Please provide the manuscript in Microsoft Word format and highlight all changes in the same file or a copy.

* Include a “Response to reviewers” document detailing, point-by-point, how you addressed each referee comment. If no action was taken to address a point, you must provide a compelling argument. This response will be sent back to the referees along with the revised manuscript.

* If you have not done so already we suggest that you begin to revise your manuscript so that it conforms to our Article format instructions at http://www.nature.com/natecolevol/info/final-submission. Refer also to any guidelines provided in this letter.

* Include a revised version of any required reporting checklist. It will be available to referees (and, potentially, statisticians) to aid in their evaluation if the manuscript goes back for peer review. A revised checklist is essential for re-review of the paper.

We would hope to receive the revised manuscript within 6 months. If you cannot send it within this time, please let us know. We will be happy to consider your revision so long as nothing similar has been accepted for publication at Nature Ecology & Evolution or published elsewhere. Once ready, please use the link below to submit a revised paper:

Link Redacted

Note: This URL links to your confidential home page and associated information about manuscripts you may have submitted, or that you are reviewing for us. If you wish to forward this email to co-authors, please delete the link to your homepage.

Nature Ecology & Evolution is committed to improving transparency in authorship. As part of our efforts in this direction, we are now requesting that all authors identified as ‘corresponding author’ on published papers create and link their Open Researcher and Contributor Identifier (ORCID) with their account on the Manuscript Tracking System (MTS), prior to

acceptance. This applies to primary research papers only. ORCID helps the scientific community achieve unambiguous attribution of all scholarly contributions. You can create and link your ORCID from the home page of the MTS by clicking on ‘Modify my Springer Nature account’. For more information please visit please visit www.springernature.com/orcid.

Please do not hesitate to contact me if you have any questions or would like to discuss the required revisions further.

[redacted]

Reviewers’ comments:

Reviewer #1 (Remarks to the Author):

The manuscript “Tree diversity more strongly promotes belowground than aboveground multitrophic diversity“ addresses how the diversity of trees impacts the diversity of other ecosystem components. This is a very interesting question, and the manuscript presents data from an impressive number of organisms. While this is undoubtedly an asset, it also presents a significant challenge for this study: to integrate the dynamics of all these different organisms into a cohesive, impactful narrative. Currently, I believe this task is not being met. In my opinion, the manuscript's writing is not clear and well-justified, as many of the statements seem unclear or speculative.

In addition, I feel this paper is missing one critical aspect of the study and this is understorey information. As understorey plants in Europe mostly form arbuscular mycorrhizas, these plants can play a crucial role in supporting the diversity of both above- and below-ground organisms. Hence, it would modify the effects of tree species in this experiment.

In addition, I am not entirely convinced that this setting, where the effect of tree species is explored not in multiple forests but in a relatively recent experimental forest with diversity ranging from 1 to 4, allows for drawing solid conclusions about the role of tree diversity on above- and belowground communities.

Moreover, all the trees in this experiment are broadleaf (which is great for an experimental setting), but the majority of the EcM trees in Europe are coniferous. This raises another aspect of the limited realism that can be offered in this study. Also, this setting makes links with other studies questionable (eg. statements like “indicating higher leaf N than AM plots (Fig. S1), which is in contrast to earlier findings of higher nutrient concentrations in AM tree leaves“)- is the referenced study also comparing only deciduous trees? I am convinced that this difference of broadleaf vs coniferous will have a significant impact on multiple measured aspects and hence it should be carefully explored is the presented aspects are valid.

Also, the writing in general could be improved (e.g., hypothesis 3 states “High-tree species richness communities with mixed-mycorrhizal types“, which is grammatically poor).

More specific comments:

Line 51 and 52: It remains unclear why these hypotheses are mentioned in the introduction when they are not included in the hypotheses or discussion part.

Line 83: Why was sampling done at the start and the end of the growing season? It is only mentioned in line 133, stating that the growing season has an effect, but this is rather obvious with deciduous plants.

Line 56-57: “bottom-up” effects are explained, but “top down” effects are not.

Line 106: what was measured?

Line 114: Comparisons with grasslands are made, while this study excludes herbaceous plants, I do not think this is correct.

Line 126-128 “The increase in predator richness contributed to the significant rise in taxonomic richness and Shannon-Wiener diversity of the total aboveground invertebrate communities (Fig. 1; Fig. S4).” It is confusing as Fig. 1 presents abundances, and it is difficult to follow what this sentence is based on.

Line 145-147: “Tree diversity enhanced fungal biomass, likely promoting saprotrophic fungi and, in turn, supporting higher abundance of nematode fungivores. ” This speculation of increasing saprotrophic fungi could be tested, for example, using a database such as FungalTraits to assign functional groups to fungal sequences and including those in the analyses. Also, I do not see the data supporting the increasing abundance of nematode fungivores.

Line 211: “The greater abundance of herbivorous nematodes, together with higher absorptive root turnover, may also explain why ” I do not see where the absorptive root turnover is measured, hence this seems speculative. Moreover, as pointed out earlier, I think this can additionally be influenced by the herbaceous vegetation which is not incorporated to the analyses.

Line 216-217: “low quality of leaf litter from EcM trees to have accumulated on the soil surface“ Is the low quality of ECM litter

demonstrated specifically on decidous EcM trees, which are incorporated to this study?

Line 220-221: This writing is misleading: “diversity of predators than AM and AM+EcM communities (Figs. 1B & 2B).“ As statistical difference was only between ECM and AM (not AM+ECM).

Line 222: Talking abvout longer lifespan of trees in a 6 years experiment seems inappropriate.

Line 506-507: Phospholipid fatty acid content is not suitable method for estimating microbial community composition.

Line 529: Information about bioinformatic analyses is missing.

Supplementary analyses line 146-148: . Lines were predicted based on the model: lme ($yi \sim month + Mycor * log(Richness)$), ~1| composition/month, data), with values averaged from both sampling seasons. I do not understand the analyses, why is month included when values are averaged from both sampling seasons?

Also, I find it highly disturbing that the manuscript does not present the number of fungal and bacterial richness (including ECM and AM fungal richness), to allow the reader to see if these conclusions can be made based on this data (for example higher AM fungal richness and abundance in plots with AM trees is a basic assumption that has to be met to justify any following analyses. The data presented in the supplementary material is based not on the raw values but on some groupings (omnivores etc.) and often represent min-max transformed values, which is not helpful for future research.

Reviewer #2 (Remarks to the Author):

This manuscript addresses a relevant and timely question in forest biodiversity research—how tree diversity and mycorrhizal associations jointly influence multi-trophic biodiversity across aboveground and belowground compartments. The authors used a large-scale tree-mycorrhizal diversity experiment (MyDiv) and integrated extensive datasets spanning microbial and invertebrate communities. However, while the manuscript presents some interesting findings, several major issues need to be addressed. My comments are outlined below.

1. Conceptual framework and hypotheses
•The study sets out four hypotheses (lines 92–101), yet the logical connections among them and the underlying ecological rationale could be clarified. For instance, Hypothesis (2) assumes stronger mycorrhizal effects belowground, while Hypothesis (3) assumes synergistic effects of AM+EcM mixing—but the mechanistic distinction between “additive” and “synergistic” outcomes is not clearly defined. Please revise the Introduction to more explicitly articulate how each hypothesis arises from existing theory (e.g., resource partitioning, functional complementarity, or the mycorrhizal-associated nutrient economy framework).
•The authors may also consider clarifying how the current study advances beyond prior MyDiv publications (e.g., Ferlian et al. 2018, Eisenhauer et al. 2023) that have examined similar belowground patterns. The novelty of integrating multi-trophic levels should be highlighted more explicitly.
2. Statistical and analytical methods
•The power-law framework used to model tree diversity effects (Fig. 1A, C, E) should be explained in greater detail in the Methods. Specifically, please clarify (1) why a power function was selected over other diversity–abundance models, (2) how scaling to [0,1] affects effect size interpretation, and (3) whether slopes were compared statistically among compartments.
•The structural equation model (SEM) is central to the paper but requires additional transparency: (1) Provide a supplementary figure or table describing the ecological rationale and evidence supporting each hypothesized path in the initial SEM. （2）Explain how multicollinearity among mediators (e.g., ENL, leaf C:N, root Ca) was handled. （3）Present total effects (direct + indirect) with confidence intervals and clarify how model selection affected path retention.
3. Interpretation of key results
•The manuscript convincingly shows that tree diversity promotes belowground biodiversity more strongly than aboveground. However, the discussion should better integrate mechanistic explanations for this pattern—such as differences in trophic connectivity, resource stability, and dispersal limitation between above- and belowground systems.
•The authors state that their findings partially support hypothesis (1) but contradict hypotheses (1a) and (1b) (Lines 111–119). This is confusing, as hypothesis (1) appears to consist of sub-hypotheses (1a) and (1b). If both 1a and 1b are contradicted, then hypothesis (1) as a whole should also be considered unsupported. Please clarify the logical relationship between these hypotheses and revise accordingly.
•As an experimental study, the authors have clearly highlighted main findings based on previous experimental research. However, it would be better to consider some relevant empirical studies, especially where the results diverge from earlier experimental findings. For instance, in lines 171–239, the authors report that AM+EcM mixtures have relatively weak effects on biodiversity, whereas AM and EcM tree communities exhibit distinct diversity patterns. This outcome is somewhat unexpected for experimental studies but aligns with patterns reported in empirical work supporting the EcM dominance hypothesis (e.g., Mao et al. 2023, Ecol Monogr, Luo et al. 2024. New Phytologist). Incorporating discussion of such empirical findings would help contextualize the results within a broader ecological framework.
•The statement regarding canopy complexity differences between AM and EcM tree communities should be qualified (Lines 184–186). In natural forests, canopy structural complexity indeed differs between these two mycorrhizal types. Therefore, this conclusion should be explicitly limited to the experimental forest communities examined here.
4. Figures and data presentation
•Figures 1 and 2 are informative but overly dense. Consider simplifying by grouping trophic levels or adding schematic

legends to enhance readability.
Additionally, clarify whether the shown relationships (e.g., solid vs. dashed lines) were tested across life forms or pooled across them.
•Figure 3 (SEM) is visually complex. I recommend moving the detailed trophic-level SEMs (currently Fig. 4) to Supplementary Materials and keeping a simplified version in the main text showing only key mediating paths.
5. Limitations and broader implications
•The Discussion would benefit from a short section on study limitations, particularly: (a) the relatively young age of the MyDiv trees (six years), (b) potential underestimation of aboveground effects due to canopy closure stage, and (c) lack of direct measurements of litter traits or decomposition rates, which could clarify the link between mycorrhizal type and resource heterogeneity.
•While the authors emphasize the applied implications for forest design, this point could be strengthened by discussing how tree–mycorrhiza diversity might be operationalized in restoration or carbon sequestration programs.
•Furthermore, the practical significance of this study could be strengthened by linking the results to forest dynamics under future global change. Several recent studies have projected a potential decline in EcM tree dominance and a corresponding increase in AM tree dominance in temperate forests under global change (e.g., Averill et al. 2018, Global Change Biology, Jo et al. 2019 Science Advances, Clavel et al. 2024. Ecography).
6. Other comments
•Title: "Mycorrhizal type" is important in the hypotheses and results, which I think should be included in the title.
•Some key concepts—particularly additive effects and synergistic effects—are used repeatedly but are not clearly defined or quantitatively described. Please provide explicit definitions and indicate how these effects were evaluated in the analyses.
•The classification of trophic levels in the manuscript is somewhat unclear and could be improved for greater ecological accuracy and reader comprehension. (1) Predators occur across multiple size classes (e.g., nematodes, microfauna, and macrofauna). From the perspective of the overall soil food web, there are also predator–prey interactions among these groups, which should be clearly distinguished when describing trophic relationships. (2) The classification of nematode trophic groups follows specific ecological terminology (e.g., bacterivores, fungivores, plant feeders, omnivores, and predators). It would be helpful to explicitly describe the criteria or references used to assign nematode taxa to different trophic categories. (3) Many soil organisms are omnivorous, and their trophic positions may shift depending on resource availability and environmental conditions. This ecological flexibility should be acknowledged and discussed, as it can influence interpretations of soil food-web structure and biodiversity patterns.

Reviewer #3 (Remarks to the Author):

An excellent paper by Yi and others demonstrating how tree diversity and mycorrhizal type shape multitrophic biodiversity across above- and belowground compartments. The experimental and statistical methodology are well-designed and robust, integrating extensive data from the MyDiv experiment with microbial, nematode, meso- and macrofaunal communities, plant traits, carbon and nutrient content. The authors show that belowground biodiversity responses to tree diversity are stronger than aboveground responses, which fills an existing knowledge gap. These responses are further linked to canopy structure, nutrient mediation, and mycorrhizal strategies to provide new mechanistic insight. This contribution is timely, with direct implications for the design of biodiversity-based reforestation and restoration strategies, highly novel, well contextualized in BEF theory and integrates above- and belowground data in a remarkably (given the complexity of the dataset) comprehensive way with some excellent figures. I have few moderate and several minor suggestions for improvement focused on statistical transparency and clarifying comparisons across compartments.

Main comments
Figs 1&2, Tables S1-S3: While standardized slopes and 95% CIs are shown, their biological magnitude is sometimes difficult to interpret. Could absolute abundance or richness changes be provided in supplementary tables to complement standardized effects, or in the supporting text (e.g. 'Tree diversity increased soil fungal richness by ≈ 3 OTUs per additional tree species').

Figs 1&2, Tables S4-S8: The conclusion that belowground responses are stronger relies mainly on differences in standardized effect sizes and significance levels. The paper would benefit from a more explicit statistical test comparing the magnitude of diversity effects between strata (e.g. hierarchical models with stratum × log(tree richness)).

The authors employ a solid statistical framework of mixed-effects models with random factors (composition, season), power transformations for scaling, and SEM to assess mediation. However, transparency and reproducibility could be improved by 1) providing model formulae (e.g. lme(response ~ log(tree richness) + mycorrhiza + interaction + month + (1|composition/month)) in the supplement; 2) reporting the number of plots per treatment per season explicitly in the methods (not clear); 3) provide a statement on how Type I error inflation was handled (e.g. FDR correction or emphasis on effect sizes rather than p-values); 4) Provide a SEM goodness-of-fit summary table (χ^2, RMSEA, CFI) in the supplement.

Minor Comments
Throughout, suggest replacing '– … –' with ', … ,' (e.g. L19-20, L39-40, L185)
L54: Remove 'the'
L57: Suggest replacing 'stronger' with 'more strongly'
Significance is sometimes interpreted at $p < 0.1$ (marginal) which needs to be used cautiously.
Statistical notation should be consistent (e.g. $p \leq 0.05$ v $P < 0.05$, Log v $\log_{10}$).
L110: "only marginal effects on predator richness ($p = 0.07$; Tables S4-5)" – I cannot see any figures in italic to signify this in

the supplementary tables.
Suggest moving summary R^2 values from Tables S4-S8 into the main text to help readers interpret model performance.
L647-649: Can you clarify whether multitrophic abundance/diversity metrics were equally weighted or if trophic groups with missing data were excluded in each case.

Version 1:

Decision Letter:

29th May 2026

Dear Huimin,

Thank you for submitting your revised manuscript "Tree diversity and mycorrhizal type have stronger effects on belowground than aboveground multitrophic biodiversity" (NATECOLEVOL-25093396A). On the basis of the reviewers' comments, we will be happy in principle to publish it in Nature Ecology & Evolution, pending further revisions to satisfy the reviewers' requests and to comply with our editorial and formatting guidelines.

I should note that the reviewers' criticism remain stronger than we anticipated in this round, but the other editors and I decided that they can be addressed with textual revisions without compromising our editorial interest in the study. Regarding Reviewer 3's suggestion to reframe the hypotheses and the conclusions to better reflect the findings, of course we expect that the conclusions match the results but the a priori hypotheses should not be changed at this stage.

We are now performing detailed checks on your paper and will send you a checklist detailing our editorial and formatting requirements in about a week. Please do not upload the final materials and make any revisions until you receive this additional information from us.

[redacted]

***

Reviewer #1 (Remarks to the Author):

The manuscript “Tree diversity and mycorrhizal type have stronger effects on belowground than aboveground multitrophic biodiversity “ is a revised version of a previous manuscript. The authors have provided additional data and removed the speculative sections of the manuscript, thereby improving the quality. I do think the experimental setup has some limitations and is unrealistic. Nonetheless, monitoring the dynamics of above-ground and below-ground organisms simultaneously, as done in this paper, has great value.
In general, I think the writing could still be improved; it is partly cryptic, and the flow within and between paragraphs could be stronger. I make some more specific remarks below.
Line 66: This paragraph switches across trophic levels to mycorrhizal fungi very unexpectedly; perhaps some rewording would improve the flow.
Line 96: “To address these research gaps” and “to test how tree diversity” seem repetitive, revise.
Line 156: stronger effects of what? Improve the writing.
Line 158-159: I suggest being more specific and name also the groups that responded to the tree diversity aboveground.
Line 169-170: “Tree diversity enhanced fungal biomass”- add reference to the results.
Line 171-173: Long and cryptic sentence, revise and be more specific, e.g. observed increases where, compared to what?
Line 174: Be more specific: increased fungal diversity- where? Also, this paragraph is very long; consider splitting it into two paragraphs.
Line 208: “This may explain ” - what may explain?
Line 206-207: has repetitive sentences: “These findings contrast with our expectation (Hypothesis 2) that mixing mycorrhizal types would enhance microorganism and invertebrate abundance and diversity. ” and ” we found no such effects six years after establishing the MyDiv Experiment“
Line 216-218: This sentence seems to fit more in the beginning of the paragraph where the effects are discussed. Now the flow of the paragraph is the effects, the mechanisms and then the effects again.
Line 226: “However, effects on taxonomic richness “ be more specific.
Line 235: What is meant here with “mycorrhizae“? It is a fungus + plant root, and I do not think this is meant here.
Line 292: “positive effects of tree diversity on productivity“- clarify what is meant by productivity, biomass? In this case, I would ask, is the extent of damage by belowground predators sufficient to influence tree growth?
Line 295: “While the interaction between tree diversity and mycorrhizal type had no significant 295 effect on most trophic groups, “ this is already said above and repetitive here, revise.
Line 304: The link with carbon storage seems to stretch these results a bit too far. Can it be supported with evidence?
Line 383: “Our results are most applicable to broadleaf forests. “This should be better integrated within the text.
Line 386: I do not think soil biodiversity is a black box anymore, there is lots of research and knowledge.
Line 391: Where the belowground biodiversity should be prioritized?

Reviewer #2 (Remarks to the Author):

This manuscript addresses an important and timely topic in forest biodiversity research by examining how tree diversity and mycorrhizal type jointly shape multitrophic biodiversity across aboveground and belowground compartments. The study benefits from the well-established MyDiv experiment and an impressive integration of microbial and invertebrate datasets across multiple trophic levels. The revised version has improved in clarity, methodological transparency, and ecological interpretation. Overall, the manuscript has been considerably strengthened after revision, and many of the reviewers' previous concerns have been adequately addressed. However, I still have some concerns.

1. The manuscript successfully integrates tree diversity, mycorrhizal type, and multitrophic biodiversity across aboveground and belowground compartments, which is undoubtedly valuable. However, many of the central ecological ideas underlying the study are already relatively well established, including: biodiversity effects on multitrophic communities, functional differences between AM and EcM systems, and stronger belowground sensitivity to resource heterogeneity and environmental filtering. In this sense, the study primarily represents a comprehensive empirical extension of existing frameworks rather than a major conceptual advance or the development of a substantially new ecological theory. While the scale and integration of the dataset are impressive, the manuscript may not yet provide the level of theoretical innovation or paradigm-shifting insight typically associated with this journal.

2. Although the SEM framework has been substantially improved, many of the proposed mechanisms remain indirectly inferred rather than directly demonstrated. For example, key interpretations involving resource partitioning, trophic propagation, litter heterogeneity, carbon transfer pathways, and bottom-up regulation, are ecologically plausible, but were not directly measured in this study. As a result, several mechanistic conclusions remain largely correlative and conceptual. This limits the strength of the broader ecological inferences that can be drawn from the experiment.

3. A further limitation concerns the generality of the conclusions. The study is based on a single experimental site and relatively young forest stands (~6 years after establishment). Many key ecological processes discussed in the manuscript — including canopy differentiation, mycorrhizal complementarity, litter-mediated feedbacks, and long-term soil food-web restructuring — are likely to strengthen over longer temporal scales and across broader environmental contexts. Consequently, it remains unclear to what extent the observed patterns are generalizable across forest types, biogeographic regions, or mature forest systems. While this limitation is acknowledged in the revised manuscript, it nonetheless reduces the broader predictive and conceptual scope.

Reviewer #3 (Remarks to the Author):

The authors have made substantial revisions, resulting in a much clearer manuscript with improved justification of the experimental design and a more evidence-based interpretation of the results.

My previous comments have been addressed in full. However, on reading the revised manuscript, I think there remains some mismatch between the framing of the mycorrhizal type hypotheses and the strength of the evidence supporting interaction effects

The core finding that tree diversity exerts stronger effects on belowground than aboveground multitrophic biodiversity is now well supported and framed within a coherent mechanistic context. However, the treatment of mycorrhizal type effects remains less well supported, particularly given the absence of interaction effects between tree diversity and mycorrhizal type. This is important as it relates directly to one of the central hypotheses. The manuscript would benefit from more clearly reflecting that these drivers act largely independently (additive rather than synergistic effects). In this context, the title could more clearly reflect the lack of interaction effects, and the abstract should explicitly state that no synergistic effects between tree diversity and mycorrhizal type were detected, to better align the summary with the results. Given that the interaction hypothesis (#3) was not supported, the framing of hypotheses and conclusions could be more closely aligned with the strongest supported findings (main effects and mediation pathways).

More clearly distinguishing which mechanistic pathways are directly supported by the analyses versus those that remain inferential would further strengthen the manuscript. Similarly, interpretations of belowground–aboveground asymmetry should be slightly tempered where they rely on untested mechanisms. For example, explanations based on dispersal limitation and resource stability are plausible but not directly evaluated in the current analysis.

Open Access This Peer Review File is licensed under a Creative Commons Attribution 4.0 International License, which permits use, sharing, adaptation, distribution and reproduction in any medium or format, as long as you give appropriate credit to the original author(s) and the source, provide a link to the Creative Commons license, and indicate if changes were made.
In cases where reviewers are anonymous, credit should be given to 'Anonymous Referee' and the source.
The images or other third party material in this Peer Review File are included in the article's Creative Commons license, unless indicated otherwise in a credit line to the material. If material is not included in the article's Creative Commons license and your intended use is not permitted by statutory regulation or exceeds the permitted use, you will need to obtain permission directly from the copyright holder.
To view a copy of this license, visit https://creativecommons.org/licenses/by/4.0/

Reply to reviewers

We appreciate the time and efforts of the three reviewers, providing positive feedback as well as valuable suggestions to improve our manuscript. We responded to the reviewers' comments in green and the revised text in the manuscript is highlighted in bold font. All line numbers in the response letter refer to the revised manuscript without track changes.

In order to respond properly to one of the reviewer's comments, we cited our colleague's work (Bönisch et al., 2026). This manuscript is still in production at the Journal of Ecology and is expected to be published soon. Due to the limited revision timeline, we would like to submit our revision before its publication. Therefore, in both the response letter and the revised manuscript, we explicitly cited this reference by author name and year, which differs from the remaining references that are cited numerically. If our paper is accepted and published before Bönisch et al. (2026) becomes publicly available, we will request a preprint and add the citation to our publication, as suggested by the editor.

Reviewer #1 (Remarks to the Author):

The manuscript "Tree diversity more strongly promotes belowground than aboveground multitrophic diversity" addresses how the diversity of trees impacts the diversity of other ecosystem components. This is a very interesting question, and the manuscript presents data from an impressive number of organisms. While this is undoubtedly an asset, it also presents a significant challenge for this study: to integrate the dynamics of all these different organisms into a cohesive, impactful narrative. Currently, I believe this task is not being met. In my opinion, the manuscript's writing is not clear and well-justified, as many of the statements seem unclear or speculative.

We worked on our writing to make our article clearer and better justified, and we have put specific emphasis on reducing any speculative statements. Moreover, we addressed reviewers' concerns more specifically below.

1. In addition, I feel this paper is missing one critical aspect of the study and this is understorey information. As understorey plants in Europe mostly form arbuscular mycorrhizas, these plants can play a crucial role in supporting the diversity of both above- and below-ground organisms. Hence, it would modify the effects of tree species in this experiment.

In the MyDiv experiment, understory vegetation was intentionally suppressed using water-permeable weed tarps to minimize competition with tree saplings, resulting in very limited understory plant presence during the study. This allowed us to isolate tree-driven effects of diversity and mycorrhizal type. We have clarified this in the Methods (Lines 483–485): **"Plots are separated by 3 m-wide grass paths, and water-permeable weed tarps were installed between trees to suppress weed growth (see Ferlian et al [52] for details). Understory plants were rare before and during the study period."**

To address your point even more directly, we also added a caveat statement in Lines 380-383**: "The lack of understory vegetation is a strength for isolating tree-driven effects. However, it may limit comparability with observational studies in forests, where understory plants influence resource heterogeneity, microhabitats, and mycorrhizal associations."**

2. In addition, I am not entirely convinced that this setting, where the effect of tree species is explored not in multiple forests but in a relatively recent experimental forest with diversity ranging from 1 to 4, allows for drawing solid conclusions about the role of tree diversity on above- and belowground communities.

Compared to observational studies across multiple forests, controlled field experiments manipulating tree species composition are designed to isolate causal effects of tree diversity while minimizing potential confounding effects by soil, climate, land-use history, and species pools. Observational diversity gradients often conflate diversity with these factors, limiting causal inference. Thus, results from controlled experiments provide foundations to understand the results from observational studies. Therefore, we added an outlook statement for future research in Lines 370-380 **"By using a controlled field experiment, we highlight tree diversity and mycorrhizal types as key drivers of multitrophic biodiversity, primarily governing belowground bottom-up effects, and provide a mechanistic foundation for understanding biodiversity patterns in temperate forests. Notably, tree diversity effects are known to vary with climate and soil fertility, with stronger effects often observed under harsher conditions, such as drier climates or low-fertility soils [44,93]. Replicating comparable experiments across regions and long-term monitoring would therefore help test the generality and temporal development of the observed effects. Observational studies across mature forests can further complement experimental approaches by capturing longer-term and broader environmental contexts, while experiments remain essential for establishing causality [46]."**

We further added this in Lines 479-481 to justify our experimental design. **"The restricted richness range in our study reflects the limited local tree species pool and mirrors typical European forest stands, where more than 80% contain fewer than four tree species [96]."** This also explained why tree-diversity gradients in temperate forest experiments are typically narrower than those in subtropical or grassland biodiversity experiments (e.g., BEF-China).

3. Moreover, all the trees in this experiment are broadleaf (which is great for an experimental setting), but the majority of the EcM trees in Europe are coniferous. This raises another aspect of the limited realism that can be offered in this study. Also, this setting makes links with other studies questionable (eg. statements like "indicating higher leaf N than AM plots (Fig. S1), which is in contrast to earlier findings of higher nutrient concentrations in AM tree leaves")- is the referenced study also comparing only deciduous trees? I am convinced that this difference of broadleaf vs coniferous will have a significant impact on multiple measured aspects and hence it should be carefully explored is the presented aspects are valid.

We agree with the reviewer that this deliberate decision on species selection needed more explanation and added text to justify our experiment design and why we only include broadleaf tree species. It reads (in lines 464-467) as **"The experiment includes only deciduous tree species, despite many ectomycorrhizal-dominated forests in Europe containing multiple conifer species, to avoid introducing additional variation in leaf quality, soil acidification, and fungal communities associated with conifer species."**

We further stressed that our experiment results are directly applied to broadleaf forests, but not all of the European forests, and also added an outlook statement in Lines 383-386: **"Our results are most applicable to broadleaf forests. Because many ectomycorrhizal tree species in European forests are conifers, future experiments that include coniferous EcM species as an additional treatment would broaden the applicability of these findings."**

Thanks for pointing out that differences between our study and previous work may largely reflect the inclusion of coniferous species in earlier studies. Indeed, the referenced studies (Averill et al., 2019; Peng et al., 2022) include both broadleaf and coniferous EcM hosts. To avoid potentially confounded comparisons, we revised the text to rely on evidence from the same experimental site. Specifically, we revised the text (Lines 239-243) to: **"Bönisch et al. (2026) showed that EcM trees at MyDiv exhibited stronger nutrient resorption than AM trees, resulting in lower nitrogen concentrations in EcM leaf litter. Thus, the lower-quality EcM litter accumulates at the soil surface and creates shelters to protect**

invertebrate prey from larger predators, such as beetles and spiders at the soil surface [56].”

We also revised the text in lines 312-314: **“EcM plots had lower leaf C:N, indicating higher leaf N than AM plots (Fig. S6). The N-rich EcM tree leaves may attract predators via enhanced volatile emissions, such as those of linalool and indole [78,81], thereby supporting the highest abundance and diversity of predators.”**

Also, the writing in general could be improved (e.g., hypothesis 3 states “High-tree species richness communities with mixed-mycorrhizal types“, which is grammatically poor).

We have thoroughly revised the manuscript to improve the overall writing. We revised the hypothesis (3) as **“(3) Positive effects of tree species richness on the abundance and diversity of microorganisms and invertebrates are amplified in communities with mixed mycorrhizal types.”**

More specific comments:

Line 51 and 52: It remains unclear why these hypotheses are mentioned in the introduction when they are not included in the hypotheses or discussion part.

To avoid any confusion, we deleted the names of two hypotheses, as testing them was not a primary aim of this study. The text was revised to a brief background statement rather than explicit hypotheses and now reads (lines 56-58): **“The increased total abundance of organisms is more likely to support more taxa [26], and tree mixtures may reduce the dominance of individual species [27,28]. ”**

Line 83: Why was sampling done at the start and the end of the growing season? It is only mentioned in line 133, stating that the growing season has an effect, but this is rather obvious with deciduous plants.

We clarified in the text (lines 99-100) as **“Sampling was conducted at the start and end of the growing seasons, allowing us to capture seasonal dynamics and to identify treatment effects that remained consistent across seasons.”**

Line 56-57: “bottom-up” effects are explained, but “top down” effects are not.

We have revised the text to clarify top-down effects (lines 182–183). It now reads: **“Meanwhile, their populations were likely suppressed by the increased abundance of predators (top-down effects) [29].”**

Line 106: what was measured?

We measured invertebrates separated into trophic groups across three vertical compartments (aboveground, soil surface, and belowground). For microorganisms, we assessed foliar fungi, soil fungi, and soil bacteria. These details are provided in the Methods section, but to improve clarity given that results precede methods, we have now made this explicit in the Results (lines 121-125): **“Tree diversity (i.e., tree species richness) significantly increased the abundance of belowground invertebrates and microorganisms across trophic levels (Fig. 1E; Tables S7-8). However, the abundance of aboveground and soil-surface trophic groups showed only limited responses, with significant effects restricted to total foliar fungi ($F_{1,22}$ = 4.6, p = 0.04; Fig. 1A; Table S5) and soil-surface omnivores ($F_{1,54}$ = 4.52, p = 0.04, Fig. 1C; Table S6).”**

Line 114: Comparisons with grasslands are made, while this study excludes herbaceous plants, I do not think this is correct.

We have revised the Results and Discussion by removing comparisons with herbaceous plants and instead focusing on our expectations and hypotheses. To our knowledge, no previous studies have investigated tree diversity effects across trophic levels spanning both above- and belowground food webs. We therefore emphasize the underlying reasons and

mechanisms, as suggested by Reviewer 2. The revised text (lines 138-155) reads as**: "The results likely reflect differences in dispersal limitation and resource stability between above- and belowground systems. Increased aboveground productivity at higher tree diversity [19] likely leads to greater carbon inputs belowground through root growth [53], rhizodeposition [54,55], and mycorrhizal associations [22,36], thereby enhancing resource availability for belowground communities. Tree diversity probably also increased belowground resource heterogeneity via these pathways [56]. Belowground resources are often spatially structured, and soil organisms typically exhibit lower mobility than organisms in higher strata, making them more strongly influenced by local resource availability and plot-level environmental conditions [57]. This localized resource dependence could lead to stronger bottom-up effects [58], promoting consumer abundance and diversity at higher trophic levels. In contrast, aboveground organisms often exhibit stronger host specialization (e.g., herbivores) and greater mobility (e.g., predators), making consumer community structure more dependent on tree species identity or functional traits rather than plot-level conditions [59,60]. In addition, tree diversity-driven differences in resource inputs to soils may accumulate over time [61], leading to stronger biodiversity responses belowground than in higher strata, where communities are more strongly influenced by seasonal dynamics (Tables S4-S6). Notably, field samples taken at or near canopy closure (in May and September) may underestimate aboveground effects, as reduced variation in light and microclimate can dampen tree diversity responses."**

Line 126-128 "The increase in predator richness contributed to the significant rise in taxonomic richness and Shannon-Wiener diversity of the total aboveground invertebrate communities (Fig. 1; Fig. S4)." It is confusing as Fig. 1 presents abundances, and it is difficult to follow what this sentence is based on.

We apologize for this mistake. Moreover, as suggested by Reviewer 3, we applied a stricter significance threshold of $p < 0.05$ only. The overall trends remained similar and are shown in Figs. 1–2, but the revised results indicated that tree diversity did not significantly increase aboveground total taxonomic richness ($F_{1,54} = 3.8$, p = 0.06; Fig. 2A; Table S4). After carefully rechecking the analysis, the *p*-value for the effect of tree diversity on aboveground predator richness was not even smaller than 0.1 ($F_{1,54} = 2.4$, p = 0.15; Fig. 2A; Table S4). Therefore, we removed the original discussion of the effect from the revised manuscript. We now focus more on the ecological mechanisms leading to the different responses of above- and belowground communities, as indicated above.

Line 145-147: "Tree diversity enhanced fungal biomass, likely promoting saprotrophic fungi and, in turn, supporting higher abundance of nematode fungivores. " This speculation of increasing saprotrophic fungi could be tested, for example, using a database such as FungalTraits to assign functional groups to fungal sequences and including those in the analyses. Also, I do not see the data supporting the increasing abundance of nematode fungivores.

We calculated diversity indices (taxonomic richness - Chao1, and Shannon diversity) for different functional groups, including saprotrophic, pathogenic, mycorrhizal, arbuscular mycorrhizal, and ectomycorrhizal fungi (Fig. S5). Tree diversity significantly increased diversity across all groups, including saprotrophic fungi. However, as we did not directly measure saprotrophic fungal biomass, we have clarified the text (lines 169-170), which now reads: **"Tree diversity enhanced fungal biomass that serves as the food resource for nematode fungivores."**

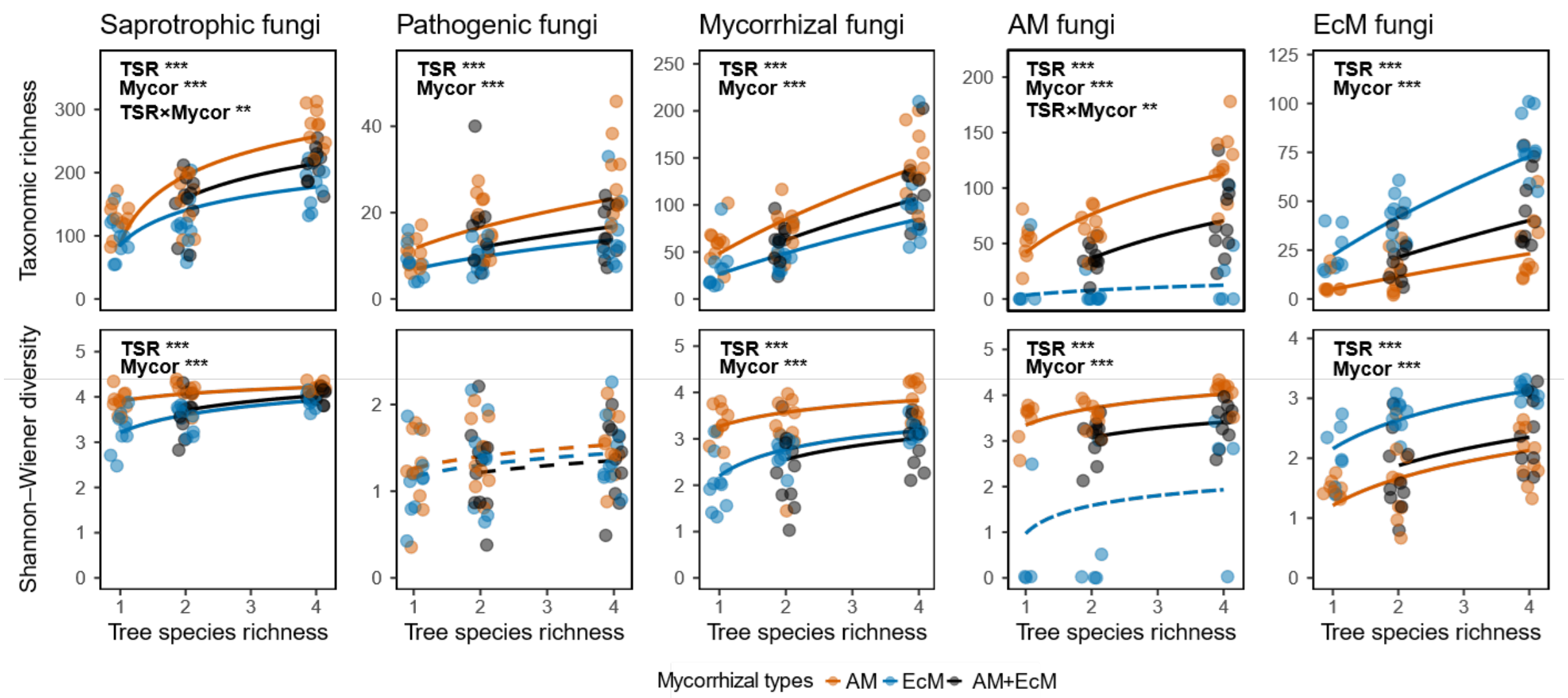

Figure S5: Effects of tree species richness (TSR), mycorrhizal type (Mycor), and their interaction on the taxonomic richness and Shannon-Wiener diversity of fungal functional groups. The significance of fixed effects (TSR, Mycor, and TSR × Mycor) is indicated within each panel. Significance levels are indicated as *** for $p < 0.001$, ** for $0.001 \le p < 0.01$, and * for $0.01 \le p < 0.05$. Effects with $p \ge 0.05$ are not labeled for visual clarity. Solid lines indicate significant effects of tree species richness, whereas dashed lines indicate non-significant effects. Points represent observed values, and lines show model-predicted responses from linear mixed-effects models fitted as: lme (y_i^t ~ Mycor × log(Richness), random = ~1 | composition, data). Fungal functional groups are labeled at the top of each column; taxonomic richness (Chao1; upper row) and Shannon–Wiener diversity (lower row) are shown separately.

Line 211: "The greater abundance of herbivorous nematodes, together with higher absorptive root turnover, may also explain why " I do not see where the absorptive root turnover is measured, hence this seems speculative. Moreover, as pointed out earlier, I think this can additionally be influenced by the herbaceous vegetation which is not incorporated to the analyses.

We agree that this statement was speculative, as absorptive root turnover was not measured in this study. We therefore deleted the sentence from the manuscript. To retain the conceptual relevance, we added a brief outlook statement. It reads as in lines 359-363: **"Despite including canopy, root, and soil nutrient mediators, we were not able to account for fine-root growth and turnover, which are key resource inputs to soil food webs and differ between AM and EcM communities [91,92]. Incorporating these processes in future studies would help clarify mechanisms underlying AM–EcM differences in soil food-web structure [53]."**

Understory vegetation could influence belowground communities. In this experiment, understory plants were absent due to the use of weed tarp. We clarified this in the Methods (lines 483-485): **"Plots are separated by 3 m-wide grass paths, and water-permeable weed tarps were installed between trees to suppress weed growth (see Ferlian et al [52] for details). Understory plants were rare before and during the study period."**

Line 216-217: "low quality of leaf litter from EcM trees to have accumulated on the soil surface" Is the low quality of ECM litter demonstrated specifically on decidous EcM trees, which are incorporated to this study?

We removed comparisons with previous studies and now rely exclusively on evidence from the same experimental site. Bönisch et al. (2026), working within MyDiv and using the same deciduous tree species, showed that EcM trees exhibit stronger nutrient resorption than AM trees, resulting in lower-quality (higher C:N) leaf litter. The current text (lines 239-243) reads

as **"Bönisch et al. (2026) showed that EcM trees at MyDiv exhibited stronger nutrient resorption than AM trees, resulting in lower nitrogen concentrations in EcM leaf litter. Thus, the lower-quality EcM litter accumulates at the soil surface and creates shelters to protect invertebrate prey from larger predators, such as beetles and spiders at the soil surface [56]."**

Line 220-221: This writing is misleading: "diversity of predators than AM and AM+EcM communities (Figs. 1B & 2B)." As statistical difference was only between ECM and AM (not AM+ECM).

Thanks for pointing out the misleading text. We revised the text (lines 248-250). Now, it reads **"Mycorrhizal type effects also extended to the aboveground organisms: EcM tree communities supported higher abundances ($F_{2,54}$ = 3.8, p = 0.029) and diversity ($F_{2,54}$ = 4.0, p = 0.025) of predators than AM communities (Figs. 1B & 2B)."**

Line 222: Talking abvout longer lifespan of trees in a 6 years experiment seems inappropriate.

We removed lifespan-related interpretations and revised the text to focus on structural and phenological differences that are relevant at this stage. The revised text now reads **"EcM tree species, e.g., oak and beech trees, with dense branch systems and later leaf senescence, provide greater and more stable habitats for predators, favoring both their abundance and diversity (Fig. 1B) [77]."**

Line 506-507: Phospholipid fatty acid content is not suitable method for estimating microbial community composition.

In our context, phospholipid fatty acid content was used to quantify the fungal to bacteria biomass ratio. We clarified the respective text in lines 568-572 **"The phospholipid fatty acid (PLFA; ng/g dry soil) contents of soil samples were used to estimate the biomass ratio between fungi and bacteria ($F{:}B$) [122–124]. More specifically, the ratio was derived from the sum of the PLFA markers 18:2ω6,9 and 16:1ω5 (neutral lipid fatty acid [NLFA]) for fungi and the sum of the PLFAs i15:0, a15:0, i16:0, and i17:0, cy17:0, and cy19:0 for bacteria [124–126]."**

Line 529: Information about bioinformatic analyses is missing.

We added more details about bioinformatic analyses (lines 587-598): **"The raw reads were processed using QIIME 2 (v2022.2) [131] as described in Ul Haq et al [127] . Briefly, primers were trimmed from the Illumina MiSeq paired-end raw reads, where the forward and reverse reads were demultiplexed based on their respective index combinations through the Illumina standard procedure, using the cutadapt function (q2-cutadapt plugin). Subsequently, the trimmed sequences were denoised, merged, cleaned from chimeric reads, and grouped into Amplicon Sequence Variants (ASVs) using DADA2 denoise-paired algorithm (via q2-dada2 plugin). Taxonomic assignment was done using the SILVA 132-99-515-806 naïve Bayesian classifier reference database [132] for bacteria and the UNITE (v9) dynamic classifier reference database for fungi [107]. The respective fungal and bacterial ASV matrices, taxonomic tables, and representative sequences were filtered to remove non-target taxa. Those ASVs assigned at least at the phylum level were retained. The per-sample read coverage was normalized to the minimum number of reads in both datasets."**

Supplementary analyses line 146-148: Lines were predicted based on the model: lme ($yi \sim month + Mycor * log(Richness)$), ~1| composition/month, data), with values averaged from both sampling seasons. I do not understand the analyses, why is month included when values are averaged from both sampling seasons?

We corrected the text as follows (lines 304-306 in Supplementary Information): **"Lines were predicted from the model *lme*(y_i^t ~ Month + Mycor × log(Richness), random = ~1 |**

composition/Month, data). For visualization, predictions were averaged across sampling seasons using the *emmeans* package."

Also, I find it highly disturbing that the manuscript does not present the number of fungal and bacterial richness (including ECM and AM fungal richness), to allow the reader to see if these conclusions can be made based on this data (for example higher AM fungal richness and abundance in plots with AM trees is a basic assumption that has to be met to justify any following analyses.

We present bacterial diversity, fungal diversity, and the diversity of different fungal functional groups in Figs. S4 (see below) and Fig. S5 (see the answer above). Arbuscular mycorrhizal (AM) fungal diversity was significantly higher in AM tree communities than in EcM communities, and AM fungi were largely absent from most EcM communities. In contrast, ectomycorrhizal (EcM) fungal diversity was significantly higher in EcM tree communities than in AM communities.

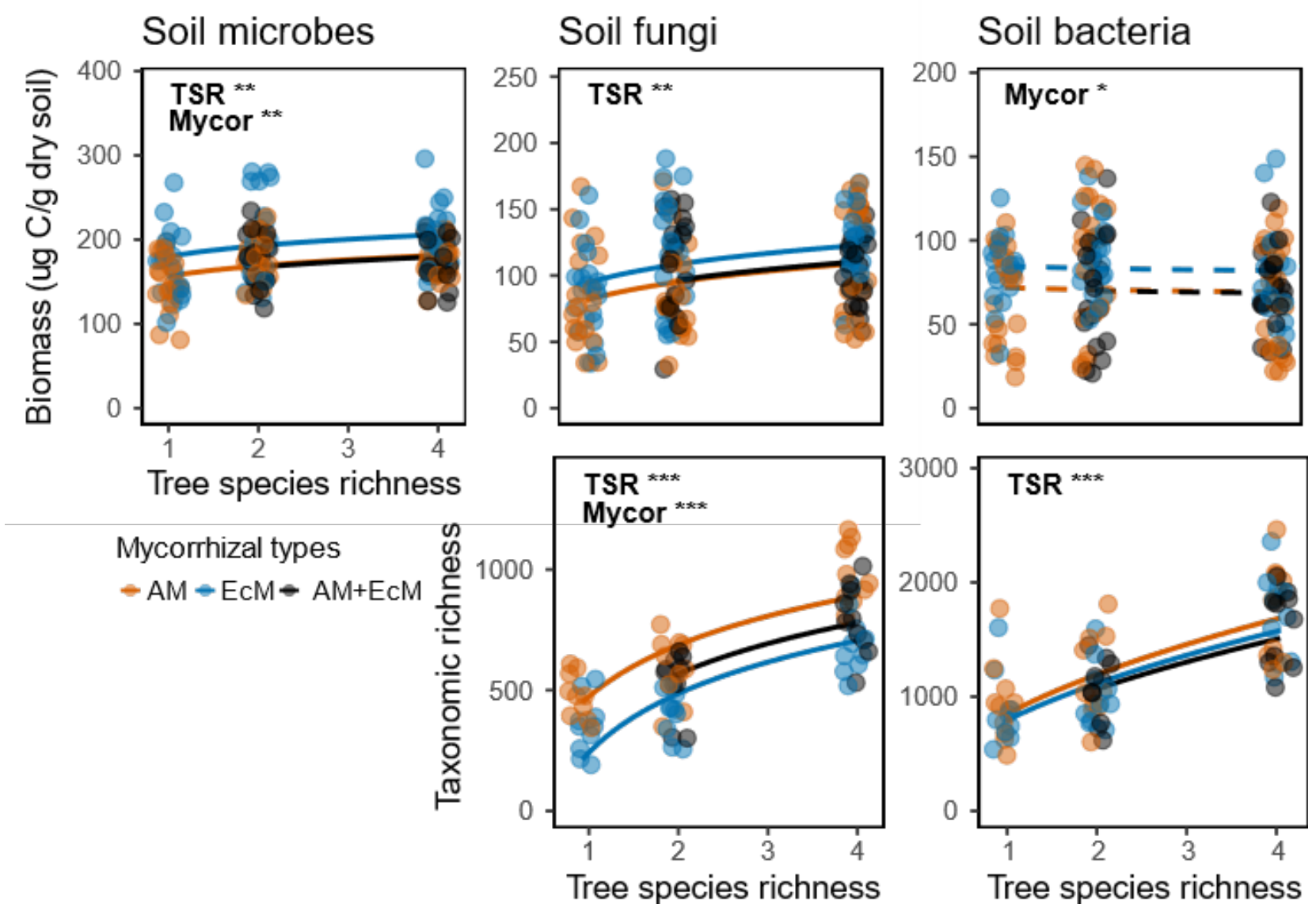

Figure S4 (continued): Effects of tree species richness (TSR), mycorrhizal type (Mycor), and their interaction on abundance and taxonomic richness of soil microbial communities in the belowground compartment. Trophic groups are labeled at the top of each column. The lower rows indicate microbial biomass (as a proxy for abundance) and estimated taxonomic richness (Chao1).

The data presented in the supplementary material is based not on the raw values but on some groupings (omnivores etc.) and often represent min-max transformed values, which is not helpful for future research.

We will make the untransformed biodiversity metrics (abundance, taxonomic richness, and Shannon diversity) for each trophic group and food-web compartment at the plot level publicly available upon acceptance (see in lines 885-883). It reads now **"The datasets and R-script used in this study will be publicly available in the MyDiv database (https://mydivdata.idiv.de) upon acceptance. The specific datasets are as follows: dataset ID 274 contains biodiversity metrics for trophic groups across food-web compartments at the plot level. Supplementary Data 1 and all R scripts are included in dataset ID 276."** We will also provide the trophic grouping table as a supplementary file (i.e., Supplementary Data 1) to ensure transparency and facilitate reuse. In addition, we reran the models using the original data **(Figs. S2–S5 & Fig. S13)**, which showed consistent significance levels and treatment effect magnitudes compared with the min–max transformed data.

Reviewer #2 (Remarks to the Author):

This manuscript addresses a relevant and timely question in forest biodiversity research—how tree diversity and mycorrhizal associations jointly influence multi-trophic biodiversity across aboveground and belowground compartments. The authors used a large-scale tree-mycorrhizal diversity experiment (MyDiv) and integrated extensive datasets spanning microbial and invertebrate communities. However, while the manuscript presents some interesting findings, several major issues need to be addressed. My comments are outlined below.

We thank Reviewer 2 for the thorough review and constructive comments on our manuscript. All concerns had been addressed point by point below, and corresponding revisions were made in the manuscript.

1. Conceptual framework and hypotheses

•The study sets out four hypotheses (lines 92–101), yet the logical connections among them and the underlying ecological rationale could be clarified. For instance, Hypothesis (2) assumes stronger mycorrhizal effects belowground, while Hypothesis (3) assumes synergistic effects of AM+EcM mixing—but the mechanistic distinction between "additive" and "synergistic" outcomes is not clearly defined. Please revise the Introduction to more explicitly articulate how each hypothesis arises from existing theory (e.g., resource partitioning, functional complementarity, or the mycorrhizal-associated nutrient economy framework).

We revised the Introduction to strengthen the logical connection leading to Hypothesis (2). The relevant theoretical concepts are now incorporated to motivate the hypotheses, while avoiding explicit naming of all ecological theories, in line with Reviewer 1's suggestion to remove theory labels that are not directly tested. Instead, these concepts are used as background rationale to derive clear, testable expectations.

In addition, we now explicitly define what is meant by synergistic effects in the context of this study, thereby clarifying the distinction between additive and non-additive outcomes as requested (in lines 70-72). The revised text (lines 66–86) reads as follows:

"Symbiosis with arbuscular (AM) and ectomycorrhizal (EcM) fungi influence host tree soil nutrient cycling and life strategies and, in turn, shape associated microbial and invertebrate communities [22,32–35]. AM fungi enhance uptake of inorganic nutrients from mineral soil layers, whereas EcM fungi secrete extracellular enzymes to access organic nutrient pools in top soil layer [22,36]. Complementary resource-use strategies may partition soil nutrients and space, such that mixed AM and EcM (AM+EcM) forests can be more productive than single-type forests (i.e., synergistic effects) [37]. Therefore, synergistic effects exceed additive effects of mixing different mycorrhizal types. This elevated productivity may in turn support higher consumer abundances, particularly among generalist taxa in soil food webs [38]. Moreover, AM trees generate nutrient-rich, fast-decomposing litter that supports bacteria and bacterivores, whereas EcM litter favors fungi, fungivores, and meso-detritivores [32–34], and EcM roots experience reduced herbivory due to protection by the fungal mantle [35]. Together, these contrasts indicate that mycorrhizal type may structure soil food webs through altering multiple energy channels. Such differences may also extend aboveground, as AM trees typically prioritize growth over defense and experience higher herbivore pressure than EcM trees [32,39]. Consequently, mycorrhizal mixtures may also generate synergistic effects on the diversity of both above- and belowground communities by broadening the trophic niches [24,40]. Because mycorrhizal-mediated processes act primarily in soils, synergistic effects are expected to be strongest belowground and at lower trophic levels. As climate warming, drought, and anthropogenic pressures shift mycorrhizal dominance in forests [41–43], empirical tests are urgently needed to predict the biodiversity responses across trophic levels."

Regarding the rationale leading to hypothesis (3) we clarified it as (lines 87-95):

"Increasing evidence shows that tree diversity effects on ecosystem functioning are amplified by functional trait differentiation among tree species [44–47]. Accordingly, tree diversity effects are expected to be stronger in mixed mycorrhizal communities than in stands dominated by a single mycorrhizal type. However, empirical support for interactive effects between tree diversity and mycorrhizal types remains largely confined to tree productivity [48]. Beyond the tree community, existing evidence has mainly focused on individual taxonomic groups in isolation (e.g., microbes [49,50] and nematodes [51]). Therefore, an integrated multitrophic approach is required to disentangle the effects of mycorrhizal types and their interactions with tree diversity across the entire food web."

The hypothesis (3) was revised as (lines 114-116) **"(3) Positive effects of tree species richness on the abundance and diversity of microorganisms and invertebrates are amplified in communities with mixed mycorrhizal types."**

•The authors may also consider clarifying how the current study advances beyond prior MyDiv publications (e.g., Ferlian et al. 2018, Eisenhauer et al. 2023) that have examined similar belowground patterns. The novelty of integrating multi-trophic levels should be highlighted more explicitly.

We have clarified the novelty of integrating multi-trophic levels more explicitly in the manuscript (lines 47–65): **"Biodiversity is structured into food webs of interacting trophic groups, and understanding the diversity across trophic levels provides insights into community structure and ecosystem functioning beyond taxonomic patterns [16,17]. Within this framework, changes in tree communities can propagate to higher trophic levels along the food chain (bottom-up effects) [18]. More specifically, tree mixtures can increase canopy complexity and productivity through spatial and resource partitioning [19,20], benefiting aboveground herbivore abundance [21]. Meanwhile, greater allocation of photosynthates to roots promotes root growth, mycorrhizal colonization, and exudation [22,23], thereby increasing the abundance of belowground herbivores and microorganisms [10,24]. Moreover, plant communities shape soil nutrient cycling, which structures soil microbial and nematode assemblages [24,25]. The increased total abundance of organisms is more likely to support more taxa [26], and tree mixtures may reduce the dominance of individual species [27,28]. These cascading effects ultimately influence populations of microbial feeders and predators[18,21]. Thus, lower trophic levels may respond more strongly than higher trophic levels [18,29], while more mobile aboveground organisms may react faster to local changes in plant diversity than belowground biota, such as shown for grasslands [18]. Therefore, investigating the responses across trophic levels provides a comprehensive perspective and mechanistic insights into how plant diversity shapes above- and belowground communities [30,31]. Yet, these links remain insufficiently tested in forests [10,15]. "**

We edited the text in lines 90–95 to justify the novelty beyond previous MyDiv research. It reads: **"However, empirical support for interactive effects between tree diversity and mycorrhizal types remains largely confined to tree productivity [48]. Beyond the tree community, existing evidence has mainly focused on individual taxonomic groups in isolation (e.g., microbes [49,50] and nematodes [51]). Therefore, an integrated multitrophic approach is required to disentangle the effects of mycorrhizal types and their interactions with tree diversity across the entire food web."**

2. Statistical and analytical methods

•The power-law framework used to model tree diversity effects (Fig. 1A, C, E) should be explained in greater detail in the Methods. Specifically, please clarify (1) why a power function was selected over other diversity–abundance models, (2) how scaling to [0,1]

affects effect size interpretation, and (3) whether slopes were compared statistically among compartments.

One small correction before addressing the reviewer's questions. Strictly speaking, we did not use a power-law framework. We apologize for this mistake.

For question (1) why a power function was selected over other diversity–abundance models.

We corrected this in lines 754–755 as follows: **"We used mixed-effects models to analyze the effects of tree diversity and mycorrhizal types on biodiversity indices."** We explained why a power transformation (y_i^t) was applied, as the following text (lines 763–769): **"The reason why we used power transformation rather than the log-transformation log(y_i + a) was because the latter one would overly suppress smaller values in y_i that was scaled to the range [0,1] beforehand [156], and also not applicable for taxonomic richness and Shannon-Wiener diversity that either followed normal or left-skewed distribution. The power transformation y_i^t could effectively handle right-skewed abundance data (with t<1) and left-skewed Shannon-Wiener indices (with t>1) [157].".** Moreover, we added an additional sentence to further justify this decision in line 769-772. The revised text now reads: **"Therefore, the same mixed-effects modeling framework allowed us to accommodate a broad range of linear and non-linear responses across trophic levels, food-web compartments, and biodiversity indices. In practice, this also streamlines the workflow and improves the reproducibility of our analyses."**

For question (2) how scaling to [0,1] affects effect size interpretation:

"The same analyses were also performed on the original data (before min–max transformation). The results are presented in the Supplementary Information (see Fig. S2–S5). The statistical significance of treatment effects, as well as the overall trends observed in the min–max transformed data, were consistent with those obtained from the original data. Importantly, the transformation facilitated comparisons of treatment effects in the figures, including the magnitude of increases along the tree diversity gradient and differences between mycorrhizal types [18]." The edited text can be found in lines 787-793 and results based on the original data found in Figs. S2–S5.

For question (3) whether slopes were compared statistically among compartments:

"Because group-specific power transformations were applied, the coefficients of $log(Richness)$ could not be directly compared to determine which groups exhibited stronger tree diversity effects across the three strata. To facilitate statistical comparisons of tree diversity effects across trophic groups and food-web compartments, we extracted model predictions (mean ± 95% confidence interval) at three levels of species richness (1, 2, 4) using the *emmeans* package. The log-response ratios of predicted values for 2 vs. 1 and 4 vs. 1 tree species were calculated as standardized effect sizes to quantify the magnitude of tree diversity effects across trophic groups and food-web compartments. In addition, the log-response ratios were calculated to assess the magnitude and robustness of tree diversity effects beyond significance levels. The log-response ratios were calculated using both the min–max transformed data and the original values. For most trophic groups, the results were consistent between the two approaches, confirming the robustness of our findings (Fig. S11 & S13). The only exception was soil fungal and bacterial taxonomic diversity, where the log-response ratios based on the original data were slightly lower than those derived from the transformed values." Please find the text in lines 794–808. The figures for effect sizes are shown below and can now be found in the supplementary information Figures S11-S13.

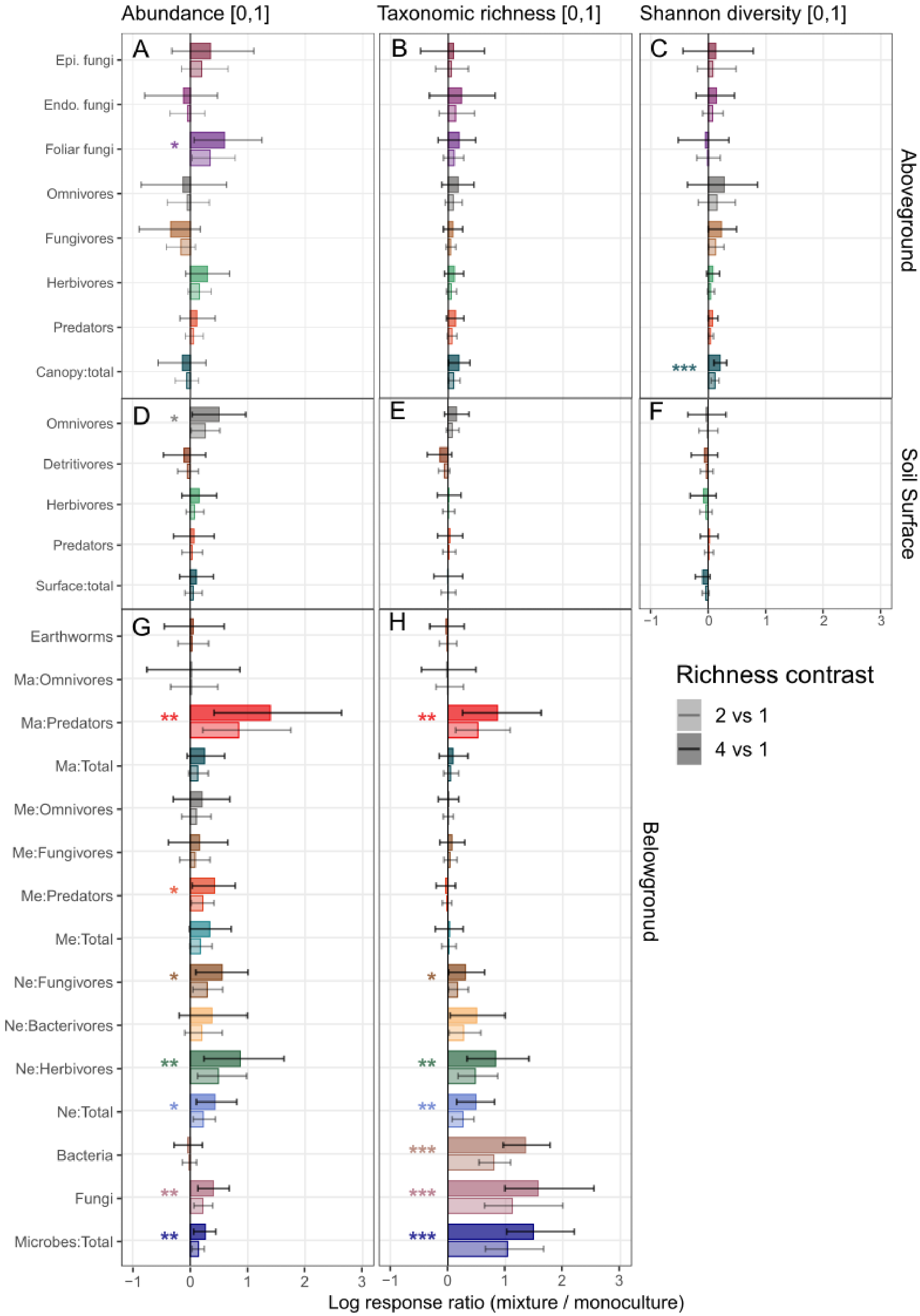

Figure S11: Standardized tree diversity effect sizes on the abundance (A, D, G), taxonomic richness (B, E, H), and Shannon–Wiener diversity (C, F) across trophic groups and food-web compartments (Aboveground, Soil surface, and Belowground).

Effect sizes were calculated from predicted values for monocultures and tree mixtures (2- and 4-species, with different transparency) that were obtained from linear mixed-effects models (*lme*: yi^t ~ Month + Mycor + log(Richness)) fitted to min–max transformed data.

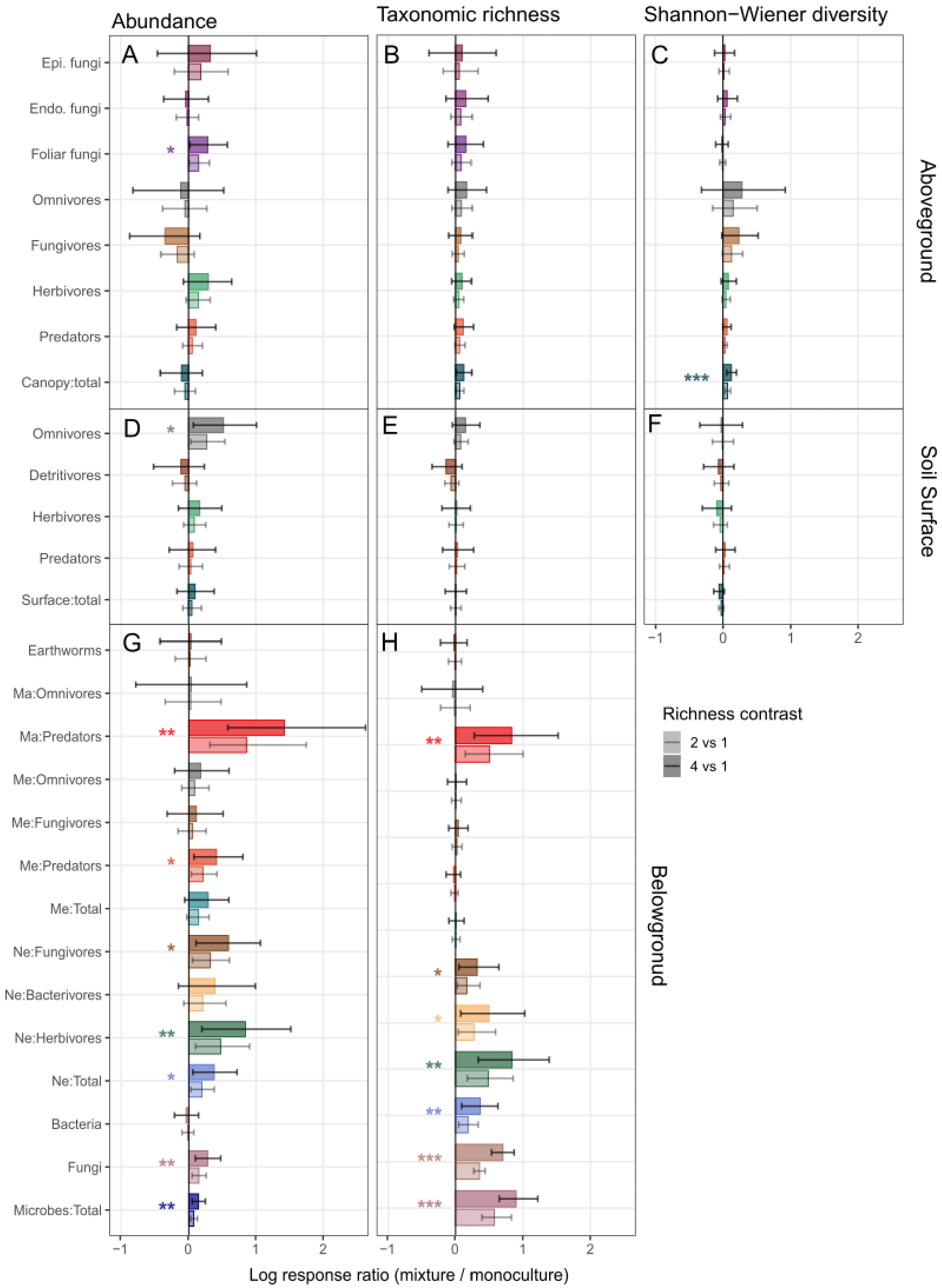

Figure S13. Standardized tree diversity effect sizes on the abundance (A, D, G), taxonomic richness (B, E, H), and Shannon–Wiener diversity (C, F) across trophic

groups and food-web compartments. This figure follows the same structure and pattern as Fig. S10, but is based on predictions from models fitted to the original data.

•The structural equation model (SEM) is central to the paper but requires additional transparency: (1) Provide a supplementary figure or table describing the ecological rationale and evidence supporting each hypothesized path in the initial SEM. 2) Explain how multicollinearity among mediators (e.g., ENL, leaf C:N, root Ca) was handled. 3) Present total effects (direct + indirect) with confidence intervals and clarify how model selection affected path retention.

For (1) Provide a supplementary figure or table describing the ecological rationale and evidence supporting each hypothesized path in the initial SEM.

In the revised version of the manuscript, we followed the reviewer's advice and developed a conceptual figure and provided the ecological rationale for each pathway together with supporting literature. Please see the response below or refer to the Supplementary Information (in lines 131-192). We indicated this change also in the main text in lines 600-606. It reads as **"We hypothesized that tree diversity and mycorrhizal type influence above- and belowground communities indirectly through changes in aboveground tree stand characteristics (e.g., canopy structure complexity [19,133], productivity [5,63], and leaf quality [86]). Moreover, soil properties [86,134] and roots nutrient contents [135–137] may mediate the effects of tree diversity and mycorrhizal type on both soil surface and belowground communities. Details of the hypothesized framework and the supporting literature are shown in Fig. S1, and all mediating parameters measured in this study are described below."** Here, we show the conceptual figures to explain why we measured many more mediating variables than what we include in Figs 3 & 4. For the final SEM framework in Fig. S7, we only included mediators that were significantly affected by the treatments see Table S9, Fig. S6) and were not strongly correlated with each other (Table S10) (details in lines 823-843**.**

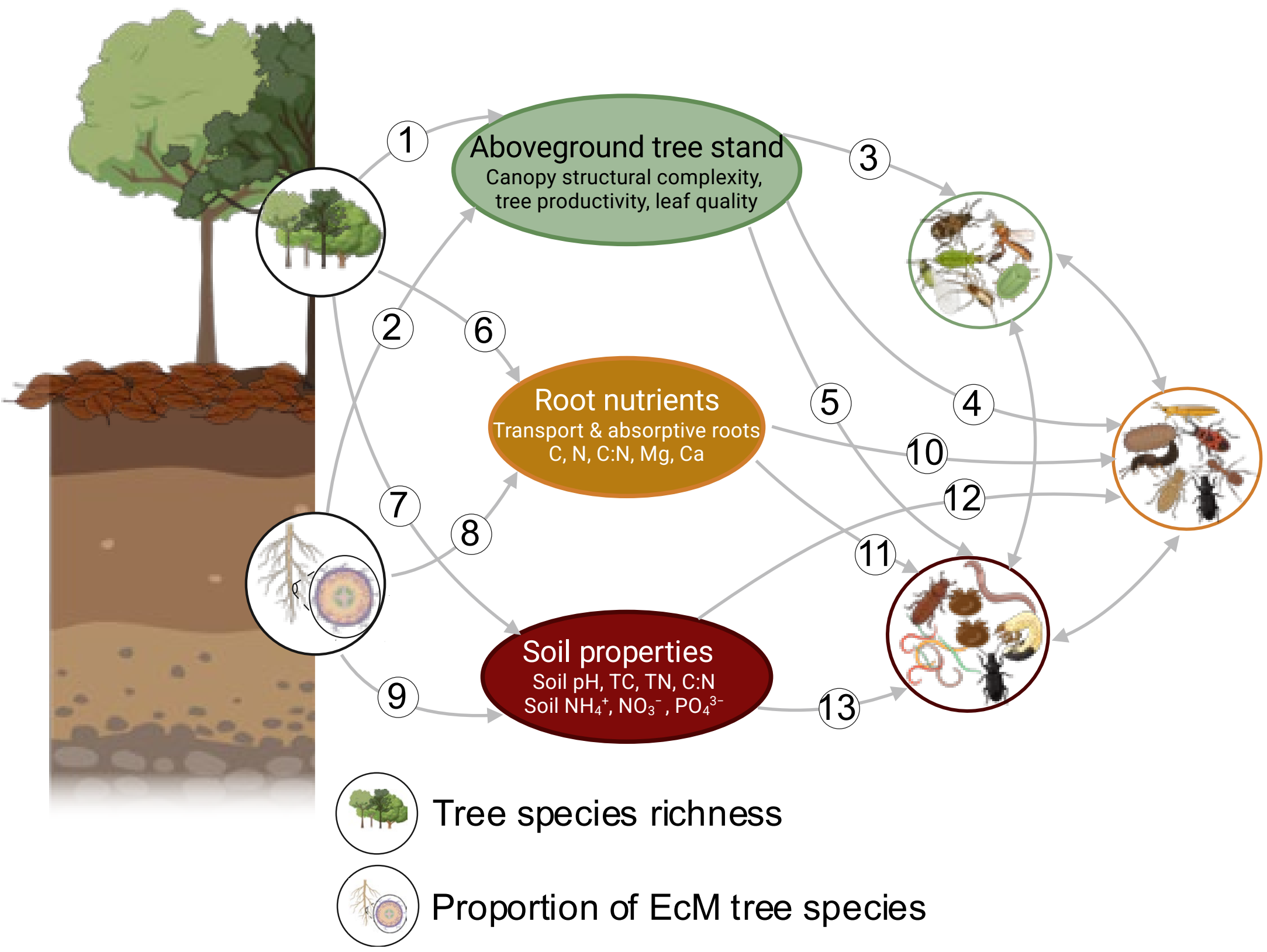

Figure S1: Conceptual figure of the hypothesized structural equation model (SEM) framework for tree and mycorrhizal type effects as well as environmental mediators on multitrophic abundance and diversity across above- and belowground food webs. The invertebrates across the aboveground (green bubbles), soil surface (orange bubbles), and belowground (brown bubbles) strata are displayed on the right side of the framework. Arrows indicate proposed causal relationships. We tested how tree species richness and mycorrhizal types (the proportion of EcM tree species; EcM%) influence multitrophic metrics via a range of mediators representing the aboveground tree stand, root nutrients, and soil properties. Mycorrhizal type was coded as the proportion of EcM tree species (1 = EcM, 0.5 = AM+EcM, 0 = AM). As this is a conceptual figure, the final analysis included only mediators that were significantly affected by the treatment factors (see Table S9, Fig. S6) and were not strongly correlated with each other (Table S10). The theoretical basis for each pathway (indicated with number in the cycles) is described below. Created in BioRender. Eisenhauer, N. (2025) https://BioRender.com/jvnwoo.

Pathways 1-2: tree diversity and mycorrhizal types → aboveground tree stand characteristics

We hypothesized that tree diversity influences above- and belowground communities indirectly through changes in canopy structure complexity [34,35], productivity [36,37], and leaf quality [38]. In addition, we assumed that these variables mediate the effects of mycorrhizal type [39–42]. Specifically, we assumed that tree canopy structural complexity (measured as the effective number of layers, ENL), aboveground tree productivity (total wood volume), and leaf quality (leaf nitrogen content and C:N ratio) act as key mediators linking tree diversity to biotic communities.

Pathway 3: aboveground tree stand characteristics → aboveground biodiversity

For aboveground communities, we expected canopy structural complexity to increase habitat heterogeneity and resource availability, thereby promoting the abundance and diversity of foliar fungi [15] and invertebrates [43,44]. Higher tree productivity was expected to increase the overall availability of plant-derived resources [45], while leaf quality was assumed to influence herbivore and foliar fungal communities through changes in leaf nutrient content and palatability [46–48]. These effects were expected to propagate to higher trophic levels such as predators [7].

Pathway 4-5: aboveground tree stand characteristics → soil surface and belowground biodiversity

For soil-surface and belowground communities, we hypothesized that canopy structural complexity enhances microclimatic stability (e.g., temperature buffering and soil moisture) [49,50], which benefits invertebrates and microbial communities. In addition, higher aboveground productivity was expected to increase carbon inputs to the soil through root growth, root exudates, and litter production, thereby supporting microbial communities, detritivores, and higher trophic levels [51–55]. Finally, leaf quality was expected to indicate litter quality and decomposition processes, affecting resource availability for microbial communities [41] and microhabitats for litter and soil fauna [56,57].

Pathway 6-9: tree diversity and mycorrhizal types → soil properties and root nutrients

For soil-surface and belowground communities, we hypothesized that the effects of tree diversity are mediated by soil properties [38,58] and root nutrient contents [59–61]. These variables are also expected to mediate the effects of mycorrhizal type [41,62–65]. Specifically, we assume that soil properties (soil pH, soil total C and N contents, and C:N ratio), soil nutrient availability (NO_3^-, NH_4^+, and PO_4^{3-}), and nutrient contents in both transport and absorptive roots (e.g., C, N, Ca, Mg, and root C:N ratio) act as key mediators linking tree diversity and mycorrhizal types to soil-surface and belowground communities.

Pathway 10-11: root nutrients → belowground and soil-surface biodiversity

Root nutrient contents can determine the palatability of roots for root-feeding organisms [66]. Following root turnover, nutrients contained in root litter further regulate the abundance of detritivores, such as earthworms [67,68], and influence soil microbial community structure. These effects can propagate through the entire soil food web [69,70]. In addition, roots, particularly those of ectomycorrhizal (EcM) trees, often forage for nutrients in the top-soil and litter layer, which may also influence soil-surface communities [71].

Pathway 12-13: soil properties → belowground and soil surface biodiversity

Soil properties directly determine the structure of soil microbial communities and nematode assemblages [41,72–74]. For example, higher nutrient availability was expected to promote soil bacteria and bacterivorous nematodes, whereas higher soil organic matter and carbon contents are likely to favor soil fungi and fungivorous nematodes [72,75,76]. Soil carbon content and the soil C:N ratio have also been shown to be key predictors of microbial biomass [77] and oribatid mite diversity [78].

For comment 2) Explain how multicollinearity among mediators (e.g., ENL, leaf C:N, root Ca) was handled.

The final SEMs only include the parameters that did not significantly correlate with each other, see Table S10. The details are now presented in the Methods section in lines 832–843:, **"We assessed collinearity among nine potential mediators influenced by the experimental treatments to simplify the model and reduce the multicollinearity (Table S10; Pearson's $r < 0.40$) [160]. Wood volume and ENL were strongly positively correlated ($r = 0.73$); because ENL captures both aboveground productivity and canopy complexity, we retained ENL and excluded wood volume (Table S10) [19]. Leaf C was strongly positively correlated with leaf N ($r = 0.52$), and leaf N was significantly negatively correlated with C:N ($r = -0.97$); we retained C:N as a single indicator of leaf nutrient quality (Table S10) [86]. Root Mg and Ca were strongly correlated ($r = -0.62$); we used root Ca to represent root nutrient content [88], alongside root C. Soil TC and TN were positively correlated ($r = 0.74$); we retained TC, while soil nitrogen status was represented by soil NO_3^-, which is a powerful predictor of soil nutrient availability (Table S10) [86]. "**

Table S10: Pearson's correlations between mediators influenced by the treatment factors (tree species richness and/or mycorrhizal types).

Variables	*Wood volume*	*ENL*	*Leaf C*	*Leaf N*	*Leaf C:N*	*Root C*	*Root Mg*	*Root Ca*	*Soil TC*	*Soil TN*
ENL	**0.73*** **									
Leaf C	**-0.29** **	-0.06								
Leaf N	-0.17	-0.16	**0.52*** **							
Leaf C:N	0.12	0.15	**-0.38*** **	**-0.97*** **						
Root C	0.14	*0.2*	-0.1	-0.14	0.08					
Root Mg	0.03	0.08	**-0.24***	-0.18	0.12	-0.1				
Root Ca	0.07	0.05	**0.22***	*0.19*	-0.14	-0.11	**-0.62*** **			
Soil TC	0.09	0	-0.15	-0.13	0.09	-0.06	**0.29** **	-0.01		
Soil TN	0.16	0.11	-0.1	-0.03	0	-0.04	**0.3** **	0.01	**0.74*** **	
Soil NO_3^-	**-0.28***	*-0.21*	**0.28***	**0.41*** **	**-0.35** **	-0.04	-0.1	*0.2*	**-0.31** **	-0.14

Note: Statistical significance is indicated by asterisks: *$p < 0.05$, **$p < 0.01$, ***$p < 0.001$. Marginal significance ($0.05 \leq p < 0.1$) is in italic.

For comment 3) Present total effects (direct + indirect) with confidence intervals and clarify how model selection affected path retention.

We plotted the net effects (dark grey), which are decomposed into direct effects (light grey) and indirect effects mediated by leaf C:N (green) or root Ca (orange). Net, direct, and indirect effects are shown only for aboveground and belowground communities. Soil-surface communities are not shown, because no significant effects were detected for either abundance or diversity. We attempted to estimate confidence intervals using bootstrapping with the R package '**semEff**'. However, leaf C:N functions as a mediator for both tree species richness (TSR) and the proportion of EcM trees (EcM %), which caused issues in '**semEff**' and produced unstable results. We therefore report the standardized effects directly from the SEM without confidence intervals. A similar presentation of standardized effects without confidence intervals has been used in previous studies (e.g., Schuldt et al., 2019).

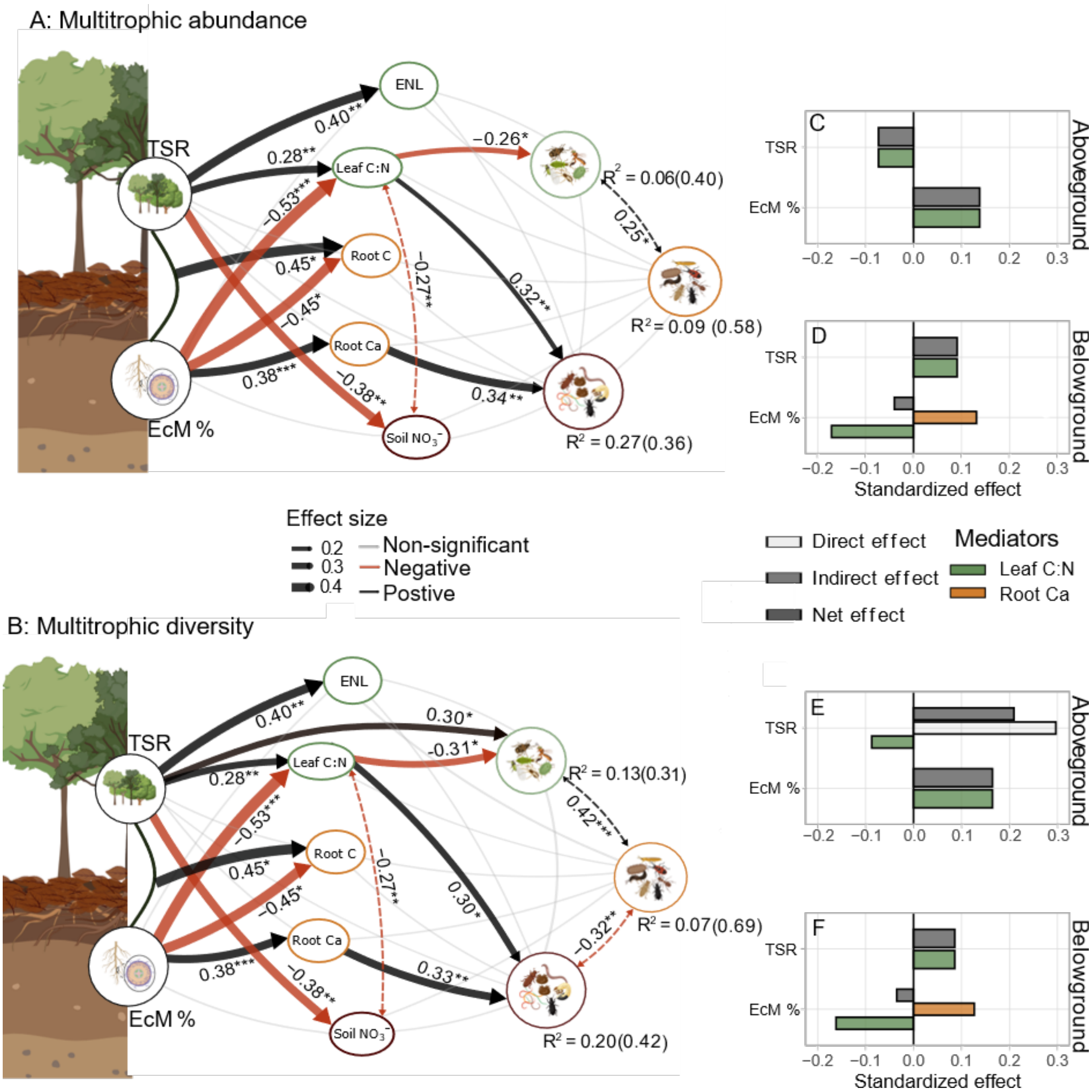

Figure 3. Structural equation models showing how tree species richness (TSR) and mycorrhizal type (proportion of EcM tree species; EcM%) affect multitrophic abundance (A) and diversity of invertebrates (B). The invertebrates are grouped by strata: aboveground (green bubbles), soil surface (orange bubbles), and belowground (brown bubbles). Treatment effects are mediated by canopy complexity (effective number of layers; ENL), leaf C:N, root C and Ca, and soil NO_3^-. Model fit: abundance (Fisher's C = 35.63, df = 44, p = 0.812); diversity (Fisher's C = 32.52, df = 42, p = 0.85). Right panels show total (direct + indirect) effects. Panels C-D and E-F present SEM results for abundance and diversity across strata. Significant effects ($p < 0.05$) are shown as solid black (positive) or red (negative) lines; non-significant paths are shown in grey. Standardized path coefficients are shown with significance levels (*** $p < 0.001$, ** $0.001 \le p < 0.01$, * $0.01 \le p < 0.05$). Interactive effects of tree species richness and mycorrhizal type on root C are shown by a connecting line between two icons. Marginal R^2 values for each final endogenous variable are noted next to their icons (Conditional R^2 values in bracket ()). Conditional R^2 values for mediating variables are reported in Table S11. The multitrophic abundance and diversity were calculated as the mean of min–max–transformed values for each trophic group within the corresponding stratum. The sensitivity analysis based on the z-score transformation is shown in Figure S15 and Table S12. Created in BioRender. Eisenhauer, N. (2025) https://BioRender.com/jvnwool.

Model selection and simplification procedures are described in detail in the Methods (lines 823–867) and Supplementary Text ("Supplementary Text for Construction of Structural Equation Models "). Briefly, we started with a full SEM including all hypothesized pathways among treatments, mediators, and multitrophic responses. Model simplification was then performed by sequentially removing unsupported paths while monitoring model fit (AIC), marginal R^2, and tests of directed separation. All a priori hypothesized pathways among key variables (leaf C:N, ENL, root Ca and C, soil nitrate) were retained unless clearly unsupported. Removal of these paths had minimal effects on marginal R^2 and overall model fit, indicating that the reduced models remained robust representations of the hypothesized system. The links from treatment factors (TSR & EcM %) to each final endogenous variable were only introduced when tests of directed separation indicated missing relationships ($p < 0.1$). As a result, the final models represent simplified versions of the full hypothesized network, rather than data-driven restructuring of the system.

We further evaluated the robustness of model selection through a sensitivity analysis comparing ENL with total wood volume as an alternative proxy for aboveground productivity (Methods, lines 862–867). Specifically: **"To test whether aboveground productivity alone influenced the abundance or diversity of consumers, particularly multitrophic abundance, we conducted a sensitivity analysis by replacing ENL with total wood volume, and by adding total wood volume to the SEMs (Tables S13-14). In both cases, wood volume had no significant or marginal effects on any endogenous variables (Tables S13-S14), further supporting the structural robustness of our final model."**

3. Interpretation of key results

•The manuscript convincingly shows that tree diversity promotes belowground biodiversity more strongly than aboveground. However, the discussion should better integrate mechanistic explanations for this pattern—such as differences in trophic connectivity, resource stability, and dispersal limitation between above- and belowground systems.

Thanks for this excellent suggestion. We revised the explanation of our results in lines 138-153. Now, it reads: **"The results likely reflect differences in dispersal limitation and resource stability between above- and belowground systems. Increased aboveground productivity at higher tree diversity [19] likely leads to greater carbon inputs belowground through root growth [53], rhizodeposition [54,55], and mycorrhizal associations [22,36], thereby enhancing resource availability for belowground communities. Tree diversity probably also increased belowground resource heterogeneity via these pathways [56]. Belowground resources are often spatially structured, and soil organisms typically exhibit lower mobility than organisms in higher strata, making them more strongly influenced by local resource availability and plot-level environmental conditions [57]. This localized resource dependence could lead to stronger bottom-up effects [58], promoting consumer abundance and diversity at higher trophic levels. In contrast, aboveground organisms often exhibit stronger host specialization (e.g., herbivores) and greater mobility (e.g., predators), making consumer community structure more dependent on tree species identity or functional traits rather than plot-level conditions [59,60]. In addition, tree diversity-driven differences in resource inputs to soils may accumulate over time [61], leading to stronger biodiversity responses belowground than in higher strata, where communities are more strongly influenced by seasonal dynamics (Tables S4-S6)."**

•The authors state that their findings partially support hypothesis (1) but contradict hypotheses (1a) and (1b) (Lines 111–119). This is confusing, as hypothesis (1) appears to consist of sub-hypotheses (1a) and (1b). If both 1a and 1b are contradicted, then hypothesis (1) as a whole should also be considered unsupported. Please clarify the logical relationship between these hypotheses and revise accordingly.

Thank you for pointing out the confusing parts. We have revised the text accordingly (see lines 130-133 and lines 156-157). The revised sentences now read: **"Overall, tree diversity**

increased the abundance and diversity of microorganisms and invertebrates, with the strongest effects observed in the belowground compartment. This contrasts with our expectation that plant diversity effects would be stronger aboveground (Hypothesis (1b)).” We also clarified that **“Moreover, the results did not support any clear pattern of stronger effects at lower trophic levels than at higher ones (Figs 1-2; Figs. S11 & S13; Hypothesis (1a)).”**

•As an experimental study, the authors have clearly highlighted main findings based on previous experimental research. However, it would be better to consider some relevant empirical studies, especially where the results diverge from earlier experimental findings. For instance, in lines 171–239, the authors report that AM+EcM mixtures have relatively weak effects on biodiversity, whereas AM and EcM tree communities exhibit distinct diversity patterns. This outcome is somewhat unexpected for experimental studies but aligns with patterns reported in empirical work supporting the EcM dominance hypothesis (e.g., Mao et al. 2023, Ecol Monogr, Luo et al. 2024. New Phytologist). Incorporating discussion of such empirical findings would help contextualize the results within a broader ecological framework.

Thank you very much for the helpful references; we added a new statement to our Discussion in lines 243-247. **“Our results showed that AM communities supported the highest microbial and nematode diversity, consistent with previous studies in mature forests reporting that AM dominance promotes species coexistence, thereby increasing plant diversity [75,76]. Therefore, the mycorrhizal type effects on belowground diversity may extend to large-scale, mature forests, although further empirical evidence is needed.”**

•The statement regarding canopy complexity differences between AM and EcM tree communities should be qualified (Lines 184–186). In natural forests, canopy structural complexity indeed differs between these two mycorrhizal types. Therefore, this conclusion should be explicitly limited to the experimental forest communities examined here.

We revised the text (in lines 212-216) and now it reads **“In this study, mycorrhizal mixtures did also not generate any synergistic effects on tree canopy complexity, a structural feature linked to microhabitat and refuge availability [19]. This pattern may contribute to the lack of synergistic effects of mixing different mycorrhizal types on the taxonomic richness and Shannon-Wiener diversity of aboveground organisms.”**

Indeed, we stressed the difference of canopy structure between AM and EcM tree communities in lines 248-252. **“Mycorrhizal type effects also extended to the aboveground organisms: EcM tree communities supported higher abundances ($F_{2,54}$ = 3.8, p = 0.029) and diversity ($F_{2,54}$ = 4.0, p = 0.025) of predators than AM communities (Figs. 1B & 2B). EcM tree species, e.g., oak and beech trees, with dense branch systems and later leaf senescence, provide greater and more stable habitats for predators, favoring both their abundance and diversity (Fig. 1B) [77].”**

4. Figures and data presentation

•Figures 1 and 2 are informative but overly dense. Consider simplifying by grouping trophic levels or adding schematic legends to enhance readability.
Additionally, clarify whether the shown relationships (e.g., solid vs. dashed lines) were tested across life forms or pooled across them.

We agree that Figures 1 and 2 contain dense information, as they summarize responses of 34 trophic groups across three food-web compartments. To improve readability, we have widened these figures to enhance visibility (see the updated figures). The EcM column is highlighted with a shaded background for visual clarity. We chose to retain the labels adjacent to trophic groups that respond significantly to tree diversity and mycorrhizal type, as this allows readers to immediately identify responsive groups without searching through

extensive legends. This presentation follows a successful precedent in multitrophic biodiversity studies (e.g. Scherber et al. 2010, Nature).

Regarding life forms, responses were not pooled across developmental stages. Instead, immature (juvenile) and adult stages, particularly for insects, were analysed and presented separately. This distinction is now clarified in the Methods section, and detailed information on taxonomic groupings and life forms is provided in the Supplementary Information.

The methodology in lines 666-672 reads **"Feeding niches often differ between life stages, particularly in insects. Therefore, we assigned trophic groups separately for juvenile and adult stages within the same taxonomic group [10]. For example, Diptera exhibit ontogenetic shifts in feeding preferences and habitat use between larval and adult stages. In contrast, many Hymenoptera are parasitoids throughout their life cycle; although the parasitic stages were not sampled and adults rarely feed, we classified them as parasitoids (or predators) following previous studies to reflect their ecological role [11,18]."** Meanwhile, we also attached Supplementary Data describing the taxonomic groups and their assigned trophic groups.

•Figure 3 (SEM) is visually complex. I recommend moving the detailed trophic-level SEMs (currently Fig. 4) to Supplementary Materials and keeping a simplified version in the main text showing only key mediating paths.

We chose to retain Figure 4 in the main text, because it directly illustrates how leaf traits, canopy complexity, and soil and root nutrients mediate treatment effects on biodiversity across trophic levels and food-web compartments. Moving this figure to the Supplementary Materials would obscure important differences in how individual trophic groups respond to specific mediating pathways. These trophic group-specific responses are ecologically relevant, including for forest management and biodiversity assessment, as contrasting responses among trophic groups can offset one another when aggregated (e.g. opposing effects of soil nitrate on different trophic groups). We put the model structures and their performance for each trophic group in each food web compartment in the supplementary information to increase the transparency and reproducibility (see Tables S15-S17).

We also justified the reason why we included it in the Results and Discussion sections. It now reads (lines 342-357) :

"The SEMs showed that soil NO_3^- (nitrate), which decreased with tree diversity due to efficient plant uptake [86], was not significantly associated with multitrophic abundance or diversity (Fig. 3), as responses varied among trophic groups (Fig. 4). Soil NO_3^- was negatively correlated with nematode fungivore abundance but positively with bacterivore abundance and richness (Fig. 3E-F). This pattern is consistent with the bacterial energy channel being favored by mineralization via nitrifying bacteria over the fungal channel [89].

Soil C was not included in the SEMs (see Methodology), yet it was significantly associated with increased nematode and mesofauna abundance, particularly omnivores (Fig. 4E). As an indicator of habitat structure and organic matter availability [15], soil C together with root C may explain the higher abundance of these groups in AM plots (Fig. 1). The SEMs captured mediating effects on invertebrate communities (Fig. 3) but did not include microbes. Tree diversity increased canopy complexity (ENL), reflecting greater aboveground biomass, litter input, and microclimatic buffering (Table S10) [19,90], and was positively associated with microbial biomass and diversity (Fig. 4E-F). In addition, leaf C:N, which increased with tree diversity and was higher in AM plots, was positively correlated with soil microbial diversity (Fig. 4F), e.g., pathogens, mycorrhizae, and saprotrophs (Fig. 4F; Fig. S5) [73,74]."

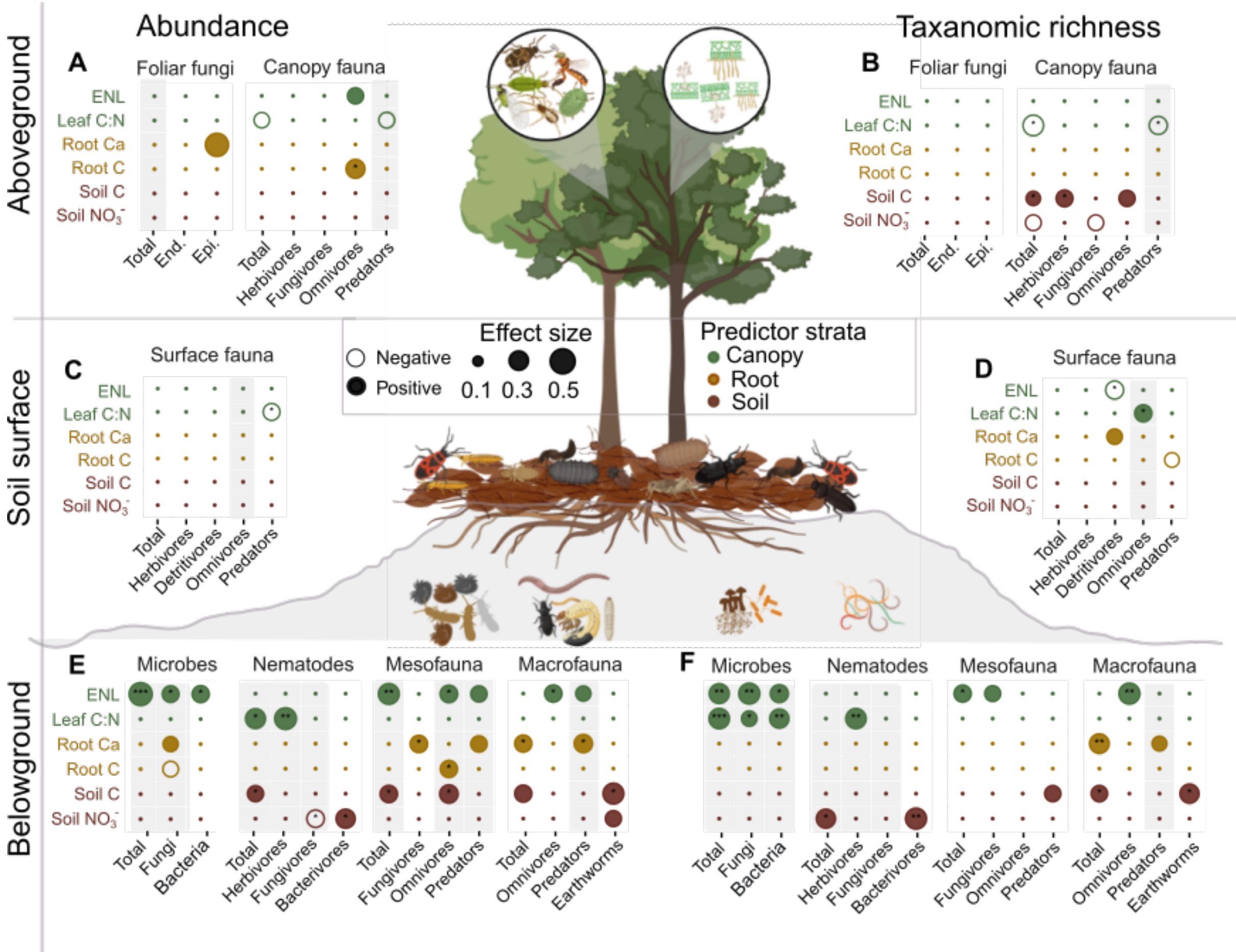

Figure 4. Results of multiple linear regressions showing the effects of six mediators on standardized organism abundance and taxonomic richness aboveground (A-B), at the soil surface (C-D), and belowground (E-F). Left-hand panels (A, C, E) display results for abundance, and right-hand panels (B, D, F) for taxonomic richness. Within each panel, individual trophic groups are shown as columns grouped into broader categories (e.g., foliar fungi, canopy fauna, microbes, nematodes, mesofauna, macrofauna) labelled at the top. Only explanatory variables retained in the best-fit models after backwards selection are shown. Significant effect sizes are represented by circles: filled for positive effects, and empty for negative effects, with size proportional to standardized coefficients. Non-selected variables are shown as small dots. Statistical significance is indicated by asterisks: ***$p < 0.001$, **$0.001 \leq p < 0.01$, * $0.01 \leq p < 0.05$; no asterisk indicates marginal significance ($0.05 \leq p < 0.1$). Colors indicate the source of each explanatory variable: green for canopy conditions (leaf C:N ratio, ENL (effective number of layers)), yellow for root nutrients, and brown for soil conditions. The model structure and performance are presented in Tables S15-S16. Panels are shaded when the biodiversity response was significantly affected by tree species richness and/or mycorrhizal type (see Figs. 1-2). Created in BioRender. Eisenhauer, N. (2025) https://BioRender.com/jvnwool.

5. Limitations and broader implications

•The Discussion would benefit from a short section on study limitations, particularly: (a) the relatively young age of the MyDiv trees (six years), (b) potential underestimation of aboveground effects due to canopy closure stage, and (c) lack of direct measurements of litter traits or decomposition rates, which could clarify the link between mycorrhizal type and resource heterogeneity.

We thank Reviewer 2 for highlighting these limitations and agree with all of the points raised. We have incorporated these limitations in the manuscript at the relevant sections where they

affect the interpretation of our results, which also improves the overall logic and flow of the manuscript.

For (a), we highlighted the limitation in lines 206-209: **"Although Luo et al [37] reported synergistic productivity effects, whereby mixed AM+EcM forests outperformed single mycorrhizal types, we found no such effects six years after establishing the MyDiv Experiment [63]. This may explain the absence of any short-term synergistic responses of the abundance of microorganisms and invertebrates."**

For (b), we noted this limitation at two different places. In lines 153-155: **"Notably, field samples taken at or near canopy closure (in May and September) may underestimate aboveground effects, as reduced variation in light and microclimate can dampen tree diversity responses."**

In lines 319–322: **"All mediators were assessed during the growing season. This may have limited our ability to capture mechanisms by which aboveground organism responses were driven by phenological variation among mycorrhizal types and tree species (Bönisch et al., 2026)."**

For (c), in lines 325-327: **"We acknowledge that limited data on litter quality and its heterogeneity constrained our ability to resolve how tree diversity influences soil-surface fauna [56]. "**

•While the authors emphasize the applied implications for forest design, this point could be strengthened by discussing how tree–mycorrhiza diversity might be operationalized in restoration or carbon sequestration programs.

In the section of "No interactive treatment effects for most trophic groups", we added the text (in lines 299-304): **"The effects of tree diversity on root feeders and predators depended on mycorrhizal type. Incorporating EcM tree species into AM dominated plantations may enhance predator abundance and diversity across above- and belowground food webs, thereby reducing herbivory and disease risk in AM tree stands. As a consequence, above- and belowground carbon storage can be enhanced."**

•Furthermore, the practical significance of this study could be strengthened by linking the results to forest dynamics under future global change. Several recent studies have projected a potential decline in EcM tree dominance and a corresponding increase in AM tree dominance in temperate forests under global change (e.g., Averill et al. 2018, Global Change Biology, Jo et al. 2019 Science Advances, Clavel et al. 2024. Ecography).

This is a very nice suggestion.

We added some respective text in Abstract (line 17-19 & 32-25) and Introduction (lines 84-86), as well as at the end of section "Mycorrhizal types structure microbial and invertebrate communities" in Results and Discussion (lines 263-269).

In abstract: **"Forests support over 80% of terrestrial biodiversity, yet global tree diversity loss and shifts in mycorrhizal dominance may threaten ecosystem functioning due to cascading multitrophic biodiversity decline." & "These findings establish a mechanistic foundation for designing diverse forests to sustain ecosystem functioning, and provide an empirical basis for predicting biodiversity responses to shifts in mycorrhizal dominance under anthropogenic pressures."**

In Introduction: **"As climate warming, drought, and anthropogenic pressures shift mycorrhizal dominance in forests [41–43], empirical tests are urgently needed to predict the biodiversity responses across trophic levels."**

In Results and Discussion: **"Anthropogenic influences (e.g., nitrogen deposition, fire suppression, and infrastructure expansion), together with climate change (warming and drought), are shifting temperate forests towards AM dominance at the expense of EcM trees [41–43]. Our results provide empirical evidence to predict how such shifts may affect multitrophic diversity across above- and belowground food webs. Further observational studies are needed to assess biodiversity changes along such gradients, which underpin ecosystem functioning and conservation."**

6. Other comments

•Title: "Mycorrhizal type" is important in the hypotheses and results, which I think should be included in the title.

Agreed. We changed the title to **"Tree diversity and mycorrhizal type have stronger effects on belowground than aboveground multitrophic biodiversity"**.

•Some key concepts—particularly additive effects and synergistic effects—are used repeatedly but are not clearly defined or quantitatively described. Please provide explicit definitions and indicate how these effects were evaluated in the analyses.

We have revised the Introduction to explicitly define synergistic effects in the context of this study.The revised text now appears in lines 70-73 as follows:

"Complementary resource-use strategies may partition soil nutrients and space, such that mixed AM and EcM (AM+EcM) forests can be more productive than single-type forests (i.e., synergistic effects) [37]. Therefore, synergistic effects exceed additive effects of mixing different mycorrhizal types."

In the analyses, synergistic effects were evaluated quantitatively by comparing responses among AM- and EcM-only plots, and mixed AM+EcM plots. Specifically, we classified effects as synergistic when the response variable (abundance or taxonomic richness) in AM+EcM communities exceeded that observed in both single mycorrhizal types, corresponding to a transgressive overyielding criterion. This approach provides a conservative test of non-additive effects and is consistent with evidence that mixed AM+EcM forests achieve productivity maxima at intermediate mycorrhizal composition (Luo et al. 2023).

In the results section, we were clarifying better with the wording of "synergistic effects and additive effects". The revised text reads **"The abundances of microorganisms and invertebrates in AM+EcM communities consistently fell between those of AM and EcM tree communities across above- and belowground compartments (Fig. 1B, D, F). A similar pattern was observed for taxonomic richness (Fig. 2B, D, F). These findings contrast with our expectation (Hypothesis 2) that mixing mycorrhizal types would enhance microorganism and invertebrate abundance and diversity. Although Luo et al [37] reported synergistic productivity effects, whereby mixed AM+EcM forests outperformed single mycorrhizal types, we found no such effects six years after establishing the MyDiv Experiment [63]. This may explain the absence of any short-term synergistic responses of the abundance of microorganisms and invertebrates."**

•The classification of trophic levels in the manuscript is somewhat unclear and could be improved for greater ecological accuracy and reader comprehension.

(1) Predators occur across multiple size classes (e.g., nematodes, microfauna, and macrofauna). From the perspective of the overall soil food web, there are also predator–prey interactions among these groups, which should be clearly distinguished when describing trophic relationships.

We added a supplementary dataset that clearly defines taxonomic groups and their trophic assignments, improving transparency and reproducibility. We agree that predation interactions occur among these groups; for example, Yi et al. (2025, *Current Biology*)

demonstrated such interactions using food-web reconstruction and energy flux estimates. However, applying this approach here is not feasible, as further subdividing trophic groups would introduce many zero values, inflating sparsity and preventing reliable fitting of mixed-effects models.

(2) The classification of nematode trophic groups follows specific ecological terminology (e.g., bacterivores, fungivores, plant feeders, omnivores, and predators). It would be helpful to explicitly describe the criteria or references used to assign nematode taxa to different trophic categories.

We primarily relied on three references (see below), now detailed in the supplementary text and Supplementary Data 1.

18. Mulder, C. & Vonk, J. A. Nematode traits and environmental constraints in 200 soil systems: scaling within the 60–6000 µm body size range. *Ecology* 92, 2004–2004 (2011).

19. Nemaplex.UCDavis.edu. Nemaplex. http://nemaplex.ucdavis.edu/ (2024).

20. Yeates, G. W., Bongers, T., De Goede, R. G. M., Freckman, D. W. & Georgieva, S. S. Feeding Habits in Soil Nematode Families and Genera-An Outline for Soil Ecologists. *J Nematol* 25, 315–331 (1993).

(3) Many soil organisms are omnivorous, and their trophic positions may shift depending on resource availability and environmental conditions. This ecological flexibility should be acknowledged and discussed, as it can influence interpretations of soil food-web structure and biodiversity patterns.

Agreed. We added respective text to the section “Tree diversity promotes belowground more than aboveground biodiversity (lines 183-190): **“Notably, for consistency, we assigned soil meso- and macrofauna to trophic groups based on established classifications; however, this did not capture trophic plasticity driven by resource availability and environmental conditions [65]. Many soil taxa, especially mesofauna (e.g., oribatids), are omnivorous and often were identified at low taxonomic resolution; thus, grouping them into broad trophic categories likely limited our ability to detect diversity responses [66]. Incorporating stable isotope approaches with finer taxonomic resolution may help address this caveat in future studies [66–68].”**

Reviewer #3 (Remarks to the Author):

An excellent paper by Yi and others demonstrating how tree diversity and mycorrhizal type shape multitrophic biodiversity across above- and belowground compartments. The experimental and statistical methodology are well-designed and robust, integrating extensive data from the MyDiv experiment with microbial, nematode, meso- and macrofaunal communities, plant traits, carbon and nutrient content. The authors show that belowground biodiversity responses to tree diversity are stronger than aboveground responses, which fills an existing knowledge gap. These responses are further linked to canopy structure, nutrient mediation, and mycorrhizal strategies to provide new mechanistic insight. This contribution is timely, with direct implications for the design of biodiversity-based reforestation and restoration strategies, highly novel, well contextualized in BEF theory and integrates above- and belowground data in a remarkably (given the complexity of the dataset) comprehensive way with some excellent figures. I have few moderate and several minor suggestions for improvement focused on statistical transparency and clarifying comparisons across compartments.

Thanks a lot for this very positive feedback and the valuable suggestions. We addressed your comments below one by one.

Main comments

Figs 1&2, Tables S1-S3: While standardized slopes and 95% CIs are shown, their biological magnitude is sometimes difficult to interpret. Could absolute abundance or richness changes be provided in supplementary tables to complement standardized effects, or in the supporting text (e.g. 'Tree diversity increased soil fungal richness by ≈ 3 OTUs per additional tree species').

Agreed. We ran the same mixed-effects modelling framework on the original (before min–max transformed) data. The results are now presented in the Supplementary Information: **Fig. S2** for canopy fauna, **Fig. S3** for soil-surface fauna, **Fig. S4** for belowground fauna and microbial communities, and **Fig. S5** for different functional groups of soil fungi. These plots allow direct visualization of the absolute increases along the tree diversity gradient as well as differences between mycorrhizal types.

Figs 1&2, Tables S4-S8: The conclusion that belowground responses are stronger relies mainly on differences in standardized effect sizes and significance levels. The paper would benefit from a more explicit statistical test comparing the magnitude of diversity effects between strata (e.g. hierarchical models with stratum × log(tree richness)).

We agree that a formal cross-stratum comparison would be desirable. However, pooling all trophic groups into a single hierarchical model was not straightforward, because the responses differ in biological meaning, sampling structure, and variance among groups. In our initial analysis, we ran independent models for each trophic group and across food web compartments, and also used the group-specific power transformation values to address the difference of variance.

We agreed that our initial conclusion was drawn based on the significance. To complement this approach, we calculated the effect sizes, the log-response ratio between predicted values in polycultures (at species level 2 and 4) and monoculture. By comparing the effect sizes across trophic groups, as well as the food web compartments, we aimed to address the reviewer's valid comment and strengthen our conclusions. Meanwhile, this approach could also address the reviewer's comment below. The method details are written in the methodology section (lines 796-803): **"To facilitate statistical comparisons of tree diversity effects across trophic groups and food-web compartments, we extracted model predictions (mean ± 95% confidence interval) at three levels of species richness (1, 2, 4) using the *emmeans* package. The log-response ratios of predicted values for 2 vs. 1 and 4 vs. 1 tree species were calculated as standardized effect sizes**

to quantify the magnitude of tree diversity effects across trophic groups and food-web compartments. In addition, the log-response ratios were calculated to assess the magnitude and robustness of tree diversity effects beyond significance levels."

The results are presented in Fig. S11 (derived from min–max transformed data) and Fig. S13 (derived from the original data). In addition, we incorporated key information into the Results and Discussion sections to support our conclusion originally based on significance levels. The text now reads (lines 130-137): **"Overall, tree diversity increased the abundance and diversity of microorganisms and invertebrates, with the strongest effects observed in the belowground compartment. This contrasts with our expectation that plant diversity effects would be stronger aboveground (Hypothesis (1b)). This pattern was supported by the analysis of log-response ratios comparing predicted values of polycultures with monocultures (Figs. S11-S13). The log-response ratios for the majority of aboveground and soil-surface groups had their confidence intervals overlapping zero, whereas most belowground groups showed positive responses with confidence intervals excluding zero (Figs. S11, S13)."**

Moreover, to provide a more explicit statistical comparison of the magnitude of diversity effects among strata, we added **"We calculated mean effect sizes (log-response ratios) of trophic groups within each compartment for abundance and taxonomic richness using the min–max transformed data. Differences were tested with the model (mean(log-response ratio) ~ Stratum × Richness contrast), where stratum included aboveground, soil surface, and belowground compartments, and the richness contrast included the 2- and 4-species treatments. These results summarize the response magnitude across food-web compartments but may be biased, because more trophic groups were included in the belowground compartment, and variation among trophic groups was not accounted for."** Please find the text in lines 809-816, and the resulting figure in Fig. S12.

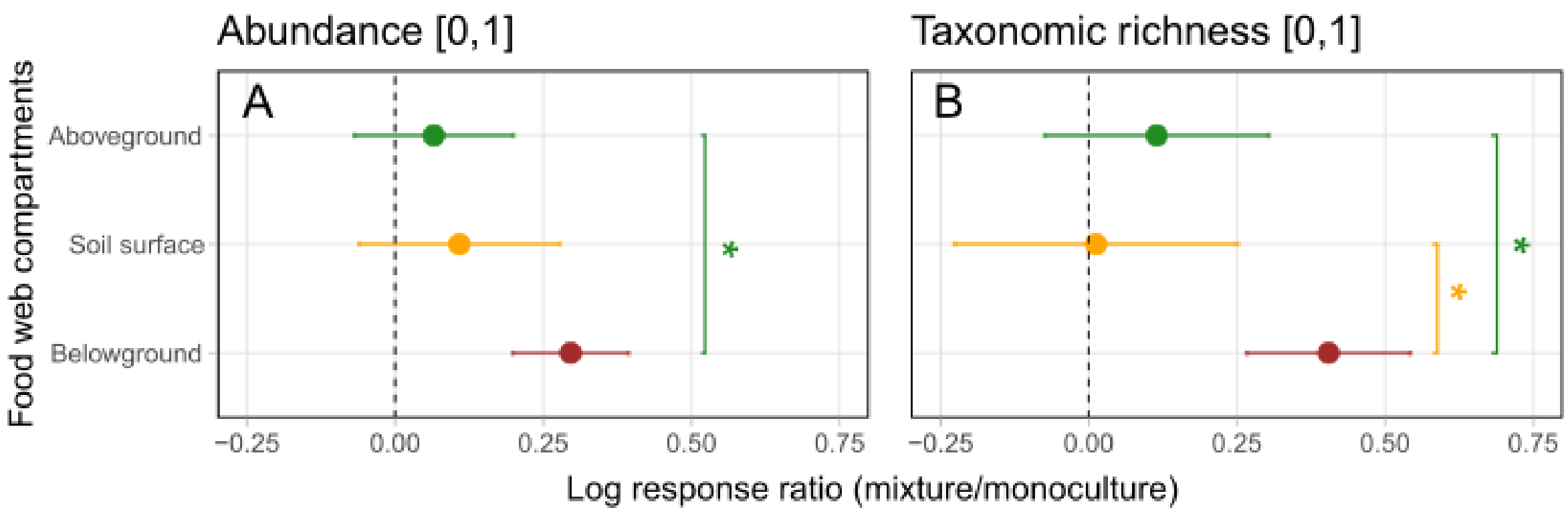

Figure S12. Averaged effect sizes of trophic groups across three food-web compartments. This figure was derived from Fig. S11 by extracting the mean effect sizes of trophic groups within each compartment for both abundance (A) and taxonomic richness (B). Differences in mean effect sizes were tested using the model (mean(log-ratio) ~ Stratum × Richness contrast). Richness contrast (2- and 4- species) had no significant effect on the averaged effect sizes, whereas effect sizes differed among food-web compartments, indicated by "*" ($p < 0.05$). Notably, these results could be biased, because more trophic groups were included in the belowground compartment than for aboveground and at the soil surface strata, and we did not account for variation among effect sizes across trophic groups. Therefore, this figure should be used only as a summary reference, while the main results rely on Fig. S11 and Fig. 1&2.

The authors employ a solid statistical framework of mixed-effects models with random factors (composition, season), power transformations for scaling, and SEM to assess mediation. However, transparency and reproducibility could be improved by 1) providing

model formulae (e.g. lme(response ~ log(tree richness) + mycorrhiza + interaction + month + (1|composition/month)) in the supplement; 2) reporting the number of plots per treatment per season explicitly in the methods (not clear); 3) provide a statement on how Type I error inflation was handled (e.g. FDR correction or emphasis on effect sizes rather than p-values); 4) Provide a SEM goodness-of-fit summary table (χ^2, RMSEA, CFI) in the supplement.

For comment 1) providing model formulae (e.g. lme(response ~ log(tree richness) + mycorrhiza + interaction + month + (1|composition/month)) in the supplement;

We added the model formulas to the notes under Tables S4–S8. For example, the footnote of Table S4 now states **"For each parameter of each trophic group (y_i), min–max transformed data were further power-transformed using the t-values reported in Table S1. Models were then fitted as linear mixed-effects models: *lme*(y_i^t ~ Month + Mycor × log(Richness), random = ~1 | composition/Month), using Type I sums of squares. Outlier removal was conducted separately for each biodiversity index and functional group, resulting in different final numbers of observations among models. Therefore, the numerator and denominator degrees of freedom ($F_{numDF, denDF}$) are reported specifically for each response variable and parameter."**

The remaining information is provided in the supplementary information.

In addition, we provide details of the selected models (using the *dredge* function) for mediating effects (Fig. 4) on each diversity metric for each trophic group across the three food-web compartments in Tables S15–S17.

For the SEMs, we added detailed descriptions in the supplementary text outlining the full models, model selection procedures, and final SEM structures. We include these details below for reference.

"Supplementary Text for Construction of Structural Equation Models

For the final SEM framework in Fig. S7, we only included mediators that were significantly affected by the treatments (see Table S9, Fig. S6) and were not strongly correlated with each other (Table S10; details written in lines 823-843). The final set of mediators included ENL, leaf C:N, root Ca, root C, and soil NO_3^-. Soil C was excluded, because its response pattern indicated non-additive treatment effects (e.g., lower values in AM + EcM mixtures than in AM plots; Fig. S1), which could not be adequately represented by the linear coding used in the SEM.

Based on the hypothesized relationships (Fig. S1; Fig. S7), we first fitted a full piecewise structural equation model (SEM) including all plausible pathways among treatment variables, mediators, and multitrophic responses. All component models were fitted as linear mixed-effects models with composition included as a random effect, except for root Ca, for which the random-effect variance was negligible. EcM% represents the proportion of EcM tree species, and log(Richness) represents log-transformed tree species richness. The multitrophic diversity and abundance are shortened as multi-indices, or multi-abundance, or multi-diversity.

aboveground multi-index ~ Leaf C:N + ENL
surface multi-index ~ ENL + Leaf C:N + root Ca + root C + Soil NO3-
belowground multi-index ~ ENL + Leaf C:N + root Ca + root C + Soil NO3
ENL ~ log(Richness) + EcM%
Leaf C:N ~ log(Richness) + EcM%
root Ca ~ log(Richness) + EcM%
root C ~ log(Richness) + EcM% + log(Richness) × EcM%
NO_3^- ~ log(Richness) + EcM%

The initial SEM was simplified by sequentially removing unsupported paths while monitoring model performance, marginal R^2, and tests of directed separation. Removal of unsupported predictors had little effect on marginal R^2 or overall model

fit. Additional direct pathways were included only when tests of directed separation indicated missing relationships ($p < 0.1$). The final SEMs therefore represent reduced versions of the hypothesized full model.

The final models for multitrophic abundance were:

aboveground multi-abundance ~ Leaf C:N + ENL
surface multi-abundance ~ ENL + Leaf C:N + root Ca
belowground multi-diversity ~ ENL + Leaf C:N + root Ca + log(Richness)

The final models for multitrophic diversity were:

aboveground multi-diversity ~ Leaf C:N + ENL + log(Richness)
surface multi-diversity ~ ENL + Leaf C:N + root Ca
belowground multi-diversity ~ ENL + Leaf C:N + root Ca "

For comment 2) reporting the number of plots per treatment per season explicitly in the methods (not clear);

The experimental design (8 treatment combinations × 10 replicates = 80 plots) was described in the section "Study site and experimental design" (lines 474–479). The text reads: **"The design consists of a tree diversity gradient (1, 2, and 4 species) and three mycorrhizal-type treatments (only AM tree species [AM plots], only EcM tree species [EcM plots], or both mixed [AM+EcM plots]). In total, there are eight treatment combinations: 3 × AM plots (1, 2, 4 species), 3 × EcM plots (1, 2, 4 species), and 2 × AM+EcM plots (2 and 4 species), each replicated ten times, resulting in 80 plots (11 × 11 m) located across two blocks."**

To clarify the sampling structure for different trophic groups, we now explicitly state this information in the Methods for invertebrates (lines 691–692), foliar fungi (lines 696–701), and soil microbial communities (lines 705–707). The revised text reads as follows:

Invertebrates: **"All invertebrate trophic groups were sampled in all 80 plots (10 replicates per treatment) during two sampling campaigns. "**

Foliar fungi: **"For foliar fungi, their taxonomic richness was estimated using the Chao1 richness estimator (Chao1) [150]. The Abundance-based Coverage Estimator (ACE [151]) was also used for comparison; the values were these similar to those obtained with Chao1, and therefore only Chao1 is presented in the results. The Shannon-Wiener diversity index was calculated. In total, we calculated biodiversity metrics for endophytes in 39 plots (out of 42) and for total foliar and epiphytic fungi in 34 plots [102]."**

Soil microbial communities: **"Soil microbial biomass was measured in both seasons (80 plots × 2 seasons), whereas fungal and bacterial diversity indices were determined once per plot (80 plots) at the end of the growing season."**

For comment 3) provide a statement on how Type I error inflation was handled (e.g. FDR correction or emphasis on effect sizes rather than p-values);

Rather than applying formal multiple-testing corrections across trophic groups, we fitted separate mixed-effects models for each trophic group using a small set of predefined predictors associated with our a priori hypotheses (month, tree diversity, mycorrhizal type, and their interaction). Because these tests were hypothesis-driven rather than exploratory, the number of statistical comparisons within each model was limited. FDR correction does not fit our context. Therefore, we evaluated the robustness of the results by using standardized effect sizes (log-response ratios) and their 95% confidence intervals derived from model predictions. These effect-size estimates supported the same conclusions as the significance tests. We specifically explained this now in the methodology in lines 794-808:

“Because group-specific power transformations were applied, the coefficients of $log(Richness)$ could not be directly compared to determine which groups exhibited stronger tree diversity effects across the three strata. To facilitate statistical comparisons of tree diversity effects across trophic groups and food-web compartments, we extracted model predictions (mean ± 95% confidence interval) at three levels of species richness (1, 2, 4) using the *emmeans* package. The log-response ratios of predicted values for 2 vs. 1 and 4 vs. 1 tree species were calculated as standardized effect sizes to quantify the magnitude of tree diversity effects across trophic groups and food-web compartments. In addition, the log-response ratios were calculated to assess the magnitude and robustness of tree diversity effects beyond significance levels. The log-response ratios were calculated using both the min–max transformed data and the original values. For most trophic groups, the results were consistent between the two approaches, confirming the robustness of our findings (Fig. S11 & S13). The only exception was soil fungal and bacterial taxonomic diversity, where the log-response ratios based on the original data were slightly lower than those derived from the transformed values.”

Moreover, we also revised the section of Results and Discussion to not only focus on the significance levels, but also the effect sizes (lines 131-137 and 159-168). They respectively read as: **“This contrasts with our expectation that plant diversity effects would be stronger aboveground (Hypothesis (1b)). This pattern was supported by the analysis of log-response ratios comparing predicted values of polycultures with monocultures (Figs. S11-S13). The log-response ratios for the majority of aboveground and soil-surface groups had their confidence intervals overlapping zero, whereas most belowground groups showed positive responses with confidence intervals excluding zero (Figs. S11, S13).”**

“In the belowground compartment, the strongest effects on abundance were observed on macrofauna predators ($F_{1,56}$=11.6, p = 0.003), followed by nematode herbivores ($F_{1,56}$=7.9, p = 0.008) and fungivores ($F_{1,56}$=5.64, p = 0.02), as well as mesofauna predators ($F_{1,56}$=4.87, p = 0.03; Fig. 1E; Tables S7-S8). Moreover, tree diversity most strongly affected the taxonomic richness of soil fungi ($F_{1,56}$ =83.0, p < 0.001) and bacteria ($F_{1,56}$ =62.3, p < 0.001), with smaller effects on macrofauna predators ($F_{1,56}$ = 10.2, p = 0.002), nematode herbivores ($F_{1,56}$=11.2, p =0.001), and fungivores ($F_{1,56}$=4.2, p = 0.04) (Fig. 2E; Tables S7-S8). The magnitude of increase among these trophic groups did not differ significantly, as indicated by the overlap of their 95% confidence intervals for effect sizes (log-response ratios of predicted values in polycultures relative to monocultures) (Fig. S11).”

For comment 4) Provide a SEM goodness-of-fit summary table (χ^2, RMSEA, CFI) in the supplement.

Model fit was assessed using Fisher’s C statistic, following standard practice for piecewise SEM (Shipley, 2013; Lefcheck, 2016). Accordingly, Fisher’s C (with df and *p*) is reported in the figure captions and in the supplementary notes, together with conditional and marginal R^2 (Fig. 3; Fig. S15; Tables S11–S14). Likelihood-based χ^2 statistics could not be calculated because we used mixed effect models (Lefcheck, 2016), and we added the explanation under Table S11-14. In addition, RMSEA and CFI are not reported, as they are not applicable to piecewise SEM.

Minor Comments

Throughout, suggest replacing ‘– … –’ with ‘, … ,’ (e.g. L19-20, L39-40, L185)

We changed throughout the whole manuscript.

L54: Remove ‘the’

Removed.

L57: Suggest replacing 'stronger' with 'more strongly'

Replaced.

Significance is sometimes interpreted at $p < 0.1$ (marginal) which needs to be used cautiously.

Marginal significance were removed from Figs.1–3. In Fig. 4, we removed the "•" marker previously used to indicate marginal significance, allowing significant mediating effects to be identified more clearly. We also removed statements based solely on marginal significance. Where such results are retained for context, we now explicitly report the corresponding F- and p-values. For example (in lines 125-130), **"A similar pattern was observed for taxonomic richness: tree diversity increased the richness of most belowground trophic groups (Fig. 2E; Tables S7-8). Only a marginally significant effect of tree diversity was observed on total aboveground taxonomic richness ($F_{1,54}$ = 3.8, p = 0.06; Fig. 2A; Table S4), while no significant effects were detected for soil-surface fauna (Fig. 2C; Table S6)."**

Statistical notation should be consistent (e.g. $p \leq 0.05$ v $P < 0.05$, Log v $\log_{10}$).

We revised the manuscript to ensure consistent statistical notation throughout. All code was reviewed to confirm that significance is defined as $p < 0.05$, and that log transformations are consistently applied using log rather than log10. Specifically, we corrected the caption of Fig. 4 to use $p < 0.05$, in line with Figs. 1 and 2. In addition, we standardized statistical notation by using lowercase p for all hypothesis tests, including SEM outputs, to maintain consistency across analyses.

L110: "only marginal effects on predator richness (p = 0.07; Tables S4-5)" – I cannot see any figures in italic to signify this in the supplementary tables.

We apologize for the mistake. This was corrected (see Table S4). We also removed the corresponding text that previously explained why tree diversity increased aboveground predator taxonomic richness.

Suggest moving summary R^2 values from Tables S4-S8 into the main text to help readers interpret model performance.

We deliberately refrain from reporting marginal R^2 (R^2m) values from the full models (y_i^t ~ month + Mycor × log(Richness)) in the main text, but still include them in the supplementary information. The primary reason is that sampling month explains a substantial proportion of the total variance, particularly for aboveground and soil-surface compartments. Consequently, R^2m values are often dominated by temporal effects, even when tree diversity and mycorrhizal type exert relatively weak effects on canopy and surface organisms. Reporting R^2m in this context would therefore risk misleading readers about the importance of our focal treatments.

Therefore, we added F-values and associated p-values for those response variables that showed significant treatment effects (lines 123–130, 159-166, 222-230). In our case, F-values are more appropriate and comparable indicators of effect strength, as they directly quantify how much variation each treatment explains relative to unexplained variance. Now, it reads as: **"However, the abundance of aboveground and soil-surface trophic groups showed only limited responses, with significant effects restricted to total foliar fungi ($F_{1,22}$ = 4.6, p = 0.04; Fig. 1A; Table S5) and soil-surface omnivores ($F_{1,54}$ = 4.52, p = 0.04, Fig. 1C; Table S6). A similar pattern was observed for taxonomic richness: tree diversity increased the richness of most belowground trophic groups (Fig. 2E; Tables S7-8). Only a marginally significant effect of tree diversity was observed on total aboveground taxonomic richness ($F_{1,54}$ = 3.8, p = 0.06; Fig. 2A; Table S4), while no significant effects were detected for soil-surface fauna (Fig. 2C; Table S6)."**

"In the belowground compartment, the strongest effects on abundance were observed on macrofauna predators ($F_{1,56}$=11.6, p = 0.003), followed by nematode herbivores ($F_{1,56}$=7.9, p = 0.008) and fungivores ($F_{1,56}$=5.64, p = 0.02), as well as mesofauna predators ($F_{1,56}$=4.87, p = 0.03; Fig. 1E; Tables S7-S8). Moreover, tree diversity most strongly affected the taxonomic richness of soil fungi ($F_{1,56}$ =83.0, p < 0.001) and bacteria ($F_{1,56}$ =62.3, p < 0.001), with smaller effects on macrofauna predators ($F_{1,56}$ = 10.2, p = 0.002), nematode herbivores ($F_{1,56}$=11.2, p =0.001), and fungivores ($F_{1,56}$=4.2, p = 0.04) (Fig. 2E; Tables S7-S8)."

"For example, AM tree communities supported the highest abundance of total nematodes ($F_{2,54}$ = 3.5, p = 0.039), nematode herbivores ($F_{2,54}$ = 8.9, p = 0.005), and mesofauna omnivores ($F_{2,54}$ = 3.4, p = 0.041), whereas EcM tree communities had the highest soil microbial biomass ($F_{2,54}$ = 5.5, p = 0.006) and mesofauna predator abundance ($F_{2,54}$ = 3.5, p = 0.038) (Fig. 1F; Tables S7-8). However, effects on taxonomic richness were more pronounced at lower trophic levels (Fig. 2F). More specifically, total soil microbes ($F_{1,54}$ = 82.8, p < 0.001), soil fungi ($F_{1,54}$ = 79.2, p < 0.001), and nematode herbivores ($F_{2,54}$ = 4.2, p = 0.020) all showed the highest richness in AM tree communities, with no significant differences observed at higher trophic levels (Fig. 2F)."

We considered an alternative approach based on partial R^2m values for individual predictors, which would quantify the variance uniquely attributable to tree diversity and mycorrhizal type. While this could aid interpretation, it also introduces potential pitfalls. For response variables with smaller sample sizes (e.g. foliar fungi) or limited temporal replication (e.g. soil microbial diversity sampled only once), total variance is inherently lower. As a result, even non-significant effects may appear to explain a relatively large proportion of variance, which could be misinterpreted by readers. In addition, adding partial R^2 visualizations alongside Figures 1 and 2 substantially increases figure complexity and reduces overall readability.

L647-649: Can you clarify whether multitrophic abundance/diversity metrics were equally weighted or if trophic groups with missing data were excluded in each case.

We now clarified this point in the manuscript (lines 746-751): **"The multitrophic abundance was calculated as the arithmetic mean across trophic groups with equal weighting. The missing trophic-group values were omitted on a per-sample basis such that each sample's metric reflects the mean of the groups available. Multitrophic diversity was calculated similarly by averaging the min-max transformed taxonomic richness of trophic groups within each stratum."**

Reply to reviewers

We appreciate the time and efforts of the three reviewers, providing valuable suggestions again. We responded to the reviewers' comments in green, and the revised text in the manuscript is highlighted in bold font. All line numbers in the response letter refer to the revised manuscript without track changes.

Reviewer #1

Remarks to the Author:
The manuscript "Tree diversity and mycorrhizal type have stronger effects on belowground than aboveground multitrophic biodiversity " is a revised version of a previous manuscript. The authors have provided additional data and removed the speculative sections of the manuscript, thereby improving the quality. I do think the experimental setup has some limitations and is unrealistic. Nonetheless, monitoring the dynamics of above-ground and below-ground organisms simultaneously, as done in this paper, has great value.

Thank you very much for reviewing our manuscript again and for providing further suggestions to improve it. We have addressed your comments one by one below.

In general, I think the writing could still be improved; it is partly cryptic, and the flow within and between paragraphs could be stronger. I make some more specific remarks below.

Line 66: This paragraph switches across trophic levels to mycorrhizal fungi very unexpectedly; perhaps some rewording would improve the flow.

Thank you for the detailed suggestions! The revised text (in lines 62-64) now reads as "**Symbiosis with arbuscular (AM) and ectomycorrhizal (EcM) fungi influence soil nutrient cycling and life strategies of host trees, with potential cascading effects on associated microbial and invertebrate communities [22,32–35].**" .

Line 96: "To address these research gaps" and "to test how tree diversity" seem repetitive, revise.

We deleted "To address these research gaps".

Line 156: stronger effects of what? Improve the writing.

The revised text (lines 153--155) now reads as **"Our results did not show consistently stronger effects on the abundance or taxonomic richness of lower than higher trophic levels across invertebrates and microorganisms (Figs 1-2; Figs. S11 & S13; Hypothesis (1a))."**

Line 158-159: I suggest being more specific and name also the groups that responded to the tree diversity aboveground.

The revised text (lines 155-157) now reads as **"Aboveground and soil-surface responses to tree diversity were limited to foliar fungi and soil-surface omnivores, providing no consistent support for stronger effects at lower trophic levels."**

Line 169-170: "Tree diversity enhanced fungal biomass"- add reference to the results.

We noticed that when listing the significant responses to tree diversity, we had missed soil fungi, which significantly increased with tree diversity ($F_{1,54}$ = 8.5, p = 0.005; Table S8; Fig. 1E). The revised text (in lines 157-161) now reads as **"In the belowground compartment, the strongest effects on abundance were observed on macrofauna predators ($F_{1,56}$=11.6, p = 0.003), followed by soil fungi ($F_{1,54}$ = 8.5, p = 0.005), nematode herbivores ($F_{1,56}$ = 7.9, p = 0.008) and fungivores ($F_{1,56}$ = 5.64, p = 0.02), as well as mesofauna predators ($F_{1,56}$ = 4.87, p = 0.03; Fig. 1E; Tables S7-S8)."** In other words, the statement that tree diversity enhanced fungal biomass was derived from our results, not from previous literature.

Line 171-173: Long and cryptic sentence, revise and be more specific, e.g. observed increases where, compared to what?
Line 174: Be more specific: increased fungal diversity- where? Also, this paragraph is very long; consider splitting it into two paragraphs.

To address the three comments above, we changed the structure of the paragraph. First, we divided it into two paragraphs: one mainly compares the results with the hypotheses and summarizes the findings, while the new paragraph focuses on the discussion. We kept most of the original text but simplified the sections that were suggested for revision. Now, it reads as **"In the belowground food web, the effects of tree diversity on soil fungi, fungivorous and herbivorous nematodes, as well as top predators may reflect bottom-up effects [18]. However, this pattern was not observed for lower-trophic-level meso- and macrofauna groups. The increase in fungivorous nematode abundance under higher tree diversity was linked to increased soil fungal biomass, whereas the lack of response in bacterivorous nematodes was consistent with unchanged bacterial biomass. Aligning with previous studies [49,62], fungal diversity increased with tree diversity, which may broaden trophic niches and support greater taxonomic richness of nematode fungivores [24]. Nematode herbivores probably benefited from greater tree productivity [63] and a larger host pool in tree polycultures. The abundance and diversity of mesofauna and macrofauna predators was likely linked to greater prey availability and diversity, such as nematodes [10,56,64]."** Find the text in lines 168-178.

Line 208: "This may explain " - what may explain?

Line 206-207: has repetitive sentences: "These findings contrast with our expectation (Hypothesis 2) that mixing mycorrhizal types would enhance microorganism and invertebrate abundance and diversity. " and " we found no such effects six years after establishing the MyDiv Experiment"

Line 216-218: This sentence seems to fit more in the beginning of the paragraph where the effects are discussed. Now the flow of the paragraph is the effects, the mechanisms and then the effects again.

We edited the text to address the three comments above together. First, we shortened the text to make it easier to follow. Second, we deleted the final sentence and instead moved the citation to the sentence: "Six years after establishing the MyDiv Experiment may not be long enough for such effects to emerge [10,19,63,73]". Now, it reads (lines 206-211) as **"These findings contrast with our expectation (Hypothesis 2) that mixing mycorrhizal types**

would enhance microorganism and invertebrate abundance and diversity. For abundance, this lack of support is consistent with the previously reported absence of synergistic effects on tree productivity in AM+EcM tree communities [63]. Six years after establishing the MyDiv Experiment may not be long enough for such effects to emerge [10,19,63,73], while Luo et al. [37] reported synergistic productivity effects in mature forests. ”

Line 226: “However, effects on taxonomic richness “ be more specific.

The revised text reads as **“Effects on taxonomic richness were limited to lower trophic levels, e.g., soil fungi and nematode herbivores (Fig. 2F). Taxonomic richness was highest in AM tree communities for total soil microbes ($F_{1,54}$ = 82.8, p < 0.001), soil fungi ($F_{1,54}$ = 79.2, p < 0.001), and nematode herbivores ($F_{2,54}$ = 4.2, p = 0.020), with no significant differences observed at higher trophic levels (Fig. 2F).”**

Line 235: What is meant here with “mycorrhizae“? It is a fungus + plant root, and I do not think this is meant here.

Yes, we only referred to mycorrhizal fungi. We changed the text (in lines 232-235) as follows: **“The elevated microbial and fungal taxonomic richness in AM tree communities was driven by the enrichment of saprotrophs, pathogens, and mycorrhizal fungi under AM-dominated tree assemblages (Fig. S5), in line with previous studies [75,76].”.**

Line 292: “positive effects of tree diversity on productivity“- clarify what is meant by productivity, biomass? In this case, I would ask, is the extent of damage by belowground predators sufficient to influence tree growth?

The comparison with tree growth was somewhat indirect, so we deleted that part and restricted the comparison to belowground predation. The text now reads as follows (in lines 281-285): **“Soil macrofauna predators increased in abundance with tree diversity only in EcM tree communities (Fig. S10B); their taxonomic richness increased in EcM and AM+EcM communities, but not in AM communities (Fig. S10D). These results are consistent with Yi et al [10], where enhanced belowground predation occurred only in EcM plots.”**

Line 295: “While the interaction between tree diversity and mycorrhizal type had no significant 295 effect on most trophic groups, “ this is already said above and repetitive here, revise.

We removed the repetitive part. The shortened sentence reads as follows (lines 287-280): **“Nematodes, with fast turnover and reproduction rates, and mobile macrofauna predators seemed to respond earlier to tree diversity with different mycorrhizal types, consistent with their strong responses to each factor individually (as discussed previously).”**

Line 304: The link with carbon storage seems to stretch these results a bit too far. Can it be supported with evidence?

Agreed. Linking with carbon storage is quite far. We removed it.

Line 383: "Our results are most applicable to broadleaf forests. "This should be better integrated within the text.

We changed the text (lines 375-378) as **"Moreover, while our findings may be applicable to broadleaf forests, many ectomycorrhizal tree species in European forests are conifers; therefore, future experiments that include coniferous EcM species as an additional treatment would broaden the applicability of these findings."**

Line 386: I do not think soil biodiversity is a black box anymore, there is lots of research and knowledge.

We deleted the statement of "soil biodiversity is a black box". The new sentence (in lines 378-381) is **"Still, this study integrates soil biodiversity with aboveground and soil surface communities, and contributes to our mechanistic understanding of above- and belowground community responses to tree diversity loss and shifts in mycorrhizal dominance."**

Line 391: Where the belowground biodiversity should be prioritized?
We agree that this statement was unclear and rephrased it (in lines 380-382) accordingly: "**We also highlight the need to consider belowground biodiversity in forest management decisions, representing ~59% of global biodiversity [12], yet currently being largely overlooked in monitoring and conservation schemes**".

Reviewer #2

Remarks to the Author:
This manuscript addresses an important and timely topic in forest biodiversity research by examining how tree diversity and mycorrhizal type jointly shape multitrophic biodiversity across aboveground and belowground compartments. The study benefits from the well-established MyDiv experiment and an impressive integration of microbial and invertebrate datasets across multiple trophic levels. The revised version has improved in clarity, methodological transparency, and ecological interpretation. Overall, the manuscript has been considerably strengthened after revision, and many of the reviewers' previous concerns have been adequately addressed. However, I still have some concerns.

Thanks a lot for the positive feedback and additional suggestions to improve our manuscript. We addressed your concerns point by point as described below.

1. The manuscript successfully integrates tree diversity, mycorrhizal type, and multitrophic biodiversity across aboveground and belowground compartments, which is undoubtedly valuable. However, many of the central ecological ideas underlying the study are already relatively well established, including: biodiversity effects on multitrophic communities, functional differences between AM and EcM systems, and stronger belowground sensitivity to resource heterogeneity and environmental filtering. In this sense, the study primarily represents a comprehensive empirical extension of existing frameworks rather than a major conceptual advance or the development of a substantially new ecological theory. While the scale and integration of the dataset are impressive, the manuscript may not yet provide the level of theoretical innovation or paradigm-shifting insight typically associated with this journal.

Thank you for highlighting the need to strengthen the novelty statements in the revised version of the manuscript. While we are happy to build on extensive ecological theory and empirical work (mostly in grasslands though), comparisons of above- vs. belowground biodiversity responses to tree diversity are exceptionally scarce, just as we stated in the Introduction (second paragraph). Moreover, the unprecedentedly comprehensive dataset presented here allowed us for the first time to test some of the main mechanisms of tree species richness and mycorrhizal type effects on above-belowground multitrophic communities. While addressing comments by the other reviewers (specifically reviewer #1), we made sure to sharpen our text and respective statements.

2. Although the SEM framework has been substantially improved, many of the proposed mechanisms remain indirectly inferred rather than directly demonstrated. For example, key interpretations involving resource partitioning, trophic propagation, litter heterogeneity, carbon transfer pathways, and bottom-up regulation, are ecologically plausible, but were not directly measured in this study. As a result, several mechanistic conclusions remain largely correlative and conceptual. This limits the strength of the broader ecological inferences that can be drawn from the experiment.

Thank you for highlighting the strength of our SEM approach, while also noting its caveats. To address this comment, we added outlook statements after the sections where these mechanisms are discussed.

In lines 132–152, we explain the contrasting above- and belowground responses to tree diversity in relation to dispersal limitation, resource stability, and trophic propagation. At the end of this section, we added the following sentence (in lines 150-152): **"Although the mechanisms we referred above are well established, their role in our study remains inferential and should be tested directly in future work."**

In lines 168–192, we explain the effects of tree diversity on multitrophic groups in belowground compartments in relation to bottom-up regulation and trophic propagation. At the end of this paragraph, we added the following outlook statement (in lines 189-192): **"To direct test the mechanisms proposed to explain the observed patterns, future studies should manipulate resource heterogeneity [67,68] and use stable-isotope analysis [69], isotope labeling, and molecular analyses of consumer tissues [70] and gut contents to trace resource transfer through the food web [71]. "**

Similarly, in lines 347–351, we clarified the interpretation of the SEM results by adding the following statement: **"Together, these results suggest that the effects of tree diversity and mycorrhizal type on microbial communities are mediated by canopy complexity and leaf C:N. Nevertheless, although these SEM pathways were grounded in established theory and supported by statistical associations, they remain inferential rather than directly causal and should be tested explicitly in future experiments."**

We acknowledge the reviewer's feedback, as the new text may inspire future experimental work and thus increase the impact of our paper.

3. A further limitation concerns the generality of the conclusions. The study is based on a single experimental site and relatively young forest stands (~6 years after establishment). Many key ecological processes discussed in the manuscript — including canopy differentiation, mycorrhizal complementarity, litter-mediated feedbacks, and long-term soil food-web restructuring — are likely to strengthen over longer temporal scales and across broader environmental contexts. Consequently, it remains unclear to what extent the observed patterns are generalizable across forest types, biogeographic regions, or mature

forest systems. While this limitation is acknowledged in the revised manuscript, it nonetheless reduces the broader predictive and conceptual scope.

Thank you for acknowledging the revisions we performed to address this comment. While the specific setting of the experiment can be seen as a limitation of the study, it is the only realistic way to perform such a detailed and comprehensive investigation of the mechanisms underlying multitrophic BEF relationships. Moreover, the MyDiv experiment is not just an artificial setup, but is an official compensation plantation of the Helmholtz Centre for Environmental Research - UFZ. Tree species selection and planting pattern largely reflects conditions in the closeby Neue Harth forest area that consists of reforestation sites of different ages on former open coal mining sites. We are thus confident that MyDiv very well resembles local reforestation sites and stages. While we discuss that tree community effects may change over time, the initial years of reforestation are particularly important for tree survival and biodiversity restoration.

Reviewer #3

Remarks to the Author:
The authors have made substantial revisions, resulting in a much clearer manuscript with improved justification of the experimental design and a more evidence-based interpretation of the results.

My previous comments have been addressed in full. However, on reading the revised manuscript, I think there remains some mismatch between the framing of the mycorrhizal type hypotheses and the strength of the evidence supporting interaction effects

The core finding that tree diversity exerts stronger effects on belowground than aboveground multitrophic biodiversity is now well supported and framed within a coherent mechanistic context. However, the treatment of mycorrhizal type effects remains less well supported, particularly given the absence of interaction effects between tree diversity and mycorrhizal type. This is important as it relates directly to one of the central hypotheses. The manuscript would benefit from more clearly reflecting that these drivers act largely independently (additive rather than synergistic effects). In this context, the title could more clearly reflect the lack of interaction effects, and the abstract should explicitly state that no synergistic effects between tree diversity and mycorrhizal type were detected, to better align the summary with the results.

We revised our title as **“Tree diversity and mycorrhizal type independently shape multitrophic biodiversity, with stronger effects belowground than aboveground”.**

We added the sentence (in lines 26-27) **“For most trophic groups, diversity responses were similar across mycorrhizal types”** to the abstract. The revised abstract now also indicates that no interactive effects of tree diversity and mycorrhizal type were found.

Given that the interaction hypothesis (#3) was not supported, the framing of hypotheses and conclusions could be more closely aligned with the strongest supported findings (main effects and mediation pathways).

We added the sentence (in lines 357-358): **“However, trophic-group responses to tree diversity were independent of mycorrhizal type.”** This directly addresses hypothesis 3, which was not supported by our results.

As suggested by the editors, we would like to retain our initial four hypotheses, because they reflect the objectives of the experimental design and the structure of our statistical analyses (i.e., Tables S4–S8, Fig. S10, and Fig. 3). We have therefore clarified the conclusion while keeping the overall hypothesis framework.

More clearly distinguishing which mechanistic pathways are directly supported by the analyses versus those that remain inferential would further strengthen the manuscript. Similarly, interpretations of belowground–aboveground asymmetry should be slightly tempered where they rely on untested mechanisms. For example, explanations based on dispersal limitation and resource stability are plausible but not directly evaluated in the current analysis.

Agreed! We paid extra attention to distinguishing the parts directly supported by our results from the inferential explanations.

For example, in the paragraph interpreting belowground–aboveground asymmetry, we tempered the wording at the start of the paragraph (in lines 132-133). It now reads: **"The results may partly reflect differences in dispersal limitation and resource stability between above- and belowground systems."**

We also added a sentence at the end of the explanation to make the inferential nature of these mechanisms explicit (in lines 150-152): **"Although the mechanisms referred to above are well established, their role in our study remains inferential and should be tested directly in future work."**

Additional caveat statements and outlook points were added. For more details, please see our response to Reviewer 2 (second comment).
